# Single-cell transcriptomics analysis reveals intratumoral heterogeneity and identifies a gene signature associated with prognosis of hepatocellular carcinoma

**DOI:** 10.1042/BSR20212560

**Published:** 2022-02-25

**Authors:** Jialu Liang, Wenhui Chen, Jianwen Ye, Chen Ni, Wenlong Zhai

**Affiliations:** 1 Department of Hepatobiliary and Pancreatic Surgery, The First Affiliated Hospital of Zhengzhou University, Zhengzhou, Henan 450052, P.R. China; 2 Key Lab of Digestive Organ Transplantation of Henan Province, Open and Key Laboratory of Hepatobiliary and Pancreatic Surgery and Digestive Organ Transplantation at Henan Universities, Zhengzhou Key Laboratory of Hepatobiliary and Pancreatic Disease and Organ Transplantation, Zhengzhou, Henan 450052, P.R. China

**Keywords:** Cell-cell communication, Hepatocellular carcinoma, Intratumoral heterogeneity, Single-cell RNA sequencing, Weighted gene co-expression network analysis

## Abstract

Hepatocellular carcinoma (HCC) tumors exhibit high heterogeneity. However, current understanding of tumor cell heterogeneity of HCC and the association with prognosis remains very limited. In the present study, we collected and examined tumor tissue from one HCC patient by single-cell RNA sequencing (scRNA-seq). We identified 5753 cells and 16 clusters including hepatocytes/cancer cells, T cells, macrophages, endothelial cells, fibroblasts, NK cells, neutrophils, and B cells. In six tumor cell subclusters, we identified a cluster of proliferative tumor cells associated with poor prognosis. We downloaded scRNA-seq data of GSE125449 from the NCBI-GEO as validation dataset, and found that a cluster of hepatocytes exhibited high proliferation activity in HCC. Furthermore, we identified a gene signature related to the proliferation of HCC cells. This gene signature is efficient to classify HCC patients into two groups with distinct prognosis in both TCGA and ICGC database cohorts. Our results reveal the intratumoral heterogeneity of HCC at single cell level and identify a gene signature associated with HCC prognosis.

## Introduction

Primary liver cancer (PLC) is the sixth most prevalent cancer and the third leading cause of cancer-related deaths worldwide. Hepatocellular carcinoma (HCC) is the most common type of liver cancer, accounting for 75–85% of PLC. Hepatitis B virus (HBV) infection, hepatitis C virus (HCV) infection, aflatoxin B1 and alcohol abuse are the major risk factors for HCC [1]. Despite significant advances in therapeutic strategies for HCC, the prognosis of HCC remains poor. It is widely accepted that HCC exhibits high heterogeneity [2,3]. However, current understanding of tumor cell heterogeneity of HCC remains very limited.

RNA sequencing (RNA-seq) technologies have been used to study the expression profile of HCC, which detect the gene expression of the sample as a whole [4]. However, the tumor tissues contain not only tumor cells but also endothelial cells, immune cells, fibroblasts, inflammatory cells, and extracellular matrix [5]. Therefore, it is difficult to investigate the tumor cell heterogeneity of HCC by RNA-seq technologies. To compensate for the disadvantages of RNA-seq technologies, single-cell RNA sequencing (scRNA-seq) has been widely used in the tumor [6]. scRNA-seq is a method that focused on the main characteristics of each cell subpopulation in the tissues [7]. Previous studies have shown that scRNA-seq is able to completely characterize cellular heterogeneity, identify known or unknown cell types, reveal cell subpopulations, and putative intercellular communication patterns [2,3,8].

In the present study, we employed scRNA-seq technology to explore intratumoral heterogeneity of HCC. We identified 5753 cells and 16 clusters in HCC including six cancer subclusters from one HCC patient. We found a proliferating tumor cluster which was associated with a worse prognosis. Furthermore, we identified a gene signature related to the proliferation in this tumor cluster, which was able to classify HCC into two groups with distinct prognosis in both TCGA and ICGC database cohorts.

## Methods

### Sample acquisition

Tumor tissue was collected at the time of surgical resection from a patient with HCC. The study was approved by the Ethics Committee of the First Affiliated Hospital of Zhengzhou University (2018-KY-83). Written informed consent was obtained from the enrolled patient.

### Preparation of single-cell suspensions

The fresh sample was rinsed with PBS and enzymatically dissociated using a tumor dissociation kit, the cell suspensions were filtered using a 70-μm filter and then centrifuged at 500 rpm for 6 min at 4°C. To prepare single-cell suspensions, pellets were washed twice and suspended in PBS with 0.5% bovine serum albumin (BSA, Sigma).

### Library preparation and sequencing

Single-cell transcriptomic amplification and library preparation were performed according to standard protocol of single-cell 3′ v2 or v3 (10x Genomics). The libraries were then pooled and sequenced on Illumina NovaSeq 6000 system following the manufacturer’s instructions.

### scRNA-seq data processing

Raw base call (BCL) files were demultiplexed and converted into FASTQ files, which were subsequently used to generate raw gene expression matrices using Cell Ranger Pipeline, with GRCh38 as a reference. Seurat R package (version 3.2.3) [9] was used to process raw gene expression matrices. Low quality cells (<200 genes/cell and >50% mitochondrial genes) were excluded. Data were log-normalized for subsequent analysis. The Seurat function ‘FindVariableFeatures’ was applied to identify the top 2000 highly variable genes (HVGs). The data were scaled using ‘ScaleData’ function and principal component analysis (PCA) and uniform manifold approximation and projection (UMAP) were carried out to analyze the scRNA-seq data. The ‘FindAllMarkers’ function was applied to find marker genes for each cell cluster (Supplementary Table S1). ‘DoubletFinder’ (version 2.0.3) [10] was employed to identify doublets in the data (Supplementary Figure S4). Clusters were annotated to known cell types according to canonical marker genes.

### Validation dataset

scRNA-seq data of GSE125449 were downloaded from the NCBI-GEO (https://www.ncbi.nlm.nih.gov/geo/). The validation set was based on 10x Genomics platform, which included 19 liver cancer tissues [11].

### Copy number variation analysis

InferCNV was used to perform copy number variation (CNV) analysis base on the normalized scRNA-seq gene expression matrices as previously described [12]. The endothelial cells and fibroblasts were designated as reference cells and background for analysis.

### Gene set variation analysis

The hallmark gene sets were downloaded from the Molecular Signature Database. The pathway activity for each cell was scored with gene set variation analysis (GSVA) methods as described previously [13].

### TCGA-LIHC and ICGC (LIRI-JP) data analysis

The 368 HCC mRNA expression data and clinical information from TCGA LIHC dataset, were downloaded from UCSC Xena (http://xena.ucsc.edu/) database. mRNA expression data and clinical information of another 240 HCC samples were obtained from the ICGC portal (https://dcc.icgc.org/projects/LIRI-JP). To evaluate the correlation between the expression of hepatocytes subcluster-specific marker genes and patient outcome, the marker genes (Supplemenntary Table S2) were identified for each cluster of hepatocytes using the ‘FindAllMarkers’ function in the Seurat package. HCC patients were further divided into ‘low expression’ and ‘high expression’ groups according to the average expression of hepatocytes subcluster-specific marker genes. The statistical analysis was performed by the ‘survival’ R package, and the ‘survfit’ function was used to create survival curves. The differences in survival curves were tested by ‘survdiff’ function.

### Cell–cell communication analysis

To investigate cell–cell interactions in HCC, the ‘CellChat’ R package was applied with default parameters [14].

### Weighted gene co-expression network construction

The genes, as selected as noise robust by OGFSC using default parameters [15], were picked for WGCNA. The WGCNA R package was applied to construct a gene co-expression network [16]. The topological overlap matrix (TOM) was constructed with softpower and was set to 9. Five gene modules were identified. The genes in the brown module were defined as proliferation-related gene signature for gene ontology (GO) analysis [17].

### Molecular subtype identification

An NMF clustering algorithm [18] was used to cluster the TCGA-LIHC cohort and ICGC cohort according to the expression of the proliferation-related gene signature. The cophenetic, dispersion and silhouette indicators were used to determine the optimal clustering number. The number of groups k was chosen as 2.

### Statistical analysis

The data were evaluated by statistical analysis with R (version 4.0.2) software.

## Results

### Cell typing of HCC

**Figure 1:**
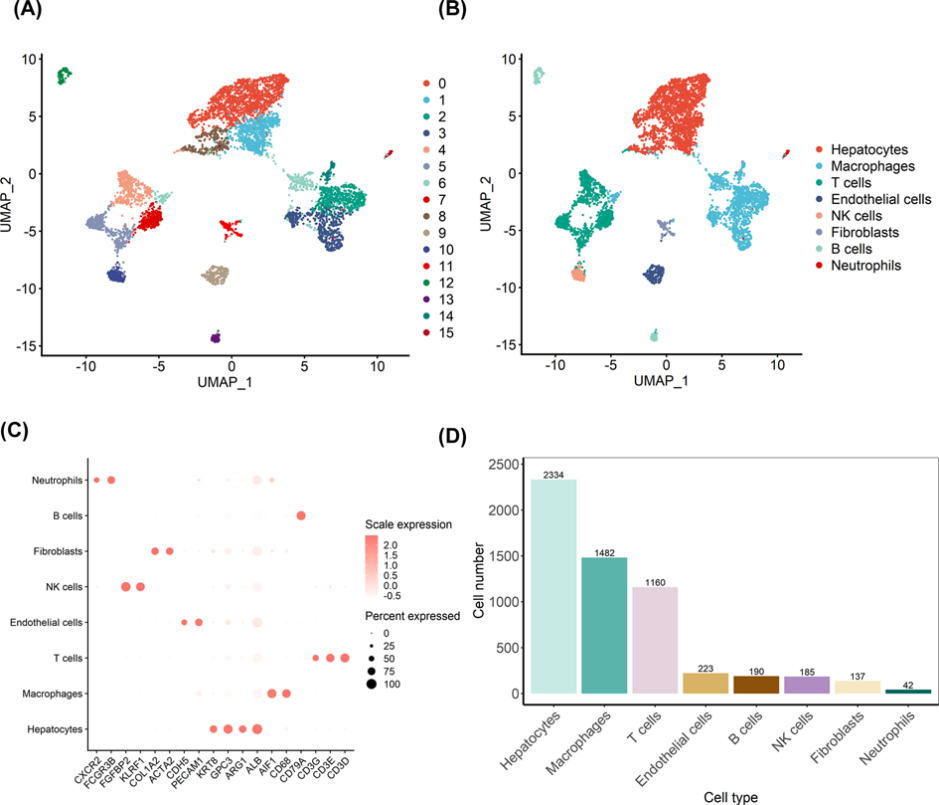
Identification of cell types with single-cell transcriptomics analysis in HCC sample (**A**) UMAP visualization in all the cells displayed with different colors for clusters. (**B**) Eight major cell types in HCC. (**C**) Marker genes of eight major cell types identified in the present study. (**D**) A bar plot showing the cell number of identified cell types in HCC.

To explore the cellular diversity and microenvironment composition in HCC, we generated single-cell RNA-seq from an HCC tumor tissue. After quality control, we identified total 5753 cells from HCC. As shown in Figure 1A,B, we identified 16 cell clusters corresponding to eight cell types, including hepatocytes/cancer cells (Cluster 0,1,8; ALB, ARG1, GPC3, KRT8), macrophages (Clusters 2,3,6,14; CD68, AIF1), T cells (Cluster 4, 5 and 7; CD3D, CD3E, CD3G), NK cells (Clusters 10; FGFBP2 and KLRF1), fibroblasts (Clusters 11; COL1A2, ACTA2), endothelial cells (Clusters 9; CDH5, PECAM1), neutrophils (clusters 15; FCGR3B, CXCR2) and B cells (Cluster 12 and 13; CD79A) (Supplementary Figure S1). To explore large-scale chromosomal aberrations in hepatocytes, we conducted CNV analyses. Our results showed that compared with reference cells (endothelial cells, fibroblasts), DNA insertions in hepatocytes mainly occurred in chromosomes 1, 2, 3, 6, 7, and 20, while deletions were present in chromosomes 1, 4, 5, 12, 14, 15 and 19 (Supplementary Figure S2).

### Single-cell transcriptomics reveal intratumoral heterogeneity in hepatocytes

**Figure 2:**
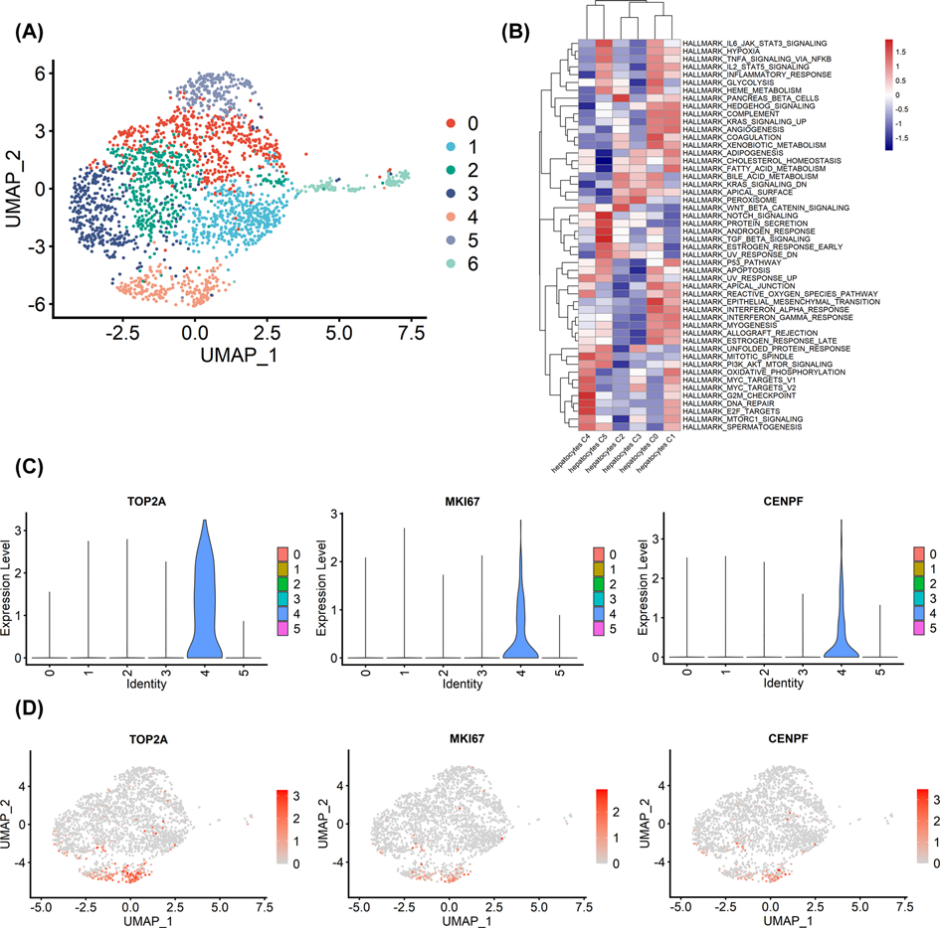
Heterogeneity of hepatocytes in HCC (**A**) UMAP plot showing seven subclusters of the hepatocytes. Note that cluster 6 consisted mainly of lower quality cells and was discarded for further analyses. (**B**) Pathway activities were scored in hepatocytes subclusters using GSVA. (**C**) Violin plots showing the expression of representative proliferation marker genes across the hepatocytes subclusters. (**D**) Expression levels of representative proliferation marker genes in each subcluster were plotted on to the UMAP map.

To reveal tumor heterogeneity in HCC, we performed the subcluster analysis for hepatocytes. A total of 2334 hepatocytes were analyzed, which were clustered into six subclusters (Figure 2A). Because cluster 6 consisted mainly of lower quality cells, it was discarded for further analyses. Our results found that proliferative genes, such as MKI67, TOP2A, CENPF, were enriched in hepatocytes C4 (Figure 2C,D). In addition, GSVA showed that proliferation biological pathways, such as mitosis, G_2_/M checkpoint, and E2F targets, were enriched in hepatocytes C4 (Figure 2B). We found that genes for hepatocytes C5 were related to notch signaling, protein secretion, and TGF-β signaling. Genes for hepatocytes C2 were mainly enriched in pancreas β-cells and wnt/β-catenin signaling. Functions of genes specific for hepatocytes C0 and C1 were mainly related to kras signaling, complement, angiogenesis, and epithelial–mesenchymal transition. Specifically, genes for hepatocytes C3 were enriched in peroxisome. In validation dataset, we identified seven cell types, which included hepatocytes, macrophages, T cells, fibroblasts, endothelial cells, epithelial cells and B cells. We performed the subcluster analysis for hepatocytes in validation dataset. We also found a cluster of hepatocytes exhibited high proliferation activity, consistent with the above results (Supplementary Figure S3). In conclusion, these data reflected the intratumoral heterogeneity of HCC. We found that hepatocytes C4 exhibited high proliferation activity, so this cluster cells were considered as malignant proliferating cells.

### Prognostic role of hepatocytes subclusters in TCGA LIHC cohort and ICGC cohort

**Figure 3:**
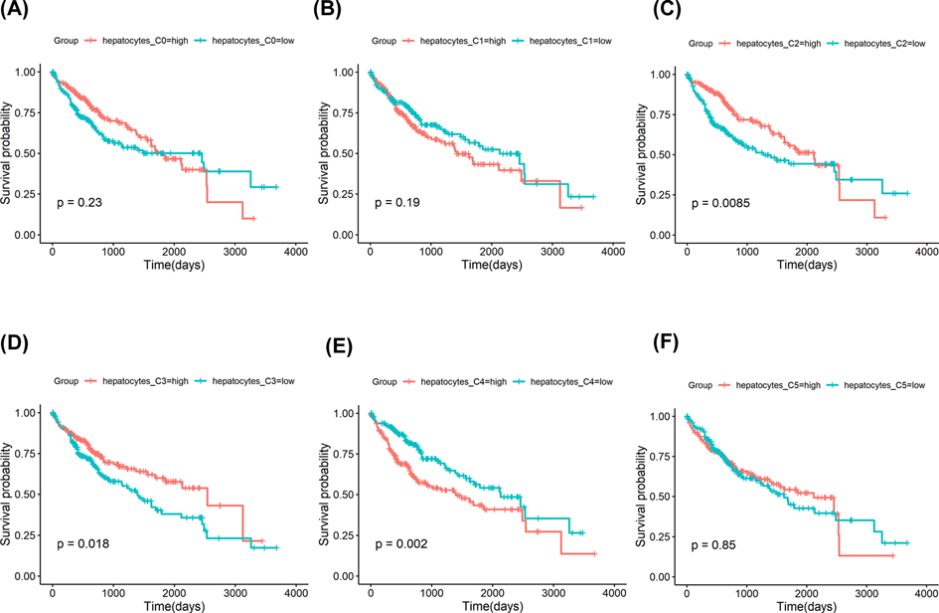
Prognostic role of hepatocytes subclusters identified by scRNA-seq in TCGA LIHC cohort (**A**) Survival curves for patients with HCC, stratified for the average expression of specific marker genes in hepatocytes C0. (**B**) Survival curves for patients with HCC, stratified for the average expression of specific marker genes in hepatocytes C1. (**C**) Survival curves for patients with HCC, stratified for the average expression of specific marker genes in hepatocytes C2. (**D**) Survival curves for patients with HCC, stratified for the average expression of specific marker genes in hepatocytes C3. (**E**) Survival curves for patients with HCC, stratified for the average expression of specific marker genes in hepatocytes C4. (**F**) Survival curves for patients with HCC, stratified for the average expression of specific marker genes in hepatocytes C5.

**Figure 4:**
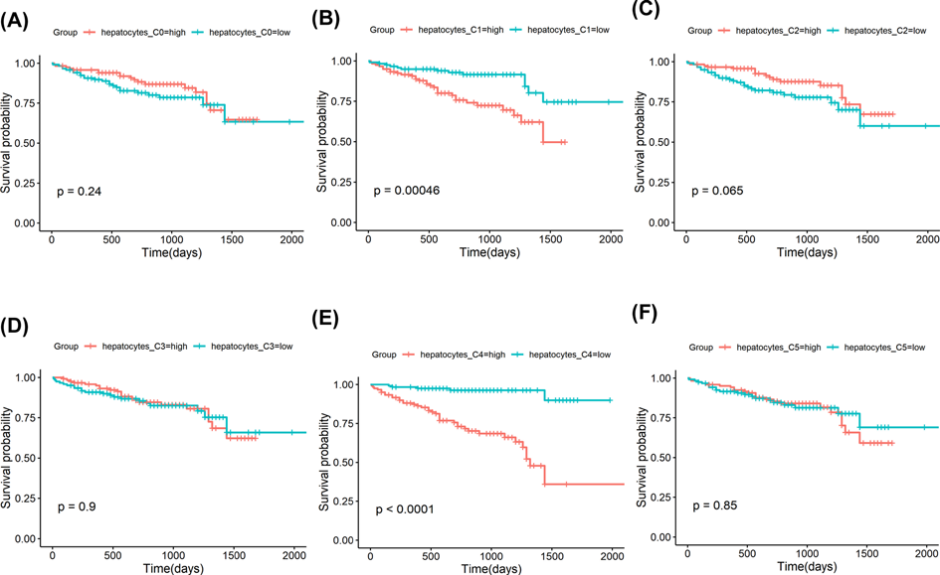
Prognostic role of hepatocytes subclusters identified by scRNA-seq in ICGC (LIRI-JP) cohort (**A**) Survival curves for patients with HCC, stratified for the average expression of specific marker genes in hepatocytes C0. (**B**) Survival curves for patients with HCC, stratified for the average expression of specific marker genes in hepatocytes C1. (**C**) Survival curves for patients with HCC, stratified for the average expression of specific marker genes in hepatocytes C2. (**D**) Survival curves for patients with HCC, stratified for the average expression of specific marker genes in hepatocytes C3. (**E**) Survival curves for patients with HCC, stratified for the average expression of specific marker genes in hepatocytes C4. (**F**) Survival curves for patients with HCC, stratified for the average expression of specific marker genes in hepatocytes C5.

We investigated the clinical value of the hepatocytes subclusters detected in our present study. In TCGA cohort, hepatocytes C0, C1, and C5 were not associated with the survival outcome of HCC patients (Figure 3A,B,F). The hepatocytes C3 and C2 were associated with a favorable survival outcome (Figure 3C,D). In contrast, hepatocytes C4 had a worse prognosis (Figure 3E). We also explore the clinical value of the hepatocytes subclusters in ICGC cohort. We found that hepatocytes C0, C2, C3, and C5 were not associated with the survival outcome of HCC patients (Figure 4A,C,D,F). The hepatocytes C1 were associated with a worse survival outcome (Figure 4B). Notably, hepatocytes C4 had a worse prognosis (Figure 4E), consistent with the results in TCGA cohort. In both TCGA and ICGC database cohorts, hepatocytes C4 were associated with a worse prognosis, which may be due to high proliferation activity.

### Cell–cell interactions between hepatocytes C4 and other cell types in HCC

**Figure 5:**
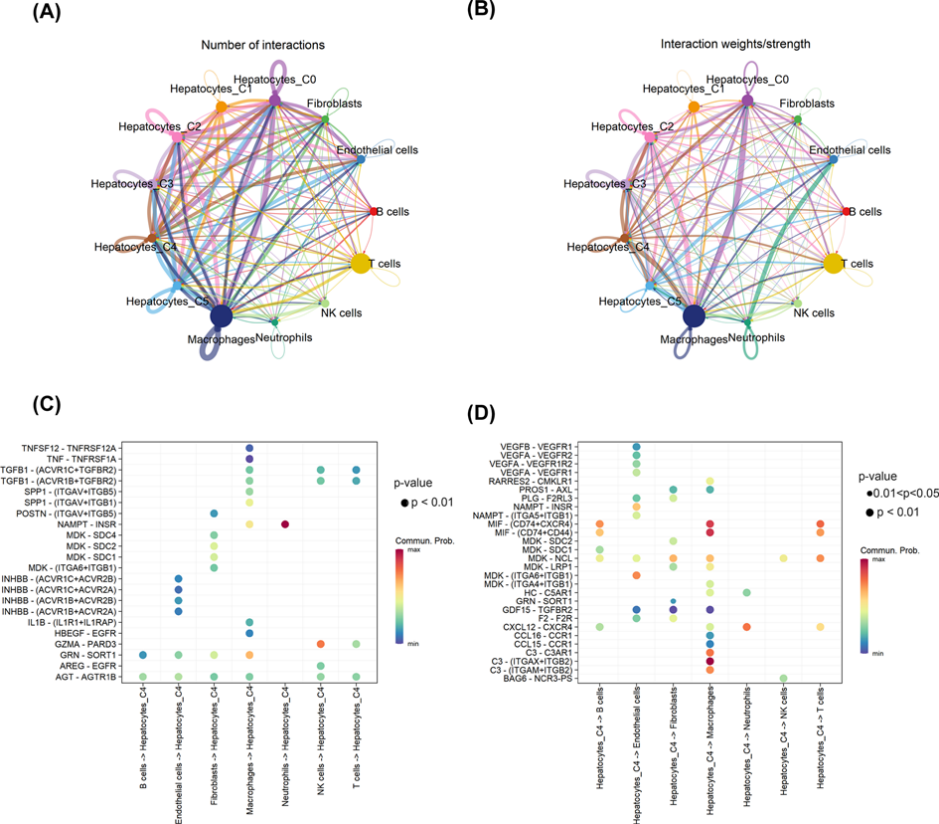
Cell–cell interactions in HCC (**A**) Circle plot showing the intercellular communication between major cell types in HCC. (**B**) Circle plot showing the interaction strength between major cell types in HCC. (**C**) The ligand–receptor pairs between B cells/endothelial cells/fibroblasts/macrophages/neutrophils/NK cells/T cells and hepatocytes C4. (**D**) The ligand–receptor pairs between hepatocytes C4 and B cells/endothelial cells/fibroblasts/macrophages/neutrophils/NK cells/T cells.

To systematically examine the interactions between hepatocytes C4 and other cell types in HCC, we used ‘CellChat’ to conduct a cell–cell communication network (Figure 5). This tool is able to analyze the intercellular communication networks from scRNA-seq data. VEGFA–VEGFR2/VEGFR1/VEGFR1R2 interactions were highly expressed between hepatocytes C4 and endothelial cells (Figure 5D), which suggested that hepatocytes C4 may play a crucial role in HCC angiogenesis. In addition, CD74–MIF interaction was highly expressed between hepatocytes C4 and macrophages/B cells/T cells in HCC (Figure 5D). CD74 has been reported to promote tumor cell growth through interaction with MIF [19]. These results suggest that CD74–MIF interaction may play an important role in the development of HCC and might serve as an effective target for HCC treatment.

Chemokines play a critical role in orchestrating the recruitment and positioning of myeloid cells within the tumor microenvironment [20]. We found that CCR1–CCL15/CCL16 interactions were highly expressed between hepatocytes C4 and macrophages (Figure 5D). CXCL12–CXCR4 interaction was highly expressed not only between hepatocytes C4 and macrophages, but also in B cells/neutrophils/T cells (Figure 5D). C3-C3AR1/ITGB2 interactions were highly expressed between hepatocytes C4 and macrophages (Figure 5D). INHBB-related interactions (INHBB-ACVR1B/ACVR1C/ACVR2A/ACVR2B) were highly expressed between hepatocytes C4 and endothelial cells (Figure 5C).

### A proliferating gene signature in hepatocytes C4 identified by WGCNA

**Figure 6:**
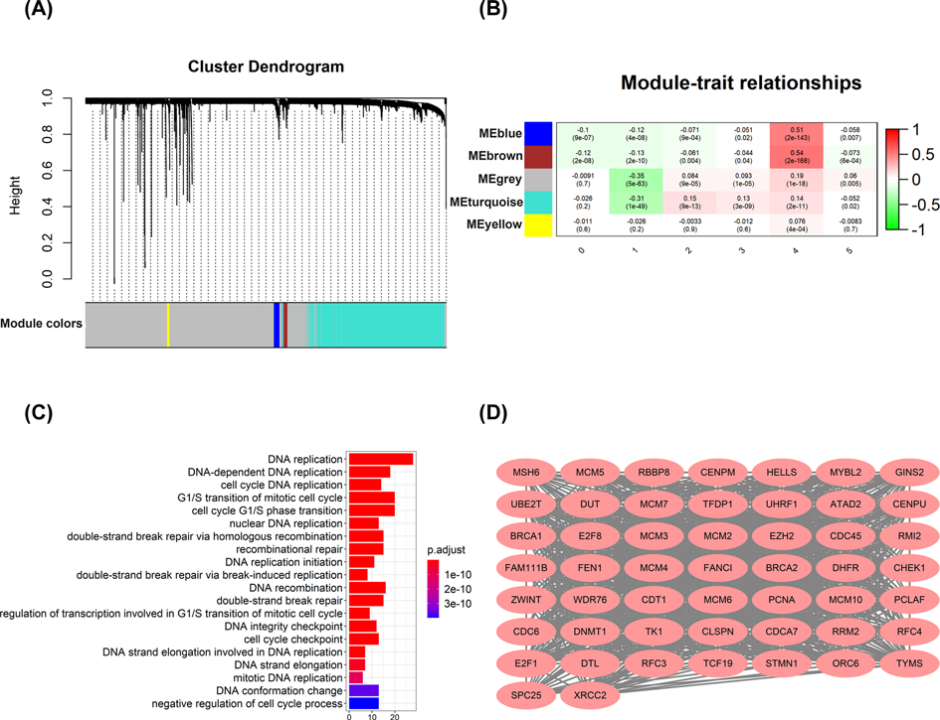
Identification of modules associated with hepatocytes C4 (**A**) Hierarchical cluster tree showing co-expression modules identified by WGCNA. (**B**) Correlation of gene modules with hepatocytes subclusters. Content in each cell represents the correlation value (first row) and the *P*-value (second row). (**C**) Top 20 enriched GO terms based on genes within the brown module. (**D**) The genes in brown module.

Next, we applied WGCNA to identify the modules composed of a group of highly correlated genes. The genes in modules could represent the biological functions of hepatocyte subclusters. WGCNA identified five gene modules (Figure 6A). Notably, we found that the brown module was most correlated with hepatocytes C4 (Figure 6B). The genes in the brown module were defined as proliferation-related gene signature because hepatocytes C4 showed high proliferation activity. Next, we wanted to know whether the genes in brown module were significantly enriched in key gene ontologies. We then performed GO analysis for genes in the brown module to explore the biological functions (Figure 6C). The results showed that genes were enriched for cell cycle-related processes, such as DNA replication, cell cycle checkpoint, and G_1_/S transition of mitotic cell cycle. Finally, we exported the genes in brown module to visualize the gene network (Figure 6D).

### The proliferating gene signature discriminated HCC into two distinct subgroups

**Figure 7:**
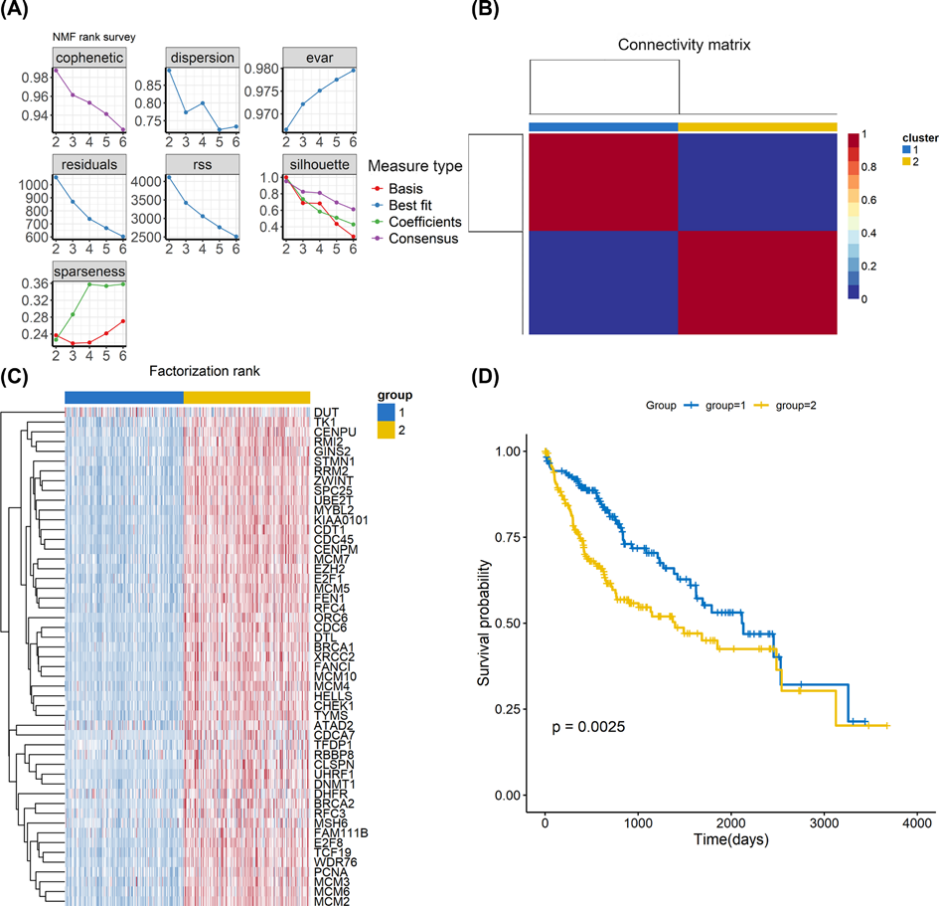
TCGA LIHC data analyses based on the proliferation-related gene signature (**A**) The relationship among cophenetic, dispersion, evar, residuals, rss, silhouette and sparseness coefficients with respect to number of clusters. (**B**) Consensus map of NMF clustering. (**C**) Heatmap showing the proliferation-related gene signature genes between the two groups. (**D**) Kaplan–Meier survival analysis of tumor samples grouped in (C).

**Figure 8:**
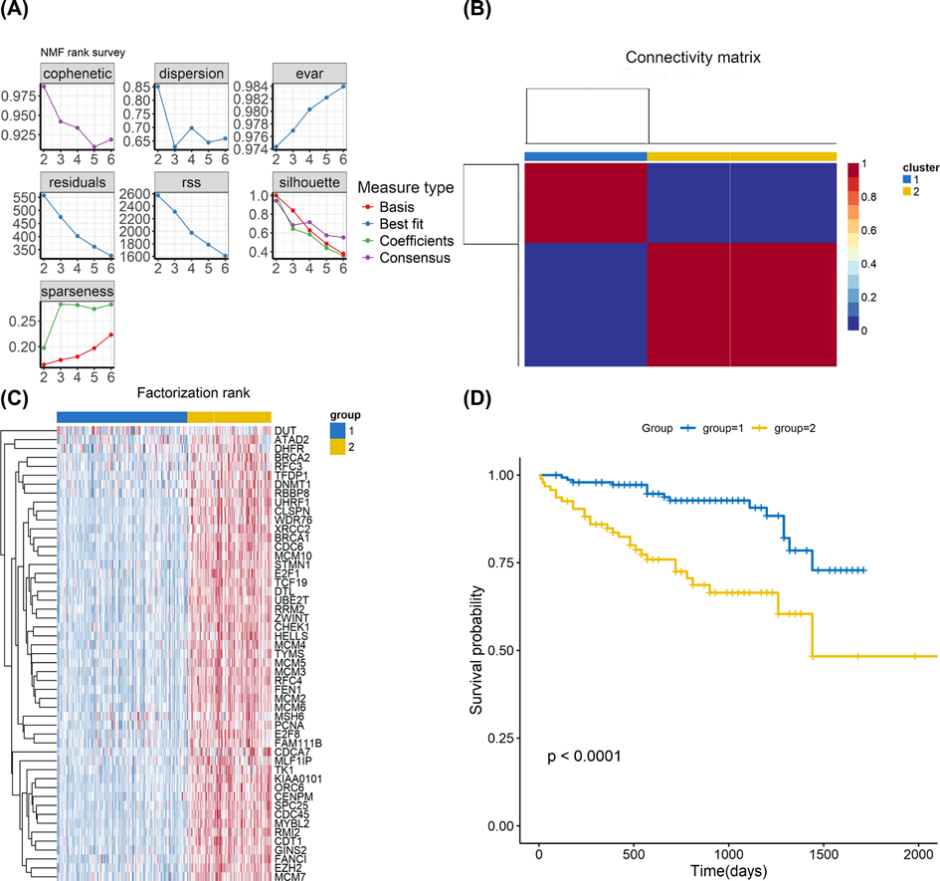
ICGC (LIRI-JP) data analyses based on the proliferation-related gene signature (**A**) The relationship among cophenetic, dispersion, evar, residuals, rss, silhouette and sparseness coefficients with respect to number of clusters. (**B**) Consensus map of NMF clustering. (**C**) Heatmap showing the proliferation-related gene signature genes between the two groups. (**D**) Kaplan–Meier survival analysis of tumor samples grouped in (C).

Using the proliferation-related gene signature, we identified two distinct subtypes (group 1: *n*=178 and group 2: *n*=–190) in TCGA cohort by NMF analysis (Figure 7A,B). The heatmap showed the expression patterns of proliferation-related gene signature genes between two groups (Figure 7C). We further explored the prognosis of two groups. The results showed that group 1 had a better prognosis than group 2 (Figure 7D). We also identified two distinct subtypes (group 1: *n*=146 and group 2: *n*=94) in ICGC cohort by NMF analysis (Figure 8A,B). The heatmap showed the same expression patterns of proliferation-related gene signature genes between two groups as in TCGA cohort (Figure 8C). In survival analysis, the results showed that group 1 had a better prognosis than group 2 (Figure 8D), suggesting that classification based on the gene expression profiles of proliferation-related genes could be used to predict HCC prognosis.

## Discussion

Heterogeneity is a typical feature of tumors, which leads to ineffective treatment. It is widely believed that HCC tumors exhibit high heterogeneity, leading to different responses to the same treatment among patients. However, little is known about tumor cell heterogeneity of HCC. Hence, we should pay more attention to exploring the intratumoral heterogeneity in HCC. In the present study, we explored intratumoral heterogeneity of HCC via scRNA-seq. Strikingly, we found that hepatocytes C4 exhibited high proliferation activity and this cluster was associated with a poor prognosis. In addition, we systematically examined the interactions between hepatocytes C4 and other cell types in HCC, and found that several interactions, such as INHBB related interactions, C3-C3AR1/ITGB2 interactions, may play an important role in the tumorigenesis of HCC.

We also employed WGCNA and identified five gene modules. We found that the brown module was most correlated with hepatocytes C4. The genes in the brown module were defined as the proliferation-related gene signature. Finally, the NMF algorithm was performed to classify HCC into two groups in both TCGA and ICGC cohorts according to the expression patterns of the proliferation-related gene signature. The survival analysis showed that patients in group 1 had a better prognosis than patients in group 2 in both TCGA and ICGC database cohorts. These results improve our understanding of intratumoral heterogeneity in HCC and help identify novel targets for HCC therapy.

Previous studies have suggested that some cancers contain a small population of cells with high proliferation capacity, which are considered to be related to tumor recurrence, metastasis, and chemoresistance [21]. Through scRNA-seq analysis, we identified a cluster of proliferative tumor cells (hepatocytes C4), which had significant levels of KI67, TOP2A, and CENPF. In validation dataset, we found a cluster of hepatocytes with high proliferation activity in HCC, consistent with the above results. KI67 is a well-known proliferation marker for the evaluation of cell proliferation [22]. KI67 expression in HCC is associated with the grade of differentiation and could be used as a prognostic indicator of HCC [23]. TOP2A has been reported to be up-regulated in HCC and could be used as a biological indicator to predict HCC prognosis [24]. CENPF associates with the centromere–kinetochore complex and influences cell proliferation and metastasis in HCC [25]. In addition, GSVA results showed that proliferation biological pathways, such as mitosis, G_2_/M checkpoint, and E2F targets, were enriched in hepatocytes C4. Notably, we found that hepatocytes C4 was associated with a worse prognosis in both TCGA and ICGC database cohorts. These results provide clues for targeting and eradicating proliferative tumor cells in HCC. In addition, our data demonstrate the feasibility of scRNA-seq in detecting rare cell subtypes in HCC.

Cross-talk between tumor and stromal/immune cells plays a key role in the modulation of tumor microenvironment, and immunotherapy is a promising approach for malignant tumors [26–28]. Therefore, we used the R package ‘CellChat’ to explore the cell–cell interactions between hepatocytes C4 and other cell types in HCC. CXCL12–CXCR4 axis plays a critical role in tumor cell survival, metastasis, and immune cell migration. Binding of CXCL12 to CXCR4 on tumor cells can inhibit the apoptosis of tumor cells and promote the epithelial-to-mesenchymal transition [29]. CXCL12 secreted by hepatocytes C4 interacts with CXCR4, which is expressed in B cells/macrophages/neutrophils/T cells. These results suggest that CXCL12 can influence the recruitment of immune cells. INHBB-related interactions (INHBB-ACVR1B/ACVR1C/ACVR2A/ACVR2B) were highly expressed between hepatocytes C4 and endothelial cells, and may play a crucial role in the angiogenesis of HCC. Indeed, INHBB has been reported to be associated with HCC metastasis [30]. However, the function and mechanism of INHBB-related interactions between hepatocytes and endothelial cells in HCC need further studies. C3 produced by hepatocytes C4 interacts with C3AR1/ITGB2, which is expressed in macrophages. In a clear-cell renal cell carcinoma murine model, C3 has been proven to be associated with tumor-associated macrophages (TAMs) infiltration and tumor growth [31]. Our results suggest that C3-C3AR1/ITGB2 interactions could serve as a promising therapeutic target to improve the efficacy of therapies, including immunotherapies for HCC by favorably modulating TAM function.

To further explore the potential roles of hepatocytes C4 in HCC, WGCNA was used to identify a proliferation-related gene signature and five modules were generated. we found that the brown module was most correlated with hepatocytes C4. The genes in the brown module were defined as the proliferation-related gene signature. Molecular phenotype identification can be used to optimize diagnosis and treatment strategies and promote the development of precision medicine. Therefore, the proliferation-related gene signature identified by WGCNA may be explored as new molecular targets for HCC. Zhang et al. classified HCC into four independent subtypes according to hypoxia-related genes [32]. In addition, Chen et al. classified HCC into three subclasses according to metabolism-related genes [33]. These subtypes encompass HCC heterogeneity and provide useful clinical information. In the present study, we profiled the expression of proliferation-related gene signature in HCC patients and identified two groups associated with differences in survival in both TCGA and ICGC database cohorts, suggesting that classification based on the gene expression profiles of proliferation-related genes could be used to predict the survival of HCC patients.

We acknowledge a limitation of the present study that our results are based on bioinformatics analysis, which should be validated by functional studies. In conclusion, our results help elucidate the intratumoral heterogeneity in HCC and develop novel treatment strategies for HCC.

## Supplementary Material

Supplementary Figures S1-S4 and Tables S1-S2

## Data Availability

All data are available from the corresponding authors upon reasonable request.

## Abbreviations

BCL: raw base call
CNV: copy number variation
GO: gene ontology
GSVA: gene set variation analysis
HBV: hepatitis B virus
HCC: hepatocellular carcinoma
HCV: hepatitis C virus
HVGs: highly variable genes
ICGC: International Cancer Genome Consortium
MIF: macrophage migration inhibitory factor
NK cells: natural killer cells
OGFSC: optimal gene filtering for single-cell data
PCA: principal component analysis
PLC: primary liver cancer
RNA-seq: RNA sequencing
scRNA-seq: single-cell RNA sequencing
TAM: tumor-associated macrophage
TCGA: The Cancer Genome Atlas
TOM: topological overlap matrix
UMAP: uniform manifold approximation and projection
WGCNA: weighted correlation network analysis
