# Supplementary material for: Single-cell transcriptomics analysis reveals intratumoral heterogeneity and identifies a gene signature associated with prognosis of hepatocellular carcinoma
Source: Biosci Rep. 2022 Feb 25;42(2):BSR20212560. doi: 10.1042/BSR20212560 (PMC8881646; doi:10.1042/BSR20212560)
Supplement: Supplementary Figures S1-S4 and Tables S1-S2 [file BSR-2021-2560_supp.pdf]

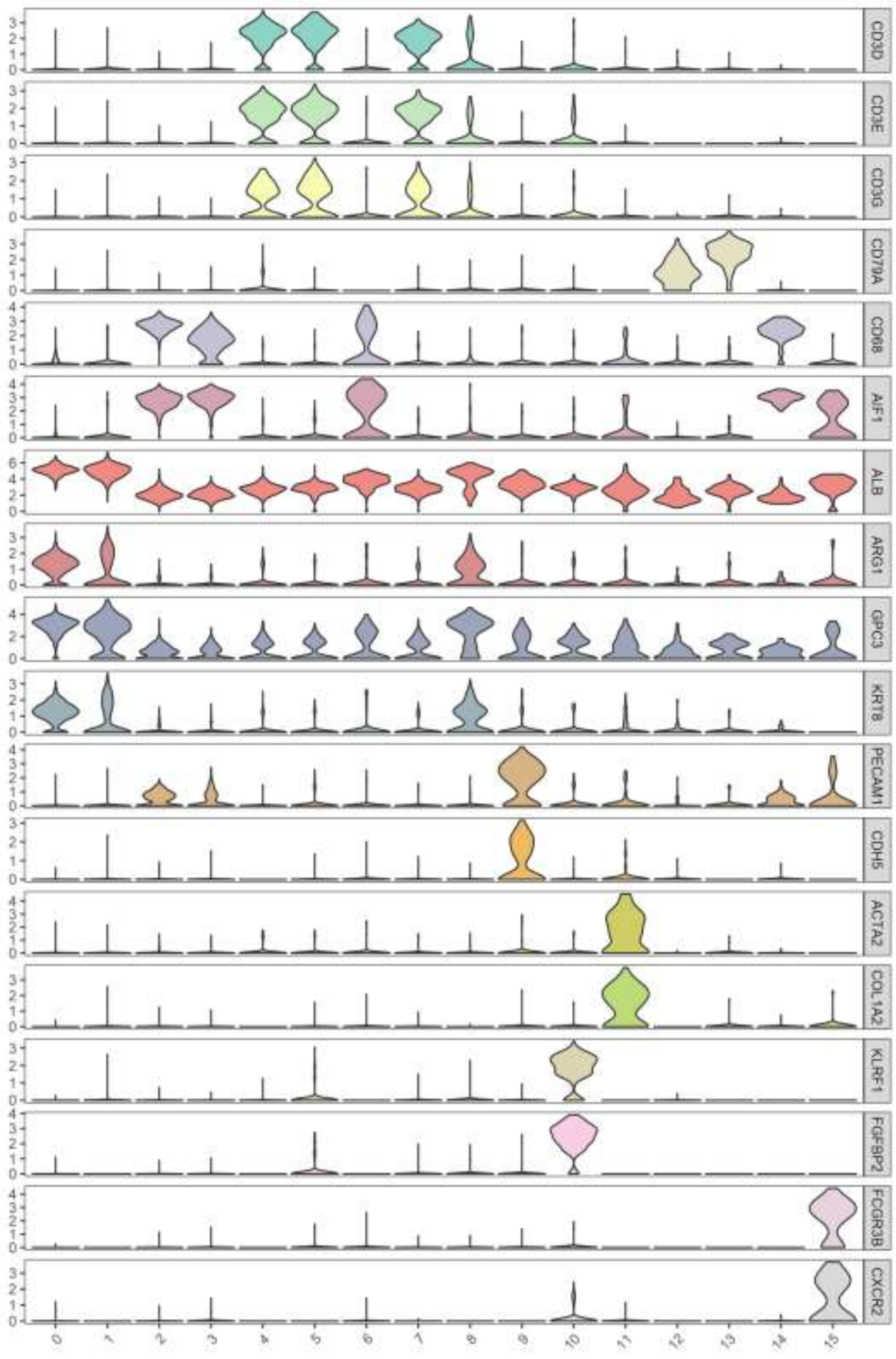

Figure S1. Violin plots showing the relative expression levels of marker genes across the clusters identified in HCC.

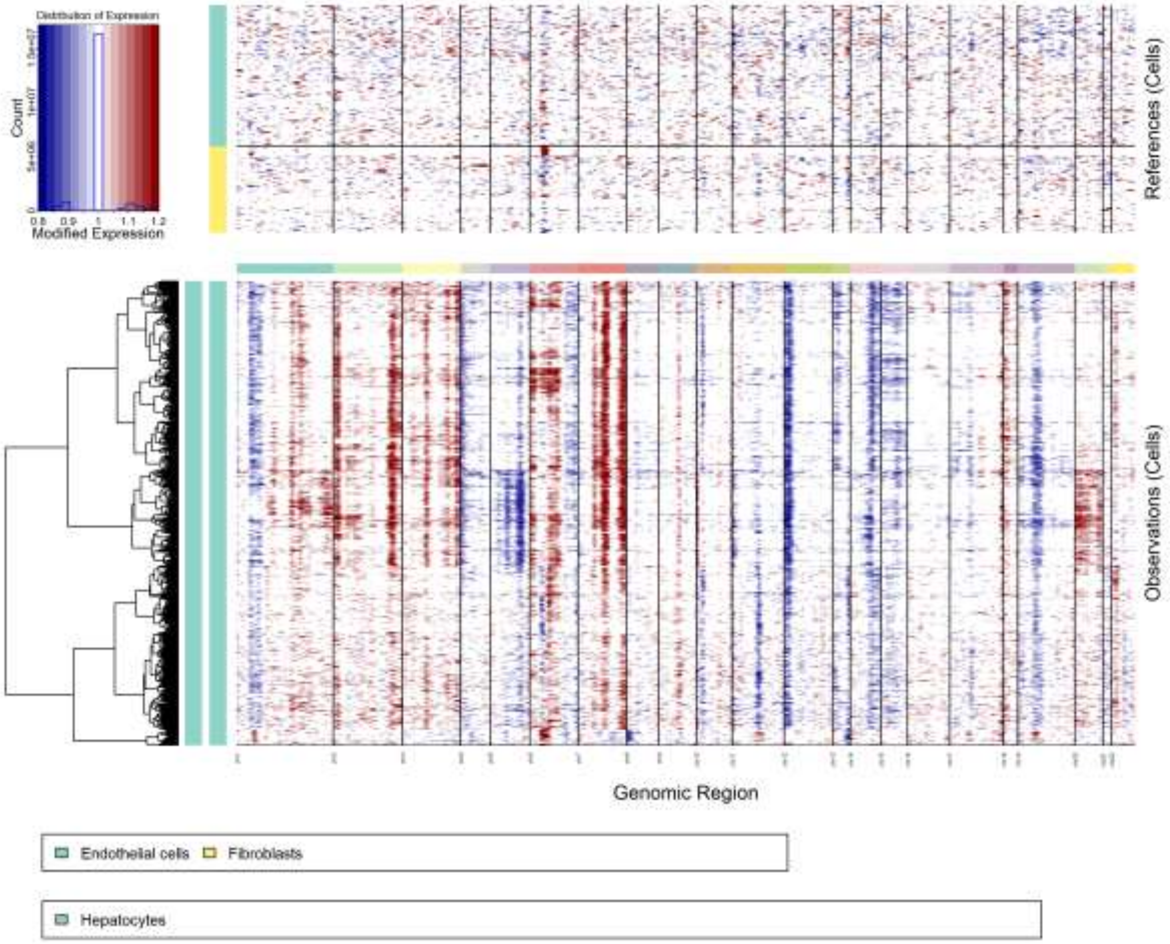

Figure S2. Copy number variation in hepatocytes. Endothelial cells and fibroblasts were used as reference. Red represented overexpression and blue represented low expression.

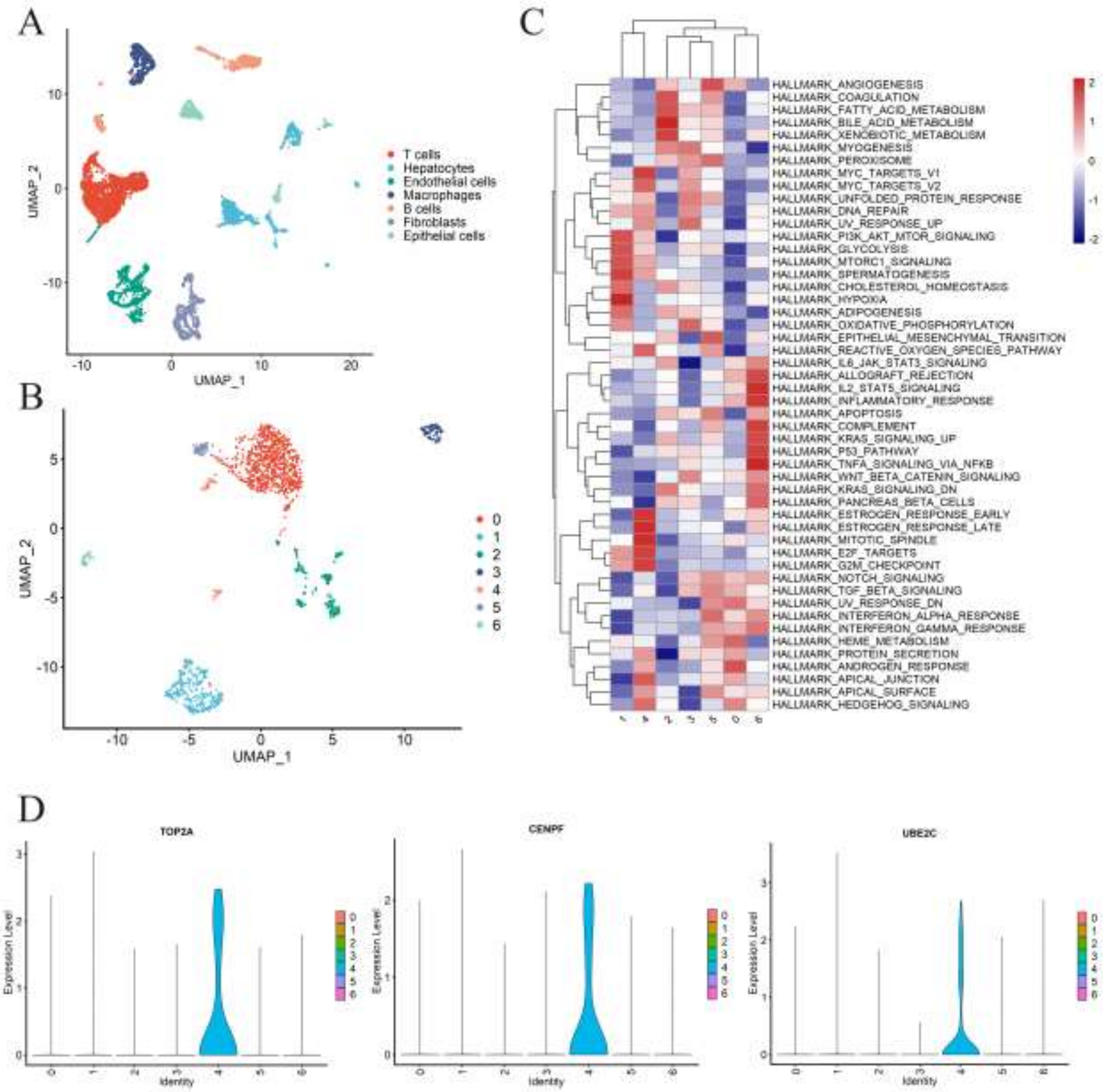

Figure S3. Heterogeneity of hepatocytes in validation dataset. (A) Seven major cell types in GSE125449. (B) UMAP plot showing seven subclusters of the hepatocytes. (C) Pathway activities were scored in hepatocytes subclusters using GSVA. (D) Violin plots showing the expression levels of representative proliferation marker genes across the hepatocytes subclusters.

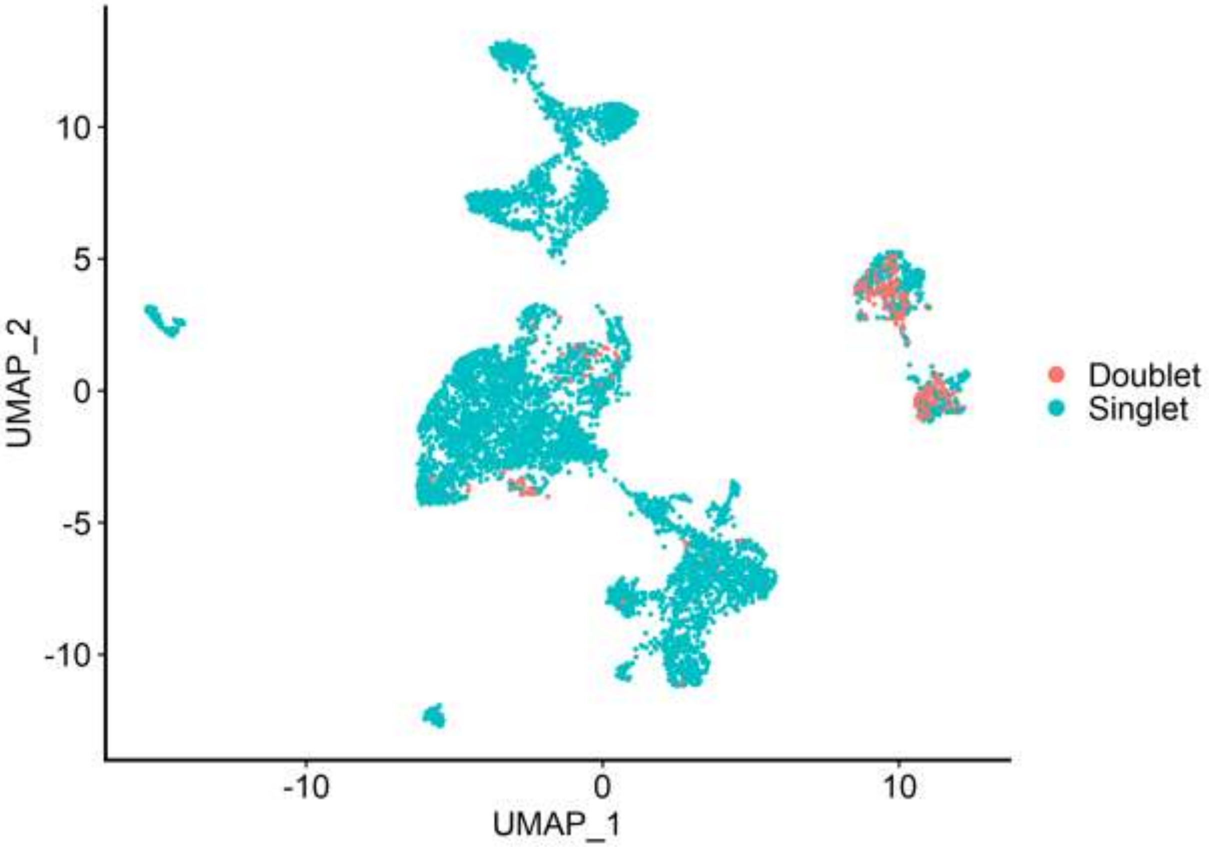

Figure S4. UMAP plot of single and doublet detection in HCC.

Table S1. Marker genes of each cluster identified in HCC.

gene	p_val	avg_logFC	pct.1	pct.2	p_val_adj	cluster
IGFBP1	0	1.921849717	0.989	0.702	0	0
PCK1	0	1.626003751	0.818	0.096	0	0
MEG3	0	1.618372899	0.805	0.118	0	0
RBP4	0	1.552097509	0.996	0.778	0	0
XIST	0	1.501829774	0.875	0.056	0	0
IGFBP2	0	1.44903899	0.842	0.324	0	0
SCD	0	1.448708909	0.959	0.395	0	0
CYP2E1	0	1.416684161	0.946	0.378	0	0
ASGR1	0	1.414118929	0.959	0.236	0	0
APOB	0	1.367476388	0.995	0.683	0	0
CP	0	1.365436773	0.981	0.465	0	0
MLXIPL	0	1.362591719	0.894	0.085	0	0
AGXT	0	1.360145518	0.977	0.42	0	0
AZGP1	0	1.359474598	0.969	0.363	0	0
AHSG	0	1.338087699	0.994	0.743	0	0
APOA1	0	1.337635418	0.994	0.871	0	0
FMO5	0	1.324312398	0.934	0.193	0	0
ALB	0	1.302649148	1	0.992	0	0
CPS1	0	1.271623251	0.828	0.175	0	0
APOC3	0	1.26957107	0.985	0.683	0	0
ITIH3	0	1.2627166	0.912	0.2	0	0
VTN	0	1.248593535	0.974	0.398	0	0
C3	0	1.242628702	0.976	0.419	0	0
ITIH1	0	1.242619204	0.971	0.326	0	0
C2	0	1.239767461	0.963	0.379	0	0
APOH	0	1.239727909	0.984	0.614	0	0
AGT	0	1.228194838	0.981	0.521	0	0
AMBP	0	1.182128477	0.984	0.66	0	0
GPC3	0	1.174395992	0.973	0.602	0	0
FGL1	0	1.166107898	0.99	0.693	0	0
APOM	0	1.162539633	0.972	0.602	0	0
KCNQ1OT1	0	1.147798186	0.823	0.204	0	0
ORM1	0	1.143647259	0.931	0.333	0	0
F10	0	1.141433214	0.927	0.215	0	0
SLC7A2	0	1.1367101	0.901	0.102	0	0
ONECUT2	0	1.135329241	0.898	0.129	0	0
TMEM176A	0	1.133734586	0.966	0.524	0	0
FGA	0	1.130208916	0.899	0.186	0	0
DSP	0	1.123445969	0.883	0.1	0	0
RARRES2	0	1.119598957	0.96	0.414	0	0
TTR	0	1.114476872	0.919	0.336	0	0
TFR2	0	1.106848844	0.912	0.128	0	0

LEPR	0	1.105426464	0.902	0.173	0	0
APOA2	0	1.104665128	1	0.998	0	0
TF	0	1.095910941	0.954	0.381	0	0
ERRFI1	0	1.093332277	0.875	0.126	0	0
SERPINA1	0	1.088758998	0.981	0.7	0	0
EFNA1	0	1.085849836	0.907	0.139	0	0
KNG1	0	1.074624346	0.894	0.193	0	0
PTGR1	0	1.067031538	0.908	0.255	0	0
FGB	0	1.065470978	0.923	0.286	0	0
ATF5	0	1.063418074	0.897	0.315	0	0
SERPIND1	0	1.057093402	0.912	0.202	0	0
CYP3A5	0	1.049774885	0.822	0.056	0	0
A1BG	0	1.047256935	0.917	0.28	0	0
FN1	0	1.044040072	0.974	0.398	0	0
CYB5A	0	1.042757048	0.967	0.536	0	0
F2	0	1.041020159	0.939	0.258	0	0
ERBB3	0	1.037740915	0.887	0.087	0	0
CLU	0	1.02472319	0.928	0.26	0	0
TFPI	0	1.023798308	0.924	0.245	0	0
EPHX1	0	1.011176296	0.903	0.279	0	0
ITIH2	0	0.996225662	0.876	0.236	0	0
CES1	0	0.990893246	0.963	0.388	0	0
MPST	0	0.985072184	0.941	0.326	0	0
PEG10	0	0.959386521	0.798	0.222	0	0
DST	0	0.95881564	0.874	0.207	0	0
FST	0	0.956479933	0.873	0.146	0	0
PROC	0	0.955449574	0.929	0.182	0	0
SERPINC1	0	0.95424763	0.92	0.322	0	0
PLG	0	0.951549238	0.902	0.203	0	0
SLC38A3	0	0.950900925	0.872	0.107	0	0
MGST1	0	0.947913711	0.939	0.335	0	0
CFHR1	0	0.928953146	0.862	0.191	0	0
MST1	0	0.928627112	0.908	0.144	0	0
ORM2	0	0.926234973	0.887	0.241	0	0
CALD1	0	0.924546099	0.939	0.252	0	0
ANGPTL3	0	0.922788634	0.899	0.21	0	0
ALDH1L1	0	0.917701473	0.84	0.122	0	0
TMEM176B	0	0.917671303	0.96	0.519	0	0
HMGCS2	0	0.916674929	0.822	0.129	0	0
FGG	0	0.91126767	0.843	0.161	0	0
ZKSCAN1	0	0.907656324	0.875	0.212	0	0
PEBP1	0	0.906669805	0.972	0.673	0	0
GC	0	0.905578393	0.899	0.287	0	0
SPTBN1	0	0.898969238	0.901	0.188	0	0
LEAP2	0	0.890990025	0.796	0.131	0	0

GPAM	0	0.886547509	0.793	0.09	0	0
TMEM56	0	0.88246131	0.864	0.093	0	0
SERPINF2	0	0.869268425	0.871	0.121	0	0
G6PC	0	0.858521624	0.815	0.077	0	0
HSD17B2	0	0.858203467	0.863	0.123	0	0
SEPHS2	0	0.857753612	0.927	0.387	0	0
TM4SF4	0	0.856824708	0.907	0.198	0	0
ASGR2	0	0.856635435	0.89	0.168	0	0
MAT1A	0	0.851020045	0.869	0.108	0	0
FASN	0	0.844203874	0.818	0.147	0	0
TM4SF5	0	0.843389692	0.868	0.15	0	0
CYP27A1	0	0.838885443	0.884	0.193	0	0
DHCR24	0	0.837841614	0.858	0.139	0	0
C1S	0	0.835283167	0.888	0.184	0	0
DHCR7	0	0.831283483	0.838	0.134	0	0
POR	0	0.827892757	0.905	0.261	0	0
IRS2	0	0.827831368	0.897	0.259	0	0
GLYCTK	0	0.82355533	0.829	0.15	0	0
AADAC	0	0.813379257	0.874	0.183	0	0
KRT8	0	0.806367407	0.89	0.187	0	0
CDH2	0	0.79692714	0.866	0.106	0	0
MTTP	0	0.791239273	0.87	0.128	0	0
AKR1C2	0	0.787405892	0.89	0.235	0	0
ARG1	0	0.786145287	0.88	0.194	0	0
PLOD2	0	0.785224498	0.796	0.085	0	0
GPX3	0	0.784016449	0.857	0.215	0	0
KRT18	0	0.782023213	0.88	0.221	0	0
SLC2A2	0	0.779941181	0.776	0.111	0	0
HSD17B6	0	0.779436596	0.789	0.109	0	0
CYP2D6	0	0.77813151	0.786	0.086	0	0
SAA4	0	0.775526877	0.841	0.174	0	0
IDH1	0	0.773106436	0.889	0.335	0	0
VEGFA	0	0.77128978	0.828	0.175	0	0
SERPING1	0	0.767775829	0.897	0.301	0	0
MASP2	0	0.766460101	0.819	0.137	0	0
PLCB1	0	0.766232007	0.806	0.096	0	0
AKR1C1	0	0.763835629	0.879	0.239	0	0
A1CF	0	0.758053205	0.862	0.118	0	0
LBP	0	0.752124098	0.773	0.162	0	0
UGT2B4	0	0.747767877	0.821	0.135	0	0
SLC6A1	0	0.745456312	0.782	0.055	0	0
C8G	0	0.743816863	0.84	0.126	0	0
KANK1	0	0.736887228	0.812	0.064	0	0
H1F0	0	0.734378384	0.848	0.19	0	0
RRBP1	0	0.73417656	0.883	0.335	0	0

STARD10	0	0.724223412	0.882	0.212	0	0
SDC2	0	0.723537342	0.88	0.19	0	0
PRKAB2	0	0.718657205	0.818	0.103	0	0
METTL7A	0	0.71595051	0.87	0.266	0	0
IL6R	0	0.715526957	0.846	0.219	0	0
SPP2	0	0.710968631	0.766	0.132	0	0
PON3	0	0.7091073	0.794	0.132	0	0
HSD3B7	0	0.700111972	0.651	0.122	0	0
AKR1C3	0	0.699888683	0.906	0.298	0	0
CERS2	0	0.696638051	0.9	0.255	0	0
ACADSB	0	0.690952212	0.834	0.125	0	0
PAH	0	0.684592726	0.799	0.09	0	0
MEG8	0	0.681837144	0.624	0.053	0	0
AOX1	0	0.681159242	0.762	0.078	0	0
CHDH	0	0.677622374	0.765	0.049	0	0
C4BPB	0	0.677465265	0.791	0.118	0	0
KHK	0	0.671983193	0.766	0.115	0	0
QPRT	0	0.669730346	0.865	0.21	0	0
NUPR1	0	0.667920878	0.886	0.236	0	0
CLDN1	0	0.664714505	0.823	0.089	0	0
BHMT2	0	0.663396432	0.796	0.08	0	0
SDC1	0	0.66162841	0.828	0.108	0	0
CXADR	0	0.660316937	0.812	0.071	0	0
NORAD	0	0.656689975	0.875	0.275	0	0
AHCY	0	0.656502599	0.853	0.24	0	0
SLC39A14	0	0.647510206	0.808	0.097	0	0
MGAT4B	0	0.647216147	0.83	0.209	0	0
ELF3	0	0.646511275	0.588	0.037	0	0
SERINC2	0	0.643896641	0.666	0.083	0	0
GALK1	0	0.641190748	0.835	0.252	0	0
ALDH1A1	0	0.639979816	0.838	0.234	0	0
PROX1	0	0.639499793	0.756	0.064	0	0
HAO1	0	0.636889633	0.798	0.134	0	0
ALDH2	0	0.634003307	0.865	0.302	0	0
ACAA1	0	0.632966686	0.878	0.325	0	0
HAL	0	0.629214546	0.746	0.079	0	0
PPARA	0	0.620320034	0.761	0.107	0	0
SQLE	0	0.618424151	0.717	0.135	0	0
HNF4A	0	0.612961139	0.792	0.069	0	0
CPB2	0	0.612707682	0.804	0.11	0	0
INSIG2	0	0.611675113	0.739	0.146	0	0
TLE1	0	0.609863145	0.804	0.107	0	0
MET	0	0.608910625	0.784	0.07	0	0
GLUD1	0	0.607476905	0.871	0.31	0	0
MSMO1	0	0.607157394	0.759	0.174	0	0

ANG	0	0.606682012	0.764	0.124	0	0
CAMK2N1	0	0.605059698	0.828	0.166	0	0
LPIN2	0	0.60339037	0.806	0.212	0	0
MUT	0	0.602471591	0.796	0.137	0	0
SGCE	0	0.60099235	0.806	0.096	0	0
CFH	0	0.599207054	0.771	0.147	0	0
ASL	0	0.597054304	0.823	0.209	0	0
NAT8	0	0.594900596	0.749	0.09	0	0
AK4	0	0.594869284	0.829	0.124	0	0
TST	0	0.593027176	0.784	0.156	0	0
CFB	0	0.592070138	0.749	0.102	0	0
SCARB1	0	0.589581846	0.719	0.133	0	0
PCOLCE2	0	0.588136889	0.767	0.076	0	0
IRS1	0	0.582708529	0.75	0.055	0	0
SORL1	0	0.58003965	0.739	0.16	0	0
CES2	0	0.576927408	0.769	0.135	0	0
DUSP9	0	0.576717769	0.63	0.093	0	0
GGCX	0	0.574397954	0.827	0.178	0	0
APOC2	0	0.567243846	0.759	0.08	0	0
NR1I3	0	0.566582266	0.739	0.069	0	0
ABCC3	0	0.564168169	0.667	0.097	0	0
GCSH	0	0.56215663	0.847	0.259	0	0
PON2	0	0.562022137	0.836	0.236	0	0
SLC25A13	0	0.558334287	0.816	0.168	0	0
C5	0	0.557999484	0.741	0.101	0	0
ABCA6	0	0.550763872	0.665	0.046	0	0
PRODH2	0	0.54984492	0.736	0.08	0	0
SOWAHC	0	0.548570054	0.724	0.096	0	0
GAMT	0	0.547178362	0.824	0.223	0	0
PCOLCE	0	0.545773388	0.726	0.116	0	0
SC5D	0	0.545378559	0.766	0.158	0	0
FTCD	0	0.545217748	0.744	0.078	0	0
PPP1R3C	0	0.54153068	0.575	0.043	0	0
KLF15	0	0.537394853	0.732	0.052	0	0
TRIM24	0	0.537004867	0.768	0.125	0	0
TM7SF2	0	0.535332163	0.704	0.105	0	0
PGRMC1	0	0.529630641	0.877	0.257	0	0
VIL1	0	0.529183234	0.724	0.096	0	0
PPP1R16A	0	0.529005325	0.771	0.107	0	0
SMARCA1	0	0.527623092	0.717	0.074	0	0
LRP5	0	0.526861342	0.737	0.055	0	0
MOGAT3	0	0.525006386	0.672	0.054	0	0
CYP4F3	0	0.522873003	0.625	0.032	0	0
BNIP3	0	0.521350683	0.826	0.227	0	0
GCAT	0	0.520134325	0.77	0.113	0	0

MTMR4	0	0.520052356	0.722	0.103	0	0
CRYZ	0	0.519936501	0.791	0.152	0	0
PTPRF	0	0.517799965	0.749	0.057	0	0
UGDH	0	0.517191264	0.789	0.145	0	0
FXYD1	0	0.516413509	0.572	0.072	0	0
SLC9A3R2	0	0.516100786	0.857	0.197	0	0
TMEM120A	0	0.515462198	0.813	0.213	0	0
SDC4	0	0.512696175	0.748	0.139	0	0
C4BPA	0	0.510301038	0.629	0.074	0	0
CMTM8	0	0.509509518	0.731	0.074	0	0
WASL	0	0.506040389	0.788	0.14	0	0
PCYT2	0	0.499516662	0.794	0.156	0	0
F7	0	0.497689543	0.73	0.062	0	0
C2orf72	0	0.495857979	0.746	0.071	0	0
COL27A1	0	0.495043993	0.577	0.034	0	0
SERPINA6	0	0.494845978	0.7	0.076	0	0
RGN	0	0.494379737	0.72	0.078	0	0
ORMDL3	0	0.494147162	0.798	0.155	0	0
PROS1	0	0.492308958	0.766	0.12	0	0
NR0B2	0	0.490886912	0.625	0.053	0	0
NR2F6	0	0.490370931	0.784	0.117	0	0
BAG6	0	0.488629565	0.843	0.236	0	0
TMCC1	0	0.484838937	0.682	0.131	0	0
ALDH3A2	0	0.482039437	0.802	0.204	0	0
HPD	0	0.479907507	0.735	0.145	0	0
MCFD2	0	0.477875758	0.819	0.227	0	0
SLC1A2	0	0.477768312	0.647	0.044	0	0
PNPLA3	0	0.477411509	0.593	0.036	0	0
HIPK2	0	0.477379715	0.781	0.188	0	0
EVA1A	0	0.473639378	0.736	0.071	0	0
UGT1A1	0	0.473199499	0.667	0.059	0	0
GPT2	0	0.471482213	0.593	0.041	0	0
PALMD	0	0.468677698	0.683	0.088	0	0
GJB1	0	0.46788061	0.731	0.07	0	0
ANGPTL8	0	0.467736508	0.555	0.051	0	0
PDZK1	0	0.465225868	0.71	0.088	0	0
REEP6	0	0.464965452	0.734	0.084	0	0
PLXNB1	0	0.461701452	0.671	0.059	0	0
PC	0	0.461190406	0.692	0.064	0	0
RORC	0	0.460160212	0.657	0.053	0	0
ABAT	0	0.459403877	0.737	0.099	0	0
AQP9	0	0.458002697	0.744	0.122	0	0
ALDH4A1	0	0.45735275	0.734	0.096	0	0
SLC5A6	0	0.45641081	0.679	0.085	0	0
MIR122HG	0	0.454547357	0.607	0.037	0	0

CHST13	0	0.451800648	0.749	0.134	0	0
ADH1A	0	0.451027008	0.678	0.083	0	0
SMLR1	0	0.449600048	0.715	0.111	0	0
ACOX1	0	0.446191996	0.763	0.148	0	0
AMDHD1	0	0.444016799	0.714	0.089	0	0
CXCL12	0	0.443299257	0.722	0.111	0	0
CGREF1	0	0.44283625	0.716	0.087	0	0
REPIN1	0	0.440943996	0.783	0.174	0	0
PDLIM1	0	0.439280664	0.833	0.202	0	0
LRPPRC	0	0.437584015	0.807	0.2	0	0
SHMT2	0	0.43726098	0.785	0.201	0	0
H6PD	0	0.430969015	0.645	0.088	0	0
MBNL3	0	0.430575463	0.635	0.091	0	0
PHKA2	0	0.42890654	0.576	0.074	0	0
DDAH1	0	0.42888383	0.725	0.084	0	0
TTC38	0	0.428265357	0.761	0.144	0	0
CYP4A11	0	0.427363002	0.58	0.036	0	0
AGTR1	0	0.427256164	0.649	0.053	0	0
SFXN5	0	0.425477765	0.655	0.076	0	0
SESTD1	0	0.424945382	0.657	0.129	0	0
ALDH6A1	0	0.424164108	0.746	0.139	0	0
DHTKD1	0	0.424017159	0.692	0.117	0	0
PERP	0	0.422847577	0.694	0.096	0	0
PLIN5	0	0.422444159	0.564	0.031	0	0
FAM8A1	0	0.422209087	0.751	0.123	0	0
GOT2	0	0.420956997	0.741	0.169	0	0
PCYOX1	0	0.420334532	0.746	0.153	0	0
CCL16	0	0.42023604	0.63	0.057	0	0
RETREG2	0	0.418498792	0.788	0.179	0	0
FARP1	0	0.418479885	0.718	0.134	0	0
EPHX2	0	0.416663255	0.7	0.083	0	0
CRLS1	0	0.416619763	0.769	0.165	0	0
GUCD1	0	0.416460769	0.745	0.141	0	0
PAWR	0	0.414827111	0.666	0.064	0	0
TCAF1	0	0.414448323	0.723	0.135	0	0
PXMP2	0	0.413388155	0.72	0.144	0	0
SORBS2	0	0.413097184	0.632	0.046	0	0
ABCD3	0	0.412822039	0.724	0.119	0	0
HAAO	0	0.411217256	0.707	0.125	0	0
SORD	0	0.408951827	0.745	0.169	0	0
PKLR	0	0.407332511	0.594	0.057	0	0
HECTD1	0	0.406612623	0.751	0.182	0	0
SLC38A4	0	0.406065966	0.596	0.053	0	0
CCNB1IP1	0	0.406030019	0.76	0.178	0	0
TKFC	0	0.405957465	0.645	0.119	0	0

NT5E	0	0.405781315	0.65	0.074	0	0
NCKAP1	0	0.405770828	0.73	0.093	0	0
LIPC	0	0.404292235	0.657	0.07	0	0
NFIA	0	0.4024084	0.71	0.106	0	0
ASS1	0	0.399646441	0.682	0.086	0	0
EBPL	0	0.39959821	0.767	0.181	0	0
HPR	0	0.39915117	0.627	0.074	0	0
OAF	0	0.398386113	0.687	0.102	0	0
TGM2	0	0.397949383	0.673	0.101	0	0
LY6K	0	0.397910824	0.655	0.089	0	0
C11orf54	0	0.397905938	0.702	0.112	0	0
BHMT	0	0.397878993	0.605	0.054	0	0
SIGMAR1	0	0.397512643	0.748	0.138	0	0
CDO1	0	0.39726689	0.665	0.066	0	0
SELENBP1	0	0.397130655	0.662	0.072	0	0
F12	0	0.395957954	0.639	0.071	0	0
LMO7	0	0.395943512	0.667	0.063	0	0
HGD	0	0.395924478	0.661	0.072	0	0
NIPSNAP1	0	0.393960589	0.736	0.137	0	0
C1orf115	0	0.390438297	0.699	0.076	0	0
AFMID	0	0.388812689	0.751	0.137	0	0
SLCO1B1	0	0.388744623	0.59	0.044	0	0
PMM1	0	0.388226785	0.709	0.155	0	0
TCEA3	0	0.387038517	0.699	0.089	0	0
COBLL1	0	0.386914664	0.667	0.078	0	0
DDI2	0	0.385576868	0.726	0.148	0	0
NAPRT	0	0.384724402	0.729	0.159	0	0
ACO1	0	0.381571596	0.677	0.106	0	0
MAVS	0	0.379700506	0.692	0.136	0	0
FOXA2	0	0.378908382	0.639	0.05	0	0
CLDN15	0	0.376526823	0.634	0.071	0	0
GOT1	0	0.3763092	0.708	0.149	0	0
PAQR9	0	0.376248188	0.603	0.05	0	0
CAP2	0	0.37622472	0.63	0.055	0	0
SLC30A10	0	0.375846096	0.555	0.033	0	0
SERPINA5	0	0.374974974	0.59	0.057	0	0
LINC00470	0	0.374905222	0.629	0.053	0	0
ATRN	0	0.374837218	0.647	0.097	0	0
CREB3L3	0	0.374432317	0.61	0.049	0	0
FZD5	0	0.374347499	0.615	0.055	0	0
RMND5A	0	0.372447987	0.758	0.164	0	0
PCK2	0	0.372268805	0.675	0.13	0	0
BCAM	0	0.370617223	0.729	0.125	0	0
FLNB	0	0.370536512	0.668	0.083	0	0
SLC37A4	0	0.369161788	0.68	0.117	0	0

LAPTM4B	0	0.368537413	0.716	0.126	0	0
PGM1	0	0.367684619	0.732	0.143	0	0
PRAP1	0	0.3675088	0.631	0.088	0	0
ARHGEF40	0	0.367188332	0.641	0.08	0	0
PARD3B	0	0.3656276	0.6	0.048	0	0
HPN	0	0.364474286	0.543	0.033	0	0
SH3BP4	0	0.364345019	0.556	0.043	0	0
EGFR	0	0.363756379	0.543	0.042	0	0
HACD2	0	0.362929409	0.714	0.145	0	0
C6orf89	0	0.362273537	0.748	0.183	0	0
NRBP2	0	0.36191044	0.546	0.061	0	0
GCGR	0	0.360809231	0.535	0.033	0	0
TRIP6	0	0.359563942	0.743	0.173	0	0
CTDSPL	0	0.359525843	0.682	0.131	0	0
S100A16	0	0.359410028	0.776	0.158	0	0
VPS13A	0	0.357836596	0.613	0.101	0	0
GGH	0	0.35715344	0.724	0.139	0	0
LECT2	0	0.353990462	0.598	0.088	0	0
KIAA1147	0	0.352240589	0.657	0.113	0	0
MPDZ	0	0.351933942	0.628	0.07	0	0
ROBO1	0	0.35039741	0.598	0.068	0	0
C1R	0	0.349275667	0.588	0.076	0	0
TSKU	0	0.348814159	0.527	0.039	0	0
SEPT10	0	0.348147853	0.679	0.11	0	0
NR1H4	0	0.346672597	0.634	0.063	0	0
SLC27A5	0	0.346430126	0.64	0.079	0	0
SERPINA11	0	0.346051749	0.58	0.059	0	0
AGMO	0	0.345598695	0.619	0.055	0	0
CGN	0	0.344766789	0.572	0.032	0	0
BPHL	0	0.343904493	0.658	0.092	0	0
GRTP1	0	0.343855767	0.583	0.043	0	0
FBXO17	0	0.343736653	0.524	0.033	0	0
SLC16A1	0	0.343216822	0.75	0.168	0	0
NEDD4L	0	0.342556824	0.61	0.068	0	0
GOLT1A	0	0.340682962	0.623	0.058	0	0
C8A	0	0.33900893	0.593	0.051	0	0
F5	0	0.338914832	0.62	0.054	0	0
L3MBTL4	0	0.338647953	0.569	0.043	0	0
ALDH5A1	0	0.336020947	0.609	0.072	0	0
ARL4D	0	0.335976295	0.671	0.109	0	0
ATP2B2	0	0.335795557	0.493	0.025	0	0
OTUD7B	0	0.333201718	0.588	0.045	0	0
ZNF395	0	0.331890379	0.563	0.083	0	0
ABCC2	0	0.331839733	0.526	0.033	0	0
THRB	0	0.331647035	0.551	0.041	0	0

TSPAN6	0	0.331332346	0.669	0.08	0	0
RIDA	0	0.330928011	0.693	0.14	0	0
SERPINF1	0	0.330780987	0.675	0.123	0	0
AL355338.1	0	0.327909095	0.636	0.081	0	0
PPP1R3G	0	0.327241508	0.449	0.042	0	0
LGR4	0	0.326999732	0.59	0.046	0	0
TOMM70	0	0.326073261	0.702	0.148	0	0
BAAT	0	0.325401612	0.461	0.035	0	0
ARSE	0	0.325204969	0.652	0.066	0	0
ITIH4	0	0.325138033	0.556	0.047	0	0
SHROOM1	0	0.324529822	0.546	0.046	0	0
RHOBTB3	0	0.323628653	0.67	0.131	0	0
LURAP1L	0	0.322409107	0.559	0.047	0	0
ALDOB	0	0.322095829	0.432	0.024	0	0
RALGAPA2	0	0.321346416	0.604	0.077	0	0
ACOX2	0	0.321035337	0.62	0.084	0	0
DEPP1	0	0.319288458	0.613	0.1	0	0
LSS	0	0.31820371	0.524	0.069	0	0
EPAS1	0	0.317231625	0.723	0.146	0	0
DIO1	0	0.316621799	0.472	0.034	0	0
PECR	0	0.315316615	0.665	0.118	0	0
DSCR8	0	0.314572429	0.666	0.079	0	0
AKAP1	0	0.314285444	0.604	0.088	0	0
ACSM2B	0	0.312873817	0.483	0.023	0	0
MIB1	0	0.312358845	0.679	0.136	0	0
COBL	0	0.312212466	0.551	0.036	0	0
NADK2	0	0.312067218	0.614	0.078	0	0
MYO1B	0	0.308960351	0.665	0.083	0	0
DCDC2	0	0.308817916	0.485	0.048	0	0
TMEM150A	0	0.308395066	0.645	0.098	0	0
SSUH2	0	0.30785004	0.608	0.062	0	0
VWCE	0	0.306478981	0.457	0.032	0	0
CFHR5	0	0.306221228	0.522	0.046	0	0
PCCA	0	0.306010272	0.623	0.091	0	0
LSR	0	0.305220145	0.627	0.085	0	0
IGF2BP2	0	0.30477578	0.597	0.063	0	0
C6orf106	0	0.303915936	0.671	0.135	0	0
ENOSF1	0	0.303644336	0.674	0.134	0	0
NDRG2	0	0.301451448	0.665	0.103	0	0
ENTPD5	0	0.300941333	0.599	0.084	0	0
KLC4	0	0.300890988	0.559	0.056	0	0
SNTB1	0	0.299866271	0.628	0.096	0	0
CENPV	0	0.299119032	0.62	0.08	0	0
CDKN2A	0	0.298021712	0.709	0.132	0	0
VKORC1L1	0	0.297384179	0.672	0.126	0	0

SEMA4G	0	0.294205366	0.523	0.039	0	0
HYAL1	0	0.294038001	0.578	0.055	0	0
YES1	0	0.293886565	0.678	0.087	0	0
CTSF	0	0.292777834	0.638	0.116	0	0
GPRC5C	0	0.292670513	0.613	0.075	0	0
CECR2	0	0.292269566	0.501	0.025	0	0
ECHDC2	0	0.291569294	0.655	0.12	0	0
HLF	0	0.291115089	0.572	0.056	0	0
ZCCHC14	0	0.29104647	0.523	0.051	0	0
GLDC	0	0.29086989	0.529	0.045	0	0
ENAH	0	0.290700241	0.595	0.065	0	0
CROT	0	0.290407694	0.581	0.086	0	0
ABCG5	0	0.288658699	0.504	0.03	0	0
ARHGAP35	0	0.288636127	0.615	0.083	0	0
NFIB	0	0.288276404	0.615	0.072	0	0
PRKAA2	0	0.287846413	0.484	0.032	0	0
ABCG8	0	0.287809259	0.503	0.028	0	0
AGFG2	0	0.286110414	0.58	0.07	0	0
ENPP1	0	0.28543974	0.514	0.04	0	0
TIMP3	0	0.283856669	0.759	0.14	0	0
SEMA6C	0	0.282783909	0.476	0.034	0	0
CTTN	0	0.282352405	0.636	0.083	0	0
ACVR2B	0	0.281318701	0.515	0.05	0	0
IL17RC	0	0.279366281	0.578	0.071	0	0
PEMT	0	0.279349249	0.632	0.106	0	0
NAV2	0	0.277204182	0.525	0.044	0	0
TBX3	0	0.276601368	0.461	0.031	0	0
VSNL1	0	0.276526741	0.612	0.077	0	0
KIF13A	0	0.275533912	0.622	0.096	0	0
MAGEA3	0	0.275422241	0.586	0.055	0	0
SPR	0	0.275273755	0.586	0.078	0	0
ALDH7A1	0	0.274966066	0.632	0.113	0	0
IGFBP4	0	0.273933254	0.725	0.148	0	0
ELOVL2	0	0.27176381	0.536	0.057	0	0
NR5A2	0	0.270545222	0.491	0.036	0	0
PNMA6A	0	0.270280419	0.472	0.032	0	0
UNC93A	0	0.26937226	0.433	0.029	0	0
ABCC6	0	0.267620265	0.538	0.041	0	0
AC009403.1	0	0.266489199	0.563	0.078	0	0
SMOC1	0	0.266233235	0.488	0.032	0	0
NID1	0	0.265148577	0.684	0.117	0	0
AC007388.1	0	0.26500043	0.558	0.067	0	0
AASS	0	0.264732128	0.414	0.023	0	0
FGFR3	0	0.264446065	0.486	0.036	0	0
NECAB3	0	0.264162057	0.585	0.08	0	0

CNN3	0	0.261582035	0.731	0.14	0	0
EPB41L5	0	0.26083952	0.529	0.041	0	0
SLC26A6	0	0.260561292	0.495	0.058	0	0
ZIC2	0	0.259950486	0.503	0.035	0	0
HS3ST3B1	0	0.25980758	0.549	0.059	0	0
CFI	0	0.259038356	0.576	0.073	0	0
UGT2B7	0	0.25871529	0.544	0.059	0	0
RTKN	0	0.258119202	0.543	0.043	0	0
GCKR	0	0.257896256	0.467	0.033	0	0
NAPEPLD	0	0.25571514	0.507	0.054	0	0
SNORC	0	0.25569342	0.476	0.044	0	0
COL18A1	0	0.255131499	0.778	0.14	0	0
MAGI1	0	0.253167104	0.541	0.046	0	0
SEC16B	0	0.252638026	0.459	0.037	0	0
PIPOX	0	0.25060816	0.558	0.065	0	0
TMEM14A	1.41E-307	0.300010423	0.695	0.136	2.91E-303	0
SLC39A7	4.76E-307	0.341384268	0.707	0.158	9.81E-303	0
FIGN	2.83E-306	0.259043986	0.434	0.037	5.84E-302	0
ADH4	3.61E-306	0.344482856	0.559	0.081	7.44E-302	0
PGPEP1	1.17E-305	0.298653296	0.657	0.128	2.41E-301	0
CYP4F11	2.82E-305	0.262265206	0.413	0.031	5.80E-301	0
CDC42EP1	6.99E-304	0.296025556	0.635	0.12	1.44E-299	0
FAM213A	1.07E-303	0.468759913	0.831	0.247	2.21E-299	0
ZCCHC24	2.07E-301	0.311880737	0.501	0.064	4.27E-297	0
CHD1L	3.26E-300	0.353987273	0.684	0.148	6.71E-296	0
ALCAM	6.14E-300	0.378146862	0.725	0.167	1.27E-295	0
GTF2I	1.30E-298	0.617660014	0.916	0.38	2.67E-294	0
TRAP1	2.87E-297	0.31490504	0.682	0.149	5.91E-293	0
ESF1	9.77E-297	0.461024433	0.832	0.247	2.01E-292	0
APOE	8.17E-296	0.817749825	0.999	0.898	1.68E-291	0
AKR1C4	1.73E-295	0.541959976	0.754	0.182	3.57E-291	0
HSBP1L1	2.97E-295	0.272560416	0.614	0.113	6.12E-291	0
TMEM97	1.18E-294	0.367159234	0.624	0.116	2.43E-290	0
SREBF2	2.59E-292	0.269734746	0.595	0.104	5.33E-288	0
CHPT1	5.54E-292	0.400143028	0.76	0.211	1.14E-287	0
CKAP4	2.17E-291	0.26329538	0.62	0.116	4.48E-287	0
VARS	1.31E-290	0.284880467	0.652	0.131	2.70E-286	0
FAH	1.92E-290	0.309447147	0.633	0.127	3.95E-286	0
STAU1	9.55E-290	0.48727465	0.845	0.285	1.97E-285	0
COL5A2	1.35E-289	0.27657967	0.511	0.069	2.79E-285	0
PPP1R35	4.93E-289	0.372681554	0.778	0.203	1.01E-284	0
S100A13	3.30E-288	0.280246067	0.677	0.14	6.79E-284	0
AK3	5.17E-288	0.349342205	0.764	0.204	1.06E-283	0
PPP2R5A	8.30E-288	0.415465495	0.786	0.215	1.71E-283	0
SLC44A1	9.75E-288	0.298817621	0.661	0.14	2.01E-283	0

LRG1	2.51E-287	0.299351449	0.513	0.073	5.17E-283	0
NDUFS1	4.59E-287	0.355565951	0.771	0.212	9.45E-283	0
RPRD2	2.74E-286	0.289847489	0.66	0.137	5.65E-282	0
GATM	3.56E-286	0.694126838	0.88	0.326	7.32E-282	0
DCXR	6.47E-286	0.741673662	0.91	0.426	1.33E-281	0
GMCL1	5.21E-285	0.253616601	0.677	0.143	1.07E-280	0
PCSK9	8.92E-285	0.274156993	0.427	0.042	1.84E-280	0
ACSL4	1.99E-284	0.585210834	0.782	0.246	4.10E-280	0
ST6GAL1	1.06E-281	0.439628613	0.736	0.195	2.18E-277	0
SLC25A1	2.33E-278	0.374888714	0.766	0.213	4.79E-274	0
MAGED1	1.68E-277	0.267573066	0.588	0.109	3.47E-273	0
PPP4R2	1.76E-277	0.40204859	0.78	0.225	3.62E-273	0
FABP1	2.49E-276	1.007401206	0.958	0.664	5.13E-272	0
UPK3A	8.77E-275	0.291194796	0.551	0.09	1.81E-270	0
HMGCR	4.25E-274	0.358402378	0.571	0.105	8.76E-270	0
SLC22A18	4.40E-274	0.337241997	0.717	0.188	9.07E-270	0
SELENOO	9.50E-274	0.251923287	0.593	0.113	1.96E-269	0
PNPLA2	3.20E-273	0.276011969	0.692	0.162	6.60E-269	0
MSRB1	1.03E-272	0.283215826	0.715	0.176	2.12E-268	0
HIBCH	1.38E-272	0.267699065	0.669	0.148	2.85E-268	0
NFE2L1	2.87E-272	0.30751047	0.699	0.173	5.90E-268	0
ADH1B	2.89E-272	0.665567391	0.422	0.046	5.94E-268	0
UBN2	4.59E-271	0.253373318	0.555	0.096	9.44E-267	0
PQLC1	6.05E-271	0.409143164	0.743	0.21	1.25E-266	0
ECI2	1.18E-270	0.422871801	0.807	0.259	2.43E-266	0
MDK	3.02E-269	0.442701809	0.709	0.177	6.22E-265	0
ACSL3	5.05E-269	0.387509564	0.748	0.204	1.04E-264	0
ARPC1A	1.60E-268	0.431274688	0.853	0.289	3.30E-264	0
POLD2	2.51E-268	0.269414916	0.692	0.16	5.17E-264	0
POM121	3.94E-268	0.289958112	0.662	0.149	8.11E-264	0
TMEM245	2.33E-267	0.259945077	0.673	0.152	4.81E-263	0
EBP	4.97E-267	0.618419143	0.839	0.287	1.02E-262	0
CYP3A7	6.07E-267	0.350755239	0.387	0.035	1.25E-262	0
POLR2J3.1	1.55E-266	0.577581517	0.82	0.264	3.20E-262	0
ARHGAP5	1.51E-265	0.280404692	0.719	0.181	3.11E-261	0
ZBTB20	1.64E-265	0.624961009	0.835	0.271	3.37E-261	0
MRPS18B	2.35E-264	0.445094208	0.829	0.287	4.85E-260	0
HIST1H2AC	2.64E-264	0.371352812	0.804	0.233	5.44E-260	0
ARFGEF2	3.72E-264	0.252576917	0.549	0.099	7.66E-260	0
MRPS26	3.36E-263	0.408868032	0.811	0.255	6.92E-259	0
SEC23A	5.51E-263	0.267185976	0.694	0.172	1.13E-258	0
HP	1.40E-262	1.281878018	0.931	0.489	2.89E-258	0
IQSEC1	2.91E-261	0.318008665	0.698	0.175	5.98E-257	0
LMAN1	3.13E-261	0.571269409	0.866	0.344	6.45E-257	0
MUC20-OT1	1.32E-260	0.373158754	0.73	0.188	2.72E-256	0

CIDEB	1.32E-259	0.253962044	0.565	0.107	2.71E-255	0
BRAF	2.71E-259	0.256655936	0.587	0.117	5.58E-255	0
PCBD1	2.84E-259	0.617481682	0.885	0.403	5.84E-255	0
EML4	6.83E-259	0.526473703	0.857	0.308	1.41E-254	0
HNMT	4.32E-258	0.558150576	0.887	0.367	8.90E-254	0
CAPN12	1.12E-257	0.296226049	0.387	0.037	2.31E-253	0
ARHGEF10L	2.05E-257	0.255252121	0.481	0.073	4.22E-253	0
LPGAT1	2.58E-257	0.372106893	0.728	0.202	5.30E-253	0
ORAI3	1.67E-256	0.33889957	0.653	0.162	3.44E-252	0
TNFRSF12A	4.94E-256	0.3353989	0.493	0.077	1.02E-251	0
APCS	7.77E-256	1.02888677	0.627	0.162	1.60E-251	0
RHOU	2.41E-255	0.260010106	0.546	0.102	4.95E-251	0
DCPS	1.77E-254	0.436569718	0.731	0.209	3.64E-250	0
FMC1	4.92E-253	0.262077852	0.692	0.175	1.01E-248	0
CD2AP	2.70E-252	0.272051982	0.653	0.155	5.56E-248	0
SHMT1	1.21E-250	0.266978301	0.647	0.154	2.50E-246	0
HIST1H1C	3.44E-250	0.560385391	0.814	0.272	7.08E-246	0
QARS	1.35E-248	0.318593443	0.786	0.245	2.78E-244	0
RAB13	2.38E-247	0.436163922	0.853	0.324	4.90E-243	0
TRIB3	1.60E-246	0.34644821	0.451	0.065	3.30E-242	0
FNDC3B	3.00E-246	0.461088548	0.802	0.258	6.18E-242	0
ST3GAL1	4.82E-246	0.364584926	0.759	0.223	9.93E-242	0
FDPS	1.16E-245	0.830152859	0.908	0.445	2.38E-241	0
GCLC	5.84E-243	0.299979244	0.682	0.174	1.20E-238	0
ELOVL5	7.70E-242	0.349942152	0.788	0.241	1.59E-237	0
PDIA4	8.57E-242	0.585225212	0.871	0.376	1.77E-237	0
HDLBP	1.09E-241	0.454010441	0.858	0.338	2.24E-237	0
EIF4EBP2	1.19E-241	0.336527783	0.778	0.239	2.45E-237	0
UBE2H	3.29E-240	0.322109284	0.768	0.234	6.78E-236	0
CHD6	3.60E-240	0.282498848	0.685	0.175	7.42E-236	0
ACADM	4.10E-240	0.307038905	0.74	0.22	8.45E-236	0
SLC50A1	1.66E-239	0.297587879	0.714	0.199	3.42E-235	0
AIG1	2.52E-239	0.327062972	0.744	0.229	5.19E-235	0
WEE1	5.18E-239	0.282411264	0.536	0.101	1.07E-234	0
YIPF4	6.11E-239	0.359106994	0.786	0.252	1.26E-234	0
DHRS3	6.74E-239	0.294398416	0.747	0.212	1.39E-234	0
GID8	9.96E-239	0.26383367	0.706	0.188	2.05E-234	0
EI24	3.57E-238	0.336835434	0.787	0.246	7.36E-234	0
RB1CC1	3.38E-237	0.296155517	0.746	0.22	6.96E-233	0
IP6K2	1.42E-236	0.252501001	0.699	0.185	2.92E-232	0
LONP2	1.05E-235	0.295164004	0.694	0.187	2.16E-231	0
MTSS1	2.48E-235	0.306458828	0.756	0.215	5.11E-231	0
C1orf43	1.14E-234	0.482730682	0.895	0.41	2.35E-230	0
TOR1AIP2	2.50E-234	0.281705887	0.699	0.187	5.15E-230	0
RAF1	4.02E-234	0.299639472	0.722	0.204	8.28E-230	0

TNS1	5.77E-234	0.328612856	0.513	0.099	1.19E-229	0
RXRA	7.51E-234	0.320081807	0.686	0.186	1.55E-229	0
ATP13A3	1.31E-233	0.281896425	0.744	0.212	2.69E-229	0
FBP1	1.73E-233	0.388319185	0.726	0.222	3.56E-229	0
MKLN1	3.21E-233	0.292281827	0.703	0.192	6.62E-229	0
ECI1	1.26E-232	0.337222926	0.788	0.263	2.60E-228	0
PRKAR2A	2.14E-232	0.305366968	0.769	0.236	4.40E-228	0
AAMP	2.29E-232	0.256207295	0.733	0.213	4.71E-228	0
YIPF3	5.80E-232	0.347547766	0.827	0.301	1.19E-227	0
CD302	1.06E-231	0.38205432	0.811	0.285	2.19E-227	0
FH	9.09E-231	0.25709991	0.67	0.18	1.87E-226	0
NUDT3	2.45E-230	0.28099091	0.699	0.196	5.06E-226	0
MCRIP2	2.48E-230	0.279483955	0.692	0.189	5.11E-226	0
ERGIC2	3.24E-230	0.360257969	0.808	0.276	6.68E-226	0
CRYL1	9.38E-229	0.288787284	0.694	0.196	1.93E-224	0
ACSS2	3.57E-228	0.346888341	0.499	0.098	7.35E-224	0
MAPK6	5.59E-228	0.256151503	0.632	0.157	1.15E-223	0
HHEX	1.73E-227	0.285940619	0.657	0.174	3.56E-223	0
PTP4A1	3.11E-227	0.607335182	0.91	0.425	6.40E-223	0
HMGCS1	2.26E-226	0.586786239	0.667	0.196	4.66E-222	0
YIF1A	7.65E-226	0.390065101	0.798	0.291	1.57E-221	0
PSMB5	1.18E-225	0.41327558	0.808	0.309	2.43E-221	0
ISOC2	5.16E-225	0.29190288	0.74	0.226	1.06E-220	0
PYGL	4.58E-224	0.251258341	0.657	0.171	9.43E-220	0
RPS7	8.60E-224	0.525691809	0.996	0.98	1.77E-219	0
SLC9A3R1	1.07E-223	0.421082303	0.823	0.291	2.20E-219	0
SLC2A4RG	3.84E-223	0.304753641	0.71	0.199	7.91E-219	0
CD46	4.04E-223	0.360552259	0.816	0.281	8.32E-219	0
SEC63	8.22E-222	0.340002808	0.785	0.267	1.69E-217	0
PLEKHA1	9.04E-222	0.301747917	0.702	0.196	1.86E-217	0
CYP2C8	3.52E-221	0.38808101	0.296	0.02	7.24E-217	0
HSD17B11	1.69E-219	0.403436375	0.84	0.336	3.49E-215	0
SLC39A1	4.17E-219	0.332490865	0.783	0.255	8.59E-215	0
ACAT2	4.19E-219	0.395001292	0.692	0.205	8.64E-215	0
SPATS2L	3.57E-218	0.369412163	0.846	0.314	7.36E-214	0
MPC2	3.86E-218	0.533585047	0.906	0.479	7.95E-214	0
TFG	6.72E-218	0.333193474	0.814	0.3	1.38E-213	0
RPS6KA3	3.01E-217	0.319858754	0.778	0.256	6.20E-213	0
RNF5	7.11E-216	0.317359181	0.797	0.271	1.46E-211	0
CCL20	2.52E-215	1.066154927	0.766	0.286	5.19E-211	0
SMARCC1	3.45E-214	0.264013576	0.736	0.23	7.11E-210	0
PHF3	1.05E-213	0.357150355	0.78	0.262	2.16E-209	0
ASH1L	5.97E-213	0.356700142	0.787	0.264	1.23E-208	0
DDT	1.28E-212	0.654204625	0.908	0.572	2.65E-208	0
ACAT1	6.69E-212	0.256878005	0.709	0.22	1.38E-207	0

ERG28	1.06E-210	0.263624858	0.642	0.172	2.18E-206	0
SERINC3	2.09E-210	0.366244283	0.831	0.311	4.29E-206	0
PHYH	3.87E-210	0.26874289	0.731	0.235	7.98E-206	0
ERGIC3	5.37E-210	0.407895379	0.868	0.385	1.10E-205	0
GSTA1	5.41E-210	0.782545417	0.788	0.313	1.11E-205	0
DPM3	7.63E-209	0.319620213	0.759	0.263	1.57E-204	0
SAT2	1.14E-205	0.466394762	0.823	0.333	2.35E-201	0
NAA20	2.72E-204	0.393182418	0.846	0.339	5.61E-200	0
TXNRD1	2.95E-204	0.333734873	0.672	0.206	6.08E-200	0
ADI1	9.28E-204	0.449319329	0.86	0.392	1.91E-199	0
SND1	9.70E-204	0.303289076	0.787	0.284	2.00E-199	0
ZBTB38	1.43E-203	0.267416286	0.731	0.228	2.95E-199	0
TMEM141	1.52E-203	0.371142743	0.741	0.259	3.13E-199	0
GOLGA4	2.08E-203	0.489428149	0.88	0.401	4.28E-199	0
ZNF638	2.63E-201	0.304989715	0.709	0.219	5.43E-197	0
DNAJC19	1.79E-200	0.277333316	0.772	0.275	3.68E-196	0
ADH5	2.27E-198	0.330249078	0.821	0.323	4.68E-194	0
SCCPDH	4.12E-198	0.253266183	0.68	0.211	8.49E-194	0
PDHB	1.19E-196	0.293673839	0.769	0.28	2.44E-192	0
HAGH	2.68E-196	0.256473023	0.731	0.24	5.52E-192	0
SUCLG2	2.98E-196	0.258566552	0.758	0.258	6.13E-192	0
ECHS1	1.23E-195	0.465589705	0.877	0.439	2.53E-191	0
BACH1	2.76E-192	0.266393208	0.668	0.204	5.68E-188	0
TOB1	2.46E-191	0.288150306	0.805	0.283	5.07E-187	0
CNIH1	3.66E-191	0.258308248	0.796	0.288	7.54E-187	0
NDUFC2	6.74E-191	0.513340223	0.922	0.573	1.39E-186	0
SERPINE1	4.05E-190	0.828900195	0.343	0.047	8.34E-186	0
RPL7L1	4.90E-190	0.290206333	0.806	0.296	1.01E-185	0
ETFB	1.14E-189	0.466994919	0.855	0.428	2.34E-185	0
UQCRC1	1.67E-188	0.389756493	0.838	0.364	3.44E-184	0
ERGIC1	6.18E-188	0.269027681	0.677	0.213	1.27E-183	0
OXA1L	2.27E-187	0.353936046	0.812	0.319	4.68E-183	0
NDUFS2	4.53E-186	0.326043812	0.821	0.335	9.34E-182	0
FXR1	1.21E-184	0.259045021	0.788	0.289	2.49E-180	0
RNMT	3.25E-183	0.291577298	0.768	0.268	6.69E-179	0
KLHL24	1.50E-182	0.252367564	0.67	0.207	3.08E-178	0
DBI	2.48E-182	0.523095547	0.951	0.72	5.10E-178	0
GLO1	7.55E-182	0.267386467	0.82	0.329	1.56E-177	0
P4HA1	2.26E-181	0.265132776	0.659	0.21	4.66E-177	0
EIF4G1	1.47E-179	0.295623777	0.732	0.254	3.02E-175	0
P4HB	3.59E-179	0.444016316	0.937	0.543	7.40E-175	0
FTX	3.42E-178	0.251794863	0.524	0.131	7.04E-174	0
ETFA	7.48E-178	0.258942461	0.795	0.309	1.54E-173	0
ECH1	1.65E-177	0.446640666	0.882	0.457	3.41E-173	0
EIF6	5.09E-177	0.386088248	0.853	0.389	1.05E-172	0

MPLKIP	8.83E-177	0.250836265	0.774	0.287	1.82E-172	0
LAPTM4A	1.00E-175	0.442518442	0.899	0.474	2.07E-171	0
FLOT1	1.26E-175	0.305410696	0.808	0.323	2.60E-171	0
CANX	1.92E-175	0.487235274	0.917	0.488	3.95E-171	0
IRF2BP2	1.72E-174	0.400475228	0.875	0.38	3.54E-170	0
AFP	5.34E-174	0.30041231	0.33	0.047	1.10E-169	0
APMAP	3.69E-173	0.317461495	0.838	0.345	7.60E-169	0
CHCHD10	4.42E-173	0.496097521	0.92	0.581	9.11E-169	0
AKR1B10	1.74E-172	0.507751164	0.529	0.143	3.59E-168	0
ZNHIT1	1.94E-171	0.416625059	0.88	0.482	4.00E-167	0
CUTA	2.16E-170	0.475275823	0.902	0.564	4.46E-166	0
APOC1	6.76E-170	0.529709727	0.979	0.728	1.39E-165	0
HDGF	1.39E-169	0.263617611	0.829	0.331	2.86E-165	0
NDUFA10	3.48E-169	0.285058289	0.805	0.33	7.18E-165	0
SURF4	4.27E-169	0.288720148	0.814	0.324	8.80E-165	0
MT-ND5	8.02E-169	0.606590963	0.992	0.963	1.65E-164	0
PRDX6	2.22E-167	0.405175103	0.879	0.478	4.58E-163	0
PNKD	1.16E-166	0.250955433	0.786	0.301	2.38E-162	0
FDFT1	1.40E-165	0.274453816	0.694	0.243	2.88E-161	0
CPNE1	1.85E-163	0.25632275	0.771	0.295	3.81E-159	0
COX7A2L	2.31E-162	0.376631327	0.885	0.475	4.75E-158	0
RPL14	2.57E-161	0.392843875	0.995	0.956	5.29E-157	0
RPL15	2.91E-161	0.437256101	0.991	0.966	6.00E-157	0
AKAP9	2.89E-160	0.364407327	0.851	0.381	5.96E-156	0
AUP1	1.00E-159	0.312904626	0.843	0.37	2.06E-155	0
MRPL14	1.53E-159	0.289542256	0.798	0.344	3.16E-155	0
KRTCAP2	8.27E-159	0.312897355	0.835	0.389	1.70E-154	0
PSMD4	8.36E-159	0.306469067	0.876	0.421	1.72E-154	0
HILPDA	3.08E-158	0.299488407	0.393	0.082	6.35E-154	0
SUCLG1	2.00E-157	0.34675791	0.792	0.344	4.11E-153	0
APLP2	3.52E-157	0.428050819	0.914	0.468	7.25E-153	0
TKT	1.34E-156	0.410996773	0.885	0.476	2.77E-152	0
TMEM123	2.25E-156	0.451418184	0.882	0.442	4.62E-152	0
HADHB	2.93E-156	0.26141388	0.814	0.342	6.04E-152	0
RPL37A	2.08E-154	0.415288938	0.989	0.969	4.29E-150	0
COMT	1.23E-153	0.282216757	0.798	0.342	2.53E-149	0
KTN1	2.55E-153	0.329690534	0.868	0.407	5.25E-149	0
FIS1	6.27E-153	0.371192686	0.887	0.516	1.29E-148	0
HADHA	6.00E-152	0.306596655	0.84	0.386	1.23E-147	0
RPL5	7.05E-152	0.423911397	0.989	0.941	1.45E-147	0
XBP1	1.17E-151	0.308945921	0.88	0.419	2.40E-147	0
LDHA	2.68E-150	0.626295638	0.95	0.7	5.53E-146	0
RHEB	1.45E-148	0.312968236	0.86	0.404	2.99E-144	0
NCOA4	5.07E-148	0.299093689	0.844	0.359	1.04E-143	0
APH1A	5.52E-148	0.262222183	0.826	0.345	1.14E-143	0

PRDX3	1.57E-147	0.311249058	0.86	0.396	3.22E-143	0
TMEM14B	1.26E-146	0.298774085	0.815	0.374	2.59E-142	0
TMCO1	2.24E-146	0.285917721	0.845	0.39	4.61E-142	0
SSR1	1.15E-145	0.320497236	0.845	0.384	2.38E-141	0
EIF4B	1.96E-141	0.352677582	0.902	0.484	4.04E-137	0
RPS18	1.48E-140	0.386439089	0.999	0.995	3.05E-136	0
UGP2	1.74E-135	0.303159324	0.844	0.397	3.58E-131	0
PDIA6	9.72E-134	0.40874114	0.904	0.562	2.00E-129	0
POLR2J	2.53E-132	0.25533707	0.832	0.403	5.21E-128	0
RPLP0	6.46E-129	0.366603379	0.978	0.865	1.33E-124	0
TMEM14C	1.05E-128	0.289284813	0.858	0.445	2.16E-124	0
MTCH1	1.03E-127	0.260080714	0.847	0.396	2.13E-123	0
NDRG1	6.95E-126	0.344803739	0.501	0.159	1.43E-121	0
TUFM	2.10E-125	0.284537237	0.858	0.424	4.32E-121	0
VKORC1	7.56E-125	0.297841948	0.786	0.354	1.56E-120	0
SEC11C	4.72E-124	0.257589195	0.806	0.376	9.72E-120	0
HSP90B1	2.98E-123	0.414793757	0.93	0.605	6.14E-119	0
MT-ND4L	2.70E-120	0.444141166	0.945	0.704	5.56E-116	0
BRI3	7.32E-119	0.402784517	0.944	0.607	1.51E-114	0
CCT3	8.24E-119	0.255893604	0.862	0.444	1.70E-114	0
IQGAP2	1.98E-116	0.252270557	0.807	0.364	4.08E-112	0
FAM13A	2.03E-114	0.29339079	0.413	0.126	4.17E-110	0
CTSA	5.14E-113	0.275913774	0.866	0.409	1.06E-108	0
PSMB4	5.19E-113	0.262816384	0.865	0.488	1.07E-108	0
MRPS21	9.88E-111	0.25189653	0.876	0.501	2.03E-106	0
RPL29	8.38E-107	0.310627468	0.988	0.962	1.73E-102	0
RPSA	6.15E-106	0.341568955	0.977	0.906	1.27E-101	0
EIF3L	3.96E-102	0.261652191	0.886	0.527	8.16E-98	0
RPL24	1.14E-101	0.325978145	0.984	0.946	2.34E-97	0
CEBPB	1.51E-100	0.289270358	0.903	0.454	3.10E-96	0
IFITM3	2.90E-100	0.327388117	0.915	0.569	5.98E-96	0
ST13	1.87E-99	0.267836315	0.873	0.517	3.86E-95	0
RPN2	2.23E-99	0.273773625	0.86	0.47	4.58E-95	0
MZT2B	5.52E-98	0.257068811	0.914	0.627	1.14E-93	0
MT-ND2	5.96E-98	0.388812217	1	0.997	1.23E-93	0
TGOLN2	6.69E-98	0.274513058	0.882	0.462	1.38E-93	0
TOMM20	4.07E-97	0.2615963	0.879	0.529	8.37E-93	0
EEF2	6.85E-92	0.327229465	0.939	0.692	1.41E-87	0
MT-CYB	2.58E-91	0.371685268	1	1	5.32E-87	0
MT-CO2	5.03E-90	0.353912752	1	1	1.04E-85	0
MT-ND1	1.93E-88	0.327206085	0.999	0.999	3.96E-84	0
GSTO1	9.38E-85	0.27738358	0.9	0.615	1.93E-80	0
ATP5MF	3.09E-84	0.282873521	0.913	0.661	6.35E-80	0
MT-ND4	1.45E-77	0.305478729	1	1	2.99E-73	0
RPS10	5.34E-63	0.295181293	0.97	0.909	1.10E-58	0

HSPA5	3.08E-62	0.256476495	0.905	0.583	6.35E-58	0
NEAT1	1.62E-60	0.583586309	0.927	0.737	3.33E-56	0
HINT1	1.72E-57	0.25582188	0.942	0.8	3.55E-53	0
MT-CO1	1.91E-39	0.277502832	1	0.999	3.94E-35	0
RPS18	2.34E-202	1.034191347	1	0.995	4.82E-198	1
APOA2	1.73E-182	1.051136761	1	0.998	3.57E-178	1
RPL32	1.03E-174	0.997363756	0.989	0.99	2.13E-170	1
RPS7	1.75E-172	1.002034348	0.982	0.984	3.61E-168	1
RPL37A	2.53E-161	0.911414498	0.974	0.974	5.20E-157	1
RPL29	7.62E-160	0.943886414	0.97	0.968	1.57E-155	1
ALB	1.36E-149	0.954932576	1	0.993	2.80E-145	1
RPL14	5.31E-145	0.891031809	0.961	0.966	1.09E-140	1
EEF1A1	1.62E-134	0.723837348	1	0.997	3.33E-130	1
RPL5	7.46E-131	0.903318663	0.946	0.954	1.54E-126	1
RPS6	3.51E-125	0.851485478	0.974	0.974	7.23E-121	1
RPL24	9.71E-125	0.832327217	0.944	0.957	2.00E-120	1
RPL39	4.29E-116	0.799581812	0.974	0.986	8.83E-112	1
SERPINA1	1.79E-115	1.022225026	0.899	0.751	3.68E-111	1
RPS10	4.92E-112	0.829588757	0.905	0.926	1.01E-107	1
RPL15	3.31E-110	0.776290062	0.958	0.974	6.81E-106	1
FABP1	1.93E-108	1.104008296	0.861	0.72	3.98E-104	1
RPS27	3.02E-108	0.70929486	0.991	0.989	6.22E-104	1
AHSG	7.84E-106	0.848604725	0.938	0.787	1.61E-101	1
RPL37	1.41E-104	0.762475972	0.953	0.974	2.91E-100	1
APOC3	1.58E-103	0.84981241	0.908	0.737	3.26E-99	1
RPS27A	5.20E-102	0.631050044	0.985	0.983	1.07E-97	1
RPL35	1.29E-100	0.696545228	0.903	0.939	2.66E-96	1
RPL11	1.15E-97	0.6269839	0.961	0.976	2.38E-93	1
RPL35A	6.88E-97	0.747619337	0.923	0.96	1.42E-92	1
FGL1	1.95E-95	0.914726413	0.914	0.746	4.01E-91	1
RPL13	3.58E-91	0.512485114	0.991	0.991	7.36E-87	1
APOA1	3.59E-90	0.735648586	0.97	0.892	7.40E-86	1
RPL22	5.07E-90	0.801639088	0.885	0.939	1.04E-85	1
RPL3	4.12E-89	0.579421393	0.964	0.966	8.48E-85	1
RBP4	1.86E-86	0.690313495	0.944	0.817	3.83E-82	1
RPLP2	1.74E-85	0.573186834	0.949	0.964	3.59E-81	1
RPL27	3.12E-85	0.755565145	0.869	0.915	6.42E-81	1
RPL27A	4.36E-85	0.666509979	0.882	0.946	8.98E-81	1
RPL13A	5.39E-85	0.549813987	0.937	0.965	1.11E-80	1
RPL10A	8.03E-84	0.721429801	0.902	0.944	1.65E-79	1
AMBP	3.27E-83	0.834420923	0.879	0.721	6.74E-79	1
APOH	9.18E-83	0.773493392	0.879	0.682	1.89E-78	1
EEF1B2	1.14E-81	0.846916331	0.837	0.9	2.35E-77	1
RPL6	1.21E-81	0.647511306	0.911	0.955	2.50E-77	1
RPS21	1.91E-79	0.738201981	0.863	0.924	3.94E-75	1

RPS24	2.79E-79	0.65853245	0.941	0.976	5.75E-75	1
FTL	8.32E-79	0.375280222	1	0.997	1.71E-74	1
RPL7	1.07E-78	0.745661517	0.872	0.929	2.21E-74	1
RPS4X	1.26E-78	0.737618304	0.914	0.956	2.60E-74	1
RPL8	3.04E-78	0.564488265	0.916	0.963	6.27E-74	1
APOC1	4.06E-78	0.594885716	0.919	0.772	8.36E-74	1
RPLP0	4.10E-76	0.607715276	0.866	0.896	8.43E-72	1
RPL38	9.02E-74	0.71080079	0.827	0.901	1.86E-69	1
RPL30	8.81E-73	0.635595784	0.947	0.977	1.81E-68	1
CYB5A	8.48E-70	0.896503257	0.756	0.626	1.75E-65	1
NACA	7.52E-68	0.630633949	0.881	0.944	1.55E-63	1
RPS2	9.27E-68	0.518072725	0.962	0.977	1.91E-63	1
RPS16	7.85E-67	0.55472213	0.926	0.962	1.62E-62	1
RPS13	3.41E-66	0.687405775	0.919	0.958	7.01E-62	1
RPS15A	3.60E-66	0.586690385	0.944	0.968	7.41E-62	1
RPS23	2.61E-64	0.59627825	0.946	0.978	5.38E-60	1
RPLP1	5.31E-60	0.486558187	0.986	0.986	1.09E-55	1
RARRES2	8.50E-59	0.812253274	0.729	0.523	1.75E-54	1
RPL18A	2.48E-57	0.515759339	0.929	0.965	5.11E-53	1
RPS5	3.21E-57	0.544658162	0.864	0.932	6.62E-53	1
RPS3	8.27E-57	0.469214135	0.935	0.964	1.70E-52	1
APOM	1.10E-56	0.76578992	0.799	0.678	2.26E-52	1
RPS14	5.67E-56	0.514400036	0.943	0.977	1.17E-51	1
RPL10	1.43E-53	0.440218122	0.98	0.988	2.95E-49	1
RPS3A	3.13E-53	0.549080329	0.935	0.966	6.45E-49	1
RPL18	2.87E-52	0.408654087	0.926	0.964	5.92E-48	1
RPL31	4.22E-52	0.887524025	0.718	0.811	8.70E-48	1
RPL12	4.26E-52	0.610314815	0.894	0.956	8.78E-48	1
RPS11	8.85E-49	0.548606217	0.89	0.943	1.82E-44	1
RPL21	1.12E-46	0.557656956	0.919	0.958	2.31E-42	1
IGFBP1	3.78E-46	0.531682546	0.852	0.762	7.78E-42	1
MGST1	4.09E-46	0.812757486	0.63	0.464	8.43E-42	1
AGT	1.04E-45	0.812342747	0.771	0.616	2.14E-41	1
RPSA	1.80E-45	0.469260958	0.89	0.928	3.70E-41	1
RPL7A	6.40E-45	0.406976581	0.922	0.959	1.32E-40	1
RPL9	1.45E-44	0.439761311	0.917	0.956	2.99E-40	1
APOE	1.79E-44	0.521961909	0.973	0.916	3.69E-40	1
GPC3	5.77E-44	0.805562529	0.784	0.68	1.19E-39	1
RPS25	1.43E-41	0.468636469	0.866	0.952	2.94E-37	1
RPL36A	2.45E-41	0.721687839	0.695	0.782	5.04E-37	1
ORM1	2.58E-41	0.86280172	0.62	0.461	5.31E-37	1
RPS20	2.88E-41	0.527606303	0.792	0.905	5.93E-37	1
RPS12	3.21E-40	0.447009737	0.94	0.973	6.61E-36	1
RPL26	1.06E-39	0.492061782	0.908	0.96	2.18E-35	1
DBI	5.43E-39	0.670472906	0.722	0.783	1.12E-34	1

CYP2E1	8.93E-39	0.685598661	0.67	0.497	1.84E-34	1
RPL36	3.10E-38	0.449407136	0.84	0.931	6.39E-34	1
AGXT	1.43E-37	0.578576091	0.716	0.535	2.94E-33	1
RPL4	1.51E-37	0.551689567	0.769	0.869	3.12E-33	1
GSTA1	1.66E-37	0.816986731	0.57	0.411	3.41E-33	1
HINT1	3.64E-37	0.583916313	0.757	0.845	7.49E-33	1
VTN	2.75E-36	0.716915441	0.676	0.521	5.67E-32	1
CHCHD10	7.05E-36	0.738350187	0.652	0.666	1.45E-31	1
RPL41	2.60E-35	0.371980198	0.961	0.985	5.35E-31	1
PEBP1	1.17E-32	0.64351322	0.719	0.75	2.42E-28	1
HP	1.73E-32	0.94882639	0.671	0.587	3.57E-28	1
RPS8	3.07E-32	0.497964254	0.872	0.947	6.32E-28	1
DDT	2.67E-30	0.744719146	0.644	0.655	5.50E-26	1
RPL34	9.37E-29	0.405227944	0.89	0.96	1.93E-24	1
AKR1C3	1.55E-28	0.75502792	0.543	0.434	3.18E-24	1
RPS19	1.61E-28	0.272936615	0.956	0.977	3.31E-24	1
IFITM3	4.66E-28	0.626259493	0.667	0.651	9.60E-24	1
RACK1	2.11E-27	0.441483964	0.793	0.914	4.35E-23	1
APOB	2.50E-27	0.788340183	0.827	0.751	5.14E-23	1
COMMD6	3.37E-27	0.690061295	0.635	0.748	6.94E-23	1
ORM2	4.72E-27	0.806021183	0.517	0.383	9.71E-23	1
RPL23	7.37E-27	0.432633784	0.771	0.893	1.52E-22	1
TTR	1.52E-26	0.649170402	0.587	0.464	3.12E-22	1
ITIH1	1.35E-25	0.634104903	0.608	0.467	2.77E-21	1
CES1	3.27E-25	0.897617414	0.611	0.518	6.74E-21	1
TMEM176A	2.99E-24	0.547154039	0.683	0.625	6.16E-20	1
RPS29	1.06E-23	0.342351829	0.848	0.937	2.18E-19	1
FTH1	1.08E-23	0.295815838	0.964	0.983	2.22E-19	1
TXN	1.98E-23	0.650205986	0.649	0.739	4.08E-19	1
BRI3	4.96E-23	0.561415115	0.664	0.693	1.02E-18	1
UBA52	2.83E-22	0.372900447	0.819	0.932	5.84E-18	1
AKR1C2	6.06E-22	0.745346294	0.484	0.383	1.25E-17	1
AKR1C1	6.62E-22	0.733767013	0.486	0.383	1.36E-17	1
GCSH	3.86E-21	0.810245778	0.468	0.394	7.95E-17	1
AZGP1	8.64E-20	0.606332492	0.599	0.499	1.78E-15	1
MPC2	1.09E-19	0.702032779	0.558	0.586	2.24E-15	1
ANGPTL3	1.09E-19	0.729830788	0.475	0.366	2.25E-15	1
RPL23A	5.27E-19	0.363047737	0.824	0.933	1.08E-14	1
GC	6.95E-19	0.734831754	0.514	0.427	1.43E-14	1
SERPINC1	1.25E-18	0.724824419	0.544	0.458	2.57E-14	1
TPT1	1.85E-18	0.398111904	0.988	0.994	3.82E-14	1
DCXR	6.03E-18	0.704207118	0.551	0.543	1.24E-13	1
FGB	7.64E-18	0.680867998	0.528	0.43	1.57E-13	1
KRT18	6.70E-17	0.68322346	0.46	0.372	1.38E-12	1
ATP5F1E	7.29E-17	0.383848058	0.759	0.907	1.50E-12	1

ARG1	9.91E-17	0.681872415	0.434	0.352	2.04E-12	1
CCL20	1.37E-16	0.630911514	0.483	0.392	2.81E-12	1
ATP5MF	2.70E-16	0.578095959	0.615	0.736	5.55E-12	1
CP	3.99E-16	0.740162568	0.647	0.584	8.21E-12	1
PCBD1	6.36E-15	0.693946324	0.508	0.522	1.31E-10	1
HACD3	3.99E-14	0.257841345	0.169	0.354	8.23E-10	1
TMCO1	1.67E-12	0.283520838	0.288	0.529	3.43E-08	1
TF	2.14E-12	0.664306337	0.554	0.516	4.41E-08	1
KRT8	2.18E-12	0.620727651	0.421	0.35	4.50E-08	1
HPD	4.09E-12	0.745787654	0.351	0.281	8.43E-08	1
UQCRH	6.63E-12	0.499926908	0.599	0.756	1.37E-07	1
CLU	9.45E-12	0.70580689	0.472	0.417	1.95E-07	1
METTL7A	1.36E-11	0.254542612	0.24	0.436	2.80E-07	1
TMEM176B	1.54E-11	0.441222737	0.64	0.625	3.18E-07	1
GSTO1	1.73E-11	0.508385814	0.59	0.697	3.56E-07	1
AADAC	1.75E-11	0.638596202	0.416	0.344	3.60E-07	1
EIF2S3	3.04E-11	0.252417283	0.238	0.444	6.26E-07	1
TXNL1	3.97E-11	0.261956891	0.264	0.472	8.17E-07	1
COX7A2L	5.02E-11	0.628474076	0.517	0.582	1.03E-06	1
ATP5MC3	7.77E-11	0.521327959	0.585	0.718	1.60E-06	1
GPX4	9.64E-11	0.403233817	0.649	0.774	1.99E-06	1
HDGF	1.35E-10	0.262460066	0.268	0.476	2.77E-06	1
QARS	1.46E-10	0.260054861	0.216	0.398	3.00E-06	1
IGFBP2	6.44E-10	0.530363553	0.495	0.445	1.33E-05	1
NUPR1	6.62E-10	0.571893292	0.431	0.39	1.36E-05	1
AKR1C4	7.13E-10	0.605874032	0.376	0.315	1.47E-05	1
MPST	9.57E-10	0.538553349	0.495	0.474	1.97E-05	1
F2	1.56E-09	0.639704264	0.468	0.418	3.21E-05	1
ALDH6A1	1.05E-08	0.327431316	0.167	0.303	0.000215415	1
SOD1	1.37E-08	0.436155751	0.632	0.775	0.000282762	1
SLC22A18	1.52E-08	0.342754525	0.183	0.335	0.000313479	1
NIFK	1.57E-08	0.297664856	0.19	0.346	0.000323311	1
FIS1	1.72E-08	0.607201634	0.52	0.618	0.000353667	1
COMT	1.85E-08	0.282517844	0.284	0.475	0.000381886	1
TFPI	1.97E-08	0.570734301	0.452	0.405	0.000406523	1
LSM4	2.26E-08	0.276250794	0.265	0.458	0.000465426	1
EIF3E	4.19E-08	0.550429755	0.569	0.717	0.000863596	1
COPS6	5.86E-08	0.272648756	0.296	0.488	0.001207196	1
SIGMAR1	5.98E-08	0.265004336	0.17	0.302	0.001232081	1
TM4SF4	6.00E-08	0.610591072	0.407	0.367	0.001235462	1
YIF1A	7.73E-08	0.273740067	0.262	0.435	0.001591175	1
ALKBH7	9.61E-08	0.254259483	0.287	0.478	0.001980172	1
CISD1	1.34E-07	0.260373554	0.175	0.308	0.002753746	1
SDC1	2.03E-07	0.258183011	0.173	0.298	0.004180686	1
COX5B	2.30E-07	0.419821216	0.594	0.754	0.004735996	1

FBL	2.30E-07	0.287907964	0.275	0.454	0.004745419	1
TPRKB	2.76E-07	0.252817702	0.204	0.348	0.005690626	1
SUCLG2	5.00E-07	0.285396489	0.238	0.399	0.010292648	1
ITIH2	5.11E-07	0.516989016	0.428	0.387	0.010515329	1
PMVK	5.28E-07	0.306179149	0.225	0.378	0.010865658	1
MRPL52	5.63E-07	0.279180402	0.303	0.491	0.011604893	1
PCNP	7.38E-07	0.310086647	0.287	0.469	0.015191576	1
GGCT	1.46E-06	0.267779464	0.151	0.261	0.030164921	1
C3	1.73E-06	0.734322042	0.543	0.557	0.035681602	1
MDK	1.77E-06	0.725946822	0.333	0.303	0.036382381	1
CNIH1	1.98E-06	0.280248689	0.264	0.431	0.040803137	1
HTATIP2	2.12E-06	0.256787618	0.238	0.387	0.043732629	1
SERPINF1	2.12E-06	0.29985448	0.158	0.271	0.04374434	1
PTGR1	2.53E-06	0.584339317	0.425	0.413	0.052025101	1
RAB5IF	2.78E-06	0.303222647	0.311	0.501	0.057206543	1
NELFE	3.01E-06	0.298467505	0.261	0.424	0.061933107	1
PRDX6	3.18E-06	0.563237937	0.492	0.587	0.065395884	1
MGST2	3.60E-06	0.253877178	0.284	0.449	0.074039865	1
P4HB	4.10E-06	0.34531721	0.427	0.666	0.084455952	1
MRPL40	4.37E-06	0.322828152	0.243	0.397	0.089986995	1
OSTC	5.06E-06	0.294138783	0.324	0.524	0.104131219	1
TAF9	5.18E-06	0.282582638	0.238	0.392	0.106597592	1
EIF2S2	5.44E-06	0.258122274	0.347	0.564	0.111938651	1
C5	5.56E-06	0.261233035	0.164	0.27	0.114471441	1
AKR1B10	6.14E-06	0.659311161	0.278	0.232	0.126539667	1
UGDH	6.22E-06	0.305431421	0.195	0.316	0.12819504	1
CERS2	6.41E-06	0.277046524	0.279	0.43	0.132114401	1
TMEM101	6.80E-06	0.317372089	0.176	0.294	0.140002296	1
SCAND1	8.65E-06	0.26477115	0.332	0.539	0.178239526	1
UQCRB	9.11E-06	0.312020253	0.627	0.829	0.187524729	1
TMEM18	9.50E-06	0.285293897	0.167	0.279	0.195701333	1
CLDN1	9.60E-06	0.316895279	0.173	0.28	0.197635091	1
NSA2	9.60E-06	0.262705693	0.338	0.541	0.197681657	1
HSPD1	9.64E-06	0.279728417	0.715	0.834	0.198458241	1
LDHA	1.14E-05	0.340718801	0.674	0.772	0.235215008	1
COX11	1.19E-05	0.264465671	0.176	0.288	0.24417448	1
MZT2B	1.31E-05	0.444082084	0.56	0.715	0.269445888	1
CLNS1A	1.78E-05	0.311741292	0.287	0.458	0.36619601	1
PEG10	1.94E-05	0.548934464	0.388	0.359	0.399325189	1
RAB13	1.96E-05	0.580808924	0.431	0.456	0.40358505	1
POR	2.05E-05	0.345484072	0.285	0.435	0.422847779	1
ZNF593	2.21E-05	0.252140129	0.189	0.306	0.454268062	1
DCPS	2.31E-05	0.261892573	0.225	0.351	0.475479413	1
NPM1	2.37E-05	0.368640018	0.627	0.812	0.487690659	1
RSL1D1	2.46E-05	0.281928239	0.306	0.484	0.507353465	1

AURKAIP1	2.68E-05	0.275958408	0.357	0.576	0.551719599	1
HDLBP	3.25E-05	0.276200579	0.315	0.484	0.670178089	1
SUMO1	3.44E-05	0.320803297	0.363	0.581	0.708745465	1
CFHR1	3.48E-05	0.565607281	0.377	0.352	0.715981085	1
PROS1	3.56E-05	0.337988843	0.181	0.29	0.734108595	1
NDUFAF8	3.69E-05	0.338534069	0.27	0.434	0.76096139	1
NDUFB3	4.12E-05	0.285428093	0.333	0.541	0.848980865	1
GGH	4.47E-05	0.280515068	0.187	0.294	0.920211508	1
PROC	4.71E-05	0.512907156	0.383	0.362	0.969720194	1
SNRPD3	5.65E-05	0.292678051	0.362	0.576	1	1
TM4SF5	7.16E-05	0.530278996	0.35	0.322	1	1
TUSC2	7.37E-05	0.303845596	0.22	0.345	1	1
FN1	7.88E-05	0.651630656	0.528	0.54	1	1
EIF6	8.46E-05	0.31080478	0.33	0.525	1	1
PCOLCE	8.75E-05	0.260361085	0.178	0.276	1	1
BTF3	9.33E-05	0.300311644	0.643	0.846	1	1
F10	9.60E-05	0.525222462	0.406	0.386	1	1
SDHC	0.000111678	0.315078923	0.338	0.531	1	1
GAMT	0.000122254	0.531616356	0.365	0.371	1	1
SERPING1	0.00013419	0.293309093	0.314	0.464	1	1
PPA1	0.000155887	0.481830499	0.478	0.565	1	1
MAT1A	0.00016369	0.259334133	0.208	0.305	1	1
CENPX	0.000164999	0.281881733	0.25	0.387	1	1
TTC39C	0.000174679	0.252008717	0.198	0.304	1	1
POLD2	0.000175503	0.268938892	0.196	0.302	1	1
ZNHIT1	0.000208008	0.542411778	0.481	0.592	1	1
LBP	0.000222762	0.7950637	0.326	0.309	1	1
CHCHD2	0.000232497	0.33998927	0.63	0.828	1	1
CUTA	0.000237753	0.41221035	0.526	0.662	1	1
ZSCAN16-AS1	0.000251807	0.308948798	0.183	0.286	1	1
TMEM256	0.000282034	0.274629349	0.281	0.43	1	1
SUCLG1	0.000282614	0.281223555	0.306	0.472	1	1
MSRB1	0.00029669	0.268022212	0.208	0.32	1	1
TOMM22	0.000304228	0.299241987	0.314	0.494	1	1
RNF181	0.000309525	0.29467096	0.338	0.528	1	1
CFL2	0.000320496	0.302025071	0.186	0.284	1	1
MRPL9	0.000329022	0.263195818	0.219	0.333	1	1
FAM213A	0.000346765	0.250025763	0.276	0.404	1	1
FMC1	0.000372327	0.271325323	0.205	0.314	1	1
S100A10	0.000433628	0.335362003	0.615	0.764	1	1
SAA4	0.000445565	0.53589347	0.348	0.335	1	1
SNRPN	0.000450605	0.253366662	0.281	0.419	1	1
FAM177A1	0.000595971	0.292927137	0.297	0.455	1	1
RNF5	0.000608596	0.279009767	0.273	0.416	1	1

STOML2	0.00062015	0.306819876	0.291	0.444	1	1
MRPL13	0.000631354	0.374232216	0.243	0.366	1	1
HAO1	0.000698125	0.540692521	0.312	0.294	1	1
ATP5PD	0.000859513	0.329502625	0.394	0.611	1	1
SRP9	0.001034689	0.325907073	0.363	0.565	1	1
S100A13	0.00113134	0.550286833	0.279	0.27	1	1
CFB	0.001250418	0.356218485	0.184	0.27	1	1
DAP3	0.001413281	0.305453615	0.288	0.431	1	1
DNAJC19	0.001413449	0.366583477	0.278	0.411	1	1
CCDC167	0.001493206	0.314663799	0.231	0.344	1	1
PDIA4	0.001541296	0.353957863	0.353	0.516	1	1
C2	0.001552956	0.375660768	0.501	0.524	1	1
AQP9	0.001565973	0.28488182	0.198	0.284	1	1
OLA1	0.001649419	0.357983709	0.255	0.385	1	1
MRPS18B	0.001714429	0.35105636	0.291	0.436	1	1
ADI1	0.001715257	0.525054566	0.442	0.515	1	1
COX7C	0.001723572	0.277937361	0.614	0.817	1	1
PABPC1	0.00179603	0.265179766	0.667	0.828	1	1
CAMK2N1	0.0017998	0.457592121	0.33	0.328	1	1
APCS	0.001870326	0.570413075	0.294	0.273	1	1
HAAO	0.001916591	0.307731542	0.189	0.277	1	1
LSM3	0.001972213	0.31888538	0.359	0.548	1	1
TMEM141	0.001974238	0.325870546	0.267	0.391	1	1
POLR2I	0.002046162	0.275738417	0.291	0.434	1	1
HIST1H2AC	0.002134796	0.288646266	0.268	0.386	1	1
CSNK2B	0.002198916	0.307248135	0.392	0.603	1	1
SEC11C	0.002314373	0.342123716	0.339	0.499	1	1
A1BG	0.002448208	0.478581551	0.422	0.438	1	1
NAT8	0.002482457	0.268960246	0.183	0.26	1	1
GATM	0.002923775	0.467178633	0.431	0.465	1	1
DNPH1	0.002966772	0.250971361	0.338	0.512	1	1
TM7SF2	0.003007624	0.301431193	0.181	0.26	1	1
VIL1	0.003271474	0.314497293	0.183	0.258	1	1
AFMID	0.00345843	0.353366181	0.205	0.297	1	1
SWI5	0.003481201	0.314451456	0.179	0.263	1	1
SNRPD2	0.003488104	0.285904686	0.389	0.599	1	1
PERP	0.003682605	0.258973687	0.175	0.251	1	1
NDUFA5	0.003788321	0.358493442	0.326	0.491	1	1
ROMO1	0.003916851	0.338723857	0.302	0.461	1	1
SORD	0.00419823	0.308937742	0.226	0.321	1	1
ASL	0.004303327	0.356768192	0.264	0.371	1	1
MIF	0.00439506	0.570401534	0.448	0.558	1	1
PIN4	0.004582586	0.284278936	0.235	0.344	1	1
FGG	0.005496396	0.525923889	0.335	0.326	1	1
ACADSB	0.006130637	0.306062153	0.225	0.308	1	1

PDCD5	0.006912454	0.357107109	0.315	0.471	1	1
PFDN4	0.007065056	0.285921576	0.258	0.376	1	1
NDUFB2	0.007482977	0.261503349	0.443	0.692	1	1
POP7	0.007543731	0.323152452	0.208	0.301	1	1
PGRMC1	0.007688206	0.373971782	0.302	0.422	1	1
KNG1	0.008031688	0.510982105	0.373	0.363	1	1
GLYCTK	0.008133624	0.270059464	0.241	0.326	1	1
POLR2F	0.009156627	0.359429739	0.315	0.463	1	1
FAM136A	0.009504033	0.291055009	0.265	0.381	1	1
EBPL	0.009694683	0.283405232	0.24	0.335	1	1
LGMN	0	2.549727756	0.998	0.24	0	2
C1QB	0	2.453299987	0.995	0.193	0	2
RNASE1	0	2.359327686	0.678	0.084	0	2
C1QA	0	2.33883989	0.995	0.203	0	2
GPNMB	0	2.264702078	0.965	0.102	0	2
C1QC	0	2.243689888	0.993	0.121	0	2
CTSB	0	2.234955165	1	0.616	0	2
SLC40A1	0	2.081416593	0.945	0.299	0	2
LIPA	0	2.04443225	0.988	0.344	0	2
CD68	0	1.871617875	0.997	0.274	0	2
PSAP	0	1.828281913	1	0.607	0	2
MS4A7	0	1.772587418	0.966	0.121	0	2
CD14	0	1.73560876	0.963	0.198	0	2
PLD3	0	1.716410359	0.971	0.283	0	2
DAB2	0	1.70928591	0.977	0.101	0	2
CTSZ	0	1.687148555	0.998	0.512	0	2
LGALS3	0	1.674555239	0.98	0.216	0	2
FOLR2	0	1.668913368	0.926	0.053	0	2
TYROBP	0	1.635836083	0.998	0.254	0	2
HLA-DPB1	0	1.604496983	0.997	0.333	0	2
NPC2	0	1.566569661	0.998	0.502	0	2
MS4A6A	0	1.558062057	0.956	0.162	0	2
FCGR3A	0	1.523349543	0.968	0.145	0	2
FCER1G	0	1.521867865	0.99	0.262	0	2
MS4A4A	0	1.511925455	0.933	0.085	0	2
CD163	0	1.498077701	0.918	0.059	0	2
ACP5	0	1.459470752	0.896	0.128	0	2
MSR1	0	1.453522536	0.93	0.06	0	2
CTSS	0	1.447856734	0.993	0.305	0	2
HMOX1	0	1.436026327	0.945	0.158	0	2
HLA-DMA	0	1.420858432	0.985	0.262	0	2
MAFB	0	1.39760024	0.975	0.209	0	2
HLA-DMB	0	1.394357837	0.965	0.153	0	2
IGSF6	0	1.3756724	0.953	0.131	0	2
FCGR2A	0	1.373766365	0.968	0.121	0	2

HLA-DRB1	0	1.371342028	0.987	0.267	0	2
CTSL	0	1.368331653	0.97	0.332	0	2
MRC1	0	1.366670049	0.906	0.08	0	2
AIF1	0	1.354439371	0.993	0.235	0	2
A2M	0	1.348650023	0.95	0.217	0	2
TIMP2	0	1.34073067	0.977	0.136	0	2
HLA-DQA1	0	1.333691565	0.978	0.188	0	2
CPVL	0	1.331322136	0.941	0.19	0	2
CREG1	0	1.326756797	0.973	0.175	0	2
PLTP	0	1.264484014	0.841	0.08	0	2
PLA2G7	0	1.250150156	0.906	0.086	0	2
CXCL16	0	1.224898856	0.948	0.207	0	2
STAB1	0	1.220192328	0.872	0.099	0	2
MMP9	0	1.212378253	0.643	0.064	0	2
MPEG1	0	1.207569324	0.945	0.118	0	2
TGFBI	0	1.200912664	0.955	0.212	0	2
FGL2	0	1.183474401	0.928	0.167	0	2
ADA2	0	1.175456585	0.958	0.138	0	2
HLA-DQB1	0	1.165956236	0.97	0.203	0	2
SGK1	0	1.161127025	0.928	0.188	0	2
RNASE6	0	1.161066082	0.926	0.104	0	2
HLA-DQA2	0	1.123352748	0.94	0.163	0	2
TREM2	0	1.122883647	0.696	0.033	0	2
CD4	0	1.107286225	0.971	0.19	0	2
KCTD12	0	1.092668236	0.923	0.115	0	2
LILRB5	0	1.080965167	0.826	0.025	0	2
CSF1R	0	1.074133011	0.903	0.094	0	2
SDC3	0	1.073993988	0.857	0.055	0	2
SLCO2B1	0	1.058976419	0.903	0.15	0	2
CYBB	0	1.048211527	0.904	0.121	0	2
C3AR1	0	1.008086863	0.888	0.071	0	2
ADAP2	0	0.998606012	0.909	0.091	0	2
NPL	0	0.992521589	0.888	0.061	0	2
C5AR1	0	0.987248149	0.8	0.074	0	2
RNF130	0	0.986984873	0.936	0.243	0	2
FPR3	0	0.977497161	0.874	0.064	0	2
LY96	0	0.969434708	0.935	0.13	0	2
TSPAN4	0	0.922608545	0.924	0.232	0	2
CD86	0	0.920300896	0.888	0.094	0	2
TNFAIP2	0	0.918702355	0.893	0.156	0	2
MPP1	0	0.907301929	0.861	0.077	0	2
CD209	0	0.906585709	0.721	0.03	0	2
MFSD1	0	0.898808708	0.896	0.229	0	2
CD84	0	0.894016235	0.866	0.126	0	2
LAIR1	0	0.886813902	0.878	0.099	0	2

SLC15A3	0	0.880487441	0.854	0.077	0	2
TNFSF13B	0	0.861115472	0.864	0.121	0	2
VSIG4	0	0.843763973	0.656	0.058	0	2
IGF1	0	0.833983993	0.653	0.024	0	2
GPR34	0	0.823235056	0.752	0.035	0	2
MCOLN1	0	0.789145065	0.773	0.123	0	2
GM2A	0	0.788400923	0.795	0.155	0	2
SPI1	0	0.786341534	0.913	0.136	0	2
BLVRA	0	0.768185935	0.854	0.17	0	2
FRMD4B	0	0.766195168	0.829	0.125	0	2
RAB20	0	0.763884076	0.826	0.076	0	2
IL18BP	0	0.756379377	0.755	0.079	0	2
F13A1	0	0.75206838	0.465	0.033	0	2
C1orf54	0	0.75086388	0.762	0.075	0	2
RAB31	0	0.749541918	0.842	0.108	0	2
VMO1	0	0.749074834	0.628	0.039	0	2
PRNP	0	0.738279337	0.871	0.133	0	2
1-Mar	0	0.737441032	0.831	0.092	0	2
CCR1	0	0.737132839	0.757	0.076	0	2
HLA-DOA	0	0.736654693	0.758	0.06	0	2
THEMIS2	0	0.736033287	0.856	0.124	0	2
BCAT1	0	0.729058978	0.733	0.056	0	2
LILRB4	0	0.721533513	0.785	0.066	0	2
SIGLEC1	0	0.71591357	0.725	0.029	0	2
TMIGD3	0	0.702290045	0.529	0.021	0	2
LYN	0	0.699444021	0.898	0.177	0	2
LY86	0	0.689864522	0.799	0.091	0	2
LGALS9	0	0.685070074	0.857	0.141	0	2
ITGAX	0	0.681799012	0.805	0.098	0	2
BMP2K	0	0.679929372	0.745	0.096	0	2
FCHO2	0	0.6793127	0.742	0.099	0	2
COLEC12	0	0.676936588	0.502	0.019	0	2
CD93	0	0.675450503	0.718	0.097	0	2
PLAUR	0	0.673300466	0.762	0.113	0	2
UNC93B1	0	0.67026436	0.81	0.157	0	2
BASP1	0	0.666177973	0.79	0.112	0	2
SLC7A7	0	0.65948211	0.782	0.082	0	2
KCNMA1	0	0.658429973	0.669	0.02	0	2
MITF	0	0.654772494	0.666	0.05	0	2
NRP2	0	0.650824701	0.705	0.05	0	2
SLAMF8	0	0.627689252	0.711	0.061	0	2
IL18	0	0.626839548	0.72	0.055	0	2
PILRA	0	0.621480368	0.753	0.086	0	2
GNB4	0	0.620526363	0.763	0.078	0	2
EPB41L3	0	0.611697192	0.738	0.053	0	2

AKR1B1	0	0.610109669	0.767	0.118	0	2
SLC1A3	0	0.598909473	0.612	0.032	0	2
HAVCR2	0	0.596047541	0.768	0.111	0	2
SLC7A8	0	0.596034679	0.606	0.018	0	2
ARHGAP18	0	0.591122283	0.762	0.103	0	2
LILRB2	0	0.590949764	0.748	0.089	0	2
CD300A	0	0.587592494	0.78	0.108	0	2
FRMD4A	0	0.5874936	0.646	0.041	0	2
IRF8	0	0.582918606	0.76	0.094	0	2
CD163L1	0	0.581182363	0.54	0.023	0	2
RGL1	0	0.580558623	0.649	0.052	0	2
HCK	0	0.576268708	0.792	0.104	0	2
CD72	0	0.572057241	0.614	0.058	0	2
AXL	0	0.570287859	0.614	0.034	0	2
CMKLR1	0	0.568456126	0.701	0.049	0	2
DHRS9	0	0.566112554	0.438	0.021	0	2
EMILIN2	0	0.551702423	0.721	0.077	0	2
SPRED1	0	0.55143671	0.624	0.046	0	2
ME1	0	0.550827637	0.589	0.038	0	2
RAB7B	0	0.542470874	0.426	0.017	0	2
NCF2	0	0.539450847	0.797	0.117	0	2
FMNL2	0	0.527982774	0.614	0.038	0	2
OTOA	0	0.527978747	0.592	0.029	0	2
SASH1	0	0.52731643	0.612	0.053	0	2
LRRC25	0	0.526159407	0.711	0.081	0	2
GPR137B	0	0.525278955	0.695	0.06	0	2
PTAFR	0	0.522911798	0.659	0.053	0	2
TBXAS1	0	0.521072927	0.708	0.089	0	2
FABP3	0	0.512986657	0.54	0.016	0	2
TLR4	0	0.509847049	0.69	0.059	0	2
PLEKHO2	0	0.502519875	0.703	0.086	0	2
SYK	0	0.490291184	0.7	0.08	0	2
PLA2G15	0	0.471957968	0.582	0.056	0	2
GFRA2	0	0.469301553	0.497	0.026	0	2
SUCNR1	0	0.46830837	0.495	0.037	0	2
SIGLEC7	0	0.463606378	0.619	0.055	0	2
TCN2	0	0.461797731	0.624	0.056	0	2
CD33	0	0.456744148	0.611	0.058	0	2
SLC8A1	0	0.440412442	0.624	0.068	0	2
GAS7	0	0.437827658	0.601	0.048	0	2
TMEM51	0	0.424988129	0.512	0.032	0	2
TFEC	0	0.422652103	0.607	0.055	0	2
LHFPL2	0	0.415210253	0.478	0.024	0	2
FAM198B	0	0.414750279	0.589	0.052	0	2
PMP22	0	0.390738115	0.547	0.051	0	2

OSBPL1A	0	0.355109204	0.505	0.032	0	2
CD180	0	0.350509275	0.49	0.036	0	2
ADORA3	0	0.309158321	0.374	0.009	0	2
KCNJ5	0	0.304994038	0.347	0.005	0	2
MRC2	0	0.264674604	0.384	0.015	0	2
MERTK	2.12E-307	0.56151874	0.649	0.094	4.36E-303	2
ZEB2	2.32E-307	0.759934805	0.926	0.178	4.77E-303	2
SH2B3	2.86E-306	0.588211683	0.777	0.13	5.89E-302	2
NAIP	5.70E-306	0.473980855	0.674	0.092	1.17E-301	2
ABCA1	1.24E-305	1.090194313	0.921	0.289	2.55E-301	2
ASAH1	2.73E-304	1.320575243	0.982	0.412	5.61E-300	2
ABCG1	1.04E-303	0.415608347	0.634	0.072	2.14E-299	2
LILRB3	4.25E-303	0.495248089	0.661	0.083	8.76E-299	2
LTC4S	1.57E-302	0.351265022	0.441	0.028	3.23E-298	2
RNASET2	2.56E-302	1.151291037	0.971	0.318	5.28E-298	2
HLA-DPA1	2.29E-301	1.675463442	1	0.396	4.72E-297	2
NRP1	9.62E-301	0.719345273	0.779	0.154	1.98E-296	2
LILRB1	3.88E-300	0.479604195	0.659	0.082	7.99E-296	2
SPINT2	3.75E-299	0.444272524	0.68	0.088	7.72E-295	2
DMXL2	4.51E-299	0.577763653	0.748	0.133	9.30E-295	2
NCEH1	1.34E-298	0.408811804	0.513	0.048	2.77E-294	2
MMP19	8.18E-298	0.505681193	0.404	0.022	1.68E-293	2
CYBRD1	4.78E-297	0.335494268	0.463	0.033	9.84E-293	2
ADAM9	4.97E-297	0.573791194	0.732	0.135	1.02E-292	2
CTSD	8.65E-297	1.765937604	1	0.652	1.78E-292	2
CDCP1	2.31E-296	0.306034834	0.424	0.026	4.76E-292	2
ABCC5	4.09E-296	0.501044944	0.638	0.094	8.42E-292	2
HLA-DRA	1.40E-295	1.82957428	1	0.47	2.88E-291	2
FAM49A	4.70E-295	0.483034791	0.7	0.097	9.68E-291	2
GRN	2.10E-293	1.32466063	0.992	0.517	4.33E-289	2
FABP5	3.65E-292	1.77317035	0.973	0.341	7.53E-288	2
CD74	1.84E-291	1.83177641	1	0.583	3.78E-287	2
OGFRL1	9.51E-290	0.498276339	0.708	0.105	1.96E-285	2
GSN	6.62E-289	0.842558358	0.948	0.233	1.36E-284	2
CR1	9.94E-289	0.308846263	0.421	0.026	2.05E-284	2
LGALS3BP	4.41E-288	0.500232818	0.678	0.096	9.07E-284	2
C1orf162	2.91E-287	0.706387136	0.891	0.168	6.00E-283	2
CETP	1.49E-286	0.572868845	0.383	0.019	3.06E-282	2
MKNK1	1.44E-285	0.633343206	0.79	0.16	2.97E-281	2
PPT1	1.86E-285	0.89778878	0.936	0.283	3.83E-281	2
MILR1	7.19E-285	0.289995895	0.451	0.033	1.48E-280	2
SHTN1	5.41E-284	0.574752351	0.742	0.145	1.11E-279	2
CFD	2.30E-283	0.85212022	0.674	0.108	4.74E-279	2
SCARB2	6.36E-283	0.834778418	0.924	0.291	1.31E-278	2
ADGRE2	6.55E-283	0.450749854	0.565	0.062	1.35E-278	2

PLAU	1.08E-282	0.640833562	0.515	0.049	2.22E-278	2
MARCKS	3.41E-282	1.365259737	0.992	0.494	7.01E-278	2
TTYH3	4.10E-282	0.700303903	0.792	0.177	8.44E-278	2
MYO5A	3.26E-280	0.372981914	0.581	0.07	6.72E-276	2
FUCA1	1.29E-279	0.927620336	0.876	0.26	2.65E-275	2
FCGRT	5.71E-278	1.238989395	0.988	0.493	1.18E-273	2
GAA	1.42E-276	0.709907499	0.847	0.232	2.92E-272	2
CYFIP1	2.57E-276	0.687670819	0.82	0.211	5.28E-272	2
SLC31A2	9.79E-276	0.433413873	0.619	0.083	2.02E-271	2
SAMHD1	1.03E-275	1.017954805	0.978	0.313	2.12E-271	2
HOMER3	2.68E-275	0.353332127	0.547	0.059	5.52E-271	2
NCKAP1L	6.25E-275	0.603913527	0.864	0.168	1.29E-270	2
DRAM1	1.62E-274	0.535401813	0.638	0.095	3.33E-270	2
CIITA	8.25E-274	0.390824721	0.582	0.069	1.70E-269	2
CLIC2	3.00E-273	0.356531028	0.549	0.065	6.17E-269	2
CTSC	5.06E-273	1.298980075	0.977	0.362	1.04E-268	2
SLC16A3	1.32E-272	0.536754843	0.669	0.099	2.71E-268	2
ATP8B4	2.41E-272	0.316210625	0.465	0.04	4.97E-268	2
ACE	2.89E-272	0.253881498	0.337	0.013	5.95E-268	2
ARRB2	1.16E-271	0.685581395	0.896	0.22	2.39E-267	2
BTK	3.10E-271	0.319283938	0.54	0.058	6.38E-267	2
GNS	7.54E-271	0.795103172	0.893	0.275	1.55E-266	2
TLR1	2.91E-270	0.285687274	0.443	0.035	6.00E-266	2
BLVRB	3.29E-269	1.185866659	0.971	0.409	6.78E-265	2
OTULINL	3.57E-269	0.376204128	0.592	0.072	7.35E-265	2
CD5L	3.68E-268	1.442670363	0.383	0.023	7.59E-264	2
IFNGR2	2.21E-267	0.670557292	0.852	0.221	4.56E-263	2
CLEC7A	2.24E-266	0.443915846	0.666	0.095	4.62E-262	2
PLEK	2.59E-266	0.846699272	0.884	0.19	5.33E-262	2
HRH1	2.66E-266	0.282583537	0.315	0.011	5.48E-262	2
SDS	8.81E-265	0.686597983	0.399	0.027	1.81E-260	2
ITGAM	1.38E-264	0.407161293	0.565	0.067	2.85E-260	2
EPB41L2	3.74E-264	0.710581414	0.72	0.157	7.71E-260	2
ALDH3B1	1.79E-262	0.315508	0.466	0.043	3.69E-258	2
IL4I1	3.94E-262	0.692768908	0.542	0.065	8.11E-258	2
IFNGR1	6.04E-262	0.81134449	0.876	0.222	1.24E-257	2
RBM47	1.16E-261	0.69080147	0.794	0.205	2.39E-257	2
GLUL	4.60E-261	1.334378742	0.987	0.479	9.48E-257	2
RGS10	5.95E-261	0.754304777	0.919	0.208	1.22E-256	2
FERMT3	8.97E-261	0.655964979	0.874	0.185	1.85E-256	2
YBX3	1.62E-260	0.63597644	0.839	0.164	3.34E-256	2
SERPINB6	2.17E-260	0.672450188	0.876	0.23	4.47E-256	2
LPL	7.40E-260	0.587610009	0.342	0.016	1.52E-255	2
ATP6V1B2	2.97E-259	0.728468655	0.889	0.258	6.11E-255	2
SLC38A6	4.34E-259	0.44371442	0.624	0.105	8.93E-255	2

AOAH	9.03E-259	0.525476993	0.676	0.103	1.86E-254	2
PLXDC2	5.08E-258	0.349067908	0.52	0.057	1.05E-253	2
RNF135	1.97E-257	0.304560078	0.502	0.052	4.06E-253	2
STX7	4.80E-256	0.536579694	0.795	0.186	9.88E-252	2
ACVRL1	1.44E-255	0.277066894	0.441	0.037	2.96E-251	2
DPYSL2	2.75E-255	0.542897731	0.799	0.174	5.66E-251	2
SCIMP	6.18E-254	0.349738311	0.537	0.063	1.27E-249	2
SPART	7.52E-254	0.296498195	0.503	0.053	1.55E-249	2
SIRPA	8.21E-254	0.551131436	0.773	0.181	1.69E-249	2
MYO9B	2.17E-253	0.472607237	0.777	0.156	4.47E-249	2
TM6SF1	2.82E-253	0.25771771	0.396	0.028	5.81E-249	2
RAB32	1.03E-252	0.701370829	0.8	0.2	2.12E-248	2
RHOQ	1.53E-252	0.529501449	0.773	0.165	3.16E-248	2
ZNF385A	3.78E-251	0.391157618	0.56	0.072	7.78E-247	2
PFKFB3	4.63E-251	0.516582319	0.582	0.077	9.54E-247	2
SDCBP	1.15E-249	1.053734545	0.978	0.475	2.38E-245	2
ENG	2.41E-249	0.314882451	0.582	0.079	4.96E-245	2
IGSF21	1.73E-247	0.325333077	0.295	0.01	3.56E-243	2
SESN3	1.08E-246	0.341144834	0.512	0.058	2.23E-242	2
CYTH4	1.83E-246	0.487396588	0.725	0.125	3.78E-242	2
CD63	3.60E-246	1.207563127	0.997	0.712	7.41E-242	2
ANXA5	4.94E-246	0.935962406	0.971	0.373	1.02E-241	2
EBI3	5.37E-246	0.316024979	0.416	0.034	1.11E-241	2
RENBP	9.32E-246	0.250200026	0.426	0.037	1.92E-241	2
FTL	2.59E-245	1.26117319	1	0.997	5.33E-241	2
FSCN1	7.23E-244	0.475177252	0.549	0.07	1.49E-239	2
LAPTM5	8.30E-244	1.038658461	0.995	0.393	1.71E-239	2
CCRL2	4.39E-243	0.31533462	0.443	0.041	9.04E-239	2
IL10RA	6.56E-243	0.606775327	0.856	0.185	1.35E-238	2
LST1	1.90E-242	0.601711259	0.881	0.185	3.92E-238	2
NCF4	6.13E-242	0.425477805	0.686	0.113	1.26E-237	2
ITGB2	2.66E-241	0.953280353	0.968	0.294	5.47E-237	2
TPP1	6.49E-241	0.892752287	0.98	0.459	1.34E-236	2
SECTM1	5.73E-239	0.30727797	0.495	0.056	1.18E-234	2
CALHM6	6.54E-239	0.562431373	0.594	0.086	1.35E-234	2
PLEKHA2	7.65E-239	0.386409873	0.631	0.095	1.58E-234	2
SNX9	1.45E-238	0.44966227	0.733	0.155	2.98E-234	2
PARVB	3.10E-238	0.358732186	0.621	0.092	6.39E-234	2
MAF	1.49E-236	0.965539631	0.96	0.379	3.07E-232	2
CTSH	4.72E-236	0.870958316	0.926	0.318	9.73E-232	2
RAB42	1.76E-235	0.377981452	0.411	0.038	3.63E-231	2
RASSF4	2.29E-234	0.564950826	0.815	0.216	4.71E-230	2
GLIPR1	2.94E-234	0.621580993	0.879	0.207	6.05E-230	2
ADCY7	5.65E-234	0.370696649	0.609	0.091	1.16E-229	2
PLEKHO1	5.94E-234	0.612413868	0.839	0.183	1.22E-229	2

PTPRE	6.09E-234	0.563163477	0.785	0.154	1.25E-229	2
MGAT1	2.63E-233	0.756094568	0.923	0.321	5.42E-229	2
SIGLEC9	2.66E-233	0.252858942	0.393	0.032	5.49E-229	2
CST3	1.10E-232	1.254349829	0.998	0.581	2.26E-228	2
GNPDA1	3.03E-231	0.485877582	0.681	0.143	6.23E-227	2
FEZ2	2.01E-230	0.558492071	0.789	0.219	4.14E-226	2
CD83	2.55E-230	0.80389057	0.78	0.175	5.25E-226	2
NAGK	4.86E-230	0.669306475	0.889	0.256	1.00E-225	2
GIMAP8	3.57E-229	0.27212573	0.47	0.051	7.35E-225	2
RNF144B	2.97E-228	0.421058999	0.579	0.098	6.11E-224	2
AP1B1	6.20E-228	0.505476761	0.74	0.179	1.28E-223	2
SELENOP	2.32E-226	1.547517318	0.982	0.627	4.78E-222	2
ATP1B1	5.03E-226	0.764769154	0.871	0.291	1.04E-221	2
M6PR	1.93E-224	0.737134788	0.933	0.346	3.97E-220	2
ARL4C	2.06E-224	0.898152482	0.96	0.293	4.25E-220	2
SLC9A9	7.66E-224	0.277693375	0.445	0.047	1.58E-219	2
GRK3	1.31E-223	0.409941117	0.633	0.12	2.69E-219	2
FGD2	3.33E-223	0.321483867	0.517	0.067	6.86E-219	2
TLR2	4.46E-223	0.284305968	0.453	0.049	9.19E-219	2
VIM	6.01E-223	1.212609343	0.997	0.533	1.24E-218	2
MCTP1	1.22E-222	0.251994896	0.416	0.04	2.50E-218	2
AP2A2	1.96E-220	0.549467863	0.745	0.196	4.03E-216	2
CSF2RA	5.67E-220	0.285588192	0.445	0.048	1.17E-215	2
CD81	6.01E-220	0.918775146	0.965	0.348	1.24E-215	2
SCPEP1	8.32E-220	0.633846491	0.836	0.265	1.71E-215	2
RB1	3.00E-219	0.510308047	0.703	0.163	6.19E-215	2
SLC43A2	2.76E-218	0.438324839	0.596	0.107	5.68E-214	2
FCGR1A	3.35E-218	0.454339223	0.502	0.065	6.89E-214	2
CCL18	1.03E-216	0.636422303	0.275	0.012	2.12E-212	2
SH3BGRL	1.63E-216	0.665395887	0.926	0.265	3.35E-212	2
EVI2B	2.54E-216	0.495356536	0.815	0.176	5.24E-212	2
CCL2	5.55E-216	1.160292234	0.398	0.039	1.14E-211	2
TBC1D9	6.19E-216	0.366617387	0.54	0.087	1.27E-211	2
METRNL	6.40E-216	0.452696858	0.73	0.141	1.32E-211	2
MEF2C	6.72E-216	0.355645761	0.732	0.142	1.38E-211	2
ADAMDEC1	7.28E-216	0.350752478	0.289	0.014	1.50E-211	2
PQLC3	4.25E-215	0.3523511	0.631	0.115	8.75E-211	2
CREBL2	7.18E-215	0.516447646	0.715	0.174	1.48E-210	2
HBEGF	2.86E-214	0.465529012	0.49	0.064	5.89E-210	2
PLBD1	3.27E-214	0.263051197	0.49	0.062	6.74E-210	2
LRP1	8.50E-214	0.62673257	0.837	0.275	1.75E-209	2
TNFRSF1A	1.18E-213	0.534237371	0.839	0.256	2.43E-209	2
BEX4	5.93E-213	0.391270741	0.622	0.113	1.22E-208	2
LAMP1	8.58E-213	0.887934692	0.97	0.491	1.77E-208	2
VAV1	6.01E-212	0.284676366	0.53	0.074	1.24E-207	2

HEXA	3.31E-211	0.611044272	0.842	0.282	6.81E-207	2
SAT1	3.47E-211	1.111672562	1	0.699	7.15E-207	2
ACP2	2.86E-210	0.539241774	0.743	0.21	5.90E-206	2
GPR183	5.18E-210	0.613776747	0.622	0.11	1.07E-205	2
DRAM2	8.10E-210	0.61028396	0.831	0.264	1.67E-205	2
PHACTR1	1.54E-209	0.454455329	0.562	0.088	3.17E-205	2
S100A11	2.20E-209	1.036565168	0.99	0.44	4.54E-205	2
OTUD1	4.44E-209	0.519762555	0.626	0.118	9.15E-205	2
DOCK2	6.19E-209	0.373270297	0.658	0.118	1.28E-204	2
SGPL1	1.53E-208	0.482023904	0.688	0.173	3.16E-204	2
KCNAB2	4.97E-208	0.300214939	0.56	0.084	1.02E-203	2
DNASE1L3	7.02E-208	1.203998809	0.47	0.064	1.44E-203	2
RAP2B	1.95E-207	0.615959877	0.859	0.276	4.02E-203	2
MFSD12	2.22E-207	0.44134798	0.649	0.141	4.57E-203	2
CHST15	9.81E-207	0.313144566	0.488	0.069	2.02E-202	2
DAPK1	1.66E-206	0.447815123	0.616	0.131	3.41E-202	2
TRPV2	1.89E-206	0.258504698	0.542	0.08	3.89E-202	2
PIK3AP1	7.30E-206	0.528098287	0.76	0.21	1.50E-201	2
SMIM25	8.86E-206	0.28463915	0.451	0.053	1.83E-201	2
SNX2	1.17E-205	0.613362807	0.849	0.279	2.42E-201	2
ACER3	3.57E-205	0.362641913	0.565	0.099	7.35E-201	2
FKBP15	6.51E-205	0.345473404	0.597	0.111	1.34E-200	2
AP1S2	7.87E-205	0.503386778	0.799	0.208	1.62E-200	2
FLI1	3.72E-204	0.281463606	0.574	0.09	7.66E-200	2
SLC2A5	7.97E-203	0.258625803	0.258	0.011	1.64E-198	2
NIPSNAP2	7.52E-202	0.351404407	0.646	0.135	1.55E-197	2
JDP2	8.92E-202	0.251453887	0.424	0.048	1.84E-197	2
DPP7	2.84E-201	0.503612147	0.872	0.231	5.85E-197	2
QKI	2.31E-200	0.665536039	0.909	0.358	4.76E-196	2
KLF4	4.04E-200	0.511008637	0.54	0.086	8.33E-196	2
CSTB	5.57E-200	1.278346294	0.985	0.6	1.15E-195	2
SCN1B	4.24E-199	0.269465772	0.342	0.03	8.73E-195	2
RNF13	5.52E-199	0.666539333	0.872	0.334	1.14E-194	2
ENPP2	8.66E-199	0.28315751	0.396	0.043	1.78E-194	2
LCP1	1.97E-197	0.814771373	0.988	0.357	4.07E-193	2
CD59	2.47E-197	0.546521242	0.849	0.287	5.08E-193	2
ETV5	4.62E-196	0.471102407	0.638	0.155	9.51E-192	2
SRGAP1	6.43E-196	0.250551669	0.324	0.025	1.32E-191	2
FTH1	6.82E-196	1.012172594	1	0.979	1.40E-191	2
CAPG	7.98E-196	0.761296018	0.752	0.211	1.64E-191	2
ST8SIA4	8.54E-196	0.315456081	0.574	0.095	1.76E-191	2
SNX24	1.60E-195	0.34022441	0.529	0.093	3.30E-191	2
CD300LF	6.83E-195	0.261420723	0.389	0.04	1.41E-190	2
LITAF	1.15E-194	0.847191972	0.966	0.461	2.37E-190	2
CCDC88A	3.83E-193	0.623506932	0.884	0.332	7.88E-189	2

LACC1	4.63E-193	0.328638512	0.47	0.071	9.53E-189	2
TIMP1	5.85E-193	0.518168511	0.894	0.248	1.20E-188	2
NAAA	6.49E-193	0.406588559	0.616	0.126	1.34E-188	2
ZSWIM6	2.84E-192	0.253432136	0.435	0.054	5.85E-188	2
SWAP70	1.19E-191	0.394564974	0.658	0.156	2.45E-187	2
P2RY6	2.09E-191	0.311273436	0.401	0.046	4.30E-187	2
MGLL	4.24E-191	0.681609148	0.703	0.21	8.73E-187	2
TENT2	6.88E-191	0.361574071	0.656	0.151	1.42E-186	2
RIN3	2.61E-190	0.289232869	0.562	0.093	5.38E-186	2
ITPRIPL2	7.42E-190	0.263531731	0.468	0.068	1.53E-185	2
CD9	1.77E-189	0.949313015	0.742	0.229	3.65E-185	2
IQGAP1	2.31E-189	0.597007955	0.923	0.283	4.76E-185	2
CPM	1.16E-188	0.748690021	0.784	0.265	2.38E-184	2
NINJ1	1.74E-188	0.689428521	0.898	0.353	3.59E-184	2
COLGALT1	4.14E-188	0.405605083	0.686	0.176	8.52E-184	2
SPP1	7.95E-188	2.89482294	0.638	0.169	1.64E-183	2
HPSE	5.43E-186	0.26414605	0.399	0.046	1.12E-181	2
PDGFC	5.34E-185	0.275651131	0.409	0.056	1.10E-180	2
BAIAP2	9.76E-185	0.280435227	0.383	0.046	2.01E-180	2
SNX18	1.53E-184	0.373615708	0.597	0.128	3.16E-180	2
SNN	3.97E-184	0.279176117	0.512	0.086	8.17E-180	2
SAMSN1	9.18E-184	0.350116726	0.822	0.193	1.89E-179	2
SGPP1	1.44E-183	0.309837656	0.465	0.075	2.96E-179	2
ZYX	3.48E-183	0.519265513	0.81	0.239	7.17E-179	2
DICER1	5.70E-183	0.424488019	0.747	0.217	1.17E-178	2
PLEKHB2	1.26E-182	0.506179737	0.832	0.27	2.60E-178	2
LPCAT2	2.29E-182	0.275869537	0.49	0.081	4.71E-178	2
VEGFB	3.45E-182	0.483889005	0.797	0.264	7.12E-178	2
SNX6	3.74E-182	0.693080611	0.933	0.43	7.71E-178	2
ATP6V1A	1.84E-181	0.496289637	0.768	0.253	3.78E-177	2
WWP1	2.22E-181	0.640890784	0.789	0.275	4.58E-177	2
PYCARD	2.23E-180	0.539299881	0.879	0.267	4.59E-176	2
HVCN1	3.07E-180	0.270183469	0.468	0.07	6.32E-176	2
OAZ2	5.61E-180	0.494103378	0.851	0.3	1.15E-175	2
ATP6AP1	9.77E-179	0.568423734	0.894	0.367	2.01E-174	2
H2AFY	2.45E-178	0.696969489	0.933	0.382	5.05E-174	2
GSTP1	3.00E-178	0.663305887	0.95	0.306	6.17E-174	2
MANBA	5.66E-178	0.289126385	0.513	0.094	1.16E-173	2
CLEC2B	8.60E-178	0.471152599	0.913	0.259	1.77E-173	2
PTPRJ	8.44E-177	0.345806949	0.643	0.154	1.74E-172	2
GNA13	1.84E-176	0.49558284	0.683	0.184	3.79E-172	2
ATP6AP2	3.44E-176	0.694947072	0.936	0.453	7.09E-172	2
VAMP8	7.43E-176	0.778889824	0.971	0.507	1.53E-171	2
GNPTAB	5.01E-175	0.349172059	0.718	0.192	1.03E-170	2
DEGS1	8.46E-175	0.371315657	0.74	0.199	1.74E-170	2

TMSB4X	9.45E-175	0.900540239	1	0.807	1.95E-170	2
C15orf48	1.05E-174	0.549702245	0.448	0.066	2.16E-170	2
BST2	3.57E-174	0.56949244	0.914	0.273	7.35E-170	2
DENND4C	6.98E-174	0.354940895	0.617	0.152	1.44E-169	2
GYPC	8.75E-174	0.588672808	0.797	0.215	1.80E-169	2
EGR1	1.04E-173	0.842376745	0.735	0.211	2.15E-169	2
NTAN1	2.16E-173	0.347885881	0.727	0.212	4.44E-169	2
LCP2	3.04E-173	0.432336262	0.805	0.199	6.26E-169	2
RHOG	4.26E-173	0.540012633	0.911	0.32	8.77E-169	2
APH1B	1.60E-172	0.291961265	0.495	0.091	3.30E-168	2
ANKH	6.86E-172	0.467076571	0.631	0.164	1.41E-167	2
RUNX1	4.21E-171	0.326478824	0.562	0.106	8.67E-167	2
HCLS1	4.63E-171	0.511018956	0.899	0.255	9.53E-167	2
ABHD12	1.02E-170	0.396687762	0.721	0.225	2.09E-166	2
GPRIN3	1.29E-169	0.279741372	0.564	0.104	2.66E-165	2
PRKACB	2.44E-169	0.330936799	0.622	0.148	5.03E-165	2
MYO1F	3.97E-169	0.29069942	0.738	0.171	8.19E-165	2
LGALS1	1.28E-168	0.90954678	0.983	0.55	2.64E-164	2
EVI2A	3.33E-168	0.333854974	0.633	0.132	6.85E-164	2
ASAP1	4.20E-168	0.412541329	0.696	0.195	8.65E-164	2
SLC18B1	5.65E-168	0.348849525	0.45	0.08	1.16E-163	2
SCAMP2	1.04E-167	0.473532642	0.824	0.291	2.15E-163	2
SLC43A3	1.53E-167	0.366255136	0.582	0.13	3.15E-163	2
SEL1L3	2.51E-167	0.268380477	0.409	0.056	5.16E-163	2
VAT1	1.13E-166	0.471286541	0.695	0.217	2.33E-162	2
ME2	1.18E-166	0.380441088	0.629	0.157	2.44E-162	2
LPAR6	7.55E-166	0.342996311	0.643	0.149	1.55E-161	2
SRGN	9.61E-166	0.878429057	0.995	0.483	1.98E-161	2
GLMP	1.89E-165	0.365389422	0.622	0.17	3.89E-161	2
NABP1	2.93E-165	0.454123196	0.814	0.244	6.03E-161	2
FAM20A	6.43E-165	0.354908798	0.48	0.095	1.32E-160	2
C4orf48	1.66E-164	0.314139443	0.713	0.165	3.43E-160	2
CLN8	2.81E-164	0.367862721	0.616	0.159	5.80E-160	2
P2RY13	6.25E-164	0.281965989	0.376	0.047	1.29E-159	2
CAPZB	9.47E-164	0.738295664	0.987	0.577	1.95E-159	2
SKAP2	1.16E-163	0.481307054	0.79	0.27	2.38E-159	2
LAMP2	2.38E-163	0.551528593	0.93	0.408	4.89E-159	2
SMS	3.03E-163	0.47986021	0.841	0.315	6.25E-159	2
ICAM1	5.82E-163	0.553492705	0.755	0.235	1.20E-158	2
PRCP	1.44E-161	0.458431397	0.73	0.246	2.96E-157	2
PLXNC1	1.45E-161	0.304961001	0.488	0.094	2.99E-157	2
UCP2	1.48E-161	0.575026067	0.914	0.283	3.06E-157	2
GPR155	1.66E-161	0.399289255	0.591	0.144	3.43E-157	2
HCST	1.93E-161	0.7887827	0.953	0.36	3.98E-157	2
IL13RA1	3.13E-161	0.346025421	0.597	0.153	6.45E-157	2

SYNGR2	6.09E-161	0.564703265	0.901	0.397	1.25E-156	2
LAT2	1.05E-160	0.252914513	0.493	0.085	2.15E-156	2
GGA2	1.43E-160	0.319183624	0.639	0.168	2.94E-156	2
ARPIN	1.62E-160	0.258696472	0.451	0.081	3.35E-156	2
ITSN1	1.81E-160	0.371364584	0.554	0.137	3.73E-156	2
TACC1	2.19E-160	0.445527545	0.847	0.271	4.50E-156	2
ENTPD1	1.86E-159	0.299029369	0.666	0.15	3.83E-155	2
INF2	2.79E-159	0.321300318	0.508	0.103	5.74E-155	2
FYB1	3.42E-159	0.595504876	0.95	0.312	7.04E-155	2
ZFYVE16	5.61E-159	0.389785367	0.634	0.174	1.16E-154	2
PTPN6	1.10E-158	0.354173842	0.815	0.227	2.26E-154	2
CD53	1.22E-158	0.606765111	0.968	0.343	2.51E-154	2
GIMAP4	1.75E-158	0.441140337	0.832	0.228	3.61E-154	2
GRINA	1.83E-158	0.469518086	0.782	0.289	3.76E-154	2
ARHGAP4	5.78E-158	0.394156774	0.713	0.193	1.19E-153	2
TMEM106A	8.00E-158	0.322353134	0.535	0.121	1.65E-153	2
CXCL3	1.28E-157	0.770523509	0.322	0.037	2.64E-153	2
ATG7	3.10E-157	0.267781139	0.497	0.102	6.37E-153	2
BNIP3L	5.06E-157	0.585130344	0.866	0.363	1.04E-152	2
TYMP	2.11E-155	0.736261303	0.953	0.446	4.34E-151	2
PLXND1	3.12E-155	0.352882539	0.617	0.167	6.43E-151	2
OSTF1	6.99E-155	0.431594337	0.851	0.303	1.44E-150	2
TNFRSF1B	1.31E-154	0.326663299	0.871	0.242	2.70E-150	2
CCL3	5.46E-154	0.682207006	0.634	0.156	1.12E-149	2
SNX8	9.49E-154	0.290052224	0.534	0.122	1.96E-149	2
NAGA	1.15E-153	0.360087371	0.631	0.176	2.36E-149	2
EVA1B	2.63E-153	0.336105779	0.644	0.179	5.42E-149	2
WASHC4	3.82E-153	0.406546596	0.773	0.273	7.86E-149	2
PHACTR2	4.19E-153	0.332473721	0.691	0.189	8.64E-149	2
SGK3	1.02E-152	0.354566417	0.594	0.159	2.10E-148	2
CNDP2	1.46E-152	0.49050837	0.817	0.328	3.00E-148	2
ZFHX3	3.84E-152	0.444858368	0.718	0.239	7.91E-148	2
MBP	4.33E-152	0.251810893	0.639	0.142	8.93E-148	2
MT1G	9.04E-152	1.615134137	0.456	0.088	1.86E-147	2
NCF1	1.51E-151	0.291995551	0.569	0.12	3.10E-147	2
VSIR	1.70E-151	0.357114583	0.727	0.181	3.51E-147	2
PREX1	2.60E-151	0.251265155	0.659	0.152	5.35E-147	2
SLC29A3	5.82E-151	0.252245696	0.401	0.067	1.20E-146	2
CALM3	1.28E-150	0.773603541	0.933	0.47	2.64E-146	2
CCL3L1	1.93E-150	0.746072689	0.664	0.176	3.97E-146	2
TNFRSF21	8.36E-150	0.295997344	0.391	0.064	1.72E-145	2
ARAP1	1.03E-149	0.331012691	0.599	0.16	2.13E-145	2
ATP6V0A1	1.69E-149	0.391399272	0.658	0.202	3.48E-145	2
PLBD2	4.89E-149	0.262362748	0.471	0.097	1.01E-144	2
RARRES1	1.76E-148	0.401334804	0.391	0.067	3.63E-144	2

VOPP1	2.06E-148	0.414173013	0.784	0.271	4.25E-144	2
HIF1A	2.51E-148	0.394447457	0.755	0.253	5.16E-144	2
TPM4	2.72E-148	0.52910225	0.883	0.319	5.59E-144	2
ZDHHC7	7.26E-148	0.257586466	0.497	0.105	1.50E-143	2
CEP170	1.89E-147	0.338252748	0.592	0.16	3.89E-143	2
PI4K2A	4.77E-147	0.250659161	0.44	0.083	9.83E-143	2
IL10	2.10E-146	0.325025256	0.272	0.025	4.32E-142	2
PAK1	2.54E-146	0.263901263	0.544	0.124	5.23E-142	2
KCNK6	4.46E-146	0.258572639	0.463	0.082	9.19E-142	2
SNX10	4.48E-145	0.431939937	0.728	0.235	9.22E-141	2
IFI30	1.50E-144	0.352147202	0.477	0.094	3.09E-140	2
SEC14L1	3.68E-144	0.287826252	0.705	0.213	7.57E-140	2
ANXA1	4.77E-144	0.325822269	0.901	0.271	9.82E-140	2
RRAGD	7.45E-144	0.291548089	0.507	0.122	1.53E-139	2
LAP3	1.33E-143	0.498822893	0.789	0.299	2.74E-139	2
HEXB	1.48E-143	0.468817203	0.826	0.333	3.06E-139	2
LYZ	2.40E-143	0.681558411	0.985	0.575	4.94E-139	2
RNPEP	3.38E-143	0.333162322	0.668	0.204	6.95E-139	2
ADPGK	4.60E-143	0.381759368	0.716	0.231	9.47E-139	2
ANXA2	1.33E-141	0.758395342	0.973	0.589	2.74E-137	2
SERINC1	1.79E-141	0.462582209	0.862	0.366	3.69E-137	2
STX12	4.42E-141	0.340175685	0.671	0.217	9.11E-137	2
SMPDL3A	2.15E-140	0.386612447	0.634	0.2	4.42E-136	2
CASP1	2.34E-140	0.3180801	0.758	0.203	4.83E-136	2
SFT2D1	3.63E-140	0.336490825	0.762	0.245	7.48E-136	2
AKR1A1	3.44E-139	0.568836175	0.874	0.411	7.08E-135	2
ACTR2	3.59E-139	0.599651928	0.96	0.527	7.39E-135	2
SLC25A24	1.02E-138	0.283164083	0.547	0.141	2.10E-134	2
CORO1C	1.28E-138	0.339076516	0.621	0.19	2.64E-134	2
CMTM3	1.49E-138	0.369999371	0.752	0.25	3.06E-134	2
FNBP1	3.45E-138	0.406855804	0.842	0.31	7.11E-134	2
CNPY3	4.76E-138	0.400911226	0.827	0.313	9.81E-134	2
GNAQ	3.28E-137	0.400856539	0.68	0.227	6.76E-133	2
ARPC5	4.24E-137	0.59482688	0.97	0.569	8.73E-133	2
CYBA	1.00E-136	0.762062048	0.997	0.489	2.07E-132	2
RAC1	1.10E-136	0.607683919	0.987	0.66	2.27E-132	2
RTN3	1.38E-136	0.351407816	0.78	0.3	2.83E-132	2
NECAP2	1.73E-136	0.370279114	0.8	0.284	3.57E-132	2
PKM	4.49E-136	0.447564228	0.936	0.321	9.24E-132	2
PAPSS1	6.17E-136	0.330863144	0.594	0.173	1.27E-131	2
GRB2	7.64E-136	0.488299724	0.903	0.404	1.57E-131	2
SESN1	1.83E-135	0.29241277	0.471	0.106	3.77E-131	2
OAZ1	1.96E-135	0.631620159	0.998	0.782	4.04E-131	2
OSBPL11	2.49E-135	0.280090815	0.515	0.127	5.12E-131	2
RTN4	1.27E-134	0.609673759	0.978	0.584	2.62E-130	2

ITGB5	9.72E-134	0.390548733	0.663	0.23	2.00E-129	2
WIPF1	1.08E-133	0.294351448	0.809	0.245	2.22E-129	2
PTBP3	1.33E-133	0.388716498	0.77	0.29	2.73E-129	2
BNIP2	4.30E-132	0.297644468	0.725	0.249	8.85E-128	2
MMP12	1.63E-131	0.961027183	0.279	0.032	3.36E-127	2
ZMIZ1	2.65E-131	0.283598618	0.532	0.135	5.46E-127	2
TCIRG1	5.95E-131	0.34534696	0.715	0.233	1.23E-126	2
CD164	1.12E-130	0.517742644	0.94	0.501	2.30E-126	2
RAB10	1.27E-130	0.433786437	0.861	0.383	2.62E-126	2
RBPJ	2.24E-130	0.410103981	0.867	0.354	4.61E-126	2
PICALM	2.68E-130	0.259170528	0.711	0.238	5.53E-126	2
MAP3K3	9.60E-130	0.274689486	0.529	0.134	1.98E-125	2
TNFAIP8	1.85E-129	0.266134262	0.732	0.203	3.80E-125	2
UBE2D1	1.33E-128	0.30131785	0.683	0.224	2.75E-124	2
CXCL2	1.60E-128	0.677812153	0.44	0.095	3.29E-124	2
MSN	1.99E-128	0.451914007	0.921	0.359	4.10E-124	2
ZFP36L1	5.69E-128	0.727942505	0.977	0.618	1.17E-123	2
IQGAP2	9.49E-128	0.570604848	0.909	0.422	1.95E-123	2
ATP6V0B	9.88E-128	0.543662151	0.963	0.528	2.04E-123	2
FAM49B	3.07E-127	0.417180579	0.896	0.371	6.32E-123	2
OAS1	3.95E-127	0.258193207	0.512	0.126	8.13E-123	2
PTTG1IP	4.32E-127	0.407345017	0.839	0.36	8.90E-123	2
IER5	5.11E-127	0.605738512	0.928	0.424	1.05E-122	2
CEBPD	1.16E-126	0.602432077	0.945	0.476	2.40E-122	2
SDCCAG8	2.82E-126	0.289914571	0.532	0.145	5.82E-122	2
FCGR2B	3.30E-126	0.565223124	0.314	0.045	6.80E-122	2
CACUL1	1.19E-125	0.309530165	0.658	0.221	2.44E-121	2
VAMP3	1.45E-125	0.258897804	0.56	0.16	2.98E-121	2
NR1H3	3.75E-125	0.434521063	0.626	0.218	7.72E-121	2
HK2	2.45E-124	0.313752231	0.255	0.028	5.06E-120	2
PEPD	7.24E-124	0.340098861	0.747	0.286	1.49E-119	2
AP2S1	2.06E-123	0.522818319	0.941	0.501	4.25E-119	2
ATP6V1D	1.72E-122	0.29260903	0.681	0.238	3.55E-118	2
SFT2D2	4.19E-122	0.306184699	0.69	0.247	8.64E-118	2
PBDC1	4.43E-122	0.253365124	0.628	0.191	9.12E-118	2
CTNNA1	4.84E-122	0.32268306	0.775	0.31	9.97E-118	2
IER3	1.88E-121	0.675854228	0.849	0.397	3.88E-117	2
THBD	3.82E-121	0.308125955	0.309	0.044	7.86E-117	2
TENT5A	4.25E-121	0.58654792	0.676	0.242	8.75E-117	2
P2RX7	7.07E-121	0.282576729	0.458	0.105	1.46E-116	2
DOK2	1.26E-120	0.303854893	0.866	0.267	2.60E-116	2
ANXA11	2.53E-120	0.408158771	0.867	0.391	5.22E-116	2
FNIP2	3.13E-120	0.421755871	0.674	0.251	6.45E-116	2
ABI1	1.03E-119	0.309074193	0.688	0.242	2.12E-115	2
TRIM14	1.79E-119	0.263486147	0.517	0.142	3.68E-115	2

RNH1	2.17E-119	0.43624502	0.871	0.408	4.46E-115	2
DBNL	3.89E-119	0.324485769	0.797	0.309	8.02E-115	2
CARD16	1.43E-118	0.276974399	0.857	0.275	2.95E-114	2
GNAI2	1.88E-118	0.562908866	0.99	0.675	3.87E-114	2
WSB1	2.25E-118	0.58947081	0.948	0.501	4.63E-114	2
SNAP23	2.64E-118	0.325919808	0.728	0.277	5.44E-114	2
ATP6V0D1	4.09E-118	0.410966665	0.789	0.334	8.42E-114	2
CTDNEP1	4.86E-118	0.306747498	0.809	0.318	1.00E-113	2
NUP214	5.37E-118	0.258489684	0.572	0.172	1.11E-113	2
KIAA0930	5.88E-118	0.326372039	0.686	0.258	1.21E-113	2
EFHD2	9.83E-118	0.370091189	0.893	0.358	2.03E-113	2
SNX5	2.02E-117	0.431740953	0.866	0.404	4.16E-113	2
GNAS	2.58E-117	0.537198251	0.992	0.738	5.31E-113	2
PLEKHM2	3.14E-117	0.250702908	0.523	0.147	6.46E-113	2
SOAT1	4.90E-117	0.28350293	0.527	0.15	1.01E-112	2
FAM20C	6.18E-117	0.386070408	0.523	0.16	1.27E-112	2
CLTC	2.68E-116	0.455027182	0.893	0.404	5.52E-112	2
TMEM37	3.69E-116	0.329582078	0.628	0.218	7.60E-112	2
RAB5C	7.38E-115	0.434836884	0.899	0.468	1.52E-110	2
RHOB	1.14E-114	0.764941705	0.946	0.557	2.35E-110	2
SH3BGRL3	1.17E-114	0.680750659	0.997	0.726	2.41E-110	2
TSPO	1.33E-114	0.402623111	0.867	0.349	2.73E-110	2
TGFBR1	1.52E-114	0.257342477	0.508	0.14	3.12E-110	2
ADIPOR1	3.16E-114	0.324177787	0.747	0.309	6.52E-110	2
GABARAP	3.58E-114	0.293904108	0.715	0.266	7.37E-110	2
TMEM70	3.92E-114	0.322319417	0.641	0.225	8.07E-110	2
SQOR	4.40E-114	0.32313041	0.721	0.273	9.06E-110	2
PARP14	7.25E-114	0.38424731	0.743	0.291	1.49E-109	2
COTL1	1.63E-113	0.370101226	0.961	0.355	3.36E-109	2
TAOK1	2.15E-113	0.268265474	0.664	0.234	4.44E-109	2
RRAGC	2.92E-113	0.258333491	0.565	0.176	6.01E-109	2
NAMPT	4.04E-113	0.377504379	0.911	0.461	8.32E-109	2
LACTB	6.28E-113	0.311478349	0.708	0.278	1.29E-108	2
MT1F	1.31E-112	0.602339033	0.403	0.087	2.69E-108	2
FXYD5	2.01E-112	0.3096867	0.903	0.3	4.14E-108	2
PLXNB2	2.14E-112	0.325657192	0.644	0.235	4.40E-108	2
CAP1	5.44E-112	0.522224872	0.977	0.62	1.12E-107	2
RGS2	5.54E-112	0.398385828	0.844	0.307	1.14E-107	2
CDKN1A	7.81E-112	0.324248658	0.572	0.153	1.61E-107	2
CXCL9	2.13E-111	1.06841339	0.277	0.04	4.38E-107	2
SLC2A8	3.23E-111	0.262172853	0.46	0.124	6.65E-107	2
ACTB	4.56E-111	0.661977485	1	0.94	9.40E-107	2
ITM2B	7.20E-111	0.588893746	0.997	0.861	1.48E-106	2
USF2	1.40E-110	0.344005236	0.745	0.308	2.89E-106	2
IFNAR1	3.58E-110	0.305506815	0.737	0.295	7.37E-106	2

GPSM3	1.12E-109	0.261736354	0.861	0.275	2.31E-105	2
ATOX1	1.22E-109	0.504381603	0.903	0.481	2.51E-105	2
CD44	6.77E-109	0.328980979	0.799	0.264	1.40E-104	2
STAT1	1.27E-108	0.537441935	0.82	0.378	2.62E-104	2
CAPNS1	1.96E-108	0.35659879	0.888	0.428	4.04E-104	2
ARPC3	2.48E-108	0.532352432	0.992	0.73	5.10E-104	2
FAM91A1	1.03E-107	0.282731266	0.55	0.179	2.12E-103	2
GADD45B	1.11E-107	0.500376094	0.906	0.439	2.30E-103	2
CD40	2.11E-107	0.343082658	0.57	0.189	4.34E-103	2
MOB1A	5.34E-107	0.399186789	0.881	0.427	1.10E-102	2
SLC35F6	1.18E-106	0.259706492	0.525	0.164	2.43E-102	2
ATP6V0E1	1.37E-106	0.489638916	0.965	0.613	2.81E-102	2
DDX3Y	1.39E-106	0.30729392	0.648	0.189	2.87E-102	2
NFKBID	5.34E-106	0.251322436	0.495	0.12	1.10E-101	2
P2RX4	7.06E-106	0.300241349	0.539	0.174	1.45E-101	2
GLA	8.26E-106	0.321885416	0.544	0.168	1.70E-101	2
LIMS1	8.87E-106	0.483532319	0.881	0.448	1.83E-101	2
ARL6IP5	1.69E-105	0.436728439	0.943	0.506	3.48E-101	2
IFI16	3.33E-105	0.296557352	0.852	0.286	6.86E-101	2
EHBP1L1	1.01E-104	0.264311835	0.664	0.235	2.09E-100	2
PEA15	2.73E-104	0.271221308	0.69	0.261	5.62E-100	2
CAPZA2	3.43E-104	0.347183904	0.884	0.432	7.06E-100	2
FOXO3	4.66E-104	0.381557421	0.661	0.26	9.61E-100	2
NR4A2	7.19E-104	0.603371255	0.698	0.249	1.48E-99	2
MMD	1.56E-103	0.33473952	0.552	0.189	3.22E-99	2
YWHAH	1.76E-103	0.363508586	0.826	0.369	3.62E-99	2
AVPI1	1.76E-103	0.31863338	0.448	0.125	3.63E-99	2
ATP6V1C1	2.45E-103	0.295018708	0.576	0.205	5.05E-99	2
YBX1	2.49E-103	0.474676376	0.988	0.772	5.12E-99	2
CTNND1	5.53E-103	0.271115425	0.564	0.194	1.14E-98	2
OSBPL8	2.12E-102	0.250514995	0.695	0.268	4.37E-98	2
BCKDK	2.83E-102	0.283995563	0.609	0.226	5.83E-98	2
CPEB4	6.87E-102	0.277296997	0.622	0.224	1.41E-97	2
SMIM30	5.34E-101	0.266317951	0.703	0.283	1.10E-96	2
CCL4L2	5.40E-101	0.582278751	0.628	0.211	1.11E-96	2
CALM2	6.60E-101	0.507152091	0.987	0.74	1.36E-96	2
ZNF106	8.42E-101	0.275949238	0.688	0.272	1.73E-96	2
VMA21	1.81E-100	0.271560838	0.68	0.266	3.72E-96	2
TUBA1B	5.65E-100	0.498290793	0.985	0.649	1.16E-95	2
EFR3A	8.13E-100	0.250576504	0.572	0.2	1.67E-95	2
ATP1B3	8.96E-100	0.330793986	0.807	0.325	1.84E-95	2
SEPT9	4.02E-99	0.311855287	0.831	0.355	8.28E-95	2
CD47	9.34E-99	0.281324384	0.773	0.328	1.92E-94	2
CAPZA1	2.06E-98	0.354767029	0.884	0.442	4.24E-94	2
IL1B	4.39E-98	0.394319866	0.378	0.083	9.03E-94	2

NXF1	9.68E-98	0.260992771	0.517	0.153	1.99E-93	2
RHOA	1.09E-97	0.479607064	0.978	0.686	2.25E-93	2
H2AFJ	3.76E-97	0.319901203	0.779	0.346	7.75E-93	2
NDFIP1	4.34E-97	0.309989817	0.839	0.398	8.93E-93	2
APOC1	6.14E-97	0.837735592	0.985	0.767	1.26E-92	2
ARL8B	7.18E-97	0.268821687	0.69	0.286	1.48E-92	2
DHRS3	1.15E-96	0.293463611	0.725	0.299	2.36E-92	2
AHNAK	5.30E-96	0.497573528	0.881	0.476	1.09E-91	2
ATP6V1F	1.11E-95	0.431533168	0.918	0.533	2.28E-91	2
SLC3A2	2.08E-95	0.266259825	0.78	0.328	4.28E-91	2
ZFAND5	2.27E-95	0.440688302	0.841	0.412	4.67E-91	2
ZFP36	4.28E-95	0.310619358	0.82	0.348	8.81E-91	2
BAG3	4.49E-95	0.58309912	0.673	0.262	9.25E-91	2
NCOA4	5.38E-94	0.410190994	0.888	0.43	1.11E-89	2
VCAM1	6.47E-94	0.779425783	0.582	0.227	1.33E-89	2
DNAJA4	1.39E-93	0.27685159	0.592	0.181	2.86E-89	2
PLIN2	1.64E-93	0.713716004	0.831	0.422	3.37E-89	2
MGST3	2.02E-93	0.402398857	0.924	0.515	4.16E-89	2
TMEM147	4.58E-93	0.274339369	0.711	0.306	9.43E-89	2
PDK4	4.73E-93	0.517885687	0.601	0.233	9.75E-89	2
HLA-E	5.67E-93	0.492619592	0.995	0.713	1.17E-88	2
NENF	6.81E-93	0.301768407	0.785	0.36	1.40E-88	2
C6orf62	1.02E-92	0.34441769	0.849	0.409	2.10E-88	2
RAP1A	1.31E-92	0.275878855	0.903	0.452	2.69E-88	2
WDR1	2.37E-92	0.28051092	0.826	0.366	4.89E-88	2
TMBIM6	2.95E-92	0.42705259	0.993	0.734	6.08E-88	2
PRDX1	4.87E-92	0.603164573	0.985	0.741	1.00E-87	2
SOD2	1.27E-91	0.578330378	0.948	0.525	2.62E-87	2
ERP44	1.31E-91	0.296136168	0.784	0.363	2.70E-87	2
GDI2	1.45E-91	0.334813819	0.931	0.494	2.98E-87	2
OPN3	1.62E-91	0.2782144	0.485	0.16	3.33E-87	2
GMFG	3.16E-91	0.355321484	0.933	0.38	6.51E-87	2
ATG3	6.06E-91	0.265918274	0.743	0.322	1.25E-86	2
CTSA	6.65E-91	0.421920181	0.909	0.476	1.37E-86	2
CRTAP	9.45E-91	0.266637296	0.7	0.297	1.95E-86	2
STX4	2.21E-90	0.316436046	0.634	0.258	4.56E-86	2
FABP4	3.35E-90	0.606727999	0.284	0.051	6.90E-86	2
APPL1	6.79E-90	0.265671304	0.674	0.283	1.40E-85	2
HES1	3.38E-89	0.743692581	0.616	0.269	6.96E-85	2
OS9	4.83E-89	0.295445982	0.82	0.398	9.94E-85	2
VASP	8.90E-89	0.255000284	0.768	0.306	1.83E-84	2
ARPC4	1.23E-88	0.34435126	0.919	0.489	2.54E-84	2
IL1R2	2.02E-88	0.259186673	0.361	0.087	4.16E-84	2
APLP2	4.23E-88	0.434349103	0.975	0.531	8.72E-84	2
TLN1	4.78E-87	0.302709239	0.772	0.359	9.85E-83	2

RAB7A	5.46E-87	0.353771443	0.923	0.505	1.12E-82	2
VPS35	8.74E-87	0.281927383	0.74	0.331	1.80E-82	2
ATP2B1	1.25E-86	0.307721019	0.768	0.36	2.58E-82	2
GNAI3	1.69E-86	0.266036564	0.777	0.359	3.49E-82	2
WASF2	1.76E-86	0.346111106	0.824	0.405	3.63E-82	2
MCUR1	2.87E-86	0.286706291	0.673	0.291	5.91E-82	2
CEBPA	6.89E-86	0.2907074	0.691	0.286	1.42E-81	2
ARPC1B	1.08E-85	0.445246027	0.948	0.592	2.22E-81	2
NFIC	1.90E-85	0.253413237	0.664	0.278	3.92E-81	2
APOE	2.89E-85	0.69284016	0.998	0.914	5.96E-81	2
TAOK3	3.38E-85	0.282111018	0.777	0.359	6.97E-81	2
CYB5R1	1.82E-84	0.260972014	0.569	0.228	3.75E-80	2
TXNIP	1.89E-84	0.442099635	0.96	0.606	3.89E-80	2
CISD2	2.41E-84	0.275521805	0.659	0.289	4.96E-80	2
TNS3	9.93E-84	0.295066854	0.53	0.204	2.04E-79	2
CAST	3.71E-83	0.282291298	0.846	0.427	7.64E-79	2
NUCB1	6.04E-83	0.264332108	0.765	0.362	1.24E-78	2
ACTR3	2.79E-82	0.358855724	0.918	0.515	5.75E-78	2
ARHGDIB	3.19E-82	0.462447095	0.982	0.46	6.57E-78	2
CAB39	4.06E-82	0.252411869	0.663	0.289	8.37E-78	2
TMSB10	5.86E-82	0.474935798	0.995	0.891	1.21E-77	2
TMED10	6.05E-82	0.311264606	0.856	0.452	1.25E-77	2
ATF3	9.95E-82	0.456679748	0.664	0.271	2.05E-77	2
GLRX	1.24E-81	0.279591198	0.78	0.37	2.55E-77	2
PTPRC	1.52E-81	0.282192075	0.958	0.383	3.14E-77	2
MYL6	2.54E-81	0.464965041	0.992	0.842	5.23E-77	2
GNB1	2.55E-81	0.307139193	0.881	0.483	5.25E-77	2
PDXK	1.39E-80	0.271945104	0.676	0.303	2.87E-76	2
ARL6IP1	1.03E-79	0.319926246	0.93	0.517	2.12E-75	2
ARPC2	1.37E-79	0.441927741	0.99	0.735	2.83E-75	2
ACTN1	1.55E-79	0.255407254	0.676	0.3	3.20E-75	2
TMEM50A	4.56E-79	0.26966206	0.891	0.476	9.39E-75	2
TFRC	4.70E-79	0.527005242	0.562	0.227	9.69E-75	2
HERPUD1	5.66E-79	0.400196383	0.926	0.559	1.17E-74	2
ACSL1	7.15E-79	0.277518154	0.604	0.25	1.47E-74	2
UBE2D3	8.79E-79	0.361797739	0.941	0.589	1.81E-74	2
TNFRSF14	2.09E-78	0.272528706	0.779	0.385	4.31E-74	2
NFE2L2	8.87E-78	0.302944093	0.834	0.419	1.83E-73	2
TRAM1	7.74E-77	0.251232708	0.817	0.404	1.59E-72	2
DDAH2	2.59E-76	0.251003697	0.794	0.381	5.33E-72	2
HSPH1	4.01E-76	0.343361074	0.889	0.464	8.26E-72	2
POMP	1.86E-75	0.368776142	0.956	0.618	3.83E-71	2
LAMTOR2	3.74E-75	0.274463248	0.812	0.417	7.69E-71	2
CCNI	3.11E-74	0.363015101	0.945	0.585	6.41E-70	2
MT1E	3.21E-74	0.801316885	0.453	0.153	6.61E-70	2

LAMTOR1	1.22E-73	0.275204741	0.859	0.461	2.51E-69	2
DAZAP2	4.91E-73	0.281270448	0.899	0.494	1.01E-68	2
REL	6.10E-72	0.289713387	0.787	0.385	1.26E-67	2
MCL1	4.50E-71	0.366052371	0.936	0.583	9.26E-67	2
IFI6	7.58E-71	0.259105926	0.622	0.26	1.56E-66	2
P4HA1	7.90E-71	0.279559498	0.648	0.282	1.63E-66	2
RBMS1	5.61E-70	0.259201062	0.824	0.406	1.16E-65	2
CDC42	4.45E-68	0.27144352	0.946	0.577	9.16E-64	2
HLA-B	1.54E-67	0.486866703	1	0.628	3.18E-63	2
PABPC4	1.55E-67	0.273383756	0.789	0.401	3.20E-63	2
FOSB	4.33E-67	0.302325478	0.846	0.39	8.91E-63	2
PPP1CB	1.14E-66	0.256429409	0.794	0.415	2.36E-62	2
PGK1	1.96E-66	0.364038813	0.926	0.579	4.03E-62	2
ZNF331	2.85E-66	0.347235355	0.711	0.363	5.86E-62	2
B2M	2.30E-65	0.48989934	1	0.941	4.73E-61	2
GBP1	6.89E-65	0.445008392	0.611	0.27	1.42E-60	2
CLTA	1.46E-64	0.332996345	0.961	0.598	3.00E-60	2
SLC25A6	1.64E-64	0.293919738	0.933	0.596	3.37E-60	2
ADM	2.24E-64	0.25096976	0.441	0.154	4.62E-60	2
NFKBIA	1.42E-62	0.446146591	0.898	0.588	2.93E-58	2
MTSS1	7.14E-62	0.262548629	0.653	0.312	1.47E-57	2
CCL4	1.85E-61	0.292541169	0.525	0.204	3.80E-57	2
KLF6	8.27E-60	0.395807227	0.943	0.618	1.70E-55	2
TMBIM4	1.74E-59	0.258914041	0.883	0.513	3.58E-55	2
TPM3	2.02E-59	0.312850559	0.958	0.646	4.16E-55	2
NFKBIZ	4.71E-59	0.264205262	0.678	0.336	9.71E-55	2
BCAP31	2.31E-57	0.272443596	0.926	0.531	4.77E-53	2
CEBPB	5.52E-57	0.308473776	0.899	0.525	1.14E-52	2
MT1X	3.77E-53	1.1288931	0.612	0.321	7.76E-49	2
YWHAB	1.77E-51	0.271956762	0.971	0.683	3.64E-47	2
MT2A	3.20E-49	1.20278836	0.836	0.561	6.58E-45	2
VAMP5	7.99E-49	0.266669665	0.742	0.424	1.64E-44	2
ABL2	1.16E-47	0.252144354	0.371	0.15	2.38E-43	2
BRI3	3.75E-47	0.263768294	0.978	0.656	7.72E-43	2
CHMP1B	6.29E-47	0.337635285	0.658	0.369	1.30E-42	2
CFL1	8.18E-44	0.284380276	0.987	0.827	1.68E-39	2
ACTG1	1.02E-43	0.255175645	0.997	0.904	2.10E-39	2
PCBP1	4.35E-43	0.257265674	0.956	0.645	8.96E-39	2
RCAN1	8.47E-40	0.26345403	0.334	0.139	1.74E-35	2
HNRNPU	5.10E-32	0.263999405	0.926	0.643	1.05E-27	2
IL1RN	5.49E-13	0.40863488	0.327	0.221	1.13E-08	2
FCN1	0	2.044328949	0.669	0.02	0	3
LGALS2	0	1.553720976	0.806	0.085	0	3
CLEC10A	0	1.450704231	0.618	0.063	0	3
LST1	0	1.435313629	0.945	0.19	0	3

MNDA	0	1.287169299	0.81	0.096	0	3
FGL2	0	1.249788626	0.908	0.181	0	3
SPI1	0	1.134162114	0.879	0.152	0	3
CSTA	0	0.912359453	0.671	0.09	0	3
HCK	0	0.882152687	0.761	0.118	0	3
CD300E	0	0.644379299	0.333	0.007	0	3
AIF1	3.58E-307	1.533248965	0.984	0.248	7.38E-303	3
CYBB	3.88E-307	1.060792845	0.838	0.141	7.99E-303	3
S100A8	1.20E-305	2.045570495	0.411	0.02	2.47E-301	3
IL1B	2.82E-303	2.020339339	0.601	0.066	5.81E-299	3
C1orf162	3.23E-292	1.106475992	0.875	0.182	6.64E-288	3
VCAN	2.11E-291	1.282770749	0.378	0.017	4.34E-287	3
CD1D	3.10E-287	0.561095823	0.444	0.03	6.39E-283	3
BCL2A1	4.48E-283	1.473818316	0.712	0.116	9.23E-279	3
NCF2	5.98E-281	0.883624303	0.751	0.132	1.23E-276	3
CFP	1.26E-280	0.754229211	0.495	0.043	2.59E-276	3
COTL1	1.39E-278	1.51822077	0.992	0.362	2.87E-274	3
CPVL	1.74E-276	1.217874053	0.892	0.207	3.58E-272	3
FCER1G	1.03E-264	1.370345914	0.986	0.274	2.12E-260	3
LILRB2	1.66E-264	0.874239164	0.669	0.107	3.42E-260	3
TYROBP	9.81E-263	1.326775937	0.99	0.267	2.02E-258	3
MPEG1	3.13E-260	0.957850822	0.795	0.146	6.45E-256	3
HLA-DRB1	1.90E-259	1.552259555	0.939	0.283	3.91E-255	3
FGR	7.05E-259	0.767727329	0.603	0.081	1.45E-254	3
CTSS	1.00E-254	1.447698462	0.977	0.318	2.07E-250	3
LYZ	7.96E-252	2.216159675	0.982	0.582	1.64E-247	3
IGSF6	7.74E-245	0.852066093	0.826	0.157	1.60E-240	3
CLEC12A	8.59E-245	0.557455068	0.413	0.032	1.77E-240	3
TNFSF13B	5.24E-243	0.922912712	0.744	0.145	1.08E-238	3
CST3	3.63E-242	1.637229628	0.998	0.588	7.48E-238	3
S100A9	6.84E-240	2.471820679	0.683	0.138	1.41E-235	3
FPR1	9.80E-234	0.854575205	0.487	0.057	2.02E-229	3
PTPRE	2.18E-232	0.882942006	0.769	0.166	4.50E-228	3
S100A11	7.71E-230	1.327672623	1	0.448	1.59E-225	3
LILRB3	4.28E-226	0.654319561	0.603	0.098	8.82E-222	3
AOAH	5.25E-226	0.695747837	0.656	0.114	1.08E-221	3
GSTP1	6.15E-222	1.118240083	0.945	0.317	1.27E-217	3
HLA-DRA	1.84E-221	1.622128793	0.994	0.48	3.80E-217	3
HLA-DMB	3.41E-221	0.953182658	0.82	0.18	7.03E-217	3
LYN	7.53E-221	0.870176498	0.802	0.198	1.55E-216	3
CALHM6	4.26E-220	0.755450847	0.589	0.094	8.76E-216	3
JAML	2.05E-219	1.125233519	0.736	0.189	4.22E-215	3
MS4A6A	8.65E-219	1.035854701	0.834	0.187	1.78E-214	3
KLF4	4.46E-217	1.096588726	0.568	0.09	9.18E-213	3
CD1C	3.33E-215	0.99720478	0.341	0.024	6.86E-211	3

TYMP	5.80E-215	1.419380854	0.953	0.454	1.19E-210	3
HLA-DPA1	2.69E-212	1.511047687	0.967	0.409	5.54E-208	3
FCGR2A	3.28E-211	0.805930755	0.761	0.155	6.76E-207	3
HLA-DQB1	5.69E-211	1.444694311	0.83	0.229	1.17E-206	3
PLAUR	8.70E-211	1.146183779	0.665	0.133	1.79E-206	3
VSIR	2.78E-209	0.81861967	0.773	0.185	5.72E-205	3
HLA-DPB1	1.41E-208	1.503739828	0.937	0.349	2.91E-204	3
PILRA	2.97E-207	0.64516636	0.614	0.111	6.12E-203	3
TIMP1	4.54E-207	1.279038471	0.885	0.259	9.34E-203	3
TNFAIP2	8.55E-207	0.946961342	0.753	0.181	1.76E-202	3
CLEC4E	1.03E-203	0.685530036	0.415	0.045	2.13E-199	3
CLEC4A	2.27E-203	0.587651398	0.505	0.073	4.68E-199	3
ITGAX	9.95E-203	0.712851764	0.646	0.125	2.05E-198	3
CLEC7A	1.49E-201	0.642765023	0.607	0.11	3.07E-197	3
HLA-DMA	5.78E-200	1.065237303	0.904	0.282	1.19E-195	3
PLEKHO1	4.29E-198	0.964777694	0.763	0.201	8.84E-194	3
LRRC25	4.78E-198	0.646403311	0.581	0.104	9.84E-194	3
PYCARD	3.49E-195	0.920832976	0.847	0.28	7.18E-191	3
HLA-DQA1	8.36E-195	1.315263691	0.793	0.219	1.72E-190	3
HLA-DQA2	5.62E-193	1.179269682	0.751	0.194	1.16E-188	3
SAMHD1	2.81E-192	0.986650532	0.912	0.331	5.78E-188	3
S100A6	1.54E-191	1.337857034	0.969	0.41	3.18E-187	3
ZEB2	2.36E-191	0.816635947	0.802	0.202	4.87E-187	3
CD74	3.41E-190	1.387192309	0.994	0.59	7.03E-186	3
CSF1R	7.78E-188	0.715060773	0.656	0.131	1.60E-183	3
KCTD12	2.55E-187	0.750520136	0.697	0.151	5.25E-183	3
LCP1	1.04E-183	1.000525819	0.947	0.371	2.14E-179	3
SH3BGRL3	3.53E-183	1.115253206	1	0.73	7.27E-179	3
IFI30	1.30E-176	0.714149775	0.526	0.095	2.67E-172	3
CTSH	2.27E-176	0.989600077	0.851	0.336	4.68E-172	3
LY86	2.44E-176	0.582054368	0.601	0.121	5.03E-172	3
LILRA2	7.01E-176	0.40724901	0.335	0.032	1.44E-171	3
THEMIS2	8.82E-176	0.655415054	0.671	0.153	1.82E-171	3
BCL11A	4.19E-175	0.350442249	0.284	0.019	8.63E-171	3
ADA2	9.31E-174	0.69783653	0.734	0.173	1.92E-169	3
LILRB1	1.81E-171	0.651332515	0.538	0.103	3.73E-167	3
ITGB2	1.01E-169	0.836019433	0.922	0.31	2.09E-165	3
FAM49A	3.47E-169	0.553620625	0.579	0.119	7.14E-165	3
LGALS9	1.36E-168	0.594614655	0.701	0.168	2.80E-164	3
LMO2	8.83E-168	0.485893196	0.521	0.096	1.82E-163	3
CD14	5.35E-167	1.067876763	0.775	0.229	1.10E-162	3
FCGR1A	6.61E-166	0.591573213	0.468	0.076	1.36E-161	3
G0S2	5.78E-162	1.327071151	0.444	0.071	1.19E-157	3
ACTB	7.11E-162	1.006506719	1	0.941	1.47E-157	3
CYBA	5.71E-161	0.962335546	0.977	0.499	1.18E-156	3

YBX3	3.80E-160	0.793769806	0.708	0.188	7.83E-156	3
CASP1	3.62E-159	0.724584315	0.746	0.213	7.45E-155	3
IER5	2.51E-158	1.256769387	0.904	0.435	5.16E-154	3
PHACTR1	5.48E-158	0.84134895	0.515	0.101	1.13E-153	3
1-Mar	1.22E-157	0.540578553	0.591	0.127	2.51E-153	3
ADGRE2	2.03E-157	0.5257363	0.464	0.08	4.19E-153	3
CD86	1.09E-156	0.549259361	0.614	0.134	2.24E-152	3
WARS	1.24E-156	0.879891311	0.648	0.18	2.56E-152	3
EFHD2	4.48E-155	0.816309871	0.881	0.368	9.22E-151	3
QPCT	4.40E-153	0.34402261	0.317	0.033	9.06E-149	3
MS4A7	2.65E-152	0.644036365	0.697	0.161	5.46E-148	3
OAZ1	1.80E-150	0.790063392	0.992	0.786	3.71E-146	3
RAB31	2.01E-150	0.566462172	0.613	0.143	4.13E-146	3
SCIMP	2.85E-149	0.468433122	0.448	0.079	5.86E-145	3
PLBD1	3.28E-149	0.590905979	0.434	0.074	6.75E-145	3
GLIPR1	1.02E-148	0.668795732	0.761	0.229	2.10E-144	3
PLEK	3.01E-148	0.85327691	0.755	0.214	6.19E-144	3
CSF3R	4.94E-148	0.472969193	0.391	0.06	1.02E-143	3
BAG3	1.65E-147	1.226457635	0.742	0.262	3.40E-143	3
SLC7A7	8.03E-147	0.561917775	0.54	0.117	1.65E-142	3
H3F3A	2.95E-146	0.713922357	0.996	0.884	6.08E-142	3
S100A4	3.37E-146	1.02927634	0.957	0.411	6.94E-142	3
FGD2	1.06E-144	0.487969838	0.446	0.081	2.18E-140	3
LGALS3	9.63E-144	0.697251277	0.82	0.244	1.98E-139	3
ALOX5	1.31E-143	0.413279309	0.315	0.036	2.71E-139	3
C15orf48	3.09E-143	0.98205573	0.429	0.074	6.37E-139	3
AP1S2	2.36E-141	0.719813075	0.705	0.226	4.86E-137	3
TBXAS1	4.28E-141	0.491792634	0.53	0.116	8.82E-137	3
SAT1	9.17E-141	1.085775807	0.992	0.705	1.89E-136	3
CD68	9.82E-141	0.646543638	0.865	0.298	2.02E-136	3
IRAK3	6.25E-140	0.41636281	0.346	0.048	1.29E-135	3
RGS10	2.37E-139	0.69016368	0.759	0.235	4.88E-135	3
ARPC3	4.60E-139	0.772657722	0.969	0.737	9.48E-135	3
SNAI1	8.62E-139	0.564767603	0.276	0.027	1.77E-134	3
RNASE6	1.41E-138	0.559825469	0.622	0.147	2.91E-134	3
SERPINB9	2.05E-137	0.661904423	0.573	0.139	4.21E-133	3
CSF2RA	6.74E-137	0.407685552	0.382	0.061	1.39E-132	3
CD300A	2.42E-136	0.525104089	0.579	0.138	4.98E-132	3
CIITA	2.60E-136	0.440446653	0.458	0.089	5.36E-132	3
ABI3	8.25E-136	0.549852885	0.589	0.145	1.70E-131	3
PLSCR1	1.37E-135	0.611599692	0.595	0.17	2.82E-131	3
SYK	1.61E-134	0.449870783	0.503	0.109	3.31E-130	3
CARD16	3.34E-134	0.645641481	0.834	0.286	6.88E-130	3
SRGN	1.75E-133	1.033956532	0.992	0.492	3.60E-129	3
PTGS2	2.79E-133	0.869220006	0.354	0.053	5.74E-129	3

RGS18	2.92E-133	0.387210126	0.358	0.054	6.02E-129	3
P2RY13	4.96E-133	0.435240241	0.36	0.054	1.02E-128	3
TMSB10	1.06E-131	0.885495729	1	0.892	2.17E-127	3
PLXDC2	1.40E-131	0.418961633	0.415	0.075	2.89E-127	3
NPC2	4.51E-131	0.828655214	0.943	0.516	9.28E-127	3
MAFB	6.05E-131	1.050425	0.728	0.245	1.25E-126	3
EGR1	2.29E-130	1.219639879	0.677	0.225	4.72E-126	3
SPINT2	1.20E-129	0.521977309	0.499	0.116	2.47E-125	3
PSAP	2.62E-129	0.84675034	0.973	0.616	5.39E-125	3
EMILIN2	3.35E-129	0.484661919	0.495	0.11	6.90E-125	3
OSCAR	1.12E-127	0.350638562	0.29	0.035	2.31E-123	3
NCF1	1.50E-127	0.68720027	0.53	0.131	3.08E-123	3
MYO1F	7.41E-127	0.509789862	0.658	0.188	1.53E-122	3
ZNF385A	3.25E-126	0.405413339	0.442	0.092	6.69E-122	3
CXCL8	1.09E-125	1.038374987	0.405	0.076	2.24E-121	3
CD33	2.19E-125	0.410853103	0.429	0.085	4.51E-121	3
HCLS1	5.37E-125	0.579310075	0.812	0.274	1.11E-120	3
RGS2	2.75E-124	0.972852047	0.818	0.318	5.67E-120	3
H2AFY	1.43E-123	0.722409632	0.847	0.399	2.94E-119	3
RHOG	1.24E-121	0.651315822	0.8	0.341	2.55E-117	3
C5AR1	3.59E-121	0.921618021	0.493	0.116	7.40E-117	3
STXBP2	9.89E-121	0.587561328	0.628	0.204	2.04E-116	3
ARRB2	1.76E-120	0.589859185	0.712	0.249	3.63E-116	3
C19orf38	3.97E-120	0.303297401	0.264	0.03	8.18E-116	3
DNAJA4	5.19E-120	1.108447623	0.607	0.186	1.07E-115	3
PTPN6	8.02E-120	0.597567417	0.714	0.247	1.65E-115	3
OGFRL1	1.36E-119	0.454447988	0.526	0.133	2.80E-115	3
NAGK	3.49E-119	0.694735961	0.73	0.282	7.18E-115	3
FTH1	9.34E-119	0.740959459	1	0.979	1.92E-114	3
SLC8A1	1.22E-117	0.422672316	0.44	0.095	2.50E-113	3
SMIM25	1.29E-117	0.621274902	0.37	0.067	2.66E-113	3
BASP1	2.83E-117	0.744161433	0.55	0.147	5.84E-113	3
IFNGR1	2.61E-116	0.643912487	0.695	0.25	5.38E-112	3
EMP3	3.74E-116	0.714681353	0.89	0.366	7.71E-112	3
SIGLEC10	4.67E-116	0.40324251	0.327	0.051	9.62E-112	3
FOSB	6.44E-116	1.023889611	0.867	0.395	1.33E-111	3
NR4A1	7.24E-116	0.888665369	0.734	0.273	1.49E-111	3
IER3	1.06E-115	1.115190366	0.81	0.408	2.17E-111	3
LYL1	1.60E-115	0.346734345	0.405	0.08	3.29E-111	3
EVI2B	1.55E-114	0.568388693	0.658	0.202	3.19E-110	3
CCL3L1	2.67E-114	1.561534445	0.609	0.19	5.50E-110	3
SULF2	7.77E-114	0.423021774	0.436	0.101	1.60E-109	3
CD53	5.36E-113	0.61854178	0.908	0.359	1.10E-108	3
CREB5	6.27E-113	0.381819501	0.356	0.063	1.29E-108	3
CFD	8.04E-113	0.676688888	0.505	0.134	1.66E-108	3

HSPH1	1.43E-112	1.100500385	0.873	0.473	2.95E-108	3
ANXA5	1.55E-112	0.66691946	0.853	0.394	3.19E-108	3
RNASET2	1.72E-112	0.666136297	0.818	0.344	3.54E-108	3
SOCS3	4.09E-112	0.774099258	0.603	0.18	8.41E-108	3
FYB1	5.18E-112	0.654381895	0.867	0.33	1.07E-107	3
DSE	1.60E-111	0.466018326	0.407	0.087	3.30E-107	3
LAT2	2.97E-111	0.36460506	0.44	0.097	6.11E-107	3
ATP6V1B2	1.34E-110	0.565727234	0.712	0.286	2.76E-106	3
METRNL	1.93E-110	0.514512744	0.585	0.165	3.97E-106	3
CD44	3.27E-110	0.694489798	0.767	0.275	6.74E-106	3
LGALS1	7.08E-110	0.719907426	0.959	0.56	1.46E-105	3
TNFAIP8L2	8.77E-110	0.396073586	0.421	0.091	1.81E-105	3
WSB1	1.34E-109	0.718126906	0.898	0.514	2.75E-105	3
CD300LF	1.38E-109	0.364734199	0.319	0.053	2.84E-105	3
PARVG	1.64E-109	0.401215901	0.511	0.132	3.37E-105	3
C4orf48	6.60E-109	0.463986604	0.614	0.184	1.36E-104	3
SECTM1	9.83E-108	0.392111205	0.372	0.075	2.02E-103	3
WAS	4.58E-107	0.410166149	0.573	0.162	9.43E-103	3
CD52	7.30E-107	0.625456718	0.847	0.33	1.50E-102	3
PTPRC	2.37E-106	0.593126992	0.941	0.394	4.87E-102	3
VAMP8	4.78E-106	0.708538397	0.89	0.522	9.84E-102	3
TIMP2	6.67E-106	0.409612072	0.656	0.181	1.37E-101	3
SLAMF8	9.71E-106	0.403521604	0.432	0.099	2.00E-101	3
BATF3	1.12E-104	0.302773194	0.288	0.043	2.30E-100	3
GPSM3	2.07E-104	0.50206993	0.789	0.291	4.25E-100	3
ARHGDIB	2.30E-104	0.656005315	0.963	0.47	4.73E-100	3
RTN1	3.66E-104	0.271807753	0.286	0.043	7.53E-100	3
ATP1B3	4.11E-104	0.918081432	0.765	0.337	8.47E-100	3
ZYX	4.77E-104	0.560549613	0.677	0.261	9.82E-100	3
HSPA6	1.08E-103	0.89543248	0.701	0.256	2.22E-99	3
RAC1	2.65E-103	0.589556652	0.961	0.668	5.46E-99	3
RNF130	5.40E-103	0.559552078	0.699	0.277	1.11E-98	3
HRH2	1.71E-102	0.30372495	0.252	0.033	3.52E-98	3
CTSZ	7.32E-102	0.513857123	0.926	0.527	1.51E-97	3
IRF8	4.86E-101	0.521040281	0.493	0.131	1.00E-96	3
LILRB4	5.14E-101	0.411174519	0.454	0.11	1.06E-96	3
IFNGR2	5.80E-101	0.544553062	0.65	0.251	1.20E-96	3
NCKAP1L	1.26E-100	0.424724037	0.648	0.2	2.59E-96	3
SLC15A3	1.34E-100	0.37374845	0.493	0.124	2.76E-96	3
GMFG	1.57E-100	0.57334401	0.885	0.394	3.23E-96	3
CD4	2.30E-100	0.462394214	0.714	0.228	4.73E-96	3
PKM	3.87E-100	0.497497455	0.857	0.338	7.96E-96	3
ATG3	7.65E-100	0.585369252	0.705	0.332	1.58E-95	3
SERPINB1	2.45E-99	0.689749792	0.765	0.375	5.05E-95	3
TMSB4X	3.48E-99	0.744089867	1	0.811	7.16E-95	3

HSP90AA1	5.64E-99	0.905731034	1	0.882	1.16E-94	3
CD48	7.57E-99	0.553974694	0.81	0.319	1.56E-94	3
AP2S1	1.05E-98	0.565345551	0.885	0.514	2.16E-94	3
VIM	1.10E-98	0.637742087	0.98	0.542	2.27E-94	3
LAPTM5	2.52E-98	0.64464336	0.939	0.408	5.18E-94	3
DOK2	4.79E-98	0.60443413	0.75	0.288	9.87E-94	3
VASP	7.59E-98	0.533968176	0.742	0.316	1.56E-93	3
CORO1A	7.90E-98	0.49277476	0.869	0.335	1.63E-93	3
TGFBI	8.57E-98	0.528198081	0.681	0.251	1.76E-93	3
HCST	2.13E-97	0.618559239	0.9	0.375	4.39E-93	3
CSF2RB	2.24E-97	0.301524626	0.333	0.064	4.61E-93	3
FCGR1B	4.85E-97	0.291518972	0.266	0.04	9.98E-93	3
UCP2	6.45E-97	0.587212844	0.795	0.305	1.33E-92	3
UNC93B1	7.61E-97	0.442109261	0.575	0.19	1.57E-92	3
CDKN1A	8.77E-97	0.660264574	0.538	0.163	1.81E-92	3
CD83	1.12E-96	0.85745171	0.597	0.203	2.31E-92	3
LY96	2.96E-96	0.382973411	0.603	0.175	6.10E-92	3
GNAI2	2.27E-95	0.602567314	0.949	0.684	4.68E-91	3
ADAP2	2.39E-95	0.381636289	0.528	0.142	4.92E-91	3
P2RY6	2.45E-95	0.296571778	0.321	0.06	5.05E-91	3
SLC16A3	3.75E-95	0.405075195	0.476	0.128	7.73E-91	3
TFEC	6.97E-95	0.36266617	0.38	0.086	1.44E-90	3
UBE2D1	9.18E-95	0.481694527	0.609	0.239	1.89E-90	3
RAB32	1.49E-94	0.49180635	0.609	0.228	3.08E-90	3
LAIR1	3.31E-94	0.45363318	0.524	0.146	6.81E-90	3
HSPA1B	3.42E-94	0.655855645	0.984	0.6	7.05E-90	3
CCL3	6.54E-94	1.423651755	0.536	0.173	1.35E-89	3
RGS19	8.16E-94	0.409848921	0.569	0.184	1.68E-89	3
LSP1	6.15E-93	0.583494245	0.8	0.329	1.27E-88	3
ANXA1	1.76E-92	0.481707299	0.779	0.293	3.63E-88	3
FOS	2.68E-92	0.766526944	0.883	0.451	5.52E-88	3
MTHFD2	3.66E-92	0.404572626	0.497	0.143	7.54E-88	3
GLIPR2	1.88E-91	0.40092939	0.511	0.146	3.87E-87	3
HSPA1A	3.09E-91	0.695334077	1	0.854	6.36E-87	3
CNPY3	3.42E-91	0.543524934	0.71	0.332	7.05E-87	3
CCR1	3.72E-91	0.451658514	0.45	0.117	7.66E-87	3
TLR2	3.91E-91	0.374129651	0.329	0.068	8.06E-87	3
U62317.5	5.68E-91	0.257359084	0.26	0.041	1.17E-86	3
ZFP36	9.82E-91	0.823259982	0.779	0.36	2.02E-86	3
ITGAM	5.79E-90	0.353182962	0.391	0.092	1.19E-85	3
LAP3	2.54E-89	0.603295533	0.691	0.317	5.23E-85	3
ARPC2	6.60E-89	0.590154023	0.965	0.742	1.36E-84	3
RIN3	9.02E-89	0.340217265	0.434	0.113	1.86E-84	3
VSIG4	1.87E-88	0.570298625	0.389	0.093	3.85E-84	3
NAAA	3.49E-88	0.41359704	0.483	0.147	7.20E-84	3

MEF2C	5.13E-88	0.325637375	0.548	0.169	1.06E-83	3
DUSP1	1.19E-87	0.834301167	0.93	0.611	2.45E-83	3
NR4A2	1.48E-87	0.985565691	0.646	0.261	3.05E-83	3
CPPED1	2.55E-87	0.534970177	0.624	0.285	5.24E-83	3
PPP1R15A	2.58E-87	0.738486556	0.828	0.428	5.32E-83	3
NEAT1	9.15E-87	0.67826569	0.984	0.763	1.89E-82	3
CFL1	1.04E-86	0.569161523	0.978	0.831	2.14E-82	3
RNH1	1.41E-86	0.571483155	0.769	0.426	2.91E-82	3
CD93	1.94E-86	0.370610407	0.474	0.13	4.00E-82	3
PAK1	2.13E-86	0.45067026	0.452	0.14	4.39E-82	3
RASGEF1B	2.37E-86	0.409600592	0.348	0.079	4.89E-82	3
SOD2	3.79E-86	1.054845907	0.875	0.539	7.80E-82	3
LCP2	4.84E-86	0.426400301	0.652	0.223	9.97E-82	3
PLAC8	5.22E-86	0.543463269	0.364	0.078	1.08E-81	3
TSPO	6.28E-86	0.596919855	0.759	0.368	1.29E-81	3
GPR183	7.68E-86	0.801388856	0.466	0.134	1.58E-81	3
NCF4	5.22E-85	0.350865138	0.487	0.141	1.08E-80	3
ATP6V0D1	5.70E-85	0.492668492	0.714	0.349	1.17E-80	3
RASSF4	6.81E-85	0.527594239	0.597	0.247	1.40E-80	3
BTK	8.74E-85	0.313974917	0.354	0.084	1.80E-80	3
SLC25A6	1.71E-84	0.595177073	0.886	0.605	3.52E-80	3
RELT	1.24E-83	0.291331068	0.315	0.065	2.56E-79	3
PTAFR	1.26E-83	0.323186941	0.374	0.091	2.59E-79	3
BID	1.41E-83	0.513265014	0.671	0.329	2.91E-79	3
KCNK6	1.42E-83	0.361794405	0.386	0.096	2.93E-79	3
OTULINL	4.17E-83	0.310593356	0.393	0.1	8.58E-79	3
PPIF	5.17E-83	0.760770946	0.614	0.278	1.07E-78	3
SLAMF7	6.15E-83	0.426988769	0.366	0.085	1.27E-78	3
SLC31A2	4.54E-82	0.393266675	0.409	0.112	9.36E-78	3
GSN	5.45E-82	0.62160693	0.658	0.273	1.12E-77	3
TGFB1	2.11E-81	0.360144391	0.628	0.222	4.35E-77	3
ATP2B1-AS1	5.37E-81	0.626426381	0.448	0.135	1.11E-76	3
LITAF	1.17E-80	0.635691128	0.828	0.483	2.40E-76	3
PFN1	1.23E-80	0.54483758	0.978	0.811	2.53E-76	3
PPT1	2.55E-80	0.492725139	0.701	0.316	5.25E-76	3
GCA	7.66E-80	0.485835786	0.587	0.258	1.58E-75	3
SGK1	8.12E-80	0.634606311	0.62	0.23	1.67E-75	3
PSME2	8.66E-80	0.730362217	0.885	0.62	1.78E-75	3
TENT5A	9.18E-80	0.805197955	0.599	0.257	1.89E-75	3
STX11	1.24E-79	0.336284515	0.387	0.101	2.55E-75	3
SH3BGRL	1.26E-79	0.40112941	0.755	0.292	2.60E-75	3
IL10RA	1.78E-79	0.395521286	0.626	0.218	3.66E-75	3
BLVRA	2.46E-79	0.379311057	0.571	0.209	5.07E-75	3
FPR3	3.52E-79	0.376350485	0.436	0.12	7.24E-75	3
DAPP1	6.52E-79	0.308226818	0.35	0.083	1.34E-74	3

MYD88	6.93E-79	0.4373948	0.513	0.196	1.43E-74	3
DMXL2	1.56E-78	0.540121112	0.479	0.169	3.21E-74	3
ACTR3	2.34E-78	0.506710421	0.861	0.527	4.83E-74	3
GRN	1.40E-77	0.494437304	0.898	0.533	2.89E-73	3
ARPC5	4.42E-77	0.529055483	0.877	0.584	9.10E-73	3
EIF4A3	8.08E-77	0.746703421	0.703	0.374	1.66E-72	3
FERMT3	1.78E-76	0.378060607	0.626	0.22	3.67E-72	3
TNFRSF1B	3.61E-76	0.379558345	0.705	0.268	7.43E-72	3
FAM49B	5.83E-76	0.44191248	0.771	0.392	1.20E-71	3
ADPGK	9.25E-76	0.419511241	0.595	0.251	1.91E-71	3
ANXA2	2.32E-74	0.58874298	0.888	0.603	4.78E-70	3
PLA2G7	2.36E-74	0.36285709	0.479	0.141	4.86E-70	3
SH2B3	7.71E-74	0.352561982	0.497	0.167	1.59E-69	3
FMNL1	1.03E-73	0.320955516	0.626	0.226	2.13E-69	3
GABARAP	2.26E-73	0.437808196	0.613	0.284	4.65E-69	3
RAB20	1.85E-72	0.336991978	0.429	0.127	3.81E-68	3
SKAP2	2.23E-72	0.474687037	0.626	0.294	4.59E-68	3
MYL6	3.66E-72	0.464817111	0.99	0.844	7.55E-68	3
POU2F2	9.43E-72	0.373331491	0.456	0.141	1.94E-67	3
GNB4	2.52E-71	0.33287374	0.417	0.122	5.19E-67	3
C15orf39	2.97E-71	0.307939785	0.315	0.078	6.12E-67	3
SERPINB6	3.09E-71	0.352101528	0.624	0.265	6.37E-67	3
PPP1R18	4.26E-71	0.325441111	0.701	0.286	8.78E-67	3
GPCPD1	8.93E-71	0.451566289	0.411	0.131	1.84E-66	3
ETV6	9.46E-71	0.347123867	0.407	0.125	1.95E-66	3
TCIRG1	1.48E-70	0.380676466	0.593	0.253	3.04E-66	3
FAR1	3.38E-70	0.28591437	0.364	0.099	6.96E-66	3
EVI2A	4.18E-70	0.359257372	0.481	0.155	8.61E-66	3
DPYSL2	5.62E-70	0.39256677	0.54	0.209	1.16E-65	3
CEBPD	6.23E-70	0.73433383	0.84	0.494	1.28E-65	3
DOCK2	1.22E-69	0.333308042	0.462	0.146	2.52E-65	3
NR4A3	1.33E-69	0.456205276	0.288	0.065	2.74E-65	3
CD37	2.27E-69	0.361200057	0.757	0.32	4.67E-65	3
DNAJB1	2.67E-69	0.62543164	0.916	0.599	5.49E-65	3
ACTR2	3.64E-69	0.513145925	0.843	0.545	7.51E-65	3
HMOX1	4.16E-69	0.384372214	0.568	0.207	8.57E-65	3
PARVB	6.47E-69	0.295134005	0.409	0.122	1.33E-64	3
TACC1	1.25E-68	0.432782719	0.665	0.298	2.57E-64	3
IL4I1	1.90E-68	0.487706929	0.346	0.092	3.92E-64	3
STAB1	2.30E-68	0.310133843	0.477	0.15	4.73E-64	3
IFI16	2.65E-68	0.344121352	0.748	0.306	5.47E-64	3
TNFAIP8	3.75E-68	0.406995337	0.583	0.226	7.73E-64	3
NAIP	7.17E-68	0.348158693	0.403	0.127	1.48E-63	3
IER2	9.19E-68	0.740015065	0.826	0.471	1.89E-63	3
FES	1.80E-67	0.28932561	0.36	0.103	3.71E-63	3

ARPC1B	2.73E-67	0.492304368	0.881	0.604	5.61E-63	3
CAPG	3.19E-67	0.474378425	0.568	0.238	6.58E-63	3
SLC43A2	4.52E-67	0.390808664	0.405	0.134	9.32E-63	3
PSTPIP2	5.33E-67	0.315671809	0.294	0.072	1.10E-62	3
SLC20A1	5.69E-67	0.60640988	0.593	0.263	1.17E-62	3
PEA15	7.02E-67	0.419398617	0.587	0.278	1.45E-62	3
PSMB3	1.03E-66	0.410128191	0.863	0.551	2.13E-62	3
RHOQ	1.13E-66	0.373406455	0.517	0.2	2.33E-62	3
GLRX	2.02E-66	0.431516603	0.72	0.383	4.16E-62	3
LPCAT2	2.63E-66	0.294525251	0.348	0.102	5.42E-62	3
DENND3	3.11E-66	0.254824041	0.292	0.068	6.41E-62	3
CXCL2	4.28E-66	1.029714663	0.36	0.108	8.82E-62	3
SERF2	4.47E-66	0.387719069	0.994	0.905	9.20E-62	3
ARRB1	6.73E-66	0.346596333	0.407	0.139	1.39E-61	3
GRB2	1.46E-65	0.447140074	0.748	0.428	3.00E-61	3
SIGLEC7	1.60E-65	0.297454236	0.339	0.091	3.30E-61	3
SLC11A1	1.81E-65	0.538522118	0.26	0.059	3.73E-61	3
CYTH4	3.07E-65	0.311368141	0.477	0.159	6.32E-61	3
FCGR2B	4.57E-65	0.364671986	0.26	0.055	9.41E-61	3
FAU	6.28E-65	0.350083881	0.996	0.896	1.29E-60	3
CXCL16	7.52E-65	0.393780301	0.595	0.254	1.55E-60	3
SIRPA	8.77E-65	0.398363224	0.517	0.216	1.81E-60	3
ADCY7	9.47E-65	0.253397625	0.399	0.119	1.95E-60	3
PFKFB3	1.42E-64	0.502697817	0.366	0.106	2.93E-60	3
TUBA1A	3.24E-64	0.460635336	0.654	0.27	6.68E-60	3
FGD4	4.53E-64	0.375703254	0.411	0.146	9.33E-60	3
TPM4	5.55E-64	0.343570232	0.742	0.342	1.14E-59	3
MSN	8.75E-64	0.390947214	0.779	0.382	1.80E-59	3
CAP1	1.30E-63	0.490568699	0.888	0.634	2.67E-59	3
IL18BP	1.99E-63	0.291560271	0.407	0.124	4.10E-59	3
THBD	2.93E-63	0.387386336	0.252	0.054	6.03E-59	3
TAGLN2	8.52E-63	0.442320831	0.82	0.438	1.75E-58	3
AREG	9.39E-63	0.726149016	0.292	0.07	1.93E-58	3
CTSC	1.01E-62	0.402373328	0.804	0.389	2.08E-58	3
HK1	1.07E-62	0.289819665	0.393	0.122	2.21E-58	3
POLE4	1.68E-62	0.37144729	0.523	0.221	3.45E-58	3
NFKBID	2.54E-62	0.353458379	0.419	0.134	5.24E-58	3
MXD1	4.08E-62	0.541372475	0.481	0.187	8.39E-58	3
PRELID1	4.45E-62	0.46865823	0.859	0.579	9.17E-58	3
ASAH1	5.09E-62	0.544007657	0.763	0.442	1.05E-57	3
PRKCB	5.36E-62	0.283648204	0.452	0.149	1.10E-57	3
ATF3	6.00E-62	0.656562763	0.609	0.283	1.24E-57	3
JUNB	7.29E-62	0.368416308	0.92	0.52	1.50E-57	3
SOX4	9.28E-62	0.556422743	0.389	0.119	1.91E-57	3
GBP1	2.06E-61	0.831594671	0.581	0.278	4.24E-57	3

SLC2A3	6.94E-61	0.74281159	0.591	0.247	1.43E-56	3
NFKBIZ	7.57E-61	0.823304691	0.638	0.345	1.56E-56	3
ARHGAP27	1.22E-60	0.285650002	0.319	0.088	2.52E-56	3
GNAQ	2.15E-60	0.403529674	0.538	0.248	4.43E-56	3
FXYD5	2.51E-60	0.361721074	0.738	0.326	5.18E-56	3
RBM3	2.83E-60	0.407855956	0.82	0.527	5.83E-56	3
EPB41L3	2.96E-60	0.289735415	0.348	0.102	6.09E-56	3
GDI2	4.62E-60	0.484840399	0.779	0.516	9.52E-56	3
CCDC88A	4.88E-60	0.449973343	0.675	0.362	1.00E-55	3
TXNIP	4.93E-60	0.584201367	0.898	0.618	1.01E-55	3
LIMD2	6.38E-60	0.277740645	0.71	0.305	1.31E-55	3
GAS7	7.50E-60	0.313010001	0.311	0.086	1.54E-55	3
FAM110A	7.85E-60	0.292891863	0.37	0.116	1.62E-55	3
MOB1A	1.05E-59	0.403881934	0.755	0.447	2.17E-55	3
YBX1	1.15E-59	0.390947217	0.947	0.779	2.38E-55	3
NAMPT	4.09E-59	0.87243795	0.744	0.485	8.42E-55	3
DNAJB6	4.88E-59	0.691800805	0.787	0.525	1.01E-54	3
DUSP6	7.14E-59	0.690363422	0.638	0.35	1.47E-54	3
RIPK2	9.92E-59	0.471985063	0.421	0.162	2.04E-54	3
CAPZB	1.18E-58	0.39028802	0.888	0.593	2.43E-54	3
SMCO4	1.20E-58	0.432766057	0.521	0.252	2.48E-54	3
TET2	1.55E-58	0.285012672	0.321	0.095	3.18E-54	3
SERP1	1.59E-58	0.427805629	0.886	0.647	3.26E-54	3
SDCBP	2.11E-58	0.406739646	0.828	0.498	4.35E-54	3
CCL4L2	4.83E-58	0.49748477	0.55	0.225	9.95E-54	3
MRC1	1.16E-57	0.317492292	0.432	0.14	2.38E-53	3
ICAM1	1.23E-57	0.551865561	0.558	0.262	2.54E-53	3
SRI	2.02E-57	0.326075592	0.624	0.277	4.16E-53	3
UBE2L6	2.25E-57	0.376734474	0.614	0.293	4.63E-53	3
MCL1	3.73E-57	0.582902792	0.857	0.597	7.69E-53	3
BST2	5.24E-57	0.32644929	0.71	0.303	1.08E-52	3
IQGAP1	7.35E-57	0.36116593	0.703	0.314	1.51E-52	3
UNC119	7.86E-57	0.265160657	0.344	0.108	1.62E-52	3
RAB7A	9.09E-57	0.38153848	0.814	0.522	1.87E-52	3
ABRACL	1.03E-56	0.292669205	0.644	0.266	2.13E-52	3
ZFAND5	1.79E-56	0.567480027	0.712	0.431	3.68E-52	3
YWHAB	2.17E-56	0.420180436	0.916	0.693	4.47E-52	3
ARPC4	3.33E-56	0.404583573	0.798	0.508	6.86E-52	3
RBPJ	3.41E-56	0.360707335	0.693	0.379	7.01E-52	3
PGLS	5.44E-56	0.414171488	0.71	0.427	1.12E-51	3
DOCK8	6.12E-56	0.253224918	0.479	0.174	1.26E-51	3
GIMAP4	6.58E-56	0.35466226	0.624	0.258	1.35E-51	3
RPS4Y1	8.53E-56	0.30912563	0.769	0.358	1.76E-51	3
ACER3	8.56E-56	0.299232623	0.37	0.126	1.76E-51	3
MARCKS	1.26E-55	0.549872856	0.836	0.518	2.59E-51	3

PFDN5	1.35E-55	0.354711547	0.982	0.815	2.78E-51	3
HSPA8	1.47E-55	0.506109256	0.939	0.728	3.03E-51	3
MYO9B	5.47E-55	0.32690986	0.483	0.195	1.13E-50	3
GADD45B	6.58E-55	0.632687768	0.777	0.459	1.36E-50	3
NFKB1	2.03E-54	0.424190037	0.477	0.209	4.18E-50	3
RILPL2	2.32E-54	0.354596095	0.526	0.233	4.78E-50	3
REL	2.59E-54	0.446194634	0.714	0.399	5.32E-50	3
C3AR1	3.99E-54	0.277164928	0.405	0.131	8.21E-50	3
MGAT1	9.60E-54	0.422018232	0.656	0.357	1.98E-49	3
CAPNS1	1.93E-53	0.354175691	0.748	0.449	3.98E-49	3
SNX2	2.07E-53	0.358166437	0.609	0.312	4.25E-49	3
LTA4H	2.99E-53	0.493582122	0.513	0.257	6.15E-49	3
OTUD1	7.79E-53	0.506070638	0.401	0.148	1.60E-48	3
ACOT9	1.03E-52	0.258618515	0.407	0.153	2.11E-48	3
MVP	1.67E-52	0.297271069	0.517	0.227	3.44E-48	3
LYSMD2	2.17E-52	0.277138508	0.444	0.178	4.46E-48	3
RBX1	2.80E-52	0.334147299	0.838	0.549	5.77E-48	3
RAB8A	9.37E-52	0.328374009	0.607	0.321	1.93E-47	3
FKBP1A	1.08E-51	0.268732653	0.892	0.617	2.22E-47	3
COX4I1	1.85E-51	0.388226754	0.955	0.763	3.81E-47	3
CASP4	2.46E-51	0.313323497	0.62	0.317	5.08E-47	3
RNPEP	6.41E-51	0.304880519	0.497	0.228	1.32E-46	3
CSK	9.92E-51	0.318971357	0.611	0.296	2.04E-46	3
EHBP1L1	1.04E-50	0.358152412	0.534	0.254	2.13E-46	3
GRK3	1.25E-50	0.269141906	0.397	0.151	2.57E-46	3
PRMT9	1.35E-50	0.336244989	0.368	0.126	2.77E-46	3
SFT2D1	2.48E-50	0.304385623	0.569	0.273	5.10E-46	3
ENY2	2.69E-50	0.369344192	0.822	0.538	5.53E-46	3
LAMTOR2	2.96E-50	0.371268163	0.701	0.434	6.09E-46	3
CDC42	6.62E-50	0.34882864	0.857	0.591	1.36E-45	3
QKI	8.83E-50	0.36927502	0.681	0.389	1.82E-45	3
HSBP1	1.01E-49	0.33146976	0.802	0.508	2.09E-45	3
SNX10	1.25E-49	0.371455948	0.538	0.262	2.57E-45	3
PSMA4	1.53E-49	0.40055552	0.753	0.486	3.14E-45	3
CNN2	1.61E-49	0.343669257	0.583	0.295	3.32E-45	3
RPS9	2.61E-49	0.312225351	0.996	0.934	5.38E-45	3
GNG5	3.06E-49	0.350065416	0.916	0.672	6.31E-45	3
TPM3	3.84E-49	0.395766678	0.879	0.659	7.92E-45	3
LPAR6	7.55E-49	0.302186244	0.442	0.176	1.56E-44	3
TUBA1B	7.77E-49	0.345468129	0.885	0.665	1.60E-44	3
DAZAP2	1.03E-48	0.35140148	0.808	0.51	2.13E-44	3
GBP4	1.46E-48	0.364565095	0.434	0.163	3.00E-44	3
RHOA	2.10E-48	0.391474881	0.908	0.698	4.33E-44	3
BLVRB	2.75E-48	0.301277559	0.759	0.439	5.67E-44	3
BAZ1A	2.83E-48	0.377306176	0.605	0.324	5.83E-44	3

SMAP2	7.23E-48	0.3446642	0.658	0.38	1.49E-43	3
C9orf72	1.14E-47	0.343952372	0.313	0.108	2.34E-43	3
KLF2	1.97E-47	0.292907263	0.626	0.276	4.06E-43	3
ATP6V0B	1.98E-47	0.354083384	0.818	0.549	4.08E-43	3
SUB1	2.26E-47	0.350698429	0.906	0.693	4.66E-43	3
DRAM1	3.19E-47	0.31335641	0.36	0.13	6.56E-43	3
JUN	3.48E-47	0.398378174	0.918	0.66	7.16E-43	3
PABPC1	5.04E-47	0.390172198	0.969	0.794	1.04E-42	3
PGK1	5.49E-47	0.377212905	0.853	0.592	1.13E-42	3
PRR13	5.74E-47	0.348629272	0.755	0.493	1.18E-42	3
SHTN1	8.42E-47	0.308395505	0.431	0.185	1.73E-42	3
KDM6B	9.45E-47	0.429158115	0.495	0.218	1.95E-42	3
RBM47	1.60E-46	0.346043573	0.497	0.243	3.30E-42	3
EIF3K	2.53E-46	0.355057256	0.877	0.648	5.20E-42	3
ZNF706	2.57E-46	0.375680502	0.742	0.489	5.29E-42	3
HBEGF	3.30E-46	0.41474265	0.292	0.09	6.79E-42	3
UBC	3.61E-46	0.498711232	0.988	0.874	7.44E-42	3
DDX3Y	5.82E-46	0.390886505	0.503	0.211	1.20E-41	3
TNF	7.51E-46	0.416487333	0.352	0.12	1.55E-41	3
PLEKHB2	2.32E-45	0.345599721	0.579	0.304	4.79E-41	3
CEBPB	2.52E-45	0.562385379	0.812	0.54	5.19E-41	3
NUP214	3.51E-45	0.401232094	0.421	0.193	7.22E-41	3
MFSD1	4.75E-45	0.346562633	0.542	0.275	9.78E-41	3
ATP6V0E1	4.99E-45	0.335348266	0.881	0.626	1.03E-40	3
SNN	6.94E-45	0.252982971	0.321	0.112	1.43E-40	3
GLUL	7.35E-45	0.542296621	0.789	0.507	1.51E-40	3
M6PR	1.07E-44	0.340591329	0.658	0.383	2.21E-40	3
DNAJA1	1.29E-44	0.471626025	0.908	0.685	2.65E-40	3
TAOK3	2.57E-44	0.314147257	0.644	0.379	5.30E-40	3
BLOC1S1	3.34E-44	0.330306096	0.755	0.488	6.89E-40	3
LRRFIP1	3.38E-44	0.311627238	0.859	0.574	6.96E-40	3
SQOR	3.75E-44	0.288808208	0.56	0.296	7.72E-40	3
TNFAIP3	5.86E-44	0.627321691	0.587	0.305	1.21E-39	3
COX7B	6.64E-44	0.338455623	0.867	0.626	1.37E-39	3
UBE2D3	9.72E-44	0.351681299	0.847	0.604	2.00E-39	3
NABP1	1.11E-43	0.326193799	0.566	0.277	2.29E-39	3
KDM7A	1.32E-43	0.280293579	0.325	0.121	2.73E-39	3
COPE	1.46E-43	0.305426792	0.822	0.565	3.01E-39	3
HMGN2	2.05E-43	0.306005753	0.816	0.54	4.22E-39	3
MINOS1	3.38E-43	0.323893199	0.773	0.512	6.96E-39	3
NANS	7.10E-43	0.311009276	0.55	0.302	1.46E-38	3
CMTM7	9.53E-43	0.252761895	0.431	0.19	1.96E-38	3
CD63	1.71E-42	0.354054915	0.947	0.722	3.51E-38	3
HIGD2A	2.02E-42	0.311914329	0.773	0.524	4.17E-38	3
COX8A	2.29E-42	0.296867829	0.863	0.615	4.72E-38	3

ZFAND2A	3.47E-42	0.960597377	0.613	0.39	7.14E-38	3
POMP	3.72E-42	0.324752228	0.908	0.628	7.66E-38	3
FCGR3A	4.59E-42	0.391657821	0.499	0.204	9.45E-38	3
DNAJC15	5.32E-42	0.27407969	0.581	0.324	1.10E-37	3
TRA2B	6.47E-42	0.446391965	0.773	0.527	1.33E-37	3
PID1	7.12E-42	0.377088549	0.35	0.149	1.47E-37	3
FCGRT	7.79E-42	0.262808642	0.851	0.514	1.61E-37	3
HIF1A	8.16E-42	0.359958804	0.538	0.282	1.68E-37	3
CCL4	1.19E-41	0.730900002	0.472	0.214	2.45E-37	3
STAT1	1.24E-41	0.423653943	0.659	0.401	2.56E-37	3
SH3BP2	1.65E-41	0.272680757	0.37	0.147	3.39E-37	3
MRPL18	1.77E-41	0.366619705	0.724	0.472	3.64E-37	3
CSTB	1.91E-41	0.263372491	0.892	0.615	3.94E-37	3
NINJ1	2.31E-41	0.428853624	0.63	0.387	4.75E-37	3
DBNL	2.64E-41	0.281550043	0.605	0.336	5.43E-37	3
CLK1	4.30E-41	0.428108283	0.663	0.405	8.86E-37	3
PLXNC1	4.55E-41	0.264588267	0.315	0.118	9.37E-37	3
PSME1	8.44E-41	0.30622252	0.855	0.629	1.74E-36	3
THAP9-AS1	1.16E-40	0.31899624	0.297	0.103	2.40E-36	3
CD55	1.50E-40	0.348203893	0.378	0.156	3.10E-36	3
CCNI	1.55E-40	0.35483533	0.853	0.599	3.19E-36	3
SPCS3	3.10E-40	0.275196689	0.661	0.401	6.38E-36	3
PTP4A2	4.58E-40	0.377774065	0.779	0.554	9.44E-36	3
MAP3K1	4.72E-40	0.302080079	0.431	0.207	9.72E-36	3
TPI1	6.22E-40	0.340686179	0.888	0.697	1.28E-35	3
MBNL1	1.02E-39	0.285196158	0.718	0.446	2.11E-35	3
ZFP36L1	5.04E-39	0.617226015	0.867	0.634	1.04E-34	3
CTDNEP1	5.04E-39	0.251310043	0.609	0.345	1.04E-34	3
TCP1	1.30E-38	0.403166469	0.65	0.418	2.69E-34	3
DYNLT1	1.80E-38	0.254140676	0.648	0.386	3.71E-34	3
HNRNPU	2.22E-38	0.499375537	0.867	0.653	4.58E-34	3
GLA	2.86E-38	0.374630798	0.403	0.187	5.89E-34	3
PSMB8	2.94E-38	0.255475956	0.728	0.458	6.06E-34	3
NAP1L1	3.32E-38	0.31992318	0.879	0.632	6.84E-34	3
NDUFS7	3.38E-38	0.290703732	0.659	0.413	6.96E-34	3
ACTG1	4.11E-38	0.337052099	0.988	0.906	8.46E-34	3
CAPZA1	6.12E-38	0.252406925	0.732	0.464	1.26E-33	3
PTEN	8.83E-38	0.253331544	0.607	0.363	1.82E-33	3
OAZ2	1.25E-37	0.337297529	0.571	0.337	2.57E-33	3
COX5A	1.31E-37	0.299198904	0.822	0.577	2.70E-33	3
BNIP3L	1.47E-37	0.390218906	0.638	0.394	3.02E-33	3
PCBP1	1.76E-37	0.308719606	0.912	0.654	3.63E-33	3
NUMB	3.14E-37	0.325489508	0.454	0.233	6.46E-33	3
LACTB	3.63E-37	0.323317639	0.523	0.303	7.48E-33	3
VPS29	6.16E-37	0.256954162	0.746	0.499	1.27E-32	3

HLA-B	7.78E-37	0.349422757	0.994	0.634	1.60E-32	3
VMP1	1.22E-36	0.443519657	0.744	0.511	2.51E-32	3
TPP1	1.46E-36	0.2839467	0.771	0.488	3.00E-32	3
SRA1	2.62E-36	0.256887835	0.46	0.237	5.40E-32	3
ZDHHC7	3.28E-36	0.264244087	0.317	0.129	6.75E-32	3
CSRNP1	4.11E-36	0.30949499	0.425	0.192	8.47E-32	3
UPP1	4.61E-36	0.329079741	0.423	0.2	9.50E-32	3
NDUFB1	5.17E-36	0.270032513	0.685	0.442	1.06E-31	3
KYNU	5.29E-36	0.295500568	0.47	0.253	1.09E-31	3
CMTM6	7.23E-36	0.340015564	0.765	0.515	1.49E-31	3
EIF5A	1.20E-35	0.311469457	0.736	0.516	2.47E-31	3
SEPT9	2.88E-35	0.291967447	0.636	0.382	5.93E-31	3
GRINA	3.10E-35	0.328515211	0.534	0.321	6.39E-31	3
TAGAP	6.19E-35	0.371679001	0.401	0.172	1.28E-30	3
CHMP1B	8.44E-35	0.632821254	0.585	0.381	1.74E-30	3
NUP62	1.04E-34	0.269054588	0.499	0.275	2.13E-30	3
ARL6IP5	1.23E-34	0.257979361	0.787	0.528	2.54E-30	3
NXF1	1.50E-34	0.317537768	0.382	0.172	3.10E-30	3
LYST	1.55E-34	0.262839628	0.45	0.224	3.19E-30	3
LAMTOR1	1.92E-34	0.267814556	0.72	0.481	3.95E-30	3
DDX21	2.20E-34	0.331634305	0.638	0.426	4.53E-30	3
RHOB	3.88E-34	0.694043067	0.777	0.58	8.00E-30	3
EIF1	4.72E-34	0.277680472	0.992	0.908	9.71E-30	3
CHD1	4.80E-34	0.327170584	0.474	0.258	9.88E-30	3
NECAP2	9.37E-34	0.257580216	0.56	0.316	1.93E-29	3
MTPN	1.01E-33	0.307563571	0.648	0.439	2.08E-29	3
H3F3B	1.52E-33	0.347781911	0.984	0.873	3.13E-29	3
CHORDC1	1.59E-33	0.268607816	0.566	0.324	3.28E-29	3
FOSL2	2.46E-33	0.397769883	0.403	0.193	5.07E-29	3
UBE2R2	2.73E-33	0.269163045	0.614	0.397	5.63E-29	3
S100A10	2.91E-33	0.446285951	0.914	0.731	5.99E-29	3
PSMB1	3.22E-33	0.251847681	0.806	0.588	6.64E-29	3
MBD2	3.54E-33	0.255408	0.644	0.418	7.29E-29	3
TRAPPC1	5.08E-33	0.266185201	0.699	0.463	1.05E-28	3
VPS35	1.03E-32	0.278780349	0.568	0.354	2.13E-28	3
ARL5B	1.07E-32	0.612361593	0.395	0.21	2.21E-28	3
YWHAZ	1.51E-32	0.273247291	0.804	0.557	3.10E-28	3
SLC25A5	1.56E-32	0.335292474	0.83	0.632	3.21E-28	3
PICALM	6.34E-32	0.258968844	0.476	0.268	1.31E-27	3
MTHFR	1.94E-31	0.306240849	0.346	0.164	4.01E-27	3
C1QA	3.62E-31	0.309369324	0.519	0.263	7.45E-27	3
MAFF	4.03E-31	0.429637987	0.417	0.215	8.30E-27	3
SCPEP1	4.28E-31	0.310430987	0.515	0.306	8.82E-27	3
NFKBIA	4.78E-31	0.88274305	0.763	0.606	9.85E-27	3
TWISTNB	7.65E-31	0.594079077	0.438	0.25	1.58E-26	3

C19orf70	7.98E-31	0.250497959	0.607	0.393	1.64E-26	3
FKBP4	9.59E-31	0.454858213	0.542	0.342	1.98E-26	3
IL13RA1	1.17E-30	0.252605399	0.366	0.182	2.41E-26	3
KIAA0930	1.44E-30	0.291830662	0.479	0.285	2.96E-26	3
CKLF	2.23E-30	0.264496797	0.728	0.499	4.59E-26	3
SYNGR2	7.55E-30	0.261240164	0.646	0.43	1.56E-25	3
PHLDA2	9.61E-30	0.40167861	0.384	0.193	1.98E-25	3
SLC25A37	1.44E-29	0.329912332	0.393	0.213	2.97E-25	3
UBE2J1	1.70E-29	0.251196466	0.47	0.272	3.50E-25	3
ATP6V1F	2.00E-29	0.276051113	0.767	0.554	4.12E-25	3
SP110	2.41E-29	0.275321613	0.528	0.32	4.97E-25	3
PPIA	2.90E-29	0.256335404	0.957	0.838	5.97E-25	3
LIMS1	6.20E-29	0.270159118	0.691	0.474	1.28E-24	3
SNAP23	8.10E-29	0.281447272	0.503	0.306	1.67E-24	3
MBOAT7	8.19E-29	0.267732477	0.346	0.173	1.69E-24	3
C4orf3	1.80E-28	0.25884508	0.816	0.592	3.71E-24	3
TMEM167A	3.02E-28	0.250803462	0.587	0.384	6.23E-24	3
NKRF	3.53E-28	0.379479233	0.327	0.151	7.26E-24	3
IRF1	3.95E-28	0.279202526	0.677	0.415	8.14E-24	3
SCAF11	7.33E-28	0.26095242	0.681	0.478	1.51E-23	3
FBXL5	8.09E-28	0.256366602	0.372	0.19	1.67E-23	3
KLF6	1.32E-27	0.473278963	0.836	0.633	2.72E-23	3
VAMP5	2.32E-27	0.339344805	0.638	0.439	4.79E-23	3
AKR1A1	2.66E-27	0.271372306	0.64	0.441	5.47E-23	3
RNF149	3.58E-27	0.269135509	0.581	0.387	7.37E-23	3
DAPK1	4.16E-27	0.260516262	0.335	0.167	8.57E-23	3
HSPB1	5.98E-27	0.587982617	0.886	0.79	1.23E-22	3
TRIM38	6.54E-27	0.26253434	0.462	0.262	1.35E-22	3
IFI6	2.52E-26	0.325937916	0.481	0.28	5.18E-22	3
P2RX7	2.58E-26	0.294375684	0.286	0.127	5.32E-22	3
DDX3X	2.65E-26	0.339173305	0.691	0.472	5.46E-22	3
IFRD1	4.62E-26	0.395205124	0.546	0.329	9.52E-22	3
CCNL1	4.91E-26	0.279149133	0.716	0.511	1.01E-21	3
PIK3AP1	1.80E-25	0.277066162	0.431	0.251	3.70E-21	3
NAPA	2.90E-25	0.259189414	0.507	0.326	5.98E-21	3
CYCS	3.74E-25	0.290656423	0.793	0.608	7.70E-21	3
C20orf27	4.37E-25	0.278581518	0.481	0.309	9.00E-21	3
SLC5A3	5.32E-25	0.43043149	0.417	0.235	1.10E-20	3
B2M	1.08E-24	0.277828977	1	0.942	2.23E-20	3
CD40	1.22E-23	0.281654951	0.378	0.214	2.51E-19	3
BIRC3	3.21E-23	0.27513573	0.464	0.244	6.60E-19	3
HERPUD1	7.32E-22	0.324152896	0.769	0.581	1.51E-17	3
ETS2	7.92E-22	0.265090533	0.429	0.266	1.63E-17	3
MKNK2	1.35E-21	0.289401294	0.523	0.335	2.78E-17	3
DUSP2	2.35E-21	0.288202311	0.442	0.242	4.84E-17	3

MIDN	5.83E-20	0.251397788	0.546	0.372	1.20E-15	3
YPEL5	4.71E-19	0.270684543	0.581	0.423	9.70E-15	3
EIF4E	1.21E-18	0.29368431	0.464	0.307	2.49E-14	3
CKS2	6.92E-18	0.316667608	0.386	0.23	1.43E-13	3
MARCKSL1	1.81E-17	0.250321632	0.509	0.339	3.73E-13	3
GABARAPL1	1.73E-16	0.288251611	0.321	0.184	3.56E-12	3
GK	9.28E-16	0.25705933	0.258	0.143	1.91E-11	3
TNFSF10	3.74E-15	0.296310738	0.571	0.403	7.69E-11	3
IER5L	4.54E-15	0.297667559	0.481	0.345	9.34E-11	3
TRIB1	1.13E-13	0.300588396	0.331	0.215	2.33E-09	3
PPA1	1.44E-13	0.357599273	0.683	0.543	2.96E-09	3
PPP1R15B	1.64E-13	0.326235783	0.36	0.249	3.37E-09	3
ADM	1.77E-13	0.324134823	0.294	0.173	3.64E-09	3
MAT2A	3.80E-13	0.257899981	0.513	0.39	7.83E-09	3
PLIN2	1.12E-12	0.272182018	0.593	0.452	2.31E-08	3
ZNF331	1.90E-11	0.500244006	0.479	0.391	3.92E-07	3
KLF10	3.10E-11	0.262144907	0.309	0.207	6.39E-07	3
HES1	1.76E-09	0.46603472	0.386	0.297	3.63E-05	3
IL1RN	4.70E-08	0.542146901	0.295	0.225	0.000968503	3
INSIG1	6.56E-07	0.298347219	0.397	0.318	0.013520282	3
TNFRSF18	0	2.218004868	0.807	0.053	0	4
TNFRSF4	0	2.162655512	0.783	0.083	0	4
TRAC	0	1.975235633	0.951	0.151	0	4
TIGIT	0	1.656421851	0.758	0.066	0	4
SPOCK2	0	1.581948177	0.794	0.083	0	4
CD2	0	1.531063274	0.864	0.132	0	4
CD3D	0	1.504461714	0.896	0.146	0	4
DUSP4	0	1.496624005	0.624	0.065	0	4
FOXP3	0	1.378499418	0.524	0.009	0	4
CD27	0	1.377628895	0.692	0.083	0	4
LAIR2	0	1.314210005	0.648	0.066	0	4
ICOS	0	1.294288389	0.537	0.031	0	4
CTLA4	0	0.953514594	0.442	0.02	0	4
CD3E	8.58E-308	1.308207934	0.832	0.133	1.77E-303	4
TRBC2	2.86E-289	1.678676223	0.805	0.143	5.89E-285	4
IL32	1.80E-277	2.210213688	0.972	0.382	3.70E-273	4
LTB	8.10E-273	1.778392975	0.822	0.166	1.67E-268	4
BATF	7.35E-251	1.532730601	0.699	0.132	1.51E-246	4
LINC01943	1.37E-244	1.090386964	0.397	0.028	2.81E-240	4
LCK	8.76E-237	1.118153353	0.699	0.12	1.80E-232	4
SIRPG	2.14E-227	0.840211951	0.423	0.035	4.40E-223	4
CXCR3	1.19E-223	0.805049411	0.41	0.035	2.46E-219	4
CXCR6	2.31E-214	0.812512007	0.333	0.02	4.76E-210	4
CD7	5.32E-213	1.342872904	0.76	0.172	1.10E-208	4
CD3G	4.92E-203	0.929937281	0.605	0.097	1.01E-198	4

IL2RA	3.99E-196	0.977991344	0.329	0.024	8.21E-192	4
HLA-A	1.16E-189	1.046861709	0.987	0.898	2.38E-185	4
TRBC1	1.71E-187	1.543205114	0.588	0.104	3.53E-183	4
SH2D2A	4.28E-187	0.86186054	0.444	0.054	8.81E-183	4
CD247	1.07E-186	0.919946572	0.628	0.116	2.21E-182	4
TNFRSF9	3.75E-184	0.985627587	0.425	0.05	7.73E-180	4
IL7R	1.79E-180	0.897605649	0.577	0.095	3.70E-176	4
SIT1	1.85E-179	0.827302566	0.482	0.069	3.80E-175	4
RGS1	4.67E-174	1.5392806	0.758	0.227	9.63E-170	4
ITM2A	6.15E-174	1.049255757	0.577	0.106	1.27E-169	4
IL2RG	4.65E-164	1.192752305	0.8	0.299	9.57E-160	4
S100A4	7.75E-162	1.31010866	0.926	0.418	1.60E-157	4
B2M	2.14E-161	0.920483268	0.998	0.943	4.40E-157	4
RHOH	1.93E-158	0.894449551	0.51	0.093	3.97E-154	4
RASGRP1	6.60E-154	0.860088202	0.454	0.073	1.36E-149	4
CCND2	1.77E-149	1.069006562	0.58	0.143	3.65E-145	4
HLA-B	1.61E-144	0.965970183	0.985	0.638	3.33E-140	4
RTKN2	7.05E-142	0.778606876	0.285	0.028	1.45E-137	4
PIM2	2.06E-141	0.987356747	0.463	0.09	4.24E-137	4
GADD45G	3.70E-141	1.284958443	0.533	0.127	7.62E-137	4
IL2RB	8.91E-139	0.713486521	0.42	0.066	1.83E-134	4
SLAMF1	1.78E-137	0.6296294	0.301	0.032	3.67E-133	4
ACAP1	1.28E-135	0.843171422	0.594	0.147	2.63E-131	4
BTG1	5.05E-135	1.030624558	0.962	0.744	1.04E-130	4
JUND	1.14E-134	1.022042312	0.981	0.794	2.34E-130	4
TNFRSF1B	2.56E-132	1.214077875	0.707	0.271	5.28E-128	4
BIRC3	8.35E-131	1.17848309	0.671	0.227	1.72E-126	4
FYN	5.99E-129	0.924058062	0.607	0.167	1.23E-124	4
MALAT1	3.75E-128	0.876186384	1	0.949	7.73E-124	4
RAC2	1.32E-126	0.916348404	0.824	0.363	2.72E-122	4
CD96	3.29E-126	0.686522233	0.395	0.065	6.78E-122	4
ASB2	7.54E-126	0.558678933	0.263	0.026	1.55E-121	4
LAT	5.26E-123	0.786598142	0.476	0.103	1.08E-118	4
TNFRSF25	2.77E-122	0.629735422	0.335	0.048	5.70E-118	4
TSC22D3	1.55E-119	1.250162884	0.758	0.328	3.19E-115	4
HSPA1B	7.03E-118	0.969221059	0.968	0.604	1.45E-113	4
PKM	1.79E-114	1.128773267	0.779	0.349	3.68E-110	4
PMAIP1	1.04E-113	1.296594874	0.667	0.256	2.13E-109	4
PTPN7	7.88E-113	0.870671501	0.592	0.183	1.62E-108	4
TBC1D4	4.09E-112	1.039497533	0.539	0.176	8.43E-108	4
PTPRC	1.60E-110	0.850274707	0.866	0.404	3.29E-106	4
DNAJB1	2.49E-108	0.953727601	0.943	0.599	5.13E-104	4
CORO1A	7.92E-107	0.899525773	0.796	0.345	1.63E-102	4
HLA-C	8.72E-107	0.693012113	0.975	0.866	1.80E-102	4
UBC	1.12E-106	0.886122689	0.983	0.876	2.31E-102	4

CD69	1.39E-106	1.019664475	0.59	0.174	2.87E-102	4
DDX5	3.15E-104	0.798536688	0.947	0.771	6.50E-100	4
CD52	1.07E-103	0.893861114	0.794	0.339	2.21E-99	4
TRAF3IP3	2.20E-97	0.719777454	0.488	0.133	4.52E-93	4
LBH	4.90E-97	0.7282323	0.478	0.125	1.01E-92	4
CYTIP	8.46E-97	0.852818468	0.605	0.22	1.74E-92	4
IKZF2	8.22E-96	0.66754739	0.272	0.042	1.69E-91	4
BCL11B	5.64E-93	0.61419268	0.308	0.053	1.16E-88	4
GATA3	9.62E-93	0.557657252	0.308	0.052	1.98E-88	4
SKAP1	2.46E-92	0.751173047	0.461	0.13	5.06E-88	4
CST7	4.81E-92	0.595896261	0.548	0.155	9.91E-88	4
SAMSN1	1.73E-90	0.878917831	0.588	0.228	3.55E-86	4
HLA-E	4.60E-90	0.714032109	0.955	0.723	9.46E-86	4
CD79B	2.27E-88	0.592945161	0.359	0.077	4.67E-84	4
ARID5B	7.07E-87	1.051845894	0.603	0.28	1.46E-82	4
CIRBP	9.25E-86	0.83807185	0.79	0.556	1.90E-81	4
PCED1B	1.29E-85	0.566277515	0.297	0.055	2.66E-81	4
DDIT4	3.68E-85	1.098270385	0.705	0.362	7.58E-81	4
HSPA1A	5.62E-84	0.768143828	0.985	0.856	1.16E-79	4
TNFAIP3	7.68E-84	0.864096706	0.665	0.3	1.58E-79	4
STK17B	8.24E-84	0.839467968	0.556	0.214	1.70E-79	4
CD28	8.27E-84	0.632087843	0.338	0.075	1.70E-79	4
CLEC2D	8.36E-84	0.834832203	0.535	0.192	1.72E-79	4
CARD16	1.90E-83	0.878945781	0.656	0.306	3.92E-79	4
HSP90AA1	2.54E-83	0.678669102	0.989	0.884	5.22E-79	4
CORO1B	5.18E-83	1.086188722	0.688	0.432	1.07E-78	4
SOCS1	6.90E-83	0.876295482	0.507	0.179	1.42E-78	4
RPS26	1.01E-82	0.660874161	0.936	0.828	2.08E-78	4
JUN	1.97E-82	0.821706391	0.932	0.661	4.05E-78	4
SRSF7	1.05E-81	0.897888351	0.834	0.642	2.16E-77	4
TBC1D10C	2.93E-81	0.63491935	0.427	0.117	6.03E-77	4
AC008105.3	1.94E-80	0.686497028	0.31	0.065	3.99E-76	4
PFN1	6.20E-79	0.687197361	0.949	0.815	1.28E-74	4
SEPT1	9.01E-77	0.695200222	0.473	0.159	1.85E-72	4
CD6	2.18E-76	0.514310523	0.278	0.051	4.49E-72	4
JUNB	5.51E-76	0.889207768	0.837	0.53	1.13E-71	4
H3F3B	7.08E-76	0.589903121	0.972	0.875	1.46E-71	4
PHLDA1	1.77E-74	0.994872887	0.571	0.261	3.64E-70	4
APOBEC3G	3.69E-74	0.697704787	0.49	0.168	7.59E-70	4
EVL	1.24E-73	0.698746987	0.686	0.334	2.56E-69	4
NCK2	5.01E-73	0.562562573	0.306	0.068	1.03E-68	4
DUSP2	5.05E-73	0.967986744	0.577	0.231	1.04E-68	4
ARHGDIB	4.90E-72	0.675928838	0.866	0.483	1.01E-67	4
ISG20	4.98E-71	0.785413905	0.48	0.168	1.03E-66	4
CACYBP	2.30E-70	0.873601082	0.764	0.581	4.74E-66	4

SRGN	2.52E-69	0.582216831	0.909	0.503	5.20E-65	4
DNAJA1	8.33E-68	0.787574717	0.847	0.692	1.72E-63	4
ICAM3	7.14E-67	0.771629447	0.544	0.253	1.47E-62	4
FMNL1	1.23E-66	0.750860706	0.539	0.237	2.53E-62	4
RASAL3	1.35E-66	0.556729169	0.344	0.092	2.77E-62	4
AC044849.1	1.98E-66	0.514514277	0.253	0.05	4.08E-62	4
FOS	6.85E-66	0.730026738	0.82	0.46	1.41E-61	4
CD37	1.28E-65	0.590110196	0.675	0.331	2.63E-61	4
PTTG1	7.75E-65	0.69700101	0.456	0.167	1.60E-60	4
HSPA8	1.76E-64	0.67852364	0.896	0.733	3.62E-60	4
GBP5	1.53E-63	0.677895111	0.423	0.144	3.15E-59	4
DUSP1	2.00E-63	0.746944638	0.868	0.619	4.11E-59	4
HNRNPA2B1	4.59E-63	0.534074127	0.938	0.805	9.45E-59	4
BCL2	1.09E-62	0.618457604	0.367	0.114	2.25E-58	4
DDX24	1.26E-62	0.828926129	0.648	0.405	2.60E-58	4
EIF1	1.43E-62	0.490067851	0.977	0.91	2.95E-58	4
FAS	3.64E-62	0.493389169	0.251	0.053	7.50E-58	4
CD82	1.23E-61	0.814010488	0.52	0.253	2.54E-57	4
DOK2	1.28E-60	0.667517512	0.62	0.303	2.64E-56	4
ETS1	1.73E-60	0.552667456	0.344	0.096	3.56E-56	4
RGCC	3.63E-60	0.754055959	0.488	0.188	7.47E-56	4
CXCR4	6.68E-60	0.756554017	0.637	0.312	1.38E-55	4
ARL6IP5	3.09E-59	0.730486143	0.745	0.534	6.36E-55	4
GADD45A	5.41E-59	1.234635292	0.533	0.287	1.11E-54	4
LY6E	5.47E-59	0.692706338	0.611	0.324	1.13E-54	4
TMEM173	9.38E-59	0.683191179	0.416	0.156	1.93E-54	4
S100A6	1.40E-57	0.408032695	0.817	0.427	2.88E-53	4
SLA	7.11E-57	0.747694256	0.495	0.222	1.46E-52	4
CTSC	9.75E-57	0.784419269	0.701	0.401	2.01E-52	4
HLA-F	1.03E-56	0.612397102	0.473	0.194	2.12E-52	4
APOBEC3C	1.20E-56	0.703717946	0.418	0.163	2.48E-52	4
MIR155HG	4.05E-56	0.662554592	0.253	0.06	8.34E-52	4
SLC38A1	8.08E-56	0.513073961	0.335	0.098	1.66E-51	4
SUB1	9.47E-55	0.577029103	0.824	0.702	1.95E-50	4
KLF6	1.23E-53	0.897943458	0.8	0.638	2.53E-49	4
ARPC1B	1.61E-53	0.641923295	0.786	0.615	3.32E-49	4
IRF1	3.19E-53	0.684192064	0.694	0.416	6.57E-49	4
CD44	3.77E-53	0.612350575	0.584	0.296	7.76E-49	4
BTG3	1.00E-52	0.761095515	0.45	0.205	2.07E-48	4
TSPYL2	1.89E-52	0.771733869	0.446	0.184	3.89E-48	4
FYB1	2.06E-52	0.620167935	0.679	0.351	4.24E-48	4
C9orf16	1.43E-51	0.748833785	0.673	0.495	2.95E-47	4
PRDM1	2.64E-51	0.736673347	0.399	0.157	5.44E-47	4
MAF	3.19E-51	0.790388992	0.648	0.421	6.56E-47	4
ENTPD1	4.38E-51	0.740284476	0.427	0.184	9.02E-47	4

FXYD5	6.21E-51	0.632749548	0.626	0.339	1.28E-46	4
PBXIP1	8.49E-51	0.778770383	0.501	0.268	1.75E-46	4
CALM1	8.92E-51	0.511411872	0.898	0.727	1.84E-46	4
UCP2	1.21E-50	0.71348168	0.616	0.324	2.49E-46	4
GAPDH	1.91E-50	0.485697453	0.966	0.915	3.94E-46	4
ZAP70	2.05E-50	0.37946354	0.27	0.068	4.23E-46	4
RNF213	2.17E-50	0.800948656	0.667	0.487	4.47E-46	4
TENT5C	3.54E-50	0.556174533	0.263	0.07	7.29E-46	4
PSTPIP1	4.41E-50	0.492118171	0.314	0.097	9.07E-46	4
CREM	5.43E-50	0.907451323	0.548	0.316	1.12E-45	4
CCNI	6.88E-50	0.593800908	0.786	0.607	1.42E-45	4
HSPE1	7.42E-50	0.608700339	0.919	0.829	1.53E-45	4
LIMD2	1.59E-49	0.59763052	0.597	0.318	3.28E-45	4
SOD1	5.40E-49	0.522805464	0.866	0.749	1.11E-44	4
PRKCH	5.82E-49	0.468795093	0.329	0.103	1.20E-44	4
LSP1	8.11E-49	0.588145744	0.631	0.347	1.67E-44	4
PPP1R15A	1.03E-48	0.615905826	0.718	0.441	2.13E-44	4
CRIP1	2.50E-48	0.500338251	0.346	0.117	5.14E-44	4
EZR	3.58E-48	0.672220675	0.505	0.258	7.38E-44	4
ANAPC16	2.79E-47	0.690309333	0.671	0.526	5.74E-43	4
KLRB1	3.99E-47	0.633988568	0.289	0.08	8.21E-43	4
SH3KBP1	1.37E-46	0.675276751	0.463	0.224	2.82E-42	4
C12orf57	1.67E-46	0.74989866	0.616	0.415	3.45E-42	4
CD4	2.30E-46	0.567146348	0.531	0.248	4.75E-42	4
TMSB10	3.22E-46	0.407878229	0.985	0.894	6.63E-42	4
STAM	1.18E-45	0.614407153	0.327	0.123	2.44E-41	4
RUNX3	6.06E-45	0.627005366	0.429	0.183	1.25E-40	4
PIK3IP1	1.01E-44	0.76277553	0.459	0.232	2.08E-40	4
RAB11FIP1	1.76E-44	0.771368231	0.467	0.246	3.63E-40	4
UBB	2.37E-44	0.49159924	0.919	0.857	4.89E-40	4
OXNAD1	3.53E-44	0.502599809	0.28	0.088	7.28E-40	4
GMFG	6.30E-44	0.52216546	0.694	0.414	1.30E-39	4
GLCCI1	1.52E-43	0.620977525	0.338	0.131	3.13E-39	4
ACTB	1.70E-42	0.352608222	0.983	0.943	3.50E-38	4
FUS	5.94E-41	0.572514552	0.76	0.644	1.22E-36	4
CYTOR	1.10E-40	0.707120576	0.58	0.385	2.26E-36	4
ODF2L	1.93E-40	0.505897164	0.318	0.115	3.98E-36	4
PSMB8	3.92E-40	0.602564372	0.635	0.469	8.08E-36	4
MYL6	5.34E-40	0.418117377	0.932	0.85	1.10E-35	4
PSMB9	5.92E-40	0.616586612	0.654	0.458	1.22E-35	4
DUSP5	1.14E-39	0.552438299	0.312	0.112	2.36E-35	4
SON	1.16E-39	0.559807287	0.722	0.62	2.39E-35	4
STAT3	4.49E-39	0.780482763	0.62	0.47	9.25E-35	4
ACTG1	2.45E-38	0.420651585	0.966	0.909	5.05E-34	4
SP140	3.60E-38	0.38448588	0.255	0.08	7.40E-34	4

DEDD2	1.03E-37	0.607849608	0.429	0.219	2.12E-33	4
PPP1R2	1.70E-37	0.873233252	0.535	0.358	3.50E-33	4
RBPJ	1.35E-36	0.823564786	0.558	0.393	2.79E-32	4
RORA	1.88E-36	0.672802076	0.476	0.275	3.86E-32	4
SH3BGRL3	2.52E-36	0.333909537	0.947	0.737	5.18E-32	4
CALM3	1.04E-35	0.496410525	0.682	0.503	2.14E-31	4
SARAF	1.37E-35	0.579936233	0.72	0.632	2.83E-31	4
CFL1	5.14E-35	0.362674216	0.943	0.835	1.06E-30	4
FAU	6.34E-35	0.311913051	0.964	0.9	1.31E-30	4
CD53	1.20E-34	0.461367458	0.662	0.385	2.48E-30	4
MYO1G	3.36E-34	0.48522294	0.338	0.142	6.93E-30	4
RPS29	4.10E-34	0.302179475	0.966	0.923	8.45E-30	4
RSRP1	5.18E-34	0.598733218	0.624	0.477	1.07E-29	4
PNISR	7.21E-34	0.550893947	0.679	0.563	1.49E-29	4
HNRNPA1	1.29E-33	0.364836793	0.885	0.791	2.66E-29	4
GZMM	4.62E-33	0.376571595	0.282	0.096	9.52E-29	4
LPXN	9.05E-33	0.514476598	0.357	0.169	1.86E-28	4
CD48	2.31E-32	0.42392312	0.594	0.342	4.76E-28	4
ANKRD12	3.79E-32	0.541448748	0.671	0.573	7.81E-28	4
CKLF	3.83E-32	0.618571244	0.628	0.51	7.89E-28	4
RCAN3	3.91E-32	0.519735984	0.291	0.12	8.06E-28	4
AC020916.1	7.55E-32	0.555718516	0.335	0.145	1.55E-27	4
ABRACL	9.08E-32	0.491954055	0.49	0.282	1.87E-27	4
RPS4Y1	3.85E-31	0.465120672	0.603	0.376	7.93E-27	4
MIR4435-2HG	4.34E-31	0.598934895	0.41	0.229	8.93E-27	4
CSK	7.40E-31	0.547697052	0.486	0.31	1.52E-26	4
NR3C1	8.88E-31	0.855565209	0.52	0.396	1.83E-26	4
LDHB	9.72E-31	0.472914887	0.544	0.343	2.00E-26	4
ITGAL	1.04E-30	0.567812243	0.399	0.213	2.15E-26	4
CHMP4A	1.09E-30	0.485283762	0.45	0.258	2.25E-26	4
AC058791.1	1.23E-30	0.472268714	0.278	0.112	2.53E-26	4
OAZ1	1.59E-30	0.329851648	0.924	0.794	3.28E-26	4
DNPH1	2.71E-30	0.561835864	0.603	0.482	5.58E-26	4
PRDX2	2.75E-30	0.504369513	0.614	0.484	5.66E-26	4
BTN3A2	2.76E-30	0.559930896	0.437	0.252	5.68E-26	4
PLK3	3.00E-30	0.564805943	0.376	0.187	6.18E-26	4
AKNA	8.03E-30	0.476767871	0.35	0.165	1.65E-25	4
HMGN1	8.42E-30	0.428654849	0.724	0.646	1.73E-25	4
RPL28	8.68E-30	0.251162338	0.989	0.972	1.79E-25	4
IFNG	1.40E-29	0.327997917	0.255	0.087	2.88E-25	4
NFKBIA	1.71E-29	0.526905535	0.743	0.609	3.52E-25	4
AC016831.5	2.07E-29	0.722851314	0.418	0.251	4.26E-25	4
ZFP36L1	9.15E-29	0.436652383	0.775	0.644	1.88E-24	4
MRPL18	1.02E-28	0.646232849	0.603	0.485	2.11E-24	4

HSP90AB1	1.09E-28	0.411941644	0.938	0.878	2.24E-24	4
SRSF5	1.17E-28	0.536707101	0.65	0.562	2.41E-24	4
TAGAP	1.31E-28	0.631697676	0.365	0.177	2.69E-24	4
CELF2	1.32E-28	0.514456892	0.471	0.281	2.73E-24	4
SRRM2	1.52E-28	0.415903447	0.722	0.653	3.12E-24	4
IFI16	2.83E-28	0.440166269	0.539	0.328	5.83E-24	4
ZNF292	4.55E-28	0.70622849	0.495	0.361	9.37E-24	4
FAM129A	5.93E-28	0.49130246	0.321	0.151	1.22E-23	4
HSPH1	6.74E-28	0.355305249	0.711	0.49	1.39E-23	4
ARF6	7.66E-28	0.632261151	0.569	0.467	1.58E-23	4
PARK7	7.83E-28	0.399706491	0.745	0.66	1.61E-23	4
YWHAB	8.27E-28	0.417473892	0.788	0.706	1.70E-23	4
GYPC	8.83E-28	0.407721386	0.465	0.258	1.82E-23	4
DEF6	1.32E-27	0.494570062	0.314	0.149	2.72E-23	4
LAPTM5	1.35E-27	0.308073296	0.756	0.428	2.78E-23	4
PTMA	1.70E-27	0.258190463	0.985	0.952	3.50E-23	4
EMP3	2.16E-27	0.467618888	0.599	0.396	4.44E-23	4
REL	6.01E-27	0.719697756	0.554	0.415	1.24E-22	4
DUSP16	7.03E-27	0.575799491	0.293	0.139	1.45E-22	4
UBE2D2	1.12E-26	0.488370231	0.599	0.505	2.31E-22	4
PLP2	1.28E-26	0.48294753	0.346	0.176	2.64E-22	4
TMSB4X	1.79E-26	0.293958551	0.989	0.813	3.69E-22	4
YWHAZ	2.12E-26	0.500864884	0.656	0.572	4.38E-22	4
COTL1	2.16E-26	0.253833207	0.688	0.393	4.45E-22	4
IKZF1	2.65E-26	0.46769057	0.425	0.238	5.46E-22	4
RASSF5	3.88E-26	0.485933466	0.333	0.164	7.98E-22	4
OCIAD2	6.79E-26	0.500868601	0.367	0.201	1.40E-21	4
IQGAP1	8.28E-26	0.489826373	0.518	0.334	1.70E-21	4
MLLT6	1.29E-25	0.535007817	0.386	0.224	2.66E-21	4
RBM39	2.77E-25	0.403981626	0.728	0.667	5.70E-21	4
SRSF3	4.78E-25	0.431846811	0.713	0.651	9.85E-21	4
CIB1	4.80E-25	0.455957214	0.654	0.583	9.89E-21	4
DNAJB4	7.97E-25	0.664802582	0.425	0.264	1.64E-20	4
VAMP2	1.15E-24	0.511532984	0.563	0.441	2.38E-20	4
BST2	1.50E-24	0.391200784	0.527	0.323	3.10E-20	4
GNG2	1.58E-24	0.440958495	0.335	0.169	3.26E-20	4
POU2F2	2.10E-24	0.521484821	0.314	0.156	4.32E-20	4
PHTF2	2.11E-24	0.568282524	0.323	0.176	4.34E-20	4
STK4	2.52E-24	0.572928606	0.558	0.451	5.19E-20	4
LCP2	5.38E-24	0.414547077	0.423	0.247	1.11E-19	4
RPL36AL	7.55E-24	0.361812527	0.796	0.747	1.55E-19	4
GBP2	8.78E-24	0.596736266	0.544	0.431	1.81E-19	4
TAGLN2	9.70E-24	0.462137658	0.605	0.46	2.00E-19	4
MYL12A	1.68E-23	0.29381536	0.875	0.806	3.46E-19	4
TMC8	3.43E-23	0.422942602	0.28	0.13	7.07E-19	4

CDC42SE2	3.85E-23	0.528286828	0.448	0.305	7.92E-19	4
ATP5IF1	4.48E-23	0.428928433	0.652	0.587	9.22E-19	4
RHOF	5.53E-23	0.395124707	0.274	0.125	1.14E-18	4
EEF1D	9.79E-23	0.309392882	0.89	0.822	2.02E-18	4
ID3	2.42E-22	0.380942159	0.293	0.133	4.99E-18	4
BTG2	7.76E-22	0.445888533	0.577	0.414	1.60E-17	4
ARPC2	1.11E-21	0.305339529	0.832	0.755	2.30E-17	4
ARHGAP9	1.36E-21	0.395574327	0.268	0.126	2.80E-17	4
ISCU	1.67E-21	0.489247388	0.533	0.453	3.43E-17	4
IL10RA	2.08E-21	0.458844135	0.401	0.241	4.28E-17	4
IDS	3.79E-21	0.545374281	0.429	0.285	7.81E-17	4
SEMA4D	6.40E-21	0.400512372	0.251	0.114	1.32E-16	4
APBB1IP	9.72E-21	0.46622261	0.376	0.233	2.00E-16	4
SOCS3	1.31E-20	0.494899602	0.365	0.204	2.70E-16	4
SKP1	1.47E-20	0.369050428	0.707	0.675	3.04E-16	4
BUB3	2.21E-20	0.56447558	0.482	0.384	4.55E-16	4
TERF2IP	4.09E-20	0.548924653	0.505	0.421	8.43E-16	4
HDAC7	5.54E-20	0.411570735	0.259	0.124	1.14E-15	4
IFI27L2	9.65E-20	0.427841135	0.391	0.253	1.99E-15	4
STK17A	1.02E-19	0.463007771	0.386	0.238	2.09E-15	4
HNRNPDL	1.89E-19	0.40322376	0.673	0.641	3.89E-15	4
RAB27A	2.27E-19	0.354194138	0.31	0.165	4.68E-15	4
SLC2A3	3.04E-19	0.363383765	0.439	0.263	6.26E-15	4
ARID4B	3.35E-19	0.492205094	0.518	0.429	6.89E-15	4
PSME2	4.87E-19	0.357760754	0.715	0.637	1.00E-14	4
OPTN	5.42E-19	0.452308768	0.38	0.243	1.12E-14	4
RGS10	6.69E-19	0.308570175	0.446	0.267	1.38E-14	4
CLK1	8.97E-19	0.490999728	0.527	0.419	1.85E-14	4
SNHG8	1.13E-18	0.488014773	0.537	0.457	2.33E-14	4
TUBA4A	1.19E-18	0.391452981	0.471	0.338	2.45E-14	4
NABP1	1.24E-18	0.472970533	0.431	0.291	2.56E-14	4
PSME1	1.31E-18	0.370702792	0.679	0.646	2.70E-14	4
IER2	1.31E-18	0.286029722	0.658	0.489	2.71E-14	4
ATP6V0E1	1.49E-18	0.332445198	0.711	0.644	3.07E-14	4
EPSTI1	2.11E-18	0.433754541	0.306	0.173	4.34E-14	4
SUMO2	2.38E-18	0.254390783	0.822	0.771	4.90E-14	4
LEPROTL1	2.73E-18	0.525599478	0.465	0.367	5.62E-14	4
SELENOW	2.90E-18	0.509641278	0.518	0.43	5.98E-14	4
H2AFZ	3.90E-18	0.332369882	0.682	0.625	8.02E-14	4
ELF1	4.05E-18	0.531082179	0.482	0.384	8.33E-14	4
SAMD9	5.12E-18	0.508368397	0.287	0.157	1.05E-13	4
SERTAD1	6.56E-18	0.480952921	0.38	0.243	1.35E-13	4
PCSK7	9.62E-18	0.50599984	0.427	0.303	1.98E-13	4
UBL5	1.36E-17	0.283380573	0.749	0.705	2.80E-13	4
EIF4A2	1.47E-17	0.340648016	0.735	0.699	3.03E-13	4

ANKRD44	2.21E-17	0.471065855	0.384	0.265	4.55E-13	4
FAM89B	2.57E-17	0.537504915	0.399	0.292	5.28E-13	4
TAP1	7.53E-17	0.464207524	0.399	0.276	1.55E-12	4
CASP1	8.26E-17	0.348600108	0.391	0.249	1.70E-12	4
AHSA1	8.70E-17	0.495351854	0.452	0.353	1.79E-12	4
JAK1	9.15E-17	0.469090213	0.516	0.454	1.88E-12	4
PGAM1	1.03E-16	0.436387192	0.597	0.559	2.12E-12	4
ZNRF1	1.23E-16	0.485984491	0.289	0.172	2.53E-12	4
GALM	1.28E-16	0.531663282	0.427	0.338	2.63E-12	4
METTL8	1.38E-16	0.504527293	0.251	0.137	2.84E-12	4
CHORDC1	2.27E-16	0.510538896	0.439	0.337	4.67E-12	4
EDF1	2.30E-16	0.279165669	0.777	0.745	4.73E-12	4
CYFIP2	2.57E-16	0.374484843	0.251	0.13	5.29E-12	4
AC025164.1	2.66E-16	0.414424653	0.278	0.155	5.49E-12	4
C19orf53	3.94E-16	0.334882996	0.609	0.578	8.12E-12	4
RPS27L	4.96E-16	0.398547446	0.654	0.639	1.02E-11	4
WIPF1	6.25E-16	0.424057913	0.418	0.293	1.29E-11	4
TRIM22	6.32E-16	0.419574227	0.291	0.166	1.30E-11	4
SF3B5	7.12E-16	0.414880418	0.546	0.506	1.47E-11	4
COMMD7	8.08E-16	0.508597753	0.439	0.36	1.66E-11	4
FOXO1	8.43E-16	0.493342088	0.327	0.216	1.74E-11	4
TSC22D4	1.22E-15	0.405299988	0.344	0.225	2.52E-11	4
GRK6	1.95E-15	0.439883219	0.274	0.158	4.01E-11	4
DENND2D	2.35E-15	0.290285132	0.251	0.128	4.84E-11	4
MPHOSPH8	3.80E-15	0.502236987	0.495	0.445	7.82E-11	4
SERPINB9	3.84E-15	0.391837899	0.291	0.168	7.91E-11	4
ENO1	4.57E-15	0.367683962	0.771	0.73	9.41E-11	4
CBLB	5.63E-15	0.459404517	0.287	0.171	1.16E-10	4
PIM3	6.36E-15	0.634531655	0.399	0.309	1.31E-10	4
TRIR	7.59E-15	0.342503538	0.626	0.622	1.56E-10	4
UQCR11	7.77E-15	0.273308685	0.686	0.647	1.60E-10	4
NSMCE3	7.81E-15	0.41301867	0.27	0.158	1.61E-10	4
CDC42	9.95E-15	0.315845581	0.652	0.612	2.05E-10	4
ICA1	1.08E-14	0.643305	0.253	0.149	2.22E-10	4
DOCK8	1.18E-14	0.40526307	0.308	0.191	2.44E-10	4
UBE2D3	1.30E-14	0.289670378	0.662	0.622	2.68E-10	4
EIF5	1.37E-14	0.385835812	0.637	0.612	2.82E-10	4
TSTD1	1.96E-14	0.408325695	0.471	0.397	4.04E-10	4
GPSM3	2.38E-14	0.365578214	0.454	0.325	4.91E-10	4
SH3BGRL	2.52E-14	0.279835558	0.467	0.322	5.19E-10	4
GPBP1	3.90E-14	0.477314715	0.476	0.407	8.03E-10	4
CFLAR	4.27E-14	0.425936515	0.569	0.544	8.80E-10	4
HCLS1	4.30E-14	0.343013829	0.442	0.311	8.86E-10	4
TANK	4.81E-14	0.475951766	0.478	0.432	9.90E-10	4
N4BP2L2	5.15E-14	0.354497545	0.592	0.556	1.06E-09	4

GBP4	5.55E-14	0.334397759	0.297	0.178	1.14E-09	4
SP100	5.80E-14	0.460138922	0.437	0.358	1.19E-09	4
ITGA4	7.63E-14	0.378936479	0.255	0.146	1.57E-09	4
ID2	1.62E-13	0.405558904	0.652	0.603	3.34E-09	4
MYCBP2	1.69E-13	0.538686338	0.429	0.359	3.49E-09	4
MSI2	1.78E-13	0.475197496	0.338	0.242	3.67E-09	4
C16orf87	2.24E-13	0.474974222	0.287	0.19	4.61E-09	4
MAP1LC3B	2.36E-13	0.372705366	0.569	0.524	4.87E-09	4
COX8A	2.46E-13	0.29960623	0.662	0.635	5.06E-09	4
SYNE2	2.55E-13	0.420992742	0.359	0.247	5.26E-09	4
HMGB2	3.56E-13	0.35439353	0.473	0.379	7.33E-09	4
EPC1	4.35E-13	0.466555297	0.418	0.342	8.95E-09	4
PTGES3	6.00E-13	0.279821516	0.667	0.663	1.24E-08	4
PHACTR2	1.10E-12	0.462800041	0.329	0.233	2.27E-08	4
PAXX	1.21E-12	0.437871318	0.403	0.316	2.50E-08	4
NSD3	1.54E-12	0.490600632	0.437	0.378	3.16E-08	4
CCND3	1.77E-12	0.369459979	0.333	0.221	3.64E-08	4
DAZAP2	1.83E-12	0.38140001	0.563	0.534	3.77E-08	4
PRPF38B	2.22E-12	0.472722323	0.518	0.492	4.57E-08	4
AAK1	2.39E-12	0.48803613	0.429	0.357	4.92E-08	4
ANXA6	2.55E-12	0.407291731	0.484	0.439	5.25E-08	4
CSRNP1	2.62E-12	0.42866619	0.308	0.204	5.41E-08	4
ZNF281	2.64E-12	0.57552241	0.327	0.241	5.45E-08	4
SRSF11	2.79E-12	0.305758614	0.597	0.602	5.75E-08	4
ARHGEF1	3.47E-12	0.400102974	0.359	0.259	7.14E-08	4
NFKB2	3.61E-12	0.449544305	0.316	0.218	7.43E-08	4
YPEL5	8.52E-12	0.442977024	0.486	0.432	1.76E-07	4
TRA2B	9.76E-12	0.348664409	0.58	0.546	2.01E-07	4
MXD4	1.53E-11	0.494489917	0.401	0.34	3.15E-07	4
ATRX	1.53E-11	0.422152994	0.512	0.491	3.16E-07	4
AES	1.60E-11	0.36274193	0.573	0.562	3.29E-07	4
HNRNPK	1.84E-11	0.250361538	0.722	0.707	3.79E-07	4
ABHD17A	2.19E-11	0.363356377	0.401	0.312	4.51E-07	4
TRMT112	3.00E-11	0.321203243	0.618	0.632	6.18E-07	4
TAF7	3.09E-11	0.389076123	0.431	0.367	6.37E-07	4
TCF25	3.14E-11	0.383912983	0.529	0.518	6.46E-07	4
HNRNPC	3.45E-11	0.26339491	0.662	0.666	7.11E-07	4
FAM107B	4.23E-11	0.451604342	0.427	0.365	8.72E-07	4
RGS2	5.50E-11	0.305250684	0.493	0.351	1.13E-06	4
FGFR1OP2	5.76E-11	0.387569534	0.346	0.261	1.19E-06	4
PPP1R18	6.61E-11	0.313909514	0.412	0.315	1.36E-06	4
WDR83OS	6.79E-11	0.301406867	0.55	0.535	1.40E-06	4
RBM8A	6.83E-11	0.250725614	0.635	0.621	1.41E-06	4
YME1L1	8.29E-11	0.425328984	0.514	0.476	1.71E-06	4
TSPO	1.09E-10	0.335393538	0.486	0.395	2.24E-06	4

RAP1A	1.32E-10	0.362812057	0.512	0.498	2.72E-06	4
PPP1R12A	1.67E-10	0.438677506	0.456	0.419	3.45E-06	4
ARHGAP30	1.87E-10	0.324356888	0.287	0.191	3.85E-06	4
SEPT6	1.97E-10	0.498160439	0.42	0.374	4.06E-06	4
KMT2E	1.97E-10	0.403185365	0.518	0.513	4.07E-06	4
RILPL2	2.28E-10	0.493500776	0.333	0.253	4.69E-06	4
DDX3X	2.91E-10	0.369538096	0.516	0.489	6.00E-06	4
SF1	3.17E-10	0.450146199	0.505	0.494	6.52E-06	4
RPL22L1	4.40E-10	0.434057937	0.463	0.433	9.06E-06	4
KIF5B	4.67E-10	0.317009362	0.501	0.476	9.62E-06	4
ISG15	5.32E-10	0.481241978	0.484	0.438	1.10E-05	4
SEPT7	6.49E-10	0.27032482	0.571	0.567	1.34E-05	4
KIAA1551	7.21E-10	0.371386116	0.338	0.252	1.48E-05	4
FNBP1	8.14E-10	0.42110695	0.42	0.36	1.68E-05	4
RSRC2	8.19E-10	0.42491335	0.476	0.459	1.69E-05	4
TRAPPC1	8.88E-10	0.359399055	0.503	0.482	1.83E-05	4
MCL1	1.10E-09	0.267243827	0.633	0.619	2.27E-05	4
SFPQ	1.14E-09	0.354508438	0.552	0.551	2.35E-05	4
ARGLU1	1.15E-09	0.416775193	0.463	0.432	2.36E-05	4
REX1BD	1.76E-09	0.356964312	0.456	0.424	3.63E-05	4
RCSD1	1.91E-09	0.319362486	0.259	0.172	3.94E-05	4
PELI1	2.01E-09	0.393118087	0.253	0.173	4.14E-05	4
TLE4	2.74E-09	0.381255457	0.287	0.198	5.64E-05	4
CYCS	2.89E-09	0.346390161	0.611	0.625	5.94E-05	4
NKTR	2.94E-09	0.347786689	0.52	0.506	6.06E-05	4
G3BP2	3.39E-09	0.477398468	0.359	0.302	6.98E-05	4
SMAP2	3.83E-09	0.414764298	0.437	0.402	7.89E-05	4
CDKN1B	4.54E-09	0.434639375	0.363	0.303	9.35E-05	4
GLRX	4.73E-09	0.454358614	0.446	0.41	9.74E-05	4
AKAP13	6.09E-09	0.359927057	0.469	0.443	0.000125371	4
APRT	8.20E-09	0.279426981	0.571	0.573	0.000168913	4
NCKAP1L	8.29E-09	0.278367159	0.321	0.233	0.000170782	4
ZFP36L2	9.02E-09	0.456910172	0.618	0.639	0.000185794	4
MKNK2	1.06E-08	0.341648645	0.403	0.347	0.000217498	4
MRPS6	1.19E-08	0.456030506	0.352	0.296	0.000246055	4
PSMB3	1.42E-08	0.25523293	0.575	0.579	0.000292508	4
CHURC1	1.57E-08	0.400211961	0.444	0.428	0.000324017	4
RBM25	1.61E-08	0.376192825	0.52	0.521	0.000331685	4
JPT1	1.73E-08	0.338613017	0.467	0.432	0.000356459	4
SLTM	1.96E-08	0.403568919	0.429	0.409	0.000404211	4
PHPT1	2.30E-08	0.307766905	0.503	0.513	0.000473633	4
HERPUD1	2.60E-08	0.319085369	0.607	0.596	0.00053623	4
MAP2K3	2.86E-08	0.375371598	0.312	0.24	0.000589556	4
TUBA1A	2.87E-08	0.291729202	0.391	0.296	0.000592041	4
CCNDBP1	3.17E-08	0.425222881	0.335	0.277	0.000652962	4

OTUD5	4.05E-08	0.452306346	0.268	0.199	0.00083499	4
ARPC4	5.46E-08	0.302705637	0.546	0.533	0.001123819	4
COPE	5.76E-08	0.277932716	0.569	0.589	0.001185308	4
UGP2	5.88E-08	0.373238341	0.503	0.506	0.001209995	4
HEXIM1	7.40E-08	0.347349009	0.28	0.206	0.001524564	4
SRSF10	7.92E-08	0.36432171	0.452	0.447	0.001630185	4
AIP	8.32E-08	0.385853835	0.416	0.395	0.001714022	4
BCAS2	1.15E-07	0.460019439	0.401	0.37	0.002360534	4
DDX3Y	1.38E-07	0.298687234	0.314	0.23	0.002849849	4
TNFAIP8	1.39E-07	0.360837616	0.323	0.252	0.002865154	4
RNF166	1.73E-07	0.294204694	0.261	0.189	0.0035642	4
TBCB	1.82E-07	0.370115829	0.412	0.393	0.003755932	4
UBE2S	1.85E-07	0.313630837	0.423	0.362	0.003804737	4
ZNF706	1.92E-07	0.315879135	0.51	0.512	0.003951996	4
PGK1	2.03E-07	0.286766374	0.603	0.616	0.004174132	4
UBXN1	3.02E-07	0.308686083	0.488	0.505	0.00622051	4
CYLD	3.10E-07	0.351446529	0.346	0.288	0.006383608	4
COX17	3.47E-07	0.342990696	0.476	0.483	0.007139329	4
YPEL3	3.58E-07	0.351373214	0.425	0.403	0.007376943	4
SFT2D1	3.62E-07	0.350869083	0.342	0.295	0.007445933	4
MYH9	3.62E-07	0.282071106	0.52	0.528	0.00746211	4
HNRNPM	3.76E-07	0.318200484	0.495	0.515	0.007734366	4
DDX6	4.54E-07	0.459873827	0.355	0.322	0.009344789	4
KRT10	4.89E-07	0.371303116	0.384	0.348	0.010080254	4
BRD2	5.02E-07	0.368890489	0.516	0.536	0.010336651	4
PPP2R5C	5.37E-07	0.368836358	0.423	0.398	0.011061144	4
UBALD2	5.49E-07	0.411235181	0.372	0.333	0.011302485	4
PREX1	5.74E-07	0.366974072	0.265	0.199	0.011831171	4
BAX	7.13E-07	0.451000993	0.433	0.432	0.014691778	4
NDFIP1	8.26E-07	0.352638248	0.439	0.444	0.017009064	4
VMP1	8.55E-07	0.342527075	0.524	0.532	0.017600825	4
NME3	9.35E-07	0.442938071	0.374	0.356	0.019256182	4
GLS	9.57E-07	0.453284369	0.378	0.35	0.019707956	4
RNF19A	1.37E-06	0.412165971	0.325	0.276	0.028227	4
ZFAND2A	1.49E-06	0.347239822	0.439	0.408	0.030733028	4
ERH	1.52E-06	0.262442357	0.51	0.541	0.031328256	4
IFRD1	1.55E-06	0.272252283	0.397	0.344	0.031852095	4
SRSF2	1.68E-06	0.27528488	0.558	0.598	0.034593087	4
RARRES3	1.68E-06	0.278195542	0.372	0.303	0.034664553	4
PSMD8	2.32E-06	0.264402847	0.495	0.516	0.047758885	4
LYST	2.43E-06	0.337144297	0.297	0.239	0.050124332	4
H1FX	5.04E-06	0.353970498	0.51	0.517	0.103816391	4
C4orf48	6.09E-06	0.291912286	0.276	0.217	0.125513336	4
PDCD4	7.16E-06	0.334102432	0.378	0.353	0.147395682	4
EID1	7.25E-06	0.279992844	0.518	0.559	0.149382544	4

MBD2	8.28E-06	0.351407302	0.429	0.438	0.170554324	4
DPP7	8.74E-06	0.2822664	0.346	0.293	0.180007674	4
MAPKAPK3	1.30E-05	0.353549921	0.251	0.204	0.268517964	4
ZFP36	1.31E-05	0.287603121	0.437	0.393	0.269617633	4
SNRPN	1.47E-05	0.314398941	0.41	0.403	0.301877052	4
NEDD9	1.77E-05	0.361928635	0.282	0.237	0.363978208	4
WNK1	2.01E-05	0.341200232	0.45	0.474	0.414200744	4
ARID1B	2.21E-05	0.392068975	0.414	0.418	0.45489033	4
IFI6	2.85E-05	0.352322449	0.331	0.295	0.58739461	4
ZC3HAV1	3.10E-05	0.329169957	0.357	0.338	0.638558919	4
CD84	3.33E-05	0.27151194	0.259	0.198	0.685550513	4
ACTN4	4.20E-05	0.327530767	0.429	0.445	0.865574006	4
SLC3A2	4.63E-05	0.400252797	0.384	0.374	0.954521185	4
SLC5A3	7.06E-05	0.330215139	0.291	0.248	1	4
NCOR1	9.00E-05	0.327409689	0.418	0.437	1	4
NBDY	0.00010366	0.263791072	0.251	0.207	1	4
WDR1	0.000112534	0.278096163	0.42	0.413	1	4
POLR2A	0.000114191	0.336942545	0.291	0.255	1	4
BEX3	0.000120611	0.597055456	0.312	0.296	1	4
ERAP2	0.000125718	0.310529909	0.331	0.307	1	4
NFAT5	0.000129075	0.357468924	0.287	0.258	1	4
HNRNPH3	0.000135551	0.285162707	0.418	0.437	1	4
SRRM1	0.000153373	0.313497387	0.467	0.505	1	4
RSBN1	0.000158529	0.391377445	0.274	0.242	1	4
CLPP	0.000166825	0.375846114	0.293	0.27	1	4
EIF4E	0.000235362	0.382548536	0.335	0.319	1	4
ZNRD1	0.000240163	0.322641249	0.386	0.393	1	4
SMC4	0.000254234	0.275221491	0.314	0.284	1	4
RHBDD2	0.000257436	0.396282568	0.272	0.248	1	4
CMC2	0.000298802	0.302634834	0.304	0.28	1	4
ARPC5L	0.000299655	0.312693783	0.374	0.37	1	4
NDUFB1	0.000335054	0.256966426	0.439	0.466	1	4
RHOG	0.000377065	0.251280629	0.403	0.38	1	4
ARHGAP4	0.000402481	0.259707179	0.28	0.244	1	4
PIM1	0.000550494	0.362590397	0.348	0.344	1	4
CASP4	0.000685031	0.296970353	0.352	0.343	1	4
MAPKAPK2	0.000713752	0.361979444	0.291	0.274	1	4
MBNL1	0.000803777	0.318915128	0.446	0.472	1	4
ARID5A	0.000986124	0.332789506	0.278	0.256	1	4
TAF1D	0.001051723	0.271681787	0.454	0.502	1	4
KDM2A	0.001138891	0.349948038	0.346	0.351	1	4
UBE2N	0.001156219	0.327876863	0.382	0.407	1	4
ARPP19	0.001210608	0.350371099	0.342	0.35	1	4
NOP58	0.001213158	0.291330015	0.365	0.379	1	4
CLDND1	0.001445524	0.354420443	0.282	0.27	1	4

KMT2A	0.001550239	0.375538518	0.342	0.349	1	4
RSBN1L	0.001619923	0.320327881	0.389	0.422	1	4
SEPT9	0.001713226	0.272402532	0.397	0.405	1	4
SP110	0.002097315	0.304903635	0.338	0.339	1	4
SDF4	0.002220015	0.339421587	0.372	0.408	1	4
PSIP1	0.002518886	0.308023795	0.316	0.312	1	4
TMC6	0.002809258	0.319965871	0.31	0.305	1	4
SNHG15	0.002871553	0.285840436	0.259	0.234	1	4
CNN2	0.00319276	0.277274441	0.329	0.32	1	4
DGKZ	0.003272976	0.281657435	0.261	0.234	1	4
CYBC1	0.004701935	0.295139268	0.312	0.314	1	4
CBX3	0.00478643	0.271085257	0.425	0.476	1	4
CAB39	0.005003807	0.330842633	0.316	0.328	1	4
SPTAN1	0.00521311	0.335714596	0.327	0.338	1	4
BCLAF1	0.005390013	0.337789929	0.374	0.4	1	4
CCDC107	0.00549659	0.301791726	0.393	0.431	1	4
SS18L2	0.006010308	0.298523233	0.348	0.37	1	4
STAT1	0.00655642	0.335310338	0.403	0.426	1	4
BPTF	0.006903858	0.308584879	0.393	0.433	1	4
CCM2	0.008045801	0.321542535	0.282	0.28	1	4
DDIT3	0.00884677	0.293683519	0.314	0.309	1	4
VOPP1	0.009027847	0.250793276	0.323	0.324	1	4
CCL5	0	2.583911427	0.962	0.114	0	5
GZMK	0	2.101925653	0.625	0.027	0	5
CD8A	0	2.045301066	0.744	0.015	0	5
GZMA	0	2.021495809	0.841	0.072	0	5
NKG7	0	2.0121059	0.952	0.079	0	5
GZMH	0	1.716547507	0.552	0.039	0	5
TRGC2	0	1.671593197	0.608	0.021	0	5
CD8B	0	1.522441049	0.582	0.009	0	5
CTSW	0	1.369968892	0.691	0.06	0	5
CST7	5.87E-294	1.524544675	0.848	0.138	1.21E-289	5
CD3D	4.41E-286	1.606968174	0.891	0.157	9.09E-282	5
LINC01871	1.35E-258	1.039451076	0.423	0.025	2.79E-254	5
CD3E	8.10E-246	1.274761558	0.818	0.144	1.67E-241	5
EOMES	7.17E-245	0.939348016	0.375	0.019	1.48E-240	5
IFNG	7.20E-234	1.697754431	0.572	0.066	1.48E-229	5
TRAC	5.08E-233	1.326289986	0.876	0.168	1.05E-228	5
PRF1	2.88E-230	1.040551396	0.603	0.072	5.94E-226	5
XCL2	8.51E-226	1.33290823	0.377	0.023	1.75E-221	5
TRBC2	2.67E-205	1.301483769	0.78	0.154	5.49E-201	5
GZMB	4.18E-200	1.088866108	0.471	0.046	8.61E-196	5
CD3G	4.53E-190	1.138374921	0.62	0.103	9.34E-186	5
CD2	6.78E-188	1.201370445	0.742	0.151	1.40E-183	5
KLRD1	4.56E-186	1.005582755	0.418	0.038	9.39E-182	5

CD7	1.16E-180	1.388670011	0.754	0.181	2.39E-176	5
LAG3	1.75E-180	1.028525327	0.418	0.046	3.60E-176	5
GZMM	2.01E-177	0.999903093	0.542	0.08	4.14E-173	5
ITM2A	4.53E-168	1.427359415	0.592	0.111	9.33E-164	5
CD27	3.60E-160	1.11828381	0.567	0.101	7.41E-156	5
CD69	2.92E-159	1.367095761	0.719	0.17	6.01E-155	5
LCK	6.58E-153	0.927737019	0.646	0.132	1.35E-148	5
CCL4L2	5.90E-148	1.402839861	0.765	0.216	1.22E-143	5
IL32	6.82E-143	1.000791659	0.952	0.392	1.40E-138	5
CCL4	7.65E-141	1.064621101	0.749	0.199	1.58E-136	5
ACAP1	3.28E-134	0.964120919	0.623	0.151	6.75E-130	5
PDCD1	1.04E-130	0.836718248	0.306	0.032	2.14E-126	5
SH2D1A	1.24E-125	0.71124692	0.344	0.043	2.55E-121	5
IKZF3	3.35E-125	0.797191514	0.415	0.067	6.89E-121	5
GPR171	4.13E-121	0.687902208	0.314	0.036	8.51E-117	5
TRBC1	8.50E-119	1.40136028	0.527	0.116	1.75E-114	5
HCST	2.70E-115	0.964605544	0.899	0.387	5.56E-111	5
DUSP2	1.48E-114	1.22788023	0.694	0.227	3.04E-110	5
RARRES3	2.51E-112	1.100781057	0.711	0.278	5.16E-108	5
MALAT1	6.78E-110	0.819170792	1	0.95	1.40E-105	5
HLA-C	1.67E-107	0.746721569	0.977	0.867	3.44E-103	5
BTG1	1.92E-107	1.007051124	0.97	0.747	3.95E-103	5
HLA-B	1.32E-106	0.841755475	0.997	0.642	2.71E-102	5
CORO1A	1.80E-105	0.936012218	0.833	0.349	3.70E-101	5
APOBEC3G	1.20E-103	1.003414275	0.565	0.167	2.47E-99	5
TIGIT	1.70E-102	0.869153431	0.468	0.097	3.50E-98	5
HLA-A	2.31E-102	0.693091779	0.99	0.899	4.76E-98	5
HOPX	1.60E-100	0.763893996	0.314	0.044	3.29E-96	5
TNFRSF9	6.32E-99	1.040961126	0.352	0.06	1.30E-94	5
EVL	7.25E-98	0.989701899	0.747	0.334	1.49E-93	5
CLEC2D	4.38E-96	0.914750182	0.59	0.192	9.03E-92	5
PTPRC	7.46E-95	0.867685175	0.858	0.412	1.54E-90	5
JUND	2.18E-93	0.900773525	0.972	0.797	4.48E-89	5
HSPA1B	1.18E-92	0.982997495	0.947	0.611	2.43E-88	5
RAC2	8.82E-92	0.883996795	0.787	0.372	1.82E-87	5
CRTAM	2.11E-91	0.664360877	0.253	0.033	4.34E-87	5
DNAJB1	4.28E-91	1.000841921	0.934	0.605	8.81E-87	5
IL2RG	1.10E-89	0.813711497	0.734	0.311	2.27E-85	5
CD247	5.12E-89	0.577586868	0.524	0.131	1.05E-84	5
RUNX3	2.03E-88	0.931701932	0.549	0.177	4.19E-84	5
CD96	1.96E-87	0.649844357	0.365	0.071	4.03E-83	5
B2M	1.80E-85	0.670966912	1	0.944	3.70E-81	5
LIMD2	1.16E-84	0.886572333	0.711	0.314	2.39E-80	5
HSP90AA1	4.37E-81	0.846911138	0.987	0.885	9.00E-77	5
CD48	8.92E-81	0.878428044	0.724	0.336	1.84E-76	5

PRKCH	1.55E-78	0.726350163	0.405	0.101	3.19E-74	5
STK17A	1.83E-78	0.887890601	0.572	0.226	3.77E-74	5
SRSF7	9.42E-77	1.08256646	0.838	0.645	1.94E-72	5
RGS1	2.51E-76	1.362605772	0.603	0.246	5.16E-72	5
SIRPG	3.84E-76	0.637248447	0.289	0.051	7.91E-72	5
FYN	6.97E-76	0.771871223	0.539	0.179	1.44E-71	5
HLA-E	2.12E-75	0.746497862	0.937	0.728	4.36E-71	5
PYHIN1	2.44E-75	0.637486127	0.306	0.058	5.02E-71	5
HSPA1A	7.56E-72	0.870851222	0.967	0.859	1.56E-67	5
IFITM1	1.20E-71	0.61119936	0.344	0.076	2.48E-67	5
SIT1	9.97E-71	0.632696645	0.359	0.084	2.05E-66	5
RASGRP1	4.65E-70	0.617108709	0.362	0.085	9.57E-66	5
CD6	1.84E-68	0.559715062	0.284	0.054	3.79E-64	5
JUNB	7.35E-65	1.057948108	0.833	0.535	1.51E-60	5
PTPN7	7.40E-63	0.800763931	0.514	0.195	1.52E-58	5
TSC22D3	6.21E-62	0.894314905	0.684	0.339	1.28E-57	5
H3F3B	7.39E-62	0.555047168	0.967	0.876	1.52E-57	5
SYTL3	3.51E-61	0.602524796	0.271	0.056	7.23E-57	5
PMAIP1	8.66E-59	0.826796432	0.6	0.267	1.78E-54	5
JUN	1.80E-58	0.822983925	0.906	0.667	3.70E-54	5
ZAP70	1.85E-58	0.534050388	0.299	0.068	3.82E-54	5
RPS29	2.33E-58	0.472122714	0.987	0.922	4.80E-54	5
TBC1D10C	3.82E-58	0.675782355	0.4	0.123	7.87E-54	5
DDX5	4.71E-58	0.63938372	0.911	0.777	9.71E-54	5
LAT	6.28E-58	0.676464361	0.385	0.115	1.29E-53	5
FOSB	1.35E-57	0.725748751	0.775	0.412	2.78E-53	5
LBH	2.67E-57	0.657666536	0.423	0.134	5.49E-53	5
RAB27A	5.59E-57	0.849845008	0.43	0.158	1.15E-52	5
CCL3L1	1.28E-56	0.703201544	0.537	0.204	2.64E-52	5
RPS26	1.39E-56	0.553763611	0.924	0.831	2.87E-52	5
SLC38A1	4.40E-56	0.621377049	0.352	0.1	9.05E-52	5
DENND2D	4.43E-56	0.735636976	0.38	0.12	9.13E-52	5
CD52	1.56E-55	0.738614365	0.704	0.352	3.21E-51	5
ETS1	1.57E-55	0.571410881	0.354	0.098	3.23E-51	5
ALOX5AP	2.33E-54	0.657135663	0.382	0.116	4.79E-50	5
RHOH	2.03E-53	0.652489968	0.367	0.109	4.17E-49	5
CD37	7.51E-53	0.583675201	0.668	0.336	1.55E-48	5
IL2RB	1.50E-52	0.557829678	0.309	0.079	3.09E-48	5
GATA3	1.91E-52	0.560745333	0.263	0.059	3.93E-48	5
RPS4Y1	7.57E-52	0.600237622	0.694	0.372	1.56E-47	5
FYB1	8.68E-52	0.697031552	0.694	0.355	1.79E-47	5
RGS2	9.99E-52	0.849096938	0.661	0.34	2.06E-47	5
ID2	1.78E-51	0.913803397	0.777	0.594	3.67E-47	5
HSPA8	5.02E-51	0.732072503	0.886	0.736	1.03E-46	5
IRF1	1.46E-50	0.833562481	0.701	0.419	3.01E-46	5

EIF1	1.83E-50	0.44837188	0.977	0.911	3.78E-46	5
DUSP1	2.38E-50	0.799441559	0.856	0.623	4.91E-46	5
CD99	1.51E-49	0.689722588	0.742	0.564	3.12E-45	5
ITGB7	2.34E-49	0.545274242	0.281	0.073	4.82E-45	5
TSPYL2	5.35E-49	0.862320086	0.458	0.187	1.10E-44	5
CLEC2B	1.53E-48	0.765058536	0.595	0.307	3.16E-44	5
RPL23A	1.85E-48	0.41694332	0.977	0.917	3.81E-44	5
SRGN	4.56E-48	0.41394558	0.924	0.507	9.40E-44	5
ISG20	1.79E-47	0.589705762	0.456	0.174	3.69E-43	5
ADGRE5	3.96E-47	0.737519254	0.494	0.236	8.17E-43	5
IER2	5.19E-47	0.733594579	0.762	0.484	1.07E-42	5
S100A4	8.69E-47	0.436603427	0.833	0.432	1.79E-42	5
STK17B	9.69E-47	0.68573604	0.494	0.223	2.00E-42	5
SEPT1	1.26E-45	0.620521386	0.423	0.167	2.59E-41	5
BCL11B	2.40E-45	0.491933004	0.251	0.061	4.95E-41	5
PSTPIP1	2.54E-45	0.57481657	0.319	0.099	5.22E-41	5
ARL4C	2.54E-45	0.599434645	0.668	0.339	5.24E-41	5
PFN1	4.87E-45	0.478742007	0.954	0.816	1.00E-40	5
BTG2	1.20E-44	0.938822291	0.651	0.411	2.46E-40	5
FAU	5.82E-44	0.402546395	0.959	0.901	1.20E-39	5
CALM1	9.77E-42	0.51139602	0.886	0.73	2.01E-37	5
IKZF1	1.29E-41	0.672444525	0.491	0.236	2.66E-37	5
DNAJA1	4.44E-41	0.784655709	0.792	0.698	9.15E-37	5
DUSP4	5.13E-41	0.630433848	0.311	0.096	1.06E-36	5
LSP1	9.30E-40	0.575180961	0.62	0.352	1.92E-35	5
HLA-F	1.10E-39	0.64004853	0.438	0.2	2.27E-35	5
PPP1R15A	2.11E-39	0.716781785	0.696	0.447	4.34E-35	5
CXCR4	3.66E-39	0.80671111	0.59	0.32	7.54E-35	5
RPL41	6.60E-39	0.308246104	0.992	0.981	1.36E-34	5
TRAF3IP3	1.67E-38	0.543983723	0.377	0.146	3.43E-34	5
SKAP1	4.25E-38	0.599665268	0.365	0.142	8.75E-34	5
TAGAP	9.27E-38	0.690847325	0.413	0.176	1.91E-33	5
IFITM2	1.87E-37	0.644476824	0.646	0.448	3.85E-33	5
CYTIP	2.52E-37	0.619979521	0.484	0.234	5.19E-33	5
TMSB4X	9.07E-37	0.407731084	0.995	0.815	1.87E-32	5
UBB	1.17E-36	0.51174358	0.942	0.856	2.42E-32	5
VAMP2	1.26E-36	0.750050477	0.608	0.44	2.59E-32	5
LY6E	3.08E-36	0.716279586	0.557	0.333	6.34E-32	5
SH3BGRL3	4.20E-35	0.367588636	0.952	0.74	8.65E-31	5
PSMB9	6.69E-35	0.617384252	0.651	0.461	1.38E-30	5
UBC	7.98E-35	0.475383807	0.957	0.879	1.64E-30	5
DDX24	5.14E-34	0.688388003	0.585	0.413	1.06E-29	5
BTN3A2	1.08E-33	0.654212756	0.463	0.253	2.22E-29	5
MYL12A	1.71E-33	0.489177507	0.884	0.806	3.51E-29	5
CELF2	2.09E-33	0.651358504	0.499	0.282	4.30E-29	5

CFL1	2.27E-33	0.387580895	0.944	0.836	4.67E-29	5
FOS	2.95E-33	0.620100321	0.729	0.471	6.07E-29	5
RASAL3	4.77E-33	0.463601354	0.286	0.1	9.82E-29	5
RPS12	1.13E-32	0.30769774	0.987	0.968	2.33E-28	5
FUS	1.27E-32	0.59724232	0.727	0.648	2.62E-28	5
CRIP1	2.04E-32	0.507419656	0.319	0.122	4.20E-28	5
PPP2R5C	2.41E-32	0.719501118	0.554	0.388	4.96E-28	5
RPS15A	4.01E-32	0.284757704	0.992	0.963	8.26E-28	5
DUSP5	5.70E-32	0.618772155	0.306	0.116	1.17E-27	5
ITGAL	2.27E-31	0.631439872	0.415	0.215	4.67E-27	5
CCND3	3.42E-31	0.582737166	0.423	0.216	7.04E-27	5
SPN	1.15E-30	0.541604613	0.304	0.119	2.36E-26	5
LAIR2	1.52E-30	0.405218797	0.296	0.1	3.12E-26	5
GIMAP7	4.96E-30	0.593090377	0.415	0.204	1.02E-25	5
RPL19	1.32E-29	0.284237464	0.985	0.944	2.72E-25	5
TUBA4A	1.52E-29	0.80577795	0.516	0.337	3.13E-25	5
GIMAP4	4.25E-29	0.542910541	0.499	0.276	8.75E-25	5
RPL28	6.47E-29	0.263634764	0.992	0.972	1.33E-24	5
ARHGDIB	1.22E-28	0.373980783	0.828	0.491	2.52E-24	5
STK4	1.36E-28	0.691569027	0.585	0.45	2.80E-24	5
CYTOR	1.59E-28	0.651210735	0.557	0.39	3.27E-24	5
RPS3	2.29E-28	0.302593461	0.992	0.959	4.71E-24	5
UBE2S	2.45E-28	0.670667816	0.534	0.355	5.05E-24	5
WIPF1	4.83E-28	0.553816386	0.481	0.29	9.95E-24	5
S100A6	5.08E-28	0.293577559	0.732	0.439	1.05E-23	5
RSRP1	1.11E-27	0.696841105	0.6	0.481	2.28E-23	5
C12orf57	1.28E-27	0.619110852	0.57	0.421	2.64E-23	5
NR4A2	2.40E-27	0.649044216	0.511	0.28	4.95E-23	5
CD81	2.67E-27	0.367570011	0.666	0.393	5.51E-23	5
EZR	3.71E-27	0.577864429	0.456	0.266	7.63E-23	5
AKNA	4.27E-27	0.542422467	0.352	0.168	8.79E-23	5
HSPA6	6.38E-27	1.243184939	0.481	0.282	1.31E-22	5
HSPH1	1.84E-26	0.530769377	0.684	0.496	3.80E-22	5
RPS15	2.06E-26	0.27573158	0.982	0.948	4.23E-22	5
RNF213	2.26E-26	0.643297147	0.603	0.494	4.65E-22	5
COTL1	3.59E-26	0.391741123	0.673	0.399	7.38E-22	5
CHST12	4.74E-26	0.633334231	0.342	0.17	9.76E-22	5
BATF	4.98E-26	0.480464414	0.359	0.165	1.03E-21	5
AC020916.1	7.41E-26	0.532437917	0.332	0.148	1.53E-21	5
IL7R	8.14E-26	0.533356141	0.309	0.122	1.68E-21	5
CD53	3.73E-25	0.410329536	0.651	0.39	7.69E-21	5
PSME1	6.44E-25	0.517113995	0.701	0.645	1.33E-20	5
LYST	8.25E-25	0.748176297	0.397	0.232	1.70E-20	5
SERTAD1	1.16E-24	0.588019174	0.423	0.242	2.39E-20	5
ZFP36L2	1.86E-24	0.588299251	0.711	0.632	3.82E-20	5

CYBA	2.40E-24	0.258550628	0.876	0.517	4.94E-20	5
CCND2	3.36E-24	0.462183207	0.352	0.166	6.91E-20	5
DGKZ	4.33E-24	0.600460046	0.39	0.225	8.93E-20	5
CIRBP	7.25E-24	0.555679597	0.646	0.57	1.49E-19	5
LAPTM5	1.46E-23	0.306918359	0.754	0.433	3.01E-19	5
TMC8	1.04E-22	0.430613823	0.291	0.132	2.14E-18	5
CBLB	2.78E-22	0.572495241	0.329	0.169	5.73E-18	5
NR4A1	1.97E-21	0.51357398	0.494	0.3	4.06E-17	5
CIB1	5.09E-21	0.485040486	0.635	0.585	1.05E-16	5
PAXX	5.38E-21	0.596401833	0.453	0.314	1.11E-16	5
ABHD17A	5.74E-21	0.588950197	0.453	0.309	1.18E-16	5
SLA	6.31E-21	0.613342105	0.395	0.234	1.30E-16	5
RPL36AL	7.38E-21	0.35243689	0.803	0.748	1.52E-16	5
RGCC	7.85E-21	0.615058514	0.38	0.2	1.62E-16	5
SEMA4D	2.48E-20	0.454464556	0.258	0.115	5.11E-16	5
KLF2	3.49E-20	0.812806661	0.468	0.295	7.19E-16	5
LDHB	3.57E-20	0.421876115	0.514	0.348	7.35E-16	5
CACYBP	4.95E-20	0.530291255	0.651	0.592	1.02E-15	5
RBM39	7.59E-20	0.409452513	0.709	0.669	1.56E-15	5
EEF1D	7.97E-20	0.324663492	0.878	0.824	1.64E-15	5
KLF6	1.23E-19	0.441722435	0.734	0.645	2.54E-15	5
KIAA1551	1.64E-19	0.615360341	0.395	0.249	3.38E-15	5
PPP1R18	1.80E-19	0.460589778	0.463	0.313	3.71E-15	5
CMC1	2.02E-19	0.897858424	0.471	0.376	4.16E-15	5
SOCS1	2.23E-19	0.592739137	0.349	0.195	4.60E-15	5
SRSF3	2.39E-19	0.424791573	0.696	0.653	4.92E-15	5
GPR65	2.65E-19	0.496864791	0.291	0.145	5.47E-15	5
GMFG	2.88E-19	0.330969329	0.633	0.423	5.93E-15	5
ARPC2	3.71E-19	0.334477796	0.833	0.756	7.65E-15	5
SEPT7	5.31E-19	0.476881971	0.615	0.564	1.09E-14	5
TERF2IP	5.80E-19	0.579226415	0.514	0.422	1.19E-14	5
SRSF5	6.99E-19	0.528026185	0.61	0.566	1.44E-14	5
ARHGAP9	7.69E-19	0.49102099	0.266	0.129	1.58E-14	5
RASSF5	1.87E-18	0.514221931	0.314	0.168	3.85E-14	5
GPSM3	2.51E-18	0.406871835	0.486	0.324	5.16E-14	5
CYFIP2	5.90E-18	0.41020633	0.268	0.131	1.21E-13	5
PNISR	1.20E-17	0.425259389	0.623	0.569	2.48E-13	5
SRRM2	1.22E-17	0.380113158	0.681	0.657	2.51E-13	5
ITGB2	1.25E-17	0.287018168	0.585	0.348	2.58E-13	5
SUB1	1.38E-17	0.352065428	0.749	0.71	2.84E-13	5
ANAPC16	1.49E-17	0.443816524	0.587	0.534	3.07E-13	5
ANXA1	2.19E-17	0.757745521	0.481	0.325	4.52E-13	5
SH3KBP1	2.30E-17	0.515252391	0.375	0.234	4.73E-13	5
PCSK7	2.41E-17	0.560773755	0.433	0.304	4.97E-13	5
BIN2	2.49E-17	0.468260449	0.301	0.161	5.13E-13	5

RHOF	2.63E-17	0.448705434	0.261	0.128	5.42E-13	5
MSN	2.95E-17	0.415775409	0.552	0.407	6.08E-13	5
ACTG1	3.05E-17	0.281460324	0.965	0.909	6.29E-13	5
AES	4.94E-17	0.453571224	0.608	0.559	1.02E-12	5
EIF4A2	5.27E-17	0.362502688	0.734	0.7	1.08E-12	5
RBM25	6.14E-17	0.530000539	0.575	0.517	1.27E-12	5
SRI	7.73E-17	0.426786933	0.433	0.299	1.59E-12	5
DOK2	1.02E-16	0.446440273	0.478	0.318	2.11E-12	5
LCP2	1.91E-16	0.44552171	0.395	0.252	3.94E-12	5
PPP1R2	3.69E-16	0.673334497	0.466	0.365	7.59E-12	5
GADD45B	3.91E-16	0.583201704	0.597	0.479	8.06E-12	5
IFI16	5.14E-16	0.430796905	0.486	0.334	1.06E-11	5
SON	5.96E-16	0.367472117	0.651	0.627	1.23E-11	5
BIRC3	6.55E-16	0.477485522	0.418	0.252	1.35E-11	5
STAT3	7.02E-16	0.614733537	0.539	0.479	1.45E-11	5
SARAF	7.69E-16	0.382485726	0.663	0.638	1.58E-11	5
GIMAP1	1.36E-15	0.391518519	0.294	0.162	2.80E-11	5
GYPC	1.85E-15	0.338640839	0.418	0.265	3.81E-11	5
HSPE1	3.88E-15	0.364280005	0.851	0.835	7.98E-11	5
HNRNPA2B1	1.74E-14	0.279411682	0.856	0.813	3.58E-10	5
UBL5	2.43E-14	0.289646208	0.737	0.706	4.99E-10	5
YWHAZ	3.28E-14	0.429660525	0.61	0.577	6.75E-10	5
TRA2B	4.28E-14	0.433585897	0.59	0.545	8.82E-10	5
TAF7	5.61E-14	0.565807298	0.448	0.366	1.15E-09	5
SLC2A3	6.30E-14	0.419748819	0.42	0.267	1.30E-09	5
TXNIP	7.19E-14	0.360462758	0.709	0.638	1.48E-09	5
FMNL1	8.43E-14	0.362391356	0.382	0.253	1.74E-09	5
HNRNPDL	9.52E-14	0.375814946	0.656	0.643	1.96E-09	5
APOBEC3C	1.83E-13	0.4572807	0.296	0.175	3.77E-09	5
ARL6IP5	4.30E-13	0.439098737	0.595	0.548	8.85E-09	5
MYO1G	5.15E-13	0.397989903	0.268	0.15	1.06E-08	5
ARHGEF1	6.02E-13	0.477993351	0.37	0.26	1.24E-08	5
ARHGAP30	6.42E-13	0.485788345	0.304	0.191	1.32E-08	5
AC245297.3	9.13E-13	0.500368607	0.41	0.319	1.88E-08	5
PARK7	9.16E-13	0.430721917	0.676	0.666	1.89E-08	5
DEDD2	1.33E-12	0.455710895	0.342	0.228	2.75E-08	5
PSMB8	1.34E-12	0.493532923	0.527	0.479	2.76E-08	5
AKAP13	1.59E-12	0.518772724	0.496	0.442	3.28E-08	5
RNF19A	1.73E-12	0.671469087	0.365	0.274	3.56E-08	5
TRIM22	1.81E-12	0.431357048	0.281	0.169	3.72E-08	5
ABRACL	2.24E-12	0.393265565	0.408	0.291	4.62E-08	5
ANKRD12	2.43E-12	0.375593017	0.6	0.58	5.01E-08	5
CHMP4A	2.69E-12	0.404877451	0.377	0.266	5.55E-08	5
ATF3	4.98E-12	0.553900331	0.418	0.304	1.03E-07	5
TRIR	5.58E-12	0.342765767	0.618	0.622	1.15E-07	5

TNFAIP3	5.74E-12	0.506601669	0.441	0.322	1.18E-07	5
PRKCB	6.16E-12	0.3505744	0.281	0.168	1.27E-07	5
HMGB2	7.14E-12	0.395818987	0.471	0.381	1.47E-07	5
DDIT4	1.13E-11	0.411481454	0.491	0.382	2.33E-07	5
CDC42SE2	2.18E-11	0.440448456	0.403	0.31	4.49E-07	5
H2AFZ	5.28E-11	0.270062073	0.651	0.628	1.09E-06	5
ARF6	1.07E-10	0.512724317	0.499	0.474	2.20E-06	5
H2AFX	1.76E-10	0.409988219	0.334	0.237	3.63E-06	5
ICAM3	1.94E-10	0.405026338	0.365	0.27	4.00E-06	5
CCDC85B	1.99E-10	0.436136201	0.456	0.395	4.11E-06	5
RNF166	2.21E-10	0.397584396	0.286	0.188	4.55E-06	5
MAPRE2	2.31E-10	0.477913265	0.286	0.194	4.76E-06	5
TNFRSF1B	2.38E-10	0.331735915	0.413	0.299	4.90E-06	5
GNG2	2.56E-10	0.380684959	0.278	0.176	5.27E-06	5
AAK1	2.68E-10	0.400481445	0.428	0.358	5.52E-06	5
YPEL5	2.93E-10	0.63938547	0.468	0.434	6.04E-06	5
PRPF38B	4.68E-10	0.500790909	0.504	0.494	9.65E-06	5
DDX3Y	5.81E-10	0.370980261	0.337	0.23	1.20E-05	5
STK10	7.52E-10	0.389264108	0.278	0.185	1.55E-05	5
GBP5	8.39E-10	0.358041898	0.263	0.159	1.73E-05	5
RAP1B	1.61E-09	0.377728765	0.549	0.54	3.32E-05	5
IER5L	1.76E-09	0.530132962	0.415	0.352	3.63E-05	5
PPDPF	2.02E-09	0.378659329	0.481	0.436	4.16E-05	5
GPBP1	2.70E-09	0.475587155	0.448	0.41	5.55E-05	5
LPXN	2.79E-09	0.335580888	0.271	0.178	5.74E-05	5
FXYD5	3.45E-09	0.263228332	0.463	0.355	7.11E-05	5
PDCD4	3.51E-09	0.521846246	0.4	0.352	7.23E-05	5
PLK3	3.63E-09	0.405039206	0.291	0.196	7.49E-05	5
CREM	4.14E-09	0.642603545	0.395	0.33	8.52E-05	5
IDS	5.12E-09	0.456503515	0.372	0.291	0.000105461	5
MBNL1	6.13E-09	0.401334156	0.494	0.468	0.00012634	5
ELF1	6.57E-09	0.470967155	0.43	0.39	0.00013535	5
ARID4B	7.39E-09	0.46750907	0.458	0.435	0.000152271	5
IFRD1	8.88E-09	0.482543009	0.413	0.343	0.000182836	5
GUK1	1.06E-08	0.285643412	0.608	0.638	0.000217398	5
TAF1D	1.18E-08	0.419552642	0.496	0.498	0.000242372	5
DOCK8	1.23E-08	0.37365225	0.281	0.195	0.000254278	5
BRD2	1.84E-08	0.489742782	0.519	0.536	0.000378118	5
TUBA1A	1.99E-08	0.312090559	0.403	0.297	0.000410757	5
MYADM	2.52E-08	0.566003577	0.304	0.226	0.000519378	5
HMGN1	4.03E-08	0.273962148	0.63	0.654	0.000829182	5
CYCS	4.40E-08	0.39999541	0.603	0.626	0.000906187	5
PIK3IP1	4.88E-08	0.435133667	0.324	0.245	0.001004996	5
ATP6V0E1	5.89E-08	0.252891514	0.656	0.649	0.001212096	5
GABARAPL1	6.34E-08	0.397499601	0.268	0.19	0.001306248	5

CD84	6.77E-08	0.337599258	0.286	0.197	0.001395067	5
SRSF2	6.94E-08	0.403817872	0.57	0.596	0.001428964	5
TAP1	7.37E-08	0.403447818	0.349	0.281	0.001518452	5
REL	7.65E-08	0.416180435	0.466	0.424	0.001574653	5
ARPC5L	7.92E-08	0.466502292	0.405	0.367	0.001630557	5
CLK1	8.61E-08	0.443726218	0.461	0.425	0.001774203	5
SYNE2	9.56E-08	0.422254789	0.327	0.251	0.001968408	5
CCNI	1.03E-07	0.306460023	0.628	0.622	0.002122158	5
ZFAND2A	1.46E-07	0.44599057	0.448	0.407	0.002997357	5
HEXIM1	1.74E-07	0.445400296	0.281	0.207	0.003576519	5
PRRC2C	1.89E-07	0.32368236	0.559	0.595	0.003897092	5
ZFP36	2.35E-07	0.341465944	0.463	0.392	0.004844146	5
BIN1	2.80E-07	0.430402996	0.357	0.307	0.005757935	5
SAMSN1	4.08E-07	0.387022404	0.329	0.253	0.008406043	5
OCIAD2	4.45E-07	0.3781971	0.281	0.21	0.00915656	5
TLE4	4.66E-07	0.340852807	0.278	0.2	0.009590847	5
CD44	4.89E-07	0.262507033	0.4	0.313	0.01007962	5
PTTG1	5.27E-07	0.251412286	0.271	0.184	0.010851329	5
DNAJA4	5.62E-07	0.329603777	0.309	0.217	0.011573796	5
DNAJB4	7.38E-07	0.438867702	0.339	0.273	0.015208002	5
TRMT112	7.72E-07	0.307802446	0.582	0.635	0.01589457	5
SF1	9.38E-07	0.39457462	0.481	0.496	0.019308295	5
BUB3	9.52E-07	0.446477584	0.413	0.39	0.019613874	5
IL10RA	1.09E-06	0.260352995	0.329	0.249	0.022458988	5
C9orf16	1.21E-06	0.319984525	0.506	0.51	0.024830523	5
SELENOW	1.41E-06	0.38745594	0.453	0.436	0.029121865	5
WAS	1.42E-06	0.33303416	0.263	0.194	0.029264979	5
SRSF11	1.52E-06	0.326380838	0.554	0.605	0.031335032	5
SNHG8	1.90E-06	0.462534294	0.461	0.464	0.039032652	5
HNRNPF	1.94E-06	0.301940662	0.534	0.576	0.039855801	5
SOCS3	1.95E-06	0.307316772	0.291	0.212	0.040061474	5
EIF5	2.28E-06	0.295629159	0.575	0.617	0.046994734	5
APBB1IP	2.32E-06	0.350519384	0.301	0.241	0.047792165	5
OPTN	2.63E-06	0.400261993	0.309	0.25	0.054196711	5
MBP	4.31E-06	0.327721515	0.256	0.189	0.08880366	5
TUBB4B	4.52E-06	0.310563307	0.572	0.589	0.093135279	5
CSK	5.32E-06	0.345119796	0.365	0.321	0.109667629	5
BST2	5.45E-06	0.283875154	0.405	0.334	0.112308575	5
ISG15	9.33E-06	0.481400334	0.448	0.442	0.192168501	5
AIP	9.77E-06	0.404214404	0.403	0.396	0.201297714	5
RSRC2	1.02E-05	0.420452974	0.443	0.462	0.210211056	5
INTS6	1.22E-05	0.477885545	0.428	0.423	0.251851702	5
POLR2A	1.25E-05	0.503886112	0.299	0.255	0.257520488	5
TSC22D4	1.31E-05	0.343665635	0.286	0.231	0.26970388	5
ZNF331	1.36E-05	0.714900342	0.413	0.398	0.280425121	5

DDIT3	2.24E-05	0.437584415	0.344	0.307	0.462311547	5
KMT2A	2.30E-05	0.434158183	0.365	0.347	0.473315634	5
CSRNP1	2.38E-05	0.357158939	0.268	0.209	0.4910541	5
CNOT6L	4.06E-05	0.392454056	0.271	0.223	0.835509393	5
KRT10	4.22E-05	0.3580905	0.37	0.35	0.870080117	5
KMT2E	4.73E-05	0.323855116	0.478	0.516	0.974783549	5
KDM6B	4.89E-05	0.314600248	0.296	0.239	1	5
SIPA1	4.92E-05	0.346371577	0.258	0.21	1	5
C19orf53	5.70E-05	0.269474021	0.524	0.585	1	5
N4BP2L2	6.16E-05	0.258121838	0.522	0.562	1	5
SF3B5	0.000106139	0.324227252	0.471	0.512	1	5
SNHG15	0.000113308	0.404912178	0.276	0.233	1	5
SUN2	0.000127938	0.362825463	0.314	0.282	1	5
C19orf66	0.00014238	0.401866251	0.284	0.249	1	5
ARGLU1	0.000142542	0.406726106	0.418	0.436	1	5
FAM107B	0.000149412	0.369865822	0.38	0.369	1	5
PHLDA1	0.000167764	0.411953045	0.324	0.284	1	5
ATP5IF1	0.000170411	0.289891677	0.537	0.596	1	5
H1FX	0.000180427	0.409690727	0.489	0.518	1	5
CHORDC1	0.000180477	0.412298973	0.359	0.345	1	5
SIGIRR	0.000181747	0.350111287	0.268	0.227	1	5
NFKBIA	0.000184138	0.352596852	0.595	0.622	1	5
CYLD	0.000184685	0.346511182	0.324	0.29	1	5
MRPL18	0.000187603	0.449442658	0.463	0.497	1	5
WDR1	0.00022065	0.313081745	0.413	0.414	1	5
AHSA1	0.000226539	0.395881404	0.367	0.361	1	5
NKTR	0.000236275	0.347489982	0.466	0.51	1	5
SRRM1	0.000334742	0.344113145	0.458	0.505	1	5
PPP1R12A	0.000431458	0.340894279	0.41	0.423	1	5
MYH9	0.000451088	0.252692229	0.491	0.53	1	5
DAZAP2	0.000471431	0.293347446	0.494	0.539	1	5
CNN2	0.000956481	0.397346202	0.329	0.32	1	5
TAPBP	0.000967762	0.264230435	0.494	0.55	1	5
ATP1B3	0.00096919	0.255309453	0.392	0.374	1	5
CCNL1	0.001129695	0.314718926	0.478	0.533	1	5
SP100	0.001314319	0.344106479	0.362	0.365	1	5
ARID5A	0.001511472	0.348943716	0.278	0.256	1	5
ANKRD13D	0.001602932	0.384377022	0.284	0.264	1	5
ANXA6	0.001878554	0.318516294	0.415	0.445	1	5
CCNDBP1	0.00215519	0.31378415	0.296	0.281	1	5
MKNK2	0.002387847	0.336398248	0.354	0.352	1	5
ACTN4	0.002483017	0.260097862	0.413	0.446	1	5
TRAPPC1	0.002812392	0.311786825	0.435	0.487	1	5
PTPN6	0.002818135	0.321591311	0.311	0.286	1	5
ANKRD44	0.003422869	0.320103125	0.291	0.273	1	5

EIF4A1	0.003469189	0.300816813	0.466	0.522	1	5
NFKB2	0.003633352	0.288645121	0.253	0.225	1	5
PNN	0.003884638	0.34874323	0.433	0.479	1	5
CD82	0.004244348	0.363547035	0.289	0.273	1	5
KRAS	0.004520282	0.371400708	0.284	0.274	1	5
DDX3X	0.004694923	0.279210032	0.453	0.494	1	5
RABAC1	0.005227944	0.289603446	0.428	0.484	1	5
C9orf78	0.005616957	0.390403261	0.347	0.37	1	5
OFD1	0.005648589	0.313442501	0.268	0.254	1	5
WNK1	0.006154597	0.38827488	0.41	0.477	1	5
SLC5A3	0.007936693	0.410168914	0.268	0.25	1	5
WDR83OS	0.008779916	0.267707002	0.461	0.542	1	5
LEPROTL1	0.008898671	0.33775022	0.354	0.376	1	5
SSBP4	0.009568229	0.344938681	0.284	0.279	1	5
AIF1	1.52E-44	1.188340412	0.608	0.297	3.13E-40	6
FTL	2.65E-43	1.214836952	0.993	0.998	5.45E-39	6
C1QB	8.94E-41	1.165030659	0.572	0.26	1.84E-36	6
C1QA	1.23E-40	1.197708651	0.578	0.269	2.52E-36	6
TYROBP	4.87E-30	0.89899244	0.592	0.316	1.00E-25	6
C1QC	4.93E-28	1.017738499	0.441	0.199	1.02E-23	6
TMSB4X	5.47E-28	0.760130251	0.905	0.823	1.13E-23	6
FTH1	3.02E-26	0.907576942	0.951	0.983	6.22E-22	6
HLA-DPB1	3.08E-26	0.737568096	0.634	0.388	6.35E-22	6
HLA-DRA	1.79E-25	0.629677523	0.729	0.514	3.68E-21	6
HLA-DPA1	2.53E-23	0.76571631	0.657	0.447	5.20E-19	6
FCER1G	2.11E-22	0.756956222	0.569	0.324	4.34E-18	6
FABP5	8.99E-22	1.283919242	0.549	0.398	1.85E-17	6
TMSB10	2.31E-20	0.594573027	0.889	0.902	4.75E-16	6
CD74	2.32E-20	0.550366586	0.758	0.619	4.77E-16	6
LGALS3	8.39E-16	1.047212672	0.428	0.288	1.73E-11	6
S100A11	1.04E-13	0.7546795	0.598	0.491	2.14E-09	6
VIM	2.10E-13	0.519198022	0.693	0.575	4.33E-09	6
CD68	3.19E-13	0.959525793	0.464	0.342	6.58E-09	6
CST3	3.71E-13	0.673273443	0.673	0.621	7.64E-09	6
SUGT1	6.53E-12	0.263316556	0.137	0.378	1.34E-07	6
MT-CYB	7.64E-12	0.325163118	1	1	1.57E-07	6
MT-ND3	9.33E-12	0.336604093	0.99	0.999	1.92E-07	6
LGALS1	1.49E-11	0.714852305	0.621	0.594	3.08E-07	6
SAT1	7.49E-11	0.452949575	0.735	0.73	1.54E-06	6
SERF2	1.82E-10	0.418299483	0.807	0.919	3.75E-06	6
MRPL54	2.17E-10	0.31966388	0.144	0.373	4.46E-06	6
MT-ND1	3.35E-10	0.309462575	1	0.999	6.90E-06	6
APOC1	2.08E-09	0.528271611	0.771	0.79	4.28E-05	6
MT-CO3	2.13E-09	0.302866364	1	1	4.40E-05	6
MT-ND2	3.63E-09	0.297780529	0.987	0.999	7.48E-05	6

FUOM	5.10E-09	0.290737221	0.183	0.423	0.000104984	6
MT-CO2	1.09E-08	0.279658576	1	1	0.000224669	6
MT-ND4	2.23E-08	0.284738599	1	1	0.000459028	6
NPC2	2.34E-08	0.744169479	0.559	0.554	0.000481044	6
LAMTOR2	2.40E-08	0.328277583	0.216	0.471	0.00049394	6
RAB5C	2.91E-08	0.268102604	0.248	0.528	0.000600024	6
C9orf16	3.85E-08	0.335979779	0.248	0.525	0.000792189	6
SELENOP	3.99E-08	0.520456836	0.676	0.663	0.000821636	6
ACTB	4.82E-08	0.306079465	0.908	0.948	0.000992301	6
PSMC1	5.22E-08	0.276150216	0.131	0.318	0.001076065	6
SH3BGRL3	7.35E-08	0.565395762	0.699	0.757	0.001513042	6
AKR1A1	8.93E-08	0.322824845	0.222	0.472	0.001839526	6
MS4A7	9.83E-08	0.580868149	0.307	0.203	0.002025287	6
HSBP1	1.04E-07	0.287295935	0.271	0.549	0.002150676	6
SMIM30	1.06E-07	0.346096801	0.147	0.336	0.002187576	6
LAP3	1.49E-07	0.260510546	0.163	0.361	0.003069934	6
FUCA1	2.23E-07	0.271326947	0.15	0.334	0.004597309	6
PPDPF	2.43E-07	0.321158444	0.212	0.452	0.004997749	6
VPS29	2.92E-07	0.30348443	0.265	0.535	0.006020507	6
MT-ND6	3.18E-07	0.279343996	0.18	0.38	0.006555071	6
OAZ2	4.14E-07	0.255332466	0.173	0.368	0.008525452	6
HLA-DQA1	4.35E-07	0.54566328	0.366	0.264	0.008965133	6
YBX1	8.04E-07	0.643138791	0.641	0.803	0.016562801	6
BLOC1S1	8.39E-07	0.305177244	0.268	0.525	0.017273877	6
GLRX	8.67E-07	0.294171268	0.209	0.424	0.017852149	6
NME4	1.26E-06	0.319690249	0.118	0.272	0.025890882	6
LST1	1.30E-06	0.732390565	0.327	0.253	0.026683721	6
OSTF1	1.66E-06	0.296393029	0.176	0.37	0.034286095	6
MS4A6A	1.91E-06	0.513987489	0.337	0.24	0.03925996	6
NTAN1	1.96E-06	0.25483358	0.121	0.273	0.040371577	6
ATP5MPL	2.25E-06	0.271413	0.33	0.647	0.046421835	6
TMEM70	2.74E-06	0.327527913	0.124	0.276	0.056518116	6
AP2S1	4.12E-06	0.291330487	0.294	0.561	0.084840807	6
SERPINB6	5.14E-06	0.299434528	0.147	0.306	0.10586172	6
TUBA1B	5.17E-06	0.571486988	0.598	0.689	0.106421249	6
LYZ	5.48E-06	0.342031584	0.614	0.618	0.112946321	6
MGST3	8.62E-06	0.259699086	0.301	0.572	0.177583187	6
VEGFB	1.04E-05	0.322403712	0.163	0.328	0.213313614	6
PPT1	1.37E-05	0.290562789	0.183	0.36	0.282696381	6
CAPZB	1.99E-05	0.313327083	0.346	0.634	0.410142282	6
TSPO	2.30E-05	0.351768706	0.219	0.413	0.473551793	6
LGMN	3.26E-05	0.643753914	0.376	0.315	0.671878612	6
COX7B	3.80E-05	0.267101218	0.356	0.664	0.783570322	6
SNX2	4.62E-05	0.34431017	0.18	0.347	0.952491129	6
CTSB	4.63E-05	0.479344894	0.588	0.66	0.954219959	6

SLC40A1	5.30E-05	0.836665524	0.392	0.364	1	6
SNX3	5.51E-05	0.37954844	0.324	0.598	1	6
CLTA	6.70E-05	0.260467834	0.363	0.651	1	6
CSTB	8.20E-05	0.862795412	0.539	0.645	1	6
PYCARD	0.000108031	0.302165683	0.183	0.338	1	6
SPI1	0.000134103	0.660169043	0.265	0.214	1	6
CD9	0.000246521	0.261368209	0.16	0.289	1	6
MYL6	0.000289102	0.380053443	0.712	0.865	1	6
PLD3	0.000299121	0.332976316	0.209	0.362	1	6
VAMP5	0.000441021	0.399303792	0.268	0.468	1	6
COX8A	0.00049954	0.263901084	0.373	0.652	1	6
HLA-DRB1	0.000702952	0.377615672	0.405	0.338	1	6
ARRB2	0.000757232	0.298601798	0.167	0.297	1	6
CALM3	0.001304687	0.419912661	0.31	0.53	1	6
H2AFY	0.001622517	0.313768891	0.265	0.449	1	6
SPP1	0.001623805	0.884082627	0.261	0.215	1	6
CYBA	0.001681989	0.35729801	0.562	0.54	1	6
AP1S2	0.002123134	0.28949263	0.157	0.275	1	6
CTSH	0.002318841	0.340286758	0.235	0.39	1	6
IGSF6	0.002509709	0.613124796	0.258	0.214	1	6
SH3BGRL	0.002575629	0.329620411	0.203	0.341	1	6
OAZ1	0.002996895	0.48623786	0.631	0.814	1	6
TPM4	0.003025609	0.294374797	0.235	0.386	1	6
POLR2L	0.003136451	0.316526045	0.382	0.665	1	6
LAPTM5	0.003386502	0.357724085	0.471	0.454	1	6
SMS	0.003739974	0.472689306	0.225	0.378	1	6
FCGR3A	0.006653204	0.34372858	0.278	0.228	1	6
UQCR10	0.006690351	0.265443258	0.415	0.695	1	6
LITAF	0.006703639	0.301217557	0.33	0.524	1	6
HLA-DQA2	0.007105602	0.437151434	0.281	0.241	1	6
HLA-DMA	0.009442714	0.475950235	0.356	0.336	1	6
IL7R	0	2.198410517	0.895	0.094	0	7
LTB	1.59E-250	2.080567388	0.942	0.181	3.27E-246	7
CD40LG	1.43E-249	0.822576016	0.354	0.012	2.95E-245	7
CD3E	8.98E-157	1.022242987	0.796	0.157	1.85E-152	7
CD3D	7.27E-154	0.971608677	0.854	0.172	1.50E-149	7
TRAC	2.84E-147	0.972303619	0.861	0.182	5.85E-143	7
KLRB1	2.70E-146	1.291969964	0.527	0.074	5.55E-142	7
CD69	2.92E-132	1.438617855	0.745	0.179	6.02E-128	7
TRAT1	4.07E-125	0.618982694	0.313	0.027	8.38E-121	7
CD52	4.16E-125	1.296082905	0.912	0.347	8.57E-121	7
CD2	8.49E-125	0.881542764	0.752	0.162	1.75E-120	7
TRBC2	3.59E-119	1.022366229	0.731	0.169	7.40E-115	7
FLT3LG	1.84E-117	0.650162159	0.323	0.033	3.79E-113	7
SPOCK2	2.70E-114	0.788201963	0.605	0.116	5.57E-110	7

IL32	4.89E-114	0.996134819	0.973	0.402	1.01E-109	7
RPS12	1.80E-113	0.933686182	0.993	0.968	3.71E-109	7
RPS29	2.03E-99	0.830198932	0.976	0.924	4.17E-95	7
KLF2	6.07E-99	1.399061902	0.776	0.282	1.25E-94	7
RPL23A	5.30E-96	0.791427179	0.98	0.918	1.09E-91	7
JUNB	6.12E-96	1.283705551	0.935	0.535	1.26E-91	7
S100A4	8.54E-96	1.150039012	0.935	0.434	1.76E-91	7
RPL19	1.84E-95	0.719395471	0.986	0.944	3.80E-91	7
RPL13	1.19E-94	0.648976675	0.993	0.991	2.46E-90	7
RPL28	1.65E-93	0.650797811	1	0.972	3.40E-89	7
BTG1	2.26E-93	1.066437126	0.983	0.75	4.65E-89	7
RPL34	3.24E-90	0.733473051	0.99	0.95	6.67E-86	7
AQP3	4.34E-90	0.856267642	0.378	0.065	8.94E-86	7
RPL41	2.84E-89	0.640944598	0.993	0.981	5.85E-85	7
IL23A	2.03E-88	0.599284708	0.252	0.026	4.19E-84	7
RPS19	2.53E-88	0.705668287	0.993	0.974	5.21E-84	7
FAU	7.52E-88	0.688471294	0.98	0.901	1.55E-83	7
CD48	4.78E-87	1.033601232	0.796	0.339	9.84E-83	7
TPT1	1.90E-86	0.611096459	0.997	0.993	3.92E-82	7
CD3G	1.82E-85	0.807953996	0.524	0.118	3.75E-81	7
RPS3	5.58E-85	0.702641697	0.99	0.96	1.15E-80	7
ACAP1	1.44E-83	0.775337156	0.602	0.161	2.96E-79	7
JUND	8.50E-83	0.964022932	0.973	0.8	1.75E-78	7
RPL10	2.05E-82	0.615167634	0.997	0.986	4.23E-78	7
BCL11B	4.76E-82	0.575671598	0.361	0.058	9.80E-78	7
RPS15	2.10E-80	0.603005091	0.99	0.948	4.34E-76	7
RPS3A	1.24E-78	0.635528004	0.983	0.962	2.55E-74	7
RPS4Y1	2.07E-78	0.956065147	0.803	0.372	4.26E-74	7
HSPA1B	2.17E-78	1.096523103	0.935	0.618	4.48E-74	7
FOSB	3.36E-78	1.007603086	0.884	0.413	6.93E-74	7
FOS	9.32E-78	1.051234612	0.915	0.466	1.92E-73	7
TNFRSF25	1.23E-77	0.58204505	0.344	0.057	2.53E-73	7
RPL18A	1.31E-77	0.603980853	0.99	0.96	2.71E-73	7
RPS28	2.59E-77	0.569745269	0.986	0.94	5.34E-73	7
LCK	3.29E-76	0.716763588	0.568	0.145	6.79E-72	7
RPS14	1.11E-75	0.571161788	1	0.972	2.29E-71	7
ALOX5AP	5.45E-75	0.719767007	0.486	0.116	1.12E-70	7
RPS27A	1.86E-74	0.591760138	1	0.983	3.83E-70	7
RPS15A	3.74E-74	0.587172352	0.997	0.963	7.70E-70	7
S100A6	1.04E-73	0.889668992	0.881	0.437	2.14E-69	7
ANXA1	1.20E-73	1.233626633	0.735	0.315	2.47E-69	7
RPL30	4.45E-72	0.557042441	0.99	0.973	9.17E-68	7
LDHB	1.35E-71	0.932701361	0.728	0.34	2.79E-67	7
GPR171	1.09E-70	0.57042835	0.286	0.043	2.24E-66	7
B2M	3.74E-70	0.713570237	1	0.945	7.70E-66	7

RPS25	2.22E-69	0.585048836	0.969	0.94	4.58E-65	7
RPS26	1.06E-68	0.690698093	0.959	0.831	2.19E-64	7
EMP3	2.06E-68	0.832732858	0.813	0.391	4.25E-64	7
MALAT1	4.36E-68	0.70399107	1	0.951	8.98E-64	7
DNAJB1	1.14E-67	1.049943221	0.915	0.612	2.35E-63	7
DUSP1	5.20E-67	1.02826033	0.922	0.624	1.07E-62	7
GATA3	2.10E-66	0.681453331	0.327	0.06	4.33E-62	7
CALM1	1.28E-65	0.783890896	0.935	0.73	2.63E-61	7
CXCR4	6.71E-65	1.048473262	0.731	0.317	1.38E-60	7
JUN	8.21E-65	0.981125203	0.932	0.67	1.69E-60	7
RPL9	1.15E-64	0.549907968	0.976	0.95	2.37E-60	7
RPL21	2.76E-64	0.563278476	0.986	0.952	5.67E-60	7
CYTIP	1.01E-63	0.779373137	0.626	0.231	2.07E-59	7
RPS27	2.64E-63	0.494117549	0.997	0.989	5.44E-59	7
FXYD5	5.80E-63	0.788319805	0.741	0.342	1.19E-58	7
RPLP2	5.98E-63	0.492414917	0.993	0.961	1.23E-58	7
TRBC1	2.51E-62	0.791697643	0.483	0.125	5.18E-58	7
RPLP1	4.55E-62	0.532483579	0.993	0.986	9.38E-58	7
TCF7	1.15E-61	0.743389972	0.371	0.088	2.36E-57	7
BIRC3	5.76E-61	0.949535333	0.626	0.244	1.19E-56	7
RPS8	2.38E-59	0.528938721	0.99	0.935	4.90E-55	7
IFITM1	6.81E-59	0.606821303	0.361	0.08	1.40E-54	7
RPL36	1.06E-58	0.530405262	0.976	0.917	2.19E-54	7
BTG2	2.32E-58	1.102618992	0.752	0.41	4.77E-54	7
ZFP36L2	6.76E-58	1.049887052	0.844	0.626	1.39E-53	7
EEF1D	6.65E-56	0.587277564	0.952	0.821	1.37E-51	7
RPL11	4.10E-55	0.433741089	0.993	0.973	8.45E-51	7
PPP1R15A	7.22E-55	0.837006643	0.806	0.446	1.49E-50	7
ISG20	2.28E-54	0.694821352	0.527	0.175	4.69E-50	7
RPL13A	5.09E-54	0.475419742	0.99	0.96	1.05E-49	7
TSC22D3	5.65E-54	0.917991135	0.714	0.344	1.16E-49	7
CORO1A	3.06E-53	0.716239336	0.776	0.361	6.30E-49	7
RPL26	3.64E-53	0.468429541	0.983	0.953	7.50E-49	7
TNFAIP3	1.43E-51	1.262434678	0.643	0.313	2.94E-47	7
CD6	1.58E-51	0.506550184	0.289	0.058	3.26E-47	7
RHOH	2.50E-50	0.576835949	0.408	0.112	5.14E-46	7
RPS9	2.55E-50	0.454091955	0.969	0.938	5.24E-46	7
PLP2	8.04E-50	0.762487495	0.48	0.174	1.66E-45	7
HSP90AA1	9.53E-50	0.727443259	0.993	0.887	1.96E-45	7
RPL3	1.04E-49	0.460898086	0.997	0.964	2.13E-45	7
RPS2	1.08E-49	0.430889399	0.99	0.974	2.23E-45	7
CDKN2D	6.91E-49	0.564885548	0.361	0.096	1.42E-44	7
LAT	1.22E-48	0.554752094	0.412	0.118	2.52E-44	7
RPS23	7.83E-48	0.406827036	0.99	0.973	1.61E-43	7
RPL18	9.27E-48	0.433494068	0.997	0.958	1.91E-43	7

HSPA1A	3.41E-47	0.837310155	0.942	0.863	7.03E-43	7
HLA-B	1.03E-46	0.595020261	0.98	0.649	2.12E-42	7
TRAF3IP3	9.67E-46	0.575303046	0.449	0.147	1.99E-41	7
EIF1	2.61E-45	0.536467133	0.963	0.913	5.37E-41	7
DDX5	2.62E-45	0.567442671	0.942	0.777	5.39E-41	7
HSPA8	4.45E-45	0.851526855	0.881	0.739	9.16E-41	7
MYL12A	1.78E-44	0.591863592	0.918	0.805	3.67E-40	7
IRF1	2.55E-44	0.788267658	0.745	0.422	5.25E-40	7
FYB1	3.66E-44	0.63268559	0.748	0.358	7.53E-40	7
EVL	1.23E-43	0.613043696	0.68	0.345	2.53E-39	7
CD37	5.35E-43	0.608317345	0.68	0.342	1.10E-38	7
RPS16	5.80E-43	0.406811393	0.99	0.956	1.19E-38	7
SRSF7	1.59E-42	0.808836171	0.827	0.649	3.27E-38	7
RPL39	1.08E-41	0.327274898	1	0.984	2.22E-37	7
RAC2	2.71E-41	0.559408096	0.735	0.383	5.58E-37	7
H3F3B	3.83E-41	0.529289365	0.993	0.877	7.89E-37	7
RIPOR2	7.33E-41	0.553940826	0.344	0.098	1.51E-36	7
PTPRC	1.07E-40	0.522562403	0.823	0.422	2.20E-36	7
ODF2L	1.76E-40	0.642081728	0.371	0.118	3.62E-36	7
HLA-C	3.41E-40	0.447720288	0.969	0.869	7.03E-36	7
CRIP1	7.24E-40	0.586557857	0.381	0.122	1.49E-35	7
RPS4X	1.44E-39	0.390547908	0.98	0.95	2.96E-35	7
IL2RG	2.66E-39	0.572010507	0.66	0.322	5.49E-35	7
HLA-A	7.15E-39	0.371884442	0.99	0.901	1.47E-34	7
RPSA	9.00E-39	0.434966974	0.983	0.92	1.85E-34	7
SIT1	2.41E-38	0.51800091	0.327	0.091	4.97E-34	7
RPS5	5.48E-38	0.409510033	0.983	0.921	1.13E-33	7
RPL7A	6.62E-38	0.367446126	0.986	0.953	1.36E-33	7
TUBA4A	7.87E-38	0.716972707	0.612	0.335	1.62E-33	7
EEF1A1	1.93E-37	0.296790884	1	0.997	3.98E-33	7
TNFSF14	2.12E-37	0.603175478	0.296	0.083	4.36E-33	7
NR4A1	2.59E-37	0.748044581	0.612	0.297	5.34E-33	7
ARHGAP15	2.89E-37	0.599419551	0.374	0.132	5.95E-33	7
RPL10A	6.28E-37	0.388195358	0.986	0.937	1.29E-32	7
GIMAP7	6.91E-37	0.70992715	0.48	0.204	1.42E-32	7
TBC1D10C	1.17E-36	0.542950008	0.384	0.129	2.40E-32	7
RASGRP1	4.72E-36	0.444987447	0.323	0.093	9.71E-32	7
DNAJA1	7.46E-36	0.746677705	0.84	0.697	1.54E-31	7
TXNIP	8.04E-36	0.649010602	0.844	0.632	1.66E-31	7
ITM2A	8.70E-36	0.459484671	0.405	0.13	1.79E-31	7
AC020916.1	1.02E-35	0.794161472	0.398	0.148	2.11E-31	7
CD96	1.88E-35	0.529775677	0.293	0.081	3.87E-31	7
SLC2A3	2.08E-35	0.66001297	0.565	0.262	4.29E-31	7
AES	2.15E-35	0.684944741	0.718	0.554	4.43E-31	7
IER2	2.43E-35	0.739700904	0.762	0.489	5.01E-31	7

RPL12	3.94E-35	0.350133437	0.983	0.947	8.11E-31	7
RORA	2.51E-34	0.80648348	0.527	0.279	5.17E-30	7
RARRES3	3.00E-34	0.64635226	0.575	0.294	6.18E-30	7
TMSB10	4.77E-34	0.498260657	0.98	0.897	9.83E-30	7
CD247	8.46E-34	0.330304892	0.425	0.143	1.74E-29	7
UBA52	1.71E-33	0.362185391	0.959	0.917	3.52E-29	7
CD44	1.71E-33	0.61112525	0.592	0.304	3.52E-29	7
IKZF1	3.10E-32	0.628869358	0.5	0.24	6.39E-28	7
CLEC2D	3.11E-32	0.573573627	0.473	0.206	6.41E-28	7
GPSM3	3.91E-32	0.565094146	0.602	0.321	8.05E-28	7
RASGRP2	6.33E-32	0.468380856	0.255	0.071	1.30E-27	7
IFITM2	2.05E-31	0.422001504	0.697	0.449	4.22E-27	7
RPS21	3.55E-31	0.35698105	0.973	0.914	7.31E-27	7
HLA-E	7.12E-31	0.51631785	0.922	0.732	1.47E-26	7
RPS6	9.29E-31	0.293350435	0.997	0.973	1.91E-26	7
RPL36AL	1.75E-30	0.460287087	0.871	0.745	3.61E-26	7
UBC	2.59E-30	0.53449318	0.956	0.881	5.34E-26	7
RPL35A	2.60E-30	0.292499778	0.986	0.954	5.36E-26	7
RPL8	2.65E-30	0.298788184	0.99	0.956	5.46E-26	7
SARAF	3.62E-30	0.669944801	0.762	0.633	7.46E-26	7
ABRACL	8.64E-30	0.542665532	0.544	0.286	1.78E-25	7
DDIT4	9.35E-30	0.768675021	0.629	0.377	1.93E-25	7
RCAN3	9.56E-30	0.639936042	0.33	0.124	1.97E-25	7
RPS13	1.63E-29	0.303886063	0.986	0.952	3.36E-25	7
ARL4C	4.06E-29	0.499928747	0.667	0.345	8.36E-25	7
STK17B	9.26E-29	0.575787156	0.476	0.229	1.91E-24	7
VIM	1.88E-28	0.28309265	0.932	0.562	3.87E-24	7
DUSP2	6.95E-28	0.506919379	0.531	0.245	1.43E-23	7
KLF6	1.55E-27	0.693463563	0.789	0.644	3.19E-23	7
TNFAIP8	1.61E-27	0.578679553	0.48	0.246	3.32E-23	7
NFKBIA	2.22E-27	0.747696364	0.759	0.613	4.58E-23	7
SLC38A1	3.86E-27	0.408339924	0.313	0.107	7.95E-23	7
UBE2S	2.04E-26	0.71911536	0.571	0.356	4.20E-22	7
TMSB4X	5.36E-26	0.401725519	0.993	0.818	1.10E-21	7
SEPT1	9.87E-26	0.480195906	0.398	0.173	2.03E-21	7
TAGAP	1.12E-25	0.62485159	0.405	0.181	2.31E-21	7
SNHG8	2.10E-25	0.686937725	0.612	0.456	4.32E-21	7
RPL37	2.92E-25	0.256581091	0.986	0.971	6.01E-21	7
GMFG	3.32E-25	0.459373949	0.694	0.423	6.83E-21	7
NOP53	7.99E-25	0.537423399	0.735	0.617	1.65E-20	7
ARHGDIB	1.71E-24	0.425918488	0.827	0.497	3.52E-20	7
RPS20	2.47E-24	0.309089439	0.963	0.888	5.08E-20	7
LIMD2	2.90E-24	0.547523301	0.561	0.329	5.97E-20	7
PTPN7	2.04E-23	0.564126192	0.432	0.205	4.21E-19	7
PIK3IP1	2.46E-23	0.523735919	0.449	0.24	5.07E-19	7

RPL38	3.40E-23	0.301674339	0.942	0.889	7.00E-19	7
TNF	4.27E-23	0.874538628	0.32	0.131	8.78E-19	7
UBB	4.76E-23	0.528668248	0.908	0.86	9.81E-19	7
AC058791.1	4.84E-23	0.635414633	0.293	0.117	9.96E-19	7
RSRP1	1.26E-22	0.661025907	0.622	0.482	2.59E-18	7
SH3BGRL3	2.12E-22	0.330784955	0.952	0.744	4.36E-18	7
IDS	2.13E-22	0.68140147	0.48	0.286	4.39E-18	7
ANKRD12	5.20E-22	0.571667366	0.67	0.576	1.07E-17	7
ICAM2	8.33E-22	0.487874063	0.344	0.155	1.72E-17	7
GIMAP4	1.20E-21	0.466858736	0.503	0.28	2.46E-17	7
MYADM	1.24E-21	0.656491693	0.412	0.221	2.56E-17	7
BIN2	1.74E-21	0.458797177	0.35	0.16	3.58E-17	7
RGCC	2.46E-21	0.570017812	0.418	0.201	5.06E-17	7
RPL4	3.19E-21	0.344771928	0.915	0.854	6.56E-17	7
MCUB	3.23E-21	0.490366306	0.303	0.131	6.66E-17	7
CCND2	5.58E-21	0.410322241	0.374	0.168	1.15E-16	7
PFDN5	7.48E-21	0.333612794	0.915	0.825	1.54E-16	7
AAK1	9.74E-21	0.623087379	0.52	0.354	2.01E-16	7
CCND3	1.47E-20	0.46189819	0.422	0.22	3.02E-16	7
TOMM7	2.78E-20	0.396710988	0.84	0.744	5.73E-16	7
FYN	3.13E-20	0.381634704	0.408	0.192	6.44E-16	7
GADD45B	3.24E-20	0.593067714	0.67	0.478	6.68E-16	7
ETS1	3.87E-20	0.374190365	0.286	0.107	7.98E-16	7
GZMM	6.33E-20	0.283131492	0.282	0.102	1.30E-15	7
ARL4A	8.03E-20	0.635070587	0.391	0.214	1.65E-15	7
S100A10	1.39E-19	0.357704473	0.861	0.741	2.85E-15	7
SKAP1	1.61E-19	0.44775868	0.33	0.148	3.33E-15	7
PFN1	1.73E-19	0.320770448	0.929	0.82	3.57E-15	7
TAGLN2	3.44E-19	0.453949848	0.65	0.463	7.08E-15	7
CCNI	5.49E-19	0.411539908	0.741	0.615	1.13E-14	7
ZFP36	1.01E-18	0.623809324	0.561	0.388	2.07E-14	7
VAMP2	1.14E-18	0.600912765	0.575	0.445	2.35E-14	7
TLE4	1.34E-18	0.531970504	0.374	0.196	2.77E-14	7
AC016831.5	1.55E-18	0.701149412	0.422	0.256	3.19E-14	7
MYO1G	1.83E-18	0.365908739	0.327	0.149	3.77E-14	7
BCL2	2.98E-18	0.413047533	0.289	0.126	6.14E-14	7
HLA-F	4.39E-18	0.462099416	0.391	0.207	9.05E-14	7
PPP1R2	6.74E-18	0.579361834	0.517	0.364	1.39E-13	7
STK4	1.24E-17	0.61250763	0.571	0.454	2.55E-13	7
C12orf57	2.75E-17	0.417827749	0.575	0.424	5.66E-13	7
SOCS1	2.99E-17	0.57938733	0.367	0.197	6.16E-13	7
EMB	6.07E-17	0.392359363	0.276	0.123	1.25E-12	7
GPR65	7.99E-17	0.470091064	0.306	0.147	1.65E-12	7
KDM6B	2.70E-16	0.489994715	0.405	0.234	5.56E-12	7
CIB1	3.56E-16	0.427395683	0.656	0.585	7.34E-12	7

GIMAP1	3.63E-16	0.42970295	0.323	0.163	7.47E-12	7
CYLD	3.98E-16	0.51797993	0.439	0.285	8.20E-12	7
PCSK7	6.23E-16	0.574188439	0.456	0.305	1.28E-11	7
SOCS3	7.39E-16	0.536855151	0.384	0.209	1.52E-11	7
TSPYL2	8.17E-16	0.461894324	0.371	0.197	1.68E-11	7
SERTAD1	2.62E-15	0.544230582	0.405	0.246	5.40E-11	7
CD53	5.05E-15	0.32628116	0.643	0.395	1.04E-10	7
CACYBP	5.62E-15	0.419665673	0.673	0.592	1.16E-10	7
PLK3	6.11E-15	0.486196311	0.354	0.195	1.26E-10	7
LBH	6.52E-15	0.279174044	0.316	0.145	1.34E-10	7
CDC42SE2	7.02E-15	0.502764193	0.452	0.309	1.45E-10	7
PMAIP1	1.29E-14	0.367056628	0.473	0.28	2.66E-10	7
HSPE1	1.42E-14	0.420220579	0.888	0.833	2.93E-10	7
PSME1	1.44E-14	0.351942102	0.711	0.646	2.97E-10	7
PPP2R5C	2.05E-14	0.564715449	0.507	0.394	4.23E-10	7
STK17A	2.39E-14	0.502314699	0.398	0.242	4.93E-10	7
DDX24	3.07E-14	0.514747776	0.534	0.419	6.33E-10	7
HSPH1	3.86E-14	0.443608065	0.656	0.5	7.95E-10	7
APOL3	5.41E-14	0.372905386	0.252	0.118	1.11E-09	7
SPN	6.49E-14	0.354728919	0.265	0.124	1.34E-09	7
RGS2	8.90E-14	0.317207305	0.565	0.351	1.83E-09	7
PPM1K	2.20E-13	0.464153908	0.265	0.131	4.52E-09	7
LEPROTL1	2.65E-13	0.571052294	0.473	0.369	5.46E-09	7
HNRNPA1	3.12E-13	0.251379488	0.857	0.795	6.43E-09	7
RUNX3	3.72E-13	0.378679732	0.354	0.195	7.67E-09	7
TRADD	5.51E-13	0.506937567	0.34	0.214	1.13E-08	7
FLNA	5.55E-13	0.391684732	0.425	0.269	1.14E-08	7
TGFB1	7.11E-13	0.411195585	0.398	0.251	1.46E-08	7
RHOF	7.59E-13	0.343537982	0.265	0.13	1.56E-08	7
FUS	9.72E-13	0.37565775	0.684	0.652	2.00E-08	7
LY6E	1.06E-12	0.405113996	0.49	0.34	2.19E-08	7
PRMT9	1.25E-12	0.462400567	0.272	0.141	2.58E-08	7
SYNE2	2.49E-12	0.420884121	0.388	0.249	5.13E-08	7
DDX3Y	5.89E-12	0.372279292	0.381	0.229	1.21E-07	7
DUSP5	8.85E-12	0.336002436	0.252	0.122	1.82E-07	7
HNRNPDL	8.87E-12	0.331492343	0.687	0.641	1.83E-07	7
CIRBP	9.61E-12	0.337189611	0.636	0.572	1.98E-07	7
CELF2	1.05E-11	0.35389297	0.442	0.289	2.16E-07	7
YPEL5	1.06E-11	0.599591287	0.51	0.433	2.19E-07	7
CAPN2	1.93E-11	0.320454149	0.272	0.146	3.96E-07	7
PAG1	3.90E-11	0.400521614	0.303	0.175	8.02E-07	7
DOK2	4.03E-11	0.334135144	0.476	0.321	8.29E-07	7
APRT	4.47E-11	0.369596677	0.612	0.571	9.21E-07	7
ZFAS1	6.91E-11	0.274680848	0.673	0.602	1.42E-06	7
ARL6IP5	7.06E-11	0.35908604	0.629	0.547	1.45E-06	7

CYFIP2	8.48E-11	0.317423023	0.255	0.134	1.75E-06	7
GPR183	9.24E-11	0.349818787	0.286	0.156	1.90E-06	7
GSTK1	1.76E-10	0.371047064	0.616	0.583	3.62E-06	7
TRA2B	3.89E-10	0.414679196	0.602	0.546	8.01E-06	7
ICAM3	4.28E-10	0.415469115	0.388	0.271	8.81E-06	7
PNISR	4.72E-10	0.383771363	0.612	0.57	9.72E-06	7
CLEC2B	5.06E-10	0.319973215	0.466	0.319	1.04E-05	7
SF1	6.93E-10	0.40406434	0.537	0.492	1.43E-05	7
CDKN1A	8.82E-10	0.438694719	0.316	0.19	1.82E-05	7
CD55	1.24E-09	0.385588607	0.282	0.17	2.56E-05	7
SRSF5	1.90E-09	0.432092581	0.592	0.568	3.92E-05	7
GTF3A	2.65E-09	0.297423719	0.595	0.559	5.46E-05	7
INTS6	3.33E-09	0.619796196	0.486	0.42	6.86E-05	7
YWHAZ	3.44E-09	0.354312729	0.616	0.577	7.08E-05	7
EIF4A2	3.87E-09	0.276461467	0.718	0.701	7.97E-05	7
AC025164.1	4.45E-09	0.40491637	0.269	0.159	9.16E-05	7
EZR	5.82E-09	0.446873468	0.381	0.273	0.000119792	7
FAM107B	5.83E-09	0.478113884	0.442	0.366	0.000120021	7
CSRNP1	7.65E-09	0.377241054	0.32	0.207	0.000157597	7
NR4A2	8.25E-09	0.360286221	0.432	0.288	0.000169946	7
FMNL1	1.14E-08	0.322133937	0.378	0.255	0.000234263	7
EIF3K	1.22E-08	0.267730228	0.711	0.666	0.000251943	7
RBM39	1.71E-08	0.273424698	0.684	0.671	0.000351354	7
ARHGEF1	2.25E-08	0.425257802	0.361	0.263	0.000462508	7
BRD2	2.51E-08	0.38488806	0.554	0.534	0.000517636	7
ANAPC16	2.67E-08	0.31066763	0.565	0.536	0.000550229	7
CLK1	2.92E-08	0.39157116	0.5	0.424	0.000601666	7
PSMB9	3.05E-08	0.322165231	0.554	0.47	0.000627583	7
PBXIP1	3.43E-08	0.469452045	0.367	0.283	0.0007065	7
STAT3	3.83E-08	0.394786525	0.527	0.48	0.000788486	7
CD99	3.94E-08	0.271172826	0.619	0.574	0.000810899	7
HSPA6	5.28E-08	1.000895122	0.395	0.29	0.001087802	7
CHMP4A	7.46E-08	0.305616272	0.374	0.268	0.001536232	7
ILF3-DT	9.47E-08	0.433401776	0.337	0.244	0.001949986	7
AC245297.3	1.15E-07	0.426526465	0.398	0.321	0.002369366	7
PDCD4	1.26E-07	0.445941922	0.415	0.352	0.00258869	7
NFKB2	1.28E-07	0.379513359	0.316	0.222	0.002640371	7
TUBA1A	2.25E-07	0.438836937	0.422	0.298	0.004631637	7
LMO4	2.67E-07	0.318376556	0.289	0.193	0.005498409	7
UBE2D2	2.69E-07	0.280279774	0.537	0.511	0.005531967	7
TUBB4B	2.74E-07	0.271629066	0.619	0.586	0.005644355	7
ARHGAP45	3.06E-07	0.400921644	0.306	0.22	0.006295056	7
NOSIP	3.45E-07	0.543217985	0.371	0.317	0.007101049	7
GRK6	4.22E-07	0.370149477	0.252	0.163	0.008687346	7
MGAT4A	4.40E-07	0.396697028	0.282	0.202	0.009053108	7

GNG2	9.67E-07	0.267206156	0.276	0.178	0.019912027	7
YME1L1	1.46E-06	0.405853465	0.507	0.478	0.030063717	7
TMEM173	2.25E-06	0.265478837	0.265	0.172	0.046287021	7
ADD3	3.20E-06	0.4063638	0.347	0.282	0.065995144	7
OPTN	3.23E-06	0.341079	0.33	0.25	0.066583189	7
TRIM22	3.24E-06	0.334244127	0.259	0.172	0.066823303	7
OCIAD2	5.31E-06	0.369813963	0.289	0.211	0.109435062	7
TSPO	6.90E-06	0.265523222	0.473	0.399	0.142112388	7
REL	7.45E-06	0.335442018	0.483	0.424	0.153356303	7
RNF213	7.50E-06	0.29827468	0.524	0.5	0.154380845	7
SNHG15	8.33E-06	0.436353609	0.303	0.232	0.171538531	7
MBNL1	9.76E-06	0.321994189	0.5	0.468	0.201028291	7
CITED2	1.00E-05	0.580029609	0.35	0.29	0.206222973	7
NXT1	1.05E-05	0.416271681	0.289	0.223	0.21577686	7
SAMSN1	1.11E-05	0.264946729	0.344	0.253	0.22891825	7
KIAA1551	1.12E-05	0.30339258	0.333	0.255	0.230956986	7
GPBP1	1.24E-05	0.380162387	0.439	0.411	0.254505802	7
MBP	2.59E-05	0.277639821	0.265	0.19	0.533908069	7
MKNK2	2.66E-05	0.372246077	0.398	0.349	0.548091235	7
GLIPR2	2.72E-05	0.266242257	0.252	0.174	0.559667573	7
AKNA	2.88E-05	0.292955922	0.255	0.177	0.593174583	7
HEXIM1	3.86E-05	0.32238761	0.279	0.208	0.795723648	7
AHNAK	7.54E-05	0.357028461	0.517	0.518	1	7
DNAJB4	9.29E-05	0.485286705	0.333	0.274	1	7
SEPT9	0.00012095	0.351077141	0.432	0.403	1	7
NABP1	0.000126	0.389431604	0.357	0.3	1	7
PAXX	0.000127985	0.358248189	0.364	0.321	1	7
POLR2A	0.000129758	0.347364131	0.31	0.255	1	7
IFRD1	0.000138949	0.347330261	0.398	0.345	1	7
EIF4A1	0.000154649	0.313017033	0.503	0.519	1	7
ZFAND2A	0.000163563	0.526676034	0.435	0.409	1	7
APBB1IP	0.000264346	0.282489276	0.299	0.242	1	7
DDX3X	0.000308236	0.357149859	0.49	0.491	1	7
C9orf78	0.000368993	0.39611386	0.381	0.368	1	7
PRPF38B	0.000385985	0.302880319	0.476	0.496	1	7
CYCS	0.000431247	0.312515778	0.599	0.625	1	7
MRPL18	0.000468704	0.384008696	0.486	0.495	1	7
CD47	0.000593973	0.313980015	0.391	0.373	1	7
HNRNPUL1	0.000796177	0.368410746	0.374	0.369	1	7
PSIP1	0.000827541	0.347863774	0.34	0.311	1	7
EIF5	0.000874385	0.251599311	0.571	0.616	1	7
CDC42SE1	0.000945375	0.344899433	0.337	0.307	1	7
FGFR1OP2	0.001017915	0.316283051	0.31	0.266	1	7
ANXA6	0.001413637	0.280372001	0.435	0.443	1	7
DEDD2	0.001413945	0.302608167	0.282	0.234	1	7

CHURC1	0.001823068	0.328378946	0.415	0.43	1	7
DDX21	0.001870981	0.361853496	0.425	0.446	1	7
ISG15	0.001990922	0.318207337	0.446	0.442	1	7
ELF1	0.002087326	0.289888675	0.398	0.392	1	7
LMNA	0.002156341	0.473197756	0.49	0.523	1	7
CCDC107	0.002246902	0.356498184	0.412	0.429	1	7
TAF7	0.002377941	0.328303099	0.381	0.371	1	7
ANKRD44	0.002427361	0.308118672	0.306	0.273	1	7
SIGIRR	0.00250523	0.275737845	0.269	0.228	1	7
FNBP1	0.002579656	0.307828616	0.381	0.365	1	7
DDIT3	0.003032106	0.447432829	0.33	0.308	1	7
CLDND1	0.003060042	0.358336506	0.296	0.269	1	7
SMCHD1	0.003170706	0.405883403	0.344	0.334	1	7
UXT	0.003183553	0.254922049	0.452	0.488	1	7
PIM1	0.003254738	0.313900461	0.357	0.344	1	7
BIN1	0.003777684	0.288550447	0.33	0.309	1	7
CD82	0.003932128	0.273491165	0.306	0.273	1	7
RBL2	0.004285164	0.338359254	0.282	0.258	1	7
CCNL1	0.005733016	0.266158044	0.497	0.531	1	7
ORAI1	0.005993966	0.290701299	0.259	0.225	1	7
CDKN1B	0.006669985	0.349574946	0.323	0.307	1	7
G3BP2	0.00698417	0.319485928	0.32	0.306	1	7
UBALD2	0.007299343	0.306836844	0.344	0.336	1	7
BCAS2	0.007452273	0.261392537	0.378	0.373	1	7
H2AFX	0.008960212	0.271898795	0.276	0.242	1	7
TAF1D	0.009449477	0.318420602	0.452	0.5	1	7
TOP2A	0	1.566037417	0.757	0.07	0	8
PBK	0	0.563124972	0.468	0.016	0	8
UBE2C	8.64E-298	1.466032585	0.678	0.054	1.78E-293	8
PCLAF	1.23E-267	0.961341279	0.622	0.049	2.53E-263	8
MKI67	3.84E-264	1.077995385	0.573	0.04	7.91E-260	8
SPC25	2.82E-256	0.606434555	0.427	0.019	5.81E-252	8
SGO1	6.15E-255	0.590968829	0.494	0.029	1.27E-250	8
ASPM	8.08E-254	0.895426621	0.532	0.036	1.66E-249	8
ZWINT	1.47E-245	0.723039459	0.562	0.043	3.02E-241	8
NUF2	4.65E-244	0.715125692	0.464	0.026	9.58E-240	8
BIRC5	1.65E-242	0.991628687	0.61	0.054	3.40E-238	8
TPX2	6.75E-237	0.931434181	0.577	0.049	1.39E-232	8
CDK1	4.97E-229	0.699836229	0.521	0.038	1.02E-224	8
HJURP	6.89E-225	0.452104369	0.363	0.015	1.42E-220	8
KIF15	1.26E-224	0.364851015	0.367	0.016	2.59E-220	8
MYBL2	2.01E-221	0.555345653	0.431	0.025	4.14E-217	8
KIFC1	2.22E-221	0.576315233	0.506	0.038	4.57E-217	8
CCNA2	5.82E-220	0.572021516	0.401	0.021	1.20E-215	8
MELK	2.48E-215	0.347146252	0.393	0.02	5.10E-211	8

RRM2	6.42E-209	1.085866833	0.547	0.052	1.32E-204	8
AURKB	1.08E-208	0.581680079	0.397	0.022	2.22E-204	8
E2F8	3.79E-207	0.262475914	0.303	0.01	7.81E-203	8
CKAP2L	8.64E-207	0.386614457	0.416	0.025	1.78E-202	8
NCAPG	1.29E-204	0.470715014	0.393	0.022	2.66E-200	8
NUSAP1	1.29E-203	0.99082595	0.599	0.066	2.65E-199	8
CDC20	1.82E-197	0.732008502	0.419	0.028	3.75E-193	8
MAD2L1	2.59E-194	0.61257646	0.551	0.055	5.34E-190	8
CENPF	8.61E-194	0.993600481	0.528	0.051	1.77E-189	8
DLGAP5	4.29E-191	0.424317048	0.337	0.016	8.84E-187	8
TYMS	2.61E-189	1.250548072	0.663	0.094	5.37E-185	8
CENPA	1.57E-188	0.562131749	0.341	0.017	3.23E-184	8
NEK2	2.09E-188	0.458398676	0.326	0.015	4.31E-184	8
CENPM	1.46E-186	0.590822708	0.517	0.05	3.00E-182	8
GTSE1	5.11E-186	0.538425066	0.367	0.022	1.05E-181	8
CDC45	1.04E-184	0.30527426	0.307	0.013	2.15E-180	8
KIF23	9.75E-178	0.454796311	0.33	0.017	2.01E-173	8
NDC80	9.45E-176	0.418284358	0.393	0.027	1.95E-171	8
TCF19	3.70E-175	0.423619196	0.419	0.033	7.61E-171	8
KIF2C	1.25E-171	0.284374043	0.285	0.012	2.58E-167	8
CDKN3	1.07E-170	0.810800496	0.588	0.076	2.20E-166	8
UBE2T	1.93E-169	0.661234452	0.569	0.072	3.97E-165	8
CDCA5	3.51E-168	0.31181055	0.318	0.017	7.22E-164	8
CCNB2	6.54E-166	0.785963619	0.423	0.036	1.35E-161	8
TK1	8.01E-166	0.547102343	0.427	0.037	1.65E-161	8
CDCA3	5.66E-164	0.379368798	0.3	0.015	1.17E-159	8
PLK1	5.85E-162	0.554955714	0.337	0.021	1.20E-157	8
ASF1B	7.63E-156	0.443623103	0.393	0.033	1.57E-151	8
CDT1	3.93E-154	0.456084721	0.464	0.05	8.09E-150	8
DEPDC1	8.05E-151	0.390611336	0.307	0.019	1.66E-146	8
ANLN	1.21E-147	0.290358214	0.318	0.021	2.50E-143	8
SGO2	1.61E-146	0.59616427	0.476	0.057	3.31E-142	8
CENPU	3.87E-146	0.356993513	0.416	0.04	7.96E-142	8
FOXM1	7.22E-145	0.348939868	0.318	0.022	1.49E-140	8
CLSPN	3.64E-143	0.36237694	0.356	0.029	7.50E-139	8
KIF11	4.04E-142	0.328878556	0.311	0.021	8.31E-138	8
TTK	2.80E-139	0.31999816	0.285	0.017	5.76E-135	8
UHRF1	8.05E-139	0.273602038	0.296	0.019	1.66E-134	8
KIF4A	4.16E-137	0.278851329	0.303	0.021	8.57E-133	8
SKA3	1.56E-136	0.261668503	0.273	0.016	3.22E-132	8
CDCA8	2.08E-133	0.431015032	0.292	0.02	4.28E-129	8
GINS2	7.55E-132	0.368830366	0.371	0.035	1.56E-127	8
BUB1	1.84E-131	0.275022447	0.27	0.016	3.78E-127	8
CENPK	2.54E-130	0.390765613	0.438	0.05	5.24E-126	8
CCNB1	1.32E-128	0.832998981	0.427	0.051	2.72E-124	8

C21orf58	5.17E-127	0.315584751	0.367	0.036	1.06E-122	8
HMMR	8.78E-127	0.40376495	0.318	0.026	1.81E-122	8
ORC6	1.48E-125	0.361417167	0.397	0.044	3.04E-121	8
TROAP	5.64E-125	0.319414778	0.288	0.021	1.16E-120	8
E2F1	7.23E-123	0.324354394	0.318	0.028	1.49E-118	8
CHEK1	1.53E-116	0.266626433	0.382	0.043	3.15E-112	8
RAD51AP1	6.40E-116	0.366105567	0.416	0.052	1.32E-111	8
PRC1	2.84E-114	0.355349317	0.345	0.035	5.85E-110	8
ARHGAP11A	8.35E-114	0.376266512	0.363	0.04	1.72E-109	8
FANCI	1.57E-112	0.30653548	0.356	0.039	3.24E-108	8
FEN1	4.49E-112	0.443295897	0.431	0.061	9.24E-108	8
CENPE	1.01E-111	0.479599735	0.337	0.035	2.09E-107	8
TUBB	7.41E-111	1.341940358	0.959	0.674	1.53E-106	8
CKS1B	1.38E-110	1.120852593	0.816	0.298	2.84E-106	8
STMN1	1.98E-108	1.567966308	0.876	0.379	4.07E-104	8
KNL1	6.12E-108	0.337796085	0.39	0.05	1.26E-103	8
DHFR	6.67E-106	0.500633024	0.521	0.097	1.37E-101	8
FAM111B	1.87E-105	0.250160082	0.258	0.02	3.85E-101	8
BRCA1	3.96E-100	0.273718052	0.333	0.039	8.15E-96	8
CENPN	3.60E-98	0.475417561	0.494	0.092	7.41E-94	8
AURKA	5.82E-96	0.456816293	0.375	0.053	1.20E-91	8
HMGB2	1.63E-94	1.408283228	0.861	0.364	3.35E-90	8
HELLS	1.36E-93	0.468472195	0.449	0.079	2.80E-89	8
MCM2	2.00E-92	0.339941653	0.375	0.055	4.12E-88	8
ECT2	9.76E-92	0.250521655	0.333	0.042	2.01E-87	8
CENPH	2.99E-91	0.291634659	0.356	0.048	6.15E-87	8
MXD3	4.26E-90	0.373248792	0.3	0.034	8.77E-86	8
SMC4	1.54E-87	0.907904026	0.738	0.264	3.18E-83	8
RACGAP1	7.86E-85	0.281207647	0.333	0.045	1.62E-80	8
CENPW	3.50E-84	0.480624442	0.442	0.083	7.21E-80	8
ITGB3BP	3.96E-84	0.454691461	0.483	0.101	8.15E-80	8
SMC2	4.11E-84	0.601948644	0.577	0.149	8.47E-80	8
CDCA7	6.85E-83	0.279631078	0.281	0.033	1.41E-78	8
PHF19	7.85E-83	0.484216559	0.532	0.12	1.62E-78	8
DTYMK	1.81E-82	0.731760034	0.667	0.221	3.73E-78	8
CCDC34	4.41E-82	0.479166586	0.506	0.118	9.08E-78	8
BRCA2	1.60E-81	0.259854736	0.367	0.058	3.30E-77	8
FBXO5	9.22E-80	0.254788984	0.307	0.041	1.90E-75	8
MCM4	5.91E-79	0.449044099	0.401	0.075	1.22E-74	8
EZH2	2.72E-77	0.534948112	0.539	0.137	5.60E-73	8
FANCD2	1.26E-76	0.263933258	0.251	0.028	2.60E-72	8
PTTG1	2.35E-74	0.933775505	0.599	0.17	4.85E-70	8
CDCA4	5.24E-74	0.278895573	0.363	0.061	1.08E-69	8
MCM6	3.48E-68	0.495930021	0.416	0.091	7.16E-64	8
PRIM1	3.97E-67	0.252610258	0.356	0.065	8.18E-63	8

TMEM106C	5.14E-67	0.635752636	0.663	0.247	1.06E-62	8
MCM7	1.41E-66	0.750754032	0.607	0.212	2.91E-62	8
ATAD2	8.82E-65	0.465270417	0.558	0.175	1.82E-60	8
GPSM2	2.83E-64	0.277584573	0.315	0.054	5.82E-60	8
NCAPD2	7.33E-64	0.27650661	0.345	0.063	1.51E-59	8
TUBA1B	1.73E-63	1.203028862	0.948	0.671	3.56E-59	8
MIS18A	1.89E-63	0.305632409	0.412	0.094	3.89E-59	8
TACC3	1.88E-62	0.422007439	0.378	0.078	3.87E-58	8
RFC4	8.97E-61	0.287720834	0.386	0.085	1.85E-56	8
TMPO	3.90E-60	0.6251947	0.697	0.278	8.03E-56	8
DNAJC9	1.10E-59	0.443381162	0.491	0.137	2.27E-55	8
RRM1	3.17E-59	0.377607989	0.472	0.127	6.53E-55	8
HMGB1	7.18E-59	0.92270823	0.948	0.833	1.48E-54	8
MCM3	1.42E-58	0.614484172	0.539	0.181	2.93E-54	8
KIF20B	1.57E-58	0.509819088	0.479	0.132	3.23E-54	8
LMNB1	4.70E-58	0.380634395	0.487	0.132	9.67E-54	8
GMNN	9.69E-58	0.593934162	0.64	0.257	2.00E-53	8
C19orf48	1.94E-57	0.295822839	0.461	0.124	4.00E-53	8
WDR34	6.88E-57	0.254589041	0.367	0.082	1.42E-52	8
CKS2	3.49E-56	0.788051679	0.625	0.225	7.18E-52	8
DSN1	1.31E-55	0.28048011	0.375	0.087	2.70E-51	8
LRRC45	5.04E-55	0.27840678	0.412	0.106	1.04E-50	8
SAE1	8.64E-55	0.353270788	0.521	0.156	1.78E-50	8
PCNA	1.90E-54	1.014657161	0.618	0.263	3.92E-50	8
ANP32E	1.87E-52	0.676652922	0.749	0.384	3.85E-48	8
GGH	1.99E-52	0.565466993	0.644	0.264	4.10E-48	8
PSAT1	4.56E-52	0.317568312	0.461	0.131	9.39E-48	8
CTNNAL1	2.79E-51	0.266345292	0.397	0.103	5.75E-47	8
DEK	5.36E-51	0.768986859	0.839	0.581	1.10E-46	8
PHGDH	2.10E-50	0.329863119	0.472	0.145	4.33E-46	8
SNRPD1	7.92E-50	0.659915065	0.779	0.454	1.63E-45	8
RANBP1	1.48E-49	0.793147214	0.813	0.528	3.05E-45	8
LIG1	1.68E-49	0.258719907	0.33	0.073	3.46E-45	8
H2AFZ	4.68E-49	0.992851196	0.88	0.618	9.63E-45	8
MZT1	1.27E-48	0.440662693	0.551	0.2	2.61E-44	8
BARD1	2.28E-48	0.305827016	0.434	0.124	4.70E-44	8
RPA3	3.90E-48	0.65302375	0.734	0.371	8.04E-44	8
FBLN1	7.44E-48	0.310127493	0.427	0.124	1.53E-43	8
LSM5	1.60E-47	0.659912176	0.787	0.449	3.30E-43	8
TUBG1	2.08E-47	0.392815005	0.491	0.17	4.28E-43	8
KNSTRN	6.40E-47	0.253588565	0.281	0.057	1.32E-42	8
TFDP1	6.68E-47	0.315373647	0.468	0.147	1.37E-42	8
CDKN2C	7.51E-47	0.419661546	0.528	0.182	1.55E-42	8
H2AFX	3.99E-46	0.546617449	0.607	0.226	8.22E-42	8
USP1	1.04E-45	0.355157032	0.547	0.205	2.15E-41	8

CDKN2A	4.17E-45	0.50696836	0.61	0.257	8.59E-41	8
JPT2	9.08E-45	0.323928884	0.461	0.149	1.87E-40	8
DBF4	2.63E-44	0.260096457	0.438	0.132	5.42E-40	8
CCHCR1	7.26E-44	0.261967029	0.378	0.103	1.50E-39	8
CENPX	1.17E-43	0.665559308	0.693	0.356	2.41E-39	8
SAC3D1	1.41E-43	0.283468497	0.461	0.151	2.91E-39	8
KPNA2	1.65E-43	0.623252461	0.581	0.234	3.39E-39	8
CDC25B	4.81E-43	0.381101273	0.378	0.103	9.92E-39	8
MGME1	1.13E-42	0.316495161	0.528	0.197	2.33E-38	8
SNRPB	1.44E-42	0.620487081	0.846	0.606	2.96E-38	8
MCM5	2.38E-42	0.415525618	0.464	0.162	4.90E-38	8
KIF22	3.60E-42	0.455554648	0.524	0.2	7.42E-38	8
DKK1	2.74E-41	0.40439112	0.285	0.066	5.64E-37	8
CMSS1	2.93E-41	0.377585384	0.543	0.21	6.04E-37	8
MZT2B	1.52E-40	0.561584437	0.899	0.687	3.13E-36	8
PPIL1	1.52E-40	0.309941946	0.427	0.135	3.14E-36	8
RAN	2.10E-40	0.68422804	0.865	0.649	4.32E-36	8
H2AFV	4.59E-40	0.764373803	0.79	0.532	9.46E-36	8
TUBB4B	6.73E-40	0.73832823	0.846	0.575	1.39E-35	8
GPX2	7.75E-40	0.550011932	0.431	0.144	1.60E-35	8
UNG	1.24E-39	0.281670851	0.382	0.114	2.56E-35	8
MZT2A	1.91E-39	0.546067678	0.865	0.541	3.94E-35	8
SNRPF	4.88E-39	0.559158576	0.805	0.521	1.01E-34	8
TEX30	1.59E-38	0.342302448	0.494	0.195	3.28E-34	8
AKR1C4	4.18E-38	0.715522275	0.625	0.307	8.61E-34	8
FAM111A	6.88E-38	0.254542804	0.472	0.161	1.42E-33	8
PTMA	1.16E-37	0.565826382	0.989	0.953	2.40E-33	8
CKAP2	2.21E-36	0.266913509	0.352	0.101	4.54E-32	8
ALYREF	3.08E-36	0.511031913	0.61	0.294	6.34E-32	8
NUCKS1	3.80E-36	0.621346195	0.82	0.584	7.83E-32	8
HMGN2	4.00E-36	1.102467442	0.843	0.551	8.25E-32	8
NT5DC2	7.95E-36	0.390316533	0.494	0.208	1.64E-31	8
PEG10	1.34E-35	0.846573297	0.648	0.348	2.76E-31	8
SNRNP25	3.21E-35	0.362963649	0.513	0.21	6.61E-31	8
SLC25A10	3.65E-35	0.266966023	0.442	0.161	7.52E-31	8
HIST1H4C	4.82E-35	1.562847574	0.625	0.331	9.93E-31	8
TMEM14A	5.91E-35	0.421474421	0.573	0.258	1.22E-30	8
PAFAH1B3	6.00E-35	0.304548005	0.461	0.169	1.24E-30	8
DUSP9	1.26E-34	0.537961206	0.513	0.21	2.60E-30	8
GSTA1	3.52E-34	0.799522653	0.708	0.416	7.25E-30	8
ACTL6A	4.00E-34	0.304577517	0.498	0.199	8.23E-30	8
SFN	1.11E-33	0.353170509	0.412	0.148	2.28E-29	8
ACAT2	1.78E-33	0.50509669	0.625	0.309	3.66E-29	8
HNRNPA3	3.83E-33	0.548352919	0.85	0.684	7.89E-29	8
ADH4	6.57E-33	0.440789955	0.464	0.184	1.35E-28	8

FDPS	1.39E-32	0.597618579	0.809	0.546	2.86E-28	8
LSM4	3.12E-32	0.460857455	0.708	0.423	6.43E-28	8
RAD21	3.12E-32	0.577818282	0.689	0.412	6.44E-28	8
TXN	5.87E-32	0.536603321	0.914	0.72	1.21E-27	8
TUBA1C	8.76E-32	0.677754182	0.674	0.351	1.80E-27	8
BOLA3	1.21E-31	0.381038952	0.652	0.341	2.50E-27	8
NUDT1	1.22E-31	0.427111998	0.479	0.187	2.52E-27	8
PYCR1	8.28E-31	0.255241818	0.404	0.148	1.70E-26	8
CSE1L	9.56E-31	0.341910077	0.491	0.221	1.97E-26	8
UBE2S	1.45E-30	0.562187145	0.685	0.352	2.99E-26	8
PSIP1	1.55E-30	0.417935637	0.599	0.298	3.19E-26	8
DUT	2.35E-30	0.805995122	0.61	0.333	4.83E-26	8
RANGAP1	4.39E-30	0.303147815	0.401	0.148	9.03E-26	8
LSM3	5.02E-30	0.458913007	0.779	0.514	1.03E-25	8
ELOVL2	6.06E-30	0.330561575	0.412	0.162	1.25E-25	8
UPK3A	6.89E-30	0.386458662	0.453	0.19	1.42E-25	8
CKAP5	8.52E-30	0.332661813	0.375	0.134	1.75E-25	8
AKR1C3	1.55E-29	0.703084793	0.715	0.434	3.19E-25	8
GCHFR	1.67E-29	0.445940474	0.599	0.302	3.44E-25	8
AP1M2	1.76E-29	0.276560667	0.345	0.115	3.63E-25	8
GAMT	4.64E-29	0.538742867	0.652	0.356	9.55E-25	8
PSPH	8.22E-29	0.25127131	0.401	0.15	1.69E-24	8
BAG2	9.61E-29	0.317267531	0.442	0.183	1.98E-24	8
SLC25A1	1.07E-28	0.443917475	0.622	0.334	2.20E-24	8
DKC1	1.76E-28	0.3771324	0.528	0.249	3.62E-24	8
EBP	2.81E-28	0.449993822	0.708	0.408	5.79E-24	8
PGP	3.06E-28	0.337741376	0.577	0.289	6.31E-24	8
NSD2	3.09E-28	0.256238758	0.356	0.122	6.37E-24	8
CPS1	3.10E-28	0.691297916	0.603	0.321	6.39E-24	8
SIVA1	5.44E-28	0.51973995	0.7	0.453	1.12E-23	8
LSM2	6.28E-28	0.449111141	0.693	0.425	1.29E-23	8
HDGF	9.78E-28	0.407481807	0.742	0.438	2.01E-23	8
DNMT1	1.47E-27	0.374489145	0.517	0.238	3.03E-23	8
ISYNA1	2.40E-27	0.270016569	0.352	0.125	4.94E-23	8
SMC3	3.29E-27	0.482454979	0.607	0.34	6.78E-23	8
RBM8A	4.35E-27	0.495155712	0.813	0.613	8.97E-23	8
DHCR24	1.09E-26	0.471154719	0.596	0.301	2.25E-22	8
ANP32B	1.25E-26	0.54512279	0.783	0.562	2.57E-22	8
ANAPC11	1.36E-26	0.443914738	0.798	0.542	2.79E-22	8
LBR	1.46E-26	0.418106793	0.618	0.33	3.01E-22	8
LECT2	1.73E-26	0.336068755	0.457	0.2	3.56E-22	8
SORD	2.22E-26	0.463807483	0.562	0.298	4.57E-22	8
SUPT16H	2.71E-26	0.298774274	0.536	0.271	5.58E-22	8
CDK4	2.75E-26	0.397232786	0.539	0.276	5.66E-22	8
SQLE	3.08E-26	0.458575415	0.543	0.264	6.35E-22	8

TMEM97	3.34E-26	0.338044541	0.487	0.228	6.88E-22	8
NCL	5.42E-26	0.510451512	0.816	0.61	1.12E-21	8
PCYT2	6.40E-26	0.370099588	0.577	0.299	1.32E-21	8
RHEB	1.08E-25	0.50684225	0.742	0.504	2.23E-21	8
SNRPC	1.22E-25	0.480524122	0.749	0.491	2.52E-21	8
AGT	1.61E-25	0.654650711	0.813	0.625	3.31E-21	8
JPT1	3.49E-25	0.676043852	0.697	0.422	7.18E-21	8
ILF2	4.10E-25	0.370601824	0.685	0.418	8.44E-21	8
SLC2A4RG	4.32E-25	0.325715243	0.596	0.31	8.90E-21	8
MDK	5.68E-25	0.44351758	0.562	0.294	1.17E-20	8
SLC2A2	8.69E-25	0.465448386	0.521	0.261	1.79E-20	8
IPO9	8.83E-25	0.255209311	0.449	0.196	1.82E-20	8
GLO1	9.09E-25	0.4632153	0.678	0.437	1.87E-20	8
PSMD14	9.17E-25	0.343289461	0.603	0.324	1.89E-20	8
MRPS26	9.32E-25	0.408235434	0.663	0.378	1.92E-20	8
CCDC167	1.01E-24	0.372900776	0.603	0.318	2.08E-20	8
DBI	1.14E-24	0.465585004	0.914	0.769	2.34E-20	8
PTGES3	1.32E-24	0.409099324	0.865	0.654	2.72E-20	8
BHMT	1.37E-24	0.327963064	0.412	0.178	2.83E-20	8
HNRNPAB	1.39E-24	0.454967508	0.622	0.341	2.87E-20	8
GGCT	1.93E-24	0.277917794	0.491	0.236	3.98E-20	8
HMGN1	2.30E-24	0.473416475	0.824	0.644	4.73E-20	8
SNRPD3	3.29E-24	0.401648037	0.764	0.541	6.78E-20	8
UQCC2	3.54E-24	0.348187993	0.663	0.398	7.29E-20	8
EXOSC8	4.16E-24	0.294419469	0.457	0.203	8.57E-20	8
CBX1	4.25E-24	0.291764388	0.468	0.218	8.75E-20	8
NASP	4.32E-24	0.442347735	0.625	0.361	8.90E-20	8
COMMD4	5.09E-24	0.315288085	0.566	0.297	1.05E-19	8
ODC1	5.58E-24	0.323494313	0.566	0.285	1.15E-19	8
TF	6.09E-24	0.544460563	0.76	0.509	1.26E-19	8
AKR1C2	6.86E-24	0.445262174	0.678	0.381	1.41E-19	8
CARHSP1	1.01E-23	0.371070499	0.652	0.369	2.08E-19	8
SNRPA	1.31E-23	0.258134728	0.536	0.264	2.69E-19	8
IGF2BP3	2.55E-23	0.272898091	0.39	0.166	5.26E-19	8
PSMC3	2.96E-23	0.299720739	0.64	0.371	6.09E-19	8
GPC3	4.52E-23	0.591420299	0.85	0.685	9.30E-19	8
CALM2	4.65E-23	0.505884262	0.929	0.757	9.57E-19	8
PDAP1	5.16E-23	0.332526659	0.685	0.43	1.06E-18	8
ALDH4A1	9.16E-23	0.332792049	0.491	0.24	1.89E-18	8
VIL1	9.30E-23	0.259040838	0.498	0.237	1.92E-18	8
NAP1L4	9.32E-23	0.321641585	0.603	0.338	1.92E-18	8
ATP5MF	1.04E-22	0.361872761	0.891	0.714	2.15E-18	8
BUB3	1.20E-22	0.389276083	0.652	0.379	2.46E-18	8
SNRPE	1.49E-22	0.374066056	0.753	0.505	3.08E-18	8
NME1	1.60E-22	0.416374019	0.663	0.413	3.30E-18	8

CCT3	1.86E-22	0.37067209	0.757	0.535	3.83E-18	8
SMARCA1	1.87E-22	0.334959999	0.457	0.22	3.86E-18	8
POLE3	2.17E-22	0.257221077	0.517	0.253	4.47E-18	8
FAH	2.33E-22	0.289848582	0.502	0.238	4.80E-18	8
SRPK1	2.53E-22	0.297319674	0.547	0.287	5.22E-18	8
POLD2	3.80E-22	0.304240976	0.528	0.278	7.84E-18	8
FADS1	3.89E-22	0.337479818	0.427	0.199	8.01E-18	8
SKA2	5.17E-22	0.281933517	0.393	0.162	1.07E-17	8
CHD1L	5.30E-22	0.310440723	0.506	0.268	1.09E-17	8
ATP5MC1	5.35E-22	0.459115406	0.715	0.506	1.10E-17	8
HSPD1	6.49E-22	0.371021672	0.914	0.815	1.34E-17	8
FGL1	7.17E-22	0.5087477	0.903	0.759	1.48E-17	8
H1FX	7.58E-22	0.437068759	0.753	0.505	1.56E-17	8
DCXR	1.10E-21	0.355433305	0.813	0.531	2.27E-17	8
AADAC	1.20E-21	0.449135893	0.599	0.34	2.47E-17	8
VDAC3	4.66E-21	0.30140616	0.663	0.4	9.60E-17	8
FABP1	5.24E-21	0.52819659	0.888	0.729	1.08E-16	8
ITIH2	5.96E-21	0.515605593	0.629	0.381	1.23E-16	8
CHCHD2	6.80E-21	0.384854146	0.906	0.8	1.40E-16	8
SEM1	7.05E-21	0.362978141	0.82	0.628	1.45E-16	8
PPM1G	8.82E-21	0.327296411	0.652	0.417	1.82E-16	8
MAZ	9.60E-21	0.272881538	0.633	0.362	1.98E-16	8
DDT	9.90E-21	0.454121099	0.835	0.645	2.04E-16	8
ARPC1A	1.30E-20	0.375722418	0.667	0.415	2.68E-16	8
BANF1	1.83E-20	0.430173988	0.742	0.503	3.78E-16	8
TMUB1	2.11E-20	0.28557816	0.558	0.299	4.34E-16	8
SNRPG	2.83E-20	0.436125858	0.783	0.589	5.83E-16	8
MEG8	3.32E-20	0.457290351	0.401	0.182	6.84E-16	8
PAQR9	3.61E-20	0.276441043	0.382	0.175	7.44E-16	8
CBX3	3.63E-20	0.335968884	0.697	0.461	7.48E-16	8
UGDH	4.05E-20	0.327884336	0.536	0.291	8.34E-16	8
PSMB2	4.17E-20	0.360131477	0.719	0.487	8.60E-16	8
MSMO1	4.51E-20	0.369487143	0.562	0.305	9.28E-16	8
CCT6A	4.73E-20	0.385640202	0.685	0.475	9.74E-16	8
BHMT2	5.04E-20	0.331513013	0.479	0.244	1.04E-15	8
TMEM245	5.24E-20	0.257777268	0.513	0.267	1.08E-15	8
UGT2B7	5.71E-20	0.301914638	0.375	0.168	1.18E-15	8
RPSA	5.98E-20	0.314506756	0.974	0.921	1.23E-15	8
RNASEH2C	6.14E-20	0.27575683	0.622	0.366	1.26E-15	8
ALDH7A1	6.33E-20	0.279048529	0.464	0.229	1.30E-15	8
SET	6.60E-20	0.386378726	0.835	0.657	1.36E-15	8
PRPSAP1	8.80E-20	0.281791055	0.551	0.294	1.81E-15	8
YWHAQ	8.86E-20	0.383695011	0.749	0.543	1.83E-15	8
TMEM14B	9.42E-20	0.325255731	0.708	0.471	1.94E-15	8
PSMD2	9.57E-20	0.30629781	0.588	0.351	1.97E-15	8

NR2F6	9.76E-20	0.305939307	0.509	0.269	2.01E-15	8
HLTF	1.10E-19	0.253115171	0.401	0.187	2.26E-15	8
SPATS2L	1.41E-19	0.37573167	0.667	0.433	2.90E-15	8
XRCC5	1.41E-19	0.375589615	0.723	0.55	2.90E-15	8
UGT1A1	3.39E-19	0.312154817	0.416	0.198	6.99E-15	8
FDFT1	3.49E-19	0.285769118	0.584	0.342	7.18E-15	8
FASN	4.50E-19	0.448741563	0.524	0.301	9.27E-15	8
MRPL11	4.50E-19	0.288888859	0.618	0.366	9.27E-15	8
PDZK1	4.93E-19	0.288090027	0.468	0.229	1.02E-14	8
POLR2J	5.35E-19	0.346526312	0.734	0.496	1.10E-14	8
TPRKB	9.93E-19	0.269393466	0.558	0.32	2.04E-14	8
COPS6	1.08E-18	0.289766494	0.693	0.454	2.23E-14	8
HNRNPR	1.12E-18	0.336557725	0.667	0.434	2.31E-14	8
APOA2	1.21E-18	0.510474916	1	0.998	2.49E-14	8
ECHS1	1.56E-18	0.378439349	0.753	0.536	3.20E-14	8
POP7	1.59E-18	0.273727547	0.502	0.28	3.28E-14	8
APOH	1.77E-18	0.40930432	0.869	0.697	3.64E-14	8
DDX39A	1.77E-18	0.27826945	0.483	0.242	3.65E-14	8
RNF5	1.82E-18	0.358159962	0.648	0.387	3.75E-14	8
HMGCS1	2.00E-18	0.2831692	0.547	0.299	4.13E-14	8
RPLP0	2.20E-18	0.335143792	0.963	0.889	4.54E-14	8
PON2	2.83E-18	0.397460853	0.592	0.373	5.82E-14	8
MAD2L2	2.98E-18	0.267929531	0.401	0.186	6.13E-14	8
FH	3.28E-18	0.282595555	0.517	0.289	6.75E-14	8
MPC2	3.61E-18	0.383024254	0.805	0.572	7.44E-14	8
SUCLG1	4.33E-18	0.343796507	0.67	0.443	8.93E-14	8
APOA1	4.39E-18	0.421875711	0.963	0.898	9.04E-14	8
COX20	4.69E-18	0.284346347	0.629	0.392	9.65E-14	8
SMC1A	4.93E-18	0.278110778	0.464	0.25	1.02E-13	8
CDK2AP2	5.56E-18	0.303955994	0.648	0.388	1.14E-13	8
APOM	5.69E-18	0.428545445	0.854	0.684	1.17E-13	8
PSMD1	7.23E-18	0.318976332	0.599	0.372	1.49E-13	8
PRKDC	7.77E-18	0.306300598	0.513	0.302	1.60E-13	8
RBMX	8.13E-18	0.327759374	0.689	0.439	1.67E-13	8
MRPL13	8.57E-18	0.292245553	0.577	0.341	1.76E-13	8
RRP7A	1.00E-17	0.321048099	0.625	0.386	2.07E-13	8
GCSH	1.09E-17	0.342974815	0.633	0.391	2.24E-13	8
KRT8	1.21E-17	0.381259025	0.599	0.347	2.49E-13	8
SLBP	1.46E-17	0.278613101	0.498	0.263	3.02E-13	8
ARL6IP1	1.56E-17	0.709675844	0.76	0.55	3.21E-13	8
SERBP1	1.58E-17	0.324586543	0.805	0.634	3.26E-13	8
NONO	1.70E-17	0.297307341	0.704	0.479	3.51E-13	8
HSPB11	1.81E-17	0.2688128	0.536	0.309	3.72E-13	8
NDUFC2	1.86E-17	0.324004855	0.828	0.65	3.83E-13	8
SRSF3	2.20E-17	0.392890155	0.846	0.646	4.53E-13	8

QPRT	2.75E-17	0.342816653	0.592	0.359	5.67E-13	8
XRCC6	3.09E-17	0.282632579	0.715	0.508	6.36E-13	8
ATP5MC3	3.12E-17	0.330611965	0.88	0.694	6.43E-13	8
MSH6	3.75E-17	0.257945605	0.356	0.172	7.72E-13	8
RAB5IF	4.30E-17	0.298137031	0.697	0.468	8.85E-13	8
SLC25A13	4.38E-17	0.31438732	0.543	0.315	9.03E-13	8
PDIA6	4.95E-17	0.293621131	0.831	0.637	1.02E-12	8
MRPL14	6.61E-17	0.336656957	0.67	0.444	1.36E-12	8
CES1	7.22E-17	0.421981117	0.738	0.518	1.49E-12	8
ECI1	8.17E-17	0.276061672	0.599	0.381	1.68E-12	8
FAM136A	1.20E-16	0.274359919	0.584	0.357	2.47E-12	8
PRDX3	1.34E-16	0.28831953	0.719	0.499	2.77E-12	8
PON3	1.40E-16	0.256150829	0.528	0.282	2.88E-12	8
KPNB1	1.42E-16	0.317081796	0.682	0.479	2.92E-12	8
HSPE1	1.44E-16	0.265908263	0.918	0.832	2.97E-12	8
HMGA1	1.52E-16	0.26809506	0.547	0.333	3.13E-12	8
TRIM24	1.85E-16	0.253421132	0.483	0.272	3.80E-12	8
ONECUT2	1.97E-16	0.356153055	0.543	0.306	4.06E-12	8
TXNRD1	2.29E-16	0.330925801	0.528	0.31	4.72E-12	8
TKFC	2.88E-16	0.290050855	0.442	0.238	5.94E-12	8
SUMO2	3.03E-16	0.322232882	0.888	0.77	6.24E-12	8
GPAM	3.85E-16	0.263045855	0.472	0.251	7.93E-12	8
ACSL4	3.99E-16	0.386246151	0.573	0.367	8.21E-12	8
POLR2K	4.54E-16	0.256937368	0.655	0.435	9.35E-12	8
DYNLL1	5.78E-16	0.382619832	0.884	0.751	1.19E-11	8
PA2G4	6.17E-16	0.292078881	0.667	0.462	1.27E-11	8
HSD17B10	7.67E-16	0.313621254	0.663	0.429	1.58E-11	8
ALDH1A1	9.07E-16	0.410054559	0.581	0.371	1.87E-11	8
PARP1	9.30E-16	0.310757058	0.614	0.404	1.91E-11	8
ARG1	1.24E-15	0.319167501	0.61	0.349	2.56E-11	8
CDH2	1.29E-15	0.293349763	0.498	0.281	2.66E-11	8
RALY	1.37E-15	0.252578119	0.644	0.412	2.82E-11	8
ERH	1.39E-15	0.35199415	0.727	0.529	2.87E-11	8
PDCD5	1.43E-15	0.311185362	0.663	0.442	2.94E-11	8
MBNL3	1.58E-15	0.302656912	0.408	0.215	3.25E-11	8
PPIA	2.00E-15	0.384591574	0.925	0.845	4.12E-11	8
CCT7	2.45E-15	0.319278539	0.67	0.454	5.05E-11	8
SMARCC1	3.66E-15	0.257961099	0.558	0.343	7.54E-11	8
IDH1	3.71E-15	0.361738009	0.648	0.462	7.64E-11	8
SRP9	3.81E-15	0.328334119	0.745	0.532	7.85E-11	8
PTBP1	4.21E-15	0.286791595	0.629	0.4	8.67E-11	8
PLG	8.47E-15	0.346246296	0.592	0.363	1.74E-10	8
TXNL4A	1.02E-14	0.339218345	0.682	0.457	2.11E-10	8
HACD2	1.09E-14	0.306859906	0.476	0.275	2.24E-10	8
SERPINF2	1.45E-14	0.260508451	0.513	0.294	2.99E-10	8

SERPINC1	1.64E-14	0.360888084	0.663	0.458	3.37E-10	8
TMEM120A	1.66E-14	0.250852233	0.577	0.349	3.42E-10	8
POLR2I	1.70E-14	0.260399868	0.629	0.408	3.50E-10	8
CYB5A	2.02E-14	0.321628153	0.831	0.632	4.15E-10	8
SRSF2	2.03E-14	0.289854092	0.753	0.587	4.18E-10	8
ACP1	2.20E-14	0.258028636	0.667	0.447	4.53E-10	8
RARRES2	2.20E-14	0.30704791	0.764	0.536	4.54E-10	8
AHSG	2.33E-14	0.295058986	0.91	0.799	4.80E-10	8
MDH1	2.33E-14	0.289295549	0.708	0.512	4.81E-10	8
AK4	2.37E-14	0.282422447	0.498	0.286	4.89E-10	8
NDUFB3	2.53E-14	0.309597069	0.7	0.508	5.21E-10	8
NELFE	2.61E-14	0.30918014	0.607	0.396	5.38E-10	8
PRDX2	3.27E-14	0.309008899	0.689	0.485	6.74E-10	8
NDUFV2	3.66E-14	0.285432675	0.805	0.606	7.53E-10	8
CCT5	4.08E-14	0.267167037	0.648	0.446	8.40E-10	8
MRPL27	4.15E-14	0.252717898	0.513	0.3	8.55E-10	8
SAA4	4.56E-14	0.284172439	0.551	0.326	9.39E-10	8
AGXT	4.60E-14	0.349915876	0.757	0.546	9.47E-10	8
MRPL51	5.99E-14	0.312401498	0.674	0.471	1.23E-09	8
AKR1C1	6.15E-14	0.383687167	0.61	0.385	1.27E-09	8
TMEM230	6.82E-14	0.273313047	0.764	0.567	1.40E-09	8
MPLKIP	6.90E-14	0.262960724	0.633	0.395	1.42E-09	8
EPHX1	6.98E-14	0.347889331	0.637	0.421	1.44E-09	8
EIF3B	7.65E-14	0.250800155	0.554	0.349	1.58E-09	8
MRTO4	7.70E-14	0.264080706	0.412	0.217	1.59E-09	8
TCEA3	8.09E-14	0.252454554	0.434	0.229	1.67E-09	8
F2	8.65E-14	0.281449863	0.644	0.413	1.78E-09	8
AHCY	9.01E-14	0.255406918	0.588	0.38	1.86E-09	8
GC	1.06E-13	0.359038986	0.625	0.427	2.19E-09	8
PHB	1.08E-13	0.292319365	0.674	0.492	2.22E-09	8
PPA1	1.36E-13	0.262998483	0.772	0.544	2.81E-09	8
H1F0	1.47E-13	0.447135066	0.536	0.341	3.03E-09	8
HP1BP3	1.55E-13	0.278512742	0.655	0.444	3.20E-09	8
ADI1	1.70E-13	0.320407088	0.697	0.497	3.50E-09	8
ETFB	1.85E-13	0.319730635	0.693	0.525	3.80E-09	8
NDUFAB1	1.98E-13	0.261133635	0.712	0.516	4.07E-09	8
EI24	2.00E-13	0.294994226	0.558	0.369	4.12E-09	8
NUPR1	2.06E-13	0.283380091	0.618	0.384	4.24E-09	8
VTN	2.09E-13	0.32348218	0.745	0.529	4.31E-09	8
F12	2.47E-13	0.263865356	0.39	0.201	5.09E-09	8
CSNK2B	2.55E-13	0.325627865	0.779	0.569	5.25E-09	8
ECI2	3.38E-13	0.309473699	0.584	0.383	6.96E-09	8
ACSL3	3.88E-13	0.310099009	0.502	0.329	7.98E-09	8
SDHC	4.66E-13	0.256573557	0.719	0.499	9.60E-09	8
HMGN3	4.75E-13	0.263566861	0.633	0.429	9.77E-09	8

DCTN3	4.82E-13	0.323090814	0.637	0.411	9.92E-09	8
COA3	5.15E-13	0.269102011	0.607	0.4	1.06E-08	8
UBXN4	5.46E-13	0.253741414	0.742	0.553	1.13E-08	8
MGST1	5.93E-13	0.375015727	0.663	0.474	1.22E-08	8
SRSF10	6.18E-13	0.312936759	0.637	0.438	1.27E-08	8
PRDX6	6.49E-13	0.252055439	0.768	0.566	1.34E-08	8
PSMB7	6.94E-13	0.278893328	0.652	0.439	1.43E-08	8
APOC3	7.07E-13	0.318636352	0.876	0.751	1.46E-08	8
AMBP	7.25E-13	0.331719758	0.858	0.733	1.49E-08	8
PSMB5	7.27E-13	0.26787705	0.637	0.421	1.50E-08	8
PSMG2	7.71E-13	0.255209183	0.633	0.419	1.59E-08	8
DSP	8.50E-13	0.310951262	0.483	0.282	1.75E-08	8
IDI1	9.82E-13	0.324308141	0.682	0.482	2.02E-08	8
PSMA3	1.02E-12	0.275243577	0.67	0.476	2.10E-08	8
MEG3	1.14E-12	0.468591218	0.461	0.277	2.34E-08	8
NDUFAF3	1.22E-12	0.283512513	0.715	0.509	2.52E-08	8
FST	1.29E-12	0.28807139	0.513	0.314	2.65E-08	8
HNRNPD	2.27E-12	0.333986631	0.708	0.529	4.68E-08	8
HNRNPM	3.81E-12	0.250631107	0.7	0.505	7.84E-08	8
AKR1B10	3.90E-12	0.453766731	0.408	0.229	8.04E-08	8
ALB	5.31E-12	0.25549185	1	0.993	1.09E-07	8
EIF2S2	5.53E-12	0.261506241	0.712	0.53	1.14E-07	8
SUMO1	5.93E-12	0.291038689	0.742	0.546	1.22E-07	8
SLC25A5	6.05E-12	0.30205357	0.82	0.642	1.25E-07	8
RPS6KA3	8.61E-12	0.259208762	0.566	0.375	1.77E-07	8
RPL7L1	9.13E-12	0.283083995	0.596	0.412	1.88E-07	8
SFPQ	9.29E-12	0.30757566	0.727	0.542	1.91E-07	8
SSBP1	9.59E-12	0.272304743	0.753	0.581	1.97E-07	8
HNRNPA1	1.16E-11	0.313408286	0.906	0.793	2.39E-07	8
PROC	1.53E-11	0.303487017	0.569	0.354	3.14E-07	8
EIF5B	1.60E-11	0.301382547	0.644	0.478	3.29E-07	8
SYNCRIP	1.69E-11	0.259456839	0.61	0.425	3.48E-07	8
PSMB4	1.73E-11	0.291980877	0.749	0.572	3.57E-07	8
HNRNPF	2.03E-11	0.304863737	0.73	0.566	4.18E-07	8
APOB	2.16E-11	0.416816856	0.839	0.756	4.46E-07	8
DNPH1	2.32E-11	0.28169865	0.674	0.483	4.79E-07	8
LRPPRC	3.60E-11	0.270867438	0.506	0.34	7.41E-07	8
ENO1	5.56E-11	0.284410795	0.846	0.728	1.15E-06	8
ECH1	5.79E-11	0.27783296	0.738	0.552	1.19E-06	8
TALDO1	7.25E-11	0.277220095	0.753	0.583	1.49E-06	8
RRBP1	7.84E-11	0.30627294	0.625	0.461	1.61E-06	8
SLC25A3	8.64E-11	0.251118525	0.831	0.734	1.78E-06	8
PGAM1	9.12E-11	0.353592014	0.719	0.554	1.88E-06	8
PTP4A1	9.74E-11	0.313579003	0.704	0.536	2.01E-06	8
TFG	1.16E-10	0.266717456	0.596	0.417	2.39E-06	8

ATP5IF1	1.26E-10	0.278627492	0.757	0.584	2.59E-06	8
CUTA	1.40E-10	0.270293274	0.798	0.639	2.88E-06	8
GATM	1.65E-10	0.327604407	0.622	0.453	3.41E-06	8
UQCRC1	1.81E-10	0.281936368	0.64	0.472	3.73E-06	8
EIF5A	1.51E-09	0.262120509	0.727	0.526	3.10E-05	8
SRSF9	2.27E-09	0.27754865	0.749	0.58	4.68E-05	8
CHCHD10	3.73E-09	0.307881108	0.779	0.658	7.67E-05	8
GUSB	5.26E-09	0.26359092	0.584	0.43	0.000108262	8
TOP1	9.27E-09	0.256646227	0.637	0.485	0.000190813	8
PPP1CA	4.84E-08	0.290913185	0.745	0.57	0.000997009	8
IDH2	1.40E-07	0.269234169	0.521	0.366	0.002882649	8
PARK7	1.82E-07	0.26011297	0.783	0.661	0.003757874	8
HNRNPA2B1	3.24E-07	0.265254639	0.869	0.813	0.006664234	8
CCDC88A	2.34E-05	0.267813593	0.502	0.384	0.481222937	8
COX8A	0.000255069	0.261066866	0.745	0.632	1	8
ACTG1	0.009448313	0.273758458	0.955	0.911	1	8
PLVAP	0	2.568520959	0.771	0.033	0	9
EGFL7	0	2.44449276	0.744	0.059	0	9
GNG11	0	2.302759415	0.691	0.042	0	9
CLDN5	0	1.970109197	0.498	0.012	0	9
CALCRL	0	1.653084482	0.502	0.012	0	9
SOX18	0	1.596761586	0.498	0.013	0	9
CD34	0	1.56866271	0.525	0.013	0	9
CLEC14A	0	1.552223485	0.48	0.01	0	9
CDH5	0	1.529460262	0.525	0.01	0	9
ADGRL4	0	1.4853191	0.462	0.01	0	9
TIE1	7.93E-307	1.055628371	0.341	0.005	1.63E-302	9
AQP1	1.17E-295	1.567446458	0.448	0.015	2.40E-291	9
TM4SF18	6.08E-292	1.309290092	0.386	0.009	1.25E-287	9
STC1	5.61E-290	1.476275377	0.386	0.009	1.16E-285	9
PODXL	1.36E-281	1.559891147	0.484	0.021	2.80E-277	9
ADAMTS5	1.38E-272	1.381151891	0.35	0.008	2.84E-268	9
EFNB2	1.02E-271	1.350976736	0.372	0.01	2.09E-267	9
KDR	3.49E-270	1.031258007	0.327	0.006	7.19E-266	9
FLT1	1.81E-268	1.854114059	0.507	0.026	3.74E-264	9
EMCN	9.27E-268	1.198576128	0.377	0.01	1.91E-263	9
MMRN2	2.66E-267	1.217194651	0.372	0.01	5.48E-263	9
LDB2	3.45E-256	1.082849323	0.35	0.009	7.11E-252	9
ROBO4	1.91E-249	0.911582961	0.296	0.005	3.92E-245	9
GJA1	1.62E-248	1.356200836	0.336	0.008	3.33E-244	9
PCDH17	2.30E-246	1.412950504	0.439	0.02	4.73E-242	9
PTPRB	4.10E-245	1.075594695	0.287	0.005	8.45E-241	9
EDNRB	6.10E-243	1.76183168	0.516	0.032	1.26E-238	9
CRIP2	2.04E-240	1.763335827	0.605	0.049	4.20E-236	9
ESAM	1.05E-239	1.305060015	0.448	0.022	2.16E-235	9

COL4A1	3.86E-239	1.88653906	0.605	0.047	7.95E-235	9
HSPG2	1.84E-232	2.210337791	0.632	0.059	3.78E-228	9
ECSCR	1.90E-231	1.034068125	0.318	0.008	3.91E-227	9
DLL4	6.13E-229	1.066269893	0.3	0.007	1.26E-224	9
NPR1	1.67E-227	0.963681287	0.269	0.005	3.44E-223	9
NOTCH4	4.08E-224	1.440752207	0.435	0.023	8.40E-220	9
COL4A2	1.44E-222	1.5757436	0.578	0.046	2.97E-218	9
CXorf36	1.80E-216	0.982345696	0.278	0.006	3.71E-212	9
IFI27	5.10E-216	1.916309911	0.61	0.056	1.05E-211	9
ESM1	3.51E-215	1.556996065	0.296	0.008	7.24E-211	9
SPARC	1.36E-212	2.389746725	0.812	0.123	2.80E-208	9
CDH13	6.13E-207	1.046145517	0.296	0.008	1.26E-202	9
IGFBP7	7.22E-197	2.364408952	0.816	0.138	1.49E-192	9
LAMA4	1.22E-196	1.373920378	0.413	0.024	2.52E-192	9
CAVIN1	2.58E-194	1.327302282	0.457	0.032	5.32E-190	9
UNC5B	4.40E-193	1.446382429	0.377	0.02	9.07E-189	9
KCNN3	2.99E-182	0.877716157	0.296	0.011	6.16E-178	9
LAMB1	2.16E-173	1.186163099	0.363	0.021	4.44E-169	9
PCDH12	1.92E-164	0.935017074	0.256	0.008	3.96E-160	9
HTRA1	1.41E-162	1.014911939	0.327	0.018	2.91E-158	9
CAV1	1.97E-161	1.598280559	0.502	0.051	4.06E-157	9
RAMP2	1.10E-152	1.660664391	0.475	0.05	2.26E-148	9
PECAM1	2.28E-142	2.150257848	0.726	0.16	4.70E-138	9
ADGRF5	5.67E-141	0.974627378	0.327	0.021	1.17E-136	9
TP53I11	2.63E-137	1.408022415	0.408	0.039	5.43E-133	9
MCAM	1.00E-132	1.179080037	0.386	0.034	2.06E-128	9
IGFBP3	1.18E-132	1.883050096	0.278	0.016	2.42E-128	9
NPDC1	3.60E-132	1.272974104	0.435	0.047	7.41E-128	9
SEMA3F	1.04E-120	0.943770208	0.291	0.02	2.15E-116	9
PXDN	1.13E-120	0.921724568	0.256	0.014	2.32E-116	9
IGFBP5	1.82E-120	2.654338352	0.377	0.038	3.75E-116	9
SEMA6B	2.34E-118	0.916501543	0.283	0.019	4.82E-114	9
PLK2	8.29E-115	1.592037547	0.529	0.089	1.71E-110	9
TM4SF1	1.43E-109	1.736930851	0.534	0.096	2.95E-105	9
MAP1B	1.27E-105	0.911029435	0.305	0.026	2.62E-101	9
ANGPT2	1.20E-103	1.329808269	0.291	0.025	2.48E-99	9
TCIM	2.15E-102	0.93514638	0.256	0.018	4.42E-98	9
AFAP1L1	3.23E-102	0.848905111	0.265	0.02	6.65E-98	9
DOCK6	2.58E-99	1.103773272	0.363	0.044	5.31E-95	9
ADAMTS1	9.24E-88	1.010937773	0.26	0.023	1.90E-83	9
EMP1	1.24E-87	0.957409837	0.274	0.026	2.55E-83	9
SYNPO	4.61E-87	0.912155782	0.278	0.028	9.50E-83	9
DOCK9	7.71E-86	0.932152824	0.291	0.031	1.59E-81	9
CAVIN2	1.79E-82	1.139966209	0.327	0.043	3.69E-78	9
CAV2	2.13E-81	0.873779774	0.269	0.028	4.39E-77	9

HYAL2	5.81E-74	1.699124845	0.61	0.205	1.20E-69	9
INSR	3.53E-72	1.983939976	0.65	0.252	7.28E-68	9
VIM	1.69E-66	1.235517297	0.937	0.567	3.49E-62	9
FZD4	7.10E-66	0.728402645	0.269	0.035	1.46E-61	9
TCF4	6.61E-64	1.788440847	0.628	0.255	1.36E-59	9
PTPRM	1.49E-61	0.842136458	0.265	0.037	3.06E-57	9
APP	3.90E-60	1.736453309	0.556	0.199	8.02E-56	9
DYSF	2.50E-59	0.81592152	0.283	0.044	5.14E-55	9
SPRY1	3.69E-59	1.218620761	0.39	0.087	7.61E-55	9
SERPINI1	6.34E-59	1.378080744	0.314	0.055	1.31E-54	9
PLPP1	4.57E-57	1.577141777	0.439	0.119	9.41E-53	9
F2R	2.87E-56	0.714726756	0.251	0.035	5.90E-52	9
ID3	9.12E-56	1.266929863	0.475	0.133	1.88E-51	9
S1PR1	4.93E-55	0.80308864	0.278	0.045	1.02E-50	9
RAPGEF5	1.28E-53	1.163522499	0.359	0.081	2.64E-49	9
SLC9A3R2	5.39E-52	1.587944387	0.673	0.346	1.11E-47	9
CRIM1	6.14E-50	1.014916788	0.305	0.061	1.26E-45	9
CD93	1.11E-47	1.15884347	0.462	0.149	2.29E-43	9
PTP4A3	2.30E-47	0.806642725	0.269	0.048	4.73E-43	9
A2M	6.81E-46	1.516330752	0.619	0.28	1.40E-41	9
ARHGAP29	1.44E-42	1.308228943	0.493	0.198	2.96E-38	9
TIMP3	1.29E-39	1.379285664	0.561	0.28	2.65E-35	9
GSN	4.27E-38	1.324734005	0.587	0.296	8.80E-34	9
CD9	1.13E-37	1.371439021	0.556	0.271	2.33E-33	9
ENG	2.33E-36	1.149076146	0.372	0.122	4.79E-32	9
ITGA6	1.57E-35	1.289962325	0.475	0.209	3.23E-31	9
ITGA5	4.99E-35	1.00609636	0.354	0.112	1.03E-30	9
FKBP1A	9.10E-35	1.365578204	0.726	0.638	1.87E-30	9
INHBB	1.42E-34	1.21109577	0.314	0.09	2.92E-30	9
SMTN	1.43E-34	0.883441102	0.278	0.069	2.94E-30	9
NOTCH1	3.28E-33	0.790801035	0.278	0.071	6.76E-29	9
MARCKSL1	3.87E-33	1.328138583	0.587	0.345	7.96E-29	9
FSCN1	2.80E-32	0.83555454	0.345	0.11	5.77E-28	9
CD36	5.69E-32	1.354272136	0.475	0.215	1.17E-27	9
MT-ATP6	1.67E-31	0.538583117	1	1	3.44E-27	9
IFITM3	2.21E-31	1.007310823	0.78	0.648	4.55E-27	9
COL18A1	1.04E-30	1.108516469	0.534	0.286	2.15E-26	9
MT-CO3	1.08E-30	0.491562516	1	1	2.22E-26	9
SERPINH1	1.84E-30	1.085094785	0.475	0.22	3.78E-26	9
SPTBN1	5.39E-30	0.923211878	0.614	0.352	1.11E-25	9
ELK3	4.13E-29	0.673600152	0.256	0.067	8.50E-25	9
MEF2C	8.36E-28	0.930927097	0.435	0.193	1.72E-23	9
MT-CYB	5.50E-27	0.448409852	1	1	1.13E-22	9
YBX3	6.02E-27	0.917707568	0.475	0.224	1.24E-22	9
ID1	1.37E-26	1.063123865	0.26	0.075	2.81E-22	9

CD81	1.67E-26	0.934128424	0.623	0.403	3.45E-22	9
PLXND1	4.48E-26	1.091304745	0.426	0.205	9.22E-22	9
IVNS1ABP	8.43E-26	1.329029463	0.453	0.235	1.74E-21	9
LAMA5	9.59E-26	0.783200081	0.251	0.073	1.97E-21	9
PLPP3	1.33E-25	1.22069995	0.413	0.195	2.75E-21	9
MT-ND3	1.35E-25	0.432930939	0.996	0.999	2.79E-21	9
ETS1	1.50E-25	0.703087434	0.323	0.108	3.09E-21	9
CD59	1.59E-25	1.326041899	0.534	0.337	3.28E-21	9
ADAM15	7.42E-25	1.045272125	0.354	0.146	1.53E-20	9
ITGB1	1.22E-24	1.072884892	0.65	0.553	2.50E-20	9
LAMC1	2.48E-24	1.0486121	0.377	0.166	5.10E-20	9
CPNE2	4.09E-24	0.811441857	0.283	0.095	8.43E-20	9
NFIB	2.47E-23	1.012074066	0.404	0.197	5.08E-19	9
CALM1	6.92E-23	0.873316901	0.767	0.74	1.43E-18	9
STAB1	1.41E-22	0.788783354	0.395	0.17	2.91E-18	9
RGCC	1.05E-21	1.08680241	0.426	0.204	2.15E-17	9
TGFBR2	4.14E-21	1.177597137	0.484	0.31	8.53E-17	9
MT-ND1	9.92E-21	0.403330459	1	0.999	2.04E-16	9
GNAS	8.32E-19	0.747895641	0.731	0.766	1.71E-14	9
CTNNB1	8.87E-19	1.029933963	0.52	0.381	1.83E-14	9
MT-ND4	1.67E-18	0.353783457	1	1	3.44E-14	9
TNS2	2.09E-18	0.721677376	0.274	0.108	4.30E-14	9
UACA	1.15E-17	0.909800068	0.354	0.179	2.38E-13	9
IGFBP4	2.03E-17	1.052320508	0.444	0.283	4.18E-13	9
ICAM2	2.29E-17	0.816860581	0.336	0.158	4.71E-13	9
SEC14L1	6.34E-17	1.002601085	0.422	0.257	1.31E-12	9
CLIC4	2.88E-16	1.013869442	0.372	0.21	5.92E-12	9
ARRDC3	3.60E-16	0.896904312	0.377	0.207	7.41E-12	9
TSC22D1	4.80E-16	1.052425285	0.457	0.311	9.88E-12	9
MTUS1	6.04E-16	0.931969132	0.363	0.201	1.24E-11	9
TNFRSF4	9.77E-16	0.774343354	0.314	0.133	2.01E-11	9
RGS3	1.05E-15	1.001086423	0.3	0.143	2.17E-11	9
NRP2	2.32E-14	0.572690873	0.26	0.112	4.78E-10	9
MT-ND2	2.66E-14	0.288496203	1	0.998	5.48E-10	9
BCAM	2.76E-14	0.979471506	0.408	0.267	5.69E-10	9
MT-CO2	2.78E-14	0.303116847	1	1	5.72E-10	9
RHOC	4.47E-14	1.017223717	0.543	0.465	9.21E-10	9
ZEB1	4.79E-14	0.669821668	0.283	0.133	9.87E-10	9
S100A16	1.05E-13	0.889565281	0.444	0.303	2.16E-09	9
VWA1	3.01E-13	0.875371985	0.296	0.153	6.21E-09	9
TMSB10	3.59E-13	0.438521457	0.96	0.899	7.39E-09	9
ECE1	3.70E-12	0.87714428	0.309	0.172	7.61E-08	9
MAP4K4	7.39E-12	0.848211113	0.399	0.273	1.52E-07	9
CCDC85B	1.12E-11	0.970678237	0.48	0.395	2.31E-07	9
TIMP1	4.55E-11	0.64594713	0.457	0.309	9.36E-07	9

LMO2	5.59E-11	0.721980722	0.256	0.129	1.15E-06	9
KIAA0355	6.64E-11	0.632201055	0.269	0.142	1.37E-06	9
TNFAIP8L1	9.43E-11	0.808951816	0.269	0.145	1.94E-06	9
NRP1	9.66E-11	0.807063692	0.341	0.213	1.99E-06	9
LGALS1	1.17E-10	0.521701879	0.709	0.59	2.42E-06	9
SH3BP5	1.19E-10	0.965680514	0.413	0.311	2.45E-06	9
LMNA	2.07E-10	0.720076187	0.561	0.52	4.26E-06	9
MYH9	2.68E-10	0.799655415	0.543	0.527	5.52E-06	9
TMSB4X	2.70E-10	0.302390268	0.982	0.821	5.55E-06	9
IFITM2	3.98E-10	0.716510644	0.529	0.459	8.20E-06	9
AGRN	4.75E-10	0.711784818	0.265	0.146	9.79E-06	9
HDAC7	1.61E-09	0.566413382	0.251	0.131	3.31E-05	9
MYO6	4.22E-09	0.679707864	0.269	0.157	8.70E-05	9
JUP	4.81E-09	0.731499362	0.287	0.177	9.91E-05	9
PLEC	7.96E-09	0.611423877	0.296	0.184	0.000163996	9
SERPINB6	1.36E-08	0.760285314	0.386	0.294	0.000279153	9
GYPC	1.36E-07	0.667260993	0.381	0.271	0.002804764	9
NID1	2.41E-07	0.830398036	0.336	0.252	0.004953961	9
EPAS1	2.70E-07	0.759225897	0.363	0.284	0.005550501	9
TIMP2	3.09E-07	0.441407112	0.345	0.218	0.0063602	9
SNRK	1.31E-06	0.593936265	0.256	0.165	0.027081807	9
FYN	2.00E-06	0.412540203	0.309	0.199	0.041244134	9
BMPR2	2.50E-06	0.574683468	0.256	0.17	0.051520942	9
TGM2	2.59E-06	0.87840772	0.314	0.238	0.053250744	9
NEAT1	2.86E-06	0.553731403	0.704	0.786	0.058862029	9
DGKH	6.90E-06	0.571607443	0.256	0.171	0.142193811	9
LAMB2	8.45E-06	0.7225867	0.323	0.257	0.173986822	9
XAF1	2.59E-05	0.638313767	0.3	0.224	0.534123232	9
TPM4	5.05E-05	0.638398348	0.417	0.376	1	9
EFNA1	5.17E-05	0.524394556	0.39	0.324	1	9
RTN4	5.28E-05	0.473977406	0.565	0.627	1	9
DEPP1	6.34E-05	1.045126612	0.283	0.223	1	9
BEX3	7.37E-05	0.686344134	0.345	0.295	1	9
EMP2	7.87E-05	0.651020777	0.283	0.222	1	9
CCND1	9.32E-05	0.72860614	0.318	0.268	1	9
NUDT4	0.000118359	0.305375588	0.148	0.302	1	9
CD151	0.00012437	0.701774811	0.291	0.235	1	9
GNAI2	0.000217746	0.462698675	0.605	0.712	1	9
MALAT1	0.000302396	0.39519065	0.937	0.954	1	9
SNAP23	0.000341339	0.262819982	0.17	0.329	1	9
TAGLN2	0.000367713	0.567112078	0.475	0.472	1	9
YWHAH	0.000377012	0.706654372	0.413	0.417	1	9
EVA1B	0.00043839	0.583760334	0.278	0.225	1	9
TM9SF2	0.000442346	0.254748349	0.211	0.392	1	9
AGPAT1	0.000780836	0.286085534	0.139	0.273	1	9

RDX	0.000799781	0.822526324	0.399	0.416	1	9
CD99	0.000828726	0.598745943	0.507	0.579	1	9
PHC2	0.00096008	0.271007235	0.139	0.269	1	9
VAMP5	0.000997962	0.809650175	0.43	0.458	1	9
NUCB1	0.001113471	0.277703095	0.229	0.411	1	9
MSN	0.001243315	0.487593358	0.43	0.417	1	9
STOM	0.001474409	0.810618409	0.368	0.376	1	9
GLG1	0.001765928	0.266295967	0.184	0.337	1	9
GNAQ	0.001781453	0.289300465	0.148	0.279	1	9
MRPL22	0.001844426	0.268395932	0.175	0.315	1	9
RALA	0.001898359	0.293913084	0.184	0.337	1	9
AIDA	0.002098005	0.250112946	0.139	0.26	1	9
SYNE2	0.002299786	0.589940042	0.296	0.255	1	9
BPTF	0.002407759	0.260668091	0.256	0.437	1	9
MARCKS	0.002606819	0.321741368	0.547	0.546	1	9
BNIP2	0.003173705	0.345636642	0.166	0.304	1	9
APLP2	0.003494773	0.414493711	0.525	0.579	1	9
S100A6	0.003915861	0.301320734	0.511	0.457	1	9
EIF4A1	0.004501047	0.253963141	0.314	0.527	1	9
SORBS3	0.004691617	0.282685972	0.139	0.252	1	9
SREK1	0.004931525	0.258368402	0.193	0.338	1	9
SLC38A10	0.005104036	0.267885711	0.152	0.271	1	9
DDIT4	0.005106936	0.501552811	0.404	0.389	1	9
PCMTD1	0.005229649	0.257161565	0.17	0.297	1	9
SH3GLB1	0.005277042	0.263180726	0.22	0.375	1	9
UBA1	0.005888724	0.328245877	0.166	0.293	1	9
ANXA1	0.006262908	0.280325712	0.39	0.334	1	9
TCEAL4	0.006898457	0.339187454	0.179	0.311	1	9
MRPL33	0.007785997	0.264224396	0.278	0.457	1	9
FLNA	0.008074504	0.307395077	0.318	0.276	1	9
CSNK1E	0.00845341	0.574075552	0.269	0.244	1	9
BRWD1	0.009758291	0.310479274	0.161	0.278	1	9
GNLY	0	3.979495934	0.984	0.036	0	10
NKG7	0	3.059813013	1	0.11	0	10
PRF1	0	2.748720735	0.989	0.079	0	10
GZMB	0	2.720231724	0.984	0.045	0	10
FGFBP2	0	2.695027581	0.935	0.009	0	10
KLRD1	0	2.386612883	0.919	0.036	0	10
PLAC8	0	2.081562651	0.903	0.077	0	10
KLRF1	0	2.041238357	0.859	0.008	0	10
CLIC3	0	1.847797911	0.681	0.017	0	10
TRDC	0	1.774471899	0.681	0.007	0	10
SH2D1B	0	0.992863681	0.384	0.002	0	10
PTGDS	2.19E-305	2.55885592	0.432	0.01	4.52E-301	10
CTSW	3.05E-301	2.112221884	0.881	0.077	6.28E-297	10

HOPX	1.51E-283	1.697865729	0.681	0.042	3.11E-279	10
CX3CR1	5.89E-281	1.273080535	0.535	0.023	1.21E-276	10
CD160	9.72E-280	1.125135973	0.405	0.01	2.00E-275	10
GZMH	2.84E-267	1.699306161	0.735	0.052	5.85E-263	10
GZMA	2.93E-264	1.928911277	0.935	0.098	6.04E-260	10
KLRB1	3.02E-262	1.789777611	0.822	0.073	6.21E-258	10
PTGDR	1.72E-261	0.870831913	0.314	0.005	3.55E-257	10
FCRL6	8.10E-255	1.026924384	0.395	0.011	1.67E-250	10
TXK	2.54E-253	1.009480675	0.373	0.01	5.22E-249	10
S1PR5	1.16E-243	0.910385627	0.324	0.006	2.38E-239	10
CD247	2.55E-242	2.083681133	0.946	0.132	5.25E-238	10
GZMM	3.85E-234	1.62398531	0.822	0.088	7.93E-230	10
CST7	8.62E-214	2.108487685	0.973	0.161	1.78E-209	10
TBX21	1.19E-212	1.072630379	0.47	0.025	2.45E-208	10
LAIR2	3.67E-212	1.605979577	0.805	0.091	7.57E-208	10
TRGC1	1.15E-193	0.832934683	0.357	0.014	2.37E-189	10
ADGRG1	1.20E-174	1.086841925	0.443	0.028	2.46E-170	10
PYHIN1	1.70E-149	1.208620657	0.557	0.059	3.51E-145	10
CCL5	8.45E-146	1.813900565	0.832	0.15	1.74E-141	10
CD7	4.87E-131	1.408145095	0.897	0.198	1.00E-126	10
IFNG	2.65E-128	1.925813984	0.611	0.084	5.45E-124	10
CCL4	1.14E-127	2.483255601	0.892	0.215	2.35E-123	10
ZAP70	3.48E-127	1.097692748	0.546	0.069	7.17E-123	10
FCGR3A	7.19E-123	1.476201006	0.886	0.209	1.48E-118	10
SAMD3	3.83E-120	0.898659033	0.351	0.026	7.90E-116	10
SPON2	2.56E-119	2.137545111	0.87	0.283	5.28E-115	10
XCL2	7.56E-117	1.421567459	0.4	0.036	1.56E-112	10
IL2RB	1.87E-116	1.151401828	0.562	0.079	3.86E-112	10
EOMES	4.50E-104	0.836845213	0.362	0.033	9.28E-100	10
NCR3	2.28E-99	0.807423003	0.33	0.029	4.69E-95	10
MATK	6.51E-96	1.059139465	0.416	0.052	1.34E-91	10
PRSS23	8.84E-89	0.737129619	0.303	0.027	1.82E-84	10
ARL4C	2.44E-88	1.363640836	0.903	0.344	5.04E-84	10
HSH2D	9.14E-85	0.720434408	0.308	0.031	1.88E-80	10
CCL3	1.26E-84	2.172630776	0.724	0.188	2.59E-80	10
C12orf75	1.34E-84	0.900477425	0.427	0.061	2.75E-80	10
KLF2	2.84E-81	1.378449655	0.849	0.289	5.84E-77	10
SYTL3	1.88E-77	0.882480184	0.405	0.059	3.87E-73	10
CCL4L2	1.01E-64	2.128108131	0.724	0.238	2.09E-60	10
MYBL1	7.52E-64	0.643517861	0.265	0.03	1.55E-59	10
ABHD17A	2.83E-63	1.3018805	0.724	0.306	5.82E-59	10
MALAT1	6.79E-63	0.895865683	1	0.952	1.40E-58	10
EFHD2	1.89E-62	1.190687579	0.811	0.4	3.90E-58	10
RARRES3	2.01E-62	1.177341622	0.741	0.294	4.14E-58	10
RAC2	5.85E-62	1.041517128	0.859	0.386	1.21E-57	10

HLA-C	4.98E-60	0.816916863	0.989	0.871	1.03E-55	10
C1orf21	1.06E-59	0.749904362	0.319	0.048	2.17E-55	10
CYBA	1.11E-59	1.030996903	0.946	0.528	2.28E-55	10
ITGB2	3.40E-58	1.047106227	0.822	0.349	7.00E-54	10
LIMD2	5.73E-57	1.041863373	0.768	0.327	1.18E-52	10
PLEK	2.61E-56	0.913818333	0.697	0.248	5.38E-52	10
HLA-E	6.51E-56	0.890809027	0.984	0.734	1.34E-51	10
DENND2D	9.01E-56	0.990716924	0.492	0.126	1.86E-51	10
HCST	1.27E-55	0.969634189	0.886	0.406	2.62E-51	10
TXNIP	2.54E-54	1.184798025	0.897	0.635	5.22E-50	10
PRKCH	3.12E-54	0.863300905	0.465	0.11	6.43E-50	10
CORO1A	5.45E-54	0.924508836	0.843	0.367	1.12E-49	10
GIMAP7	1.20E-53	1.033590879	0.611	0.205	2.47E-49	10
ALOX5AP	1.11E-51	0.812004904	0.492	0.123	2.28E-47	10
EVL	3.86E-51	0.985897196	0.757	0.349	7.95E-47	10
B2M	1.16E-50	0.731678297	1	0.946	2.39E-46	10
FCER1G	1.62E-50	0.707109612	0.903	0.319	3.33E-46	10
BTG1	2.86E-50	0.956082971	0.957	0.755	5.89E-46	10
FGR	5.20E-48	0.885699639	0.443	0.117	1.07E-43	10
BIN2	7.89E-48	1.041435512	0.508	0.159	1.62E-43	10
ANXA1	9.91E-48	0.960650966	0.751	0.322	2.04E-43	10
ID2	1.10E-46	1.11726908	0.849	0.599	2.27E-42	10
IL2RG	1.83E-46	0.956020386	0.724	0.327	3.76E-42	10
ITGB7	3.81E-46	0.81634441	0.362	0.078	7.84E-42	10
CHST2	1.60E-45	0.748749565	0.314	0.061	3.30E-41	10
CD53	2.07E-45	0.856400272	0.811	0.394	4.26E-41	10
CD69	3.18E-45	1.086692848	0.6	0.195	6.55E-41	10
TYROBP	1.01E-44	0.53236512	0.914	0.312	2.09E-40	10
MBP	1.48E-44	0.998616356	0.524	0.183	3.04E-40	10
EMP3	1.95E-44	0.911841199	0.784	0.4	4.01E-40	10
IER2	1.12E-43	1.155716176	0.832	0.492	2.31E-39	10
MYL12A	1.43E-43	0.802124594	0.957	0.806	2.94E-39	10
CCND3	1.59E-43	0.979045446	0.573	0.219	3.27E-39	10
IFITM2	1.62E-43	1.010734284	0.768	0.451	3.33E-39	10
IRF1	8.23E-43	0.961050062	0.805	0.426	1.70E-38	10
SPN	1.34E-42	0.878344348	0.432	0.121	2.76E-38	10
CALM1	1.63E-42	0.827964998	0.919	0.735	3.37E-38	10
S100A4	2.03E-42	0.651088859	0.941	0.444	4.18E-38	10
FYN	3.06E-42	0.814013137	0.568	0.191	6.31E-38	10
PTPN4	4.12E-42	0.934453877	0.449	0.138	8.50E-38	10
HLA-B	4.34E-42	0.709112485	0.984	0.656	8.93E-38	10
JUN	8.83E-42	1.170393995	0.897	0.676	1.82E-37	10
SYTL1	3.87E-40	0.676860175	0.314	0.066	7.96E-36	10
ARHGEF3	1.57E-39	0.713129264	0.341	0.08	3.24E-35	10
CHST12	2.77E-39	0.985710321	0.486	0.172	5.71E-35	10

CLEC2B	5.83E-39	0.932880886	0.67	0.315	1.20E-34	10
HLA-A	6.60E-39	0.552081867	1	0.902	1.36E-34	10
ATM	7.11E-39	1.142588101	0.649	0.361	1.46E-34	10
RUNX3	3.63E-38	0.89843541	0.53	0.192	7.47E-34	10
PFN1	9.10E-38	0.671727391	0.984	0.821	1.87E-33	10
JUND	1.64E-37	0.915473561	0.93	0.805	3.37E-33	10
SH2D2A	2.26E-37	0.669758957	0.335	0.078	4.65E-33	10
SRGN	1.32E-36	0.620693264	0.957	0.522	2.71E-32	10
ADAM8	6.19E-36	0.661631588	0.314	0.073	1.27E-31	10
PTPRC	4.44E-35	0.746475451	0.811	0.43	9.15E-31	10
TRAF3IP3	5.27E-35	0.777263389	0.459	0.152	1.08E-30	10
IFITM1	4.18E-34	0.693547385	0.341	0.086	8.60E-30	10
CD37	2.41E-33	0.713000904	0.692	0.348	4.96E-29	10
CEP78	4.51E-33	0.931814524	0.357	0.105	9.29E-29	10
LCK	8.36E-33	0.709830264	0.481	0.157	1.72E-28	10
CELF2	2.17E-31	0.87590402	0.584	0.287	4.48E-27	10
ADGRE5	4.22E-31	0.838816333	0.535	0.245	8.70E-27	10
TBC1D10C	9.18E-31	0.714954957	0.411	0.133	1.89E-26	10
CYTIP	2.25E-30	0.790774669	0.551	0.242	4.63E-26	10
DNAJB1	3.18E-30	0.903726256	0.859	0.62	6.54E-26	10
GPR65	8.52E-30	0.845040281	0.411	0.147	1.75E-25	10
CDKN2D	9.77E-30	0.686098408	0.346	0.102	2.01E-25	10
DOK2	1.63E-29	0.769986051	0.638	0.318	3.36E-25	10
TRBC1	2.06E-29	0.398802921	0.443	0.134	4.25E-25	10
CFL1	3.76E-29	0.517715623	0.984	0.839	7.75E-25	10
MYO1F	8.32E-29	0.805675338	0.497	0.221	1.71E-24	10
ADRB2	1.08E-28	0.745472875	0.286	0.075	2.22E-24	10
LITAF	3.94E-28	0.764491288	0.746	0.506	8.11E-24	10
HSPA1B	8.31E-28	0.841984631	0.876	0.626	1.71E-23	10
CD38	1.07E-27	0.704384005	0.314	0.093	2.20E-23	10
DDX5	1.44E-27	0.621234321	0.908	0.782	2.96E-23	10
RASGRP2	1.50E-27	0.61637355	0.281	0.074	3.10E-23	10
IKZF1	6.40E-27	0.797993904	0.519	0.244	1.32E-22	10
RIPOR2	9.76E-27	0.733281352	0.335	0.103	2.01E-22	10
TSC22D3	1.16E-26	0.817798348	0.665	0.353	2.39E-22	10
GNPTAB	1.45E-26	0.98652008	0.486	0.239	2.99E-22	10
CD48	2.71E-26	0.676342732	0.659	0.353	5.58E-22	10
GADD45B	8.08E-26	1.076006626	0.703	0.48	1.66E-21	10
SYNE1	8.18E-26	0.729408775	0.335	0.11	1.69E-21	10
AREG	8.51E-26	0.88103788	0.297	0.083	1.75E-21	10
GNG2	1.35E-25	0.793423539	0.427	0.175	2.78E-21	10
ZBTB16	3.41E-25	0.730285279	0.281	0.081	7.03E-21	10
SLAMF7	4.64E-25	0.655200779	0.324	0.103	9.56E-21	10
JAK1	4.70E-25	0.95898486	0.627	0.453	9.68E-21	10
IKZF3	8.19E-25	0.556030982	0.297	0.084	1.69E-20	10

RAP1B	2.30E-24	0.838533474	0.692	0.535	4.75E-20	10
PPP1R18	2.39E-24	0.813023493	0.557	0.315	4.92E-20	10
ARPC2	2.43E-24	0.548971488	0.892	0.757	5.00E-20	10
HAVCR2	2.57E-24	0.744587616	0.416	0.172	5.30E-20	10
FLNA	2.93E-24	0.697807804	0.535	0.269	6.03E-20	10
HSPA1A	4.07E-24	0.821705182	0.946	0.864	8.38E-20	10
GFOD1	1.76E-23	0.750926445	0.319	0.107	3.63E-19	10
ARHGDIB	3.91E-23	0.562195881	0.849	0.503	8.06E-19	10
STK4	4.14E-23	0.887572522	0.632	0.454	8.53E-19	10
PMAIP1	9.23E-23	0.856490173	0.557	0.281	1.90E-18	10
CD300A	1.18E-22	0.74936253	0.4	0.17	2.44E-18	10
SLA	1.57E-22	0.768962594	0.481	0.237	3.23E-18	10
AES	3.23E-22	0.823023343	0.681	0.559	6.65E-18	10
AC245297.3	5.47E-22	0.894033295	0.53	0.319	1.13E-17	10
BTN3A2	8.41E-22	0.856039768	0.492	0.26	1.73E-17	10
DUSP2	9.84E-22	0.80420285	0.535	0.25	2.03E-17	10
SUN2	1.14E-21	0.847378192	0.497	0.277	2.34E-17	10
PTGER4	1.29E-21	0.701884101	0.362	0.142	2.66E-17	10
UBB	2.29E-21	0.489595536	0.968	0.859	4.71E-17	10
GK5	2.95E-21	0.650178445	0.281	0.093	6.08E-17	10
H3F3B	1.05E-20	0.478985533	0.962	0.88	2.16E-16	10
ITGAL	1.07E-20	0.784441442	0.449	0.221	2.21E-16	10
SEPT7	1.31E-20	0.637337988	0.692	0.563	2.70E-16	10
FAM49B	4.61E-20	0.715113346	0.605	0.42	9.49E-16	10
CCL3L1	5.59E-20	0.888691986	0.486	0.218	1.15E-15	10
CD99	6.08E-20	0.688521676	0.697	0.572	1.25E-15	10
TGFB1	9.61E-20	0.757445324	0.47	0.251	1.98E-15	10
PPP1R15A	1.41E-19	0.815448485	0.676	0.457	2.91E-15	10
FCMR	1.48E-19	0.629238977	0.281	0.094	3.06E-15	10
LBH	1.67E-19	0.64381921	0.373	0.147	3.43E-15	10
LMO4	1.80E-19	0.750763682	0.405	0.191	3.70E-15	10
ICAM2	2.55E-19	0.707224336	0.368	0.158	5.24E-15	10
TLE4	2.99E-19	0.826765488	0.416	0.198	6.15E-15	10
LY6E	3.00E-19	0.844696198	0.551	0.341	6.18E-15	10
CCDC88C	3.21E-19	0.497528866	0.27	0.088	6.62E-15	10
GSTP1	3.89E-19	0.507272459	0.654	0.364	8.00E-15	10
CMC1	5.64E-19	0.882081985	0.546	0.377	1.16E-14	10
ABI3	1.11E-18	0.606571035	0.389	0.177	2.28E-14	10
SELPLG	1.35E-18	0.706697159	0.341	0.143	2.78E-14	10
UCP2	1.75E-18	0.555714586	0.6	0.34	3.60E-14	10
ADD3	3.18E-18	0.778861545	0.476	0.279	6.56E-14	10
AKNA	4.42E-18	0.730576659	0.378	0.174	9.11E-14	10
ISG20	5.01E-18	0.658440732	0.411	0.186	1.03E-13	10
PTPN7	5.49E-18	0.721530727	0.432	0.209	1.13E-13	10
APMAP	5.57E-18	0.718550627	0.6	0.461	1.15E-13	10

HNRNPA2B1	7.96E-18	0.444386979	0.87	0.814	1.64E-13	10
GLIPR2	1.43E-17	0.679524886	0.373	0.172	2.95E-13	10
CD81	1.75E-17	0.433478259	0.692	0.402	3.61E-13	10
JUNB	3.03E-17	0.747161356	0.768	0.548	6.24E-13	10
RBM39	3.74E-17	0.521933506	0.746	0.67	7.70E-13	10
SLC38A1	4.01E-17	0.591172648	0.297	0.112	8.27E-13	10
CDC42SE1	6.64E-17	0.805964354	0.481	0.303	1.37E-12	10
CTBP2	1.70E-16	0.585843848	0.276	0.106	3.51E-12	10
PCSK7	4.99E-16	0.810536741	0.486	0.307	1.03E-11	10
S100A6	6.41E-16	0.298203205	0.751	0.45	1.32E-11	10
PXN	6.68E-16	0.644330225	0.33	0.15	1.38E-11	10
ZEB2	6.86E-16	0.619008781	0.459	0.249	1.41E-11	10
ACAP1	7.90E-16	0.546273296	0.389	0.176	1.63E-11	10
METRNL	8.77E-16	0.719879017	0.389	0.196	1.81E-11	10
SYNE2	9.45E-16	0.718213153	0.443	0.25	1.95E-11	10
GIMAP4	1.36E-15	0.566293209	0.497	0.284	2.80E-11	10
FUS	1.54E-15	0.6553984	0.708	0.652	3.18E-11	10
RNF213	3.55E-15	0.731007766	0.616	0.498	7.30E-11	10
PIP4K2A	5.54E-15	0.695385646	0.373	0.195	1.14E-10	10
HSP90AA1	1.23E-14	0.508107699	0.957	0.89	2.54E-10	10
TMSB10	1.24E-14	0.284623646	0.995	0.899	2.56E-10	10
ARPC5L	1.54E-14	0.756526282	0.508	0.365	3.16E-10	10
PSMB9	1.71E-14	0.598248406	0.611	0.47	3.52E-10	10
TMSB4X	3.21E-14	0.323768162	1	0.822	6.61E-10	10
IFI16	3.29E-14	0.549524003	0.53	0.339	6.78E-10	10
SERTAD1	4.08E-14	0.757593303	0.427	0.248	8.40E-10	10
RNF166	4.66E-14	0.724005015	0.362	0.189	9.61E-10	10
CDC42EP3	8.43E-14	0.566397114	0.303	0.132	1.74E-09	10
OSTF1	9.35E-14	0.667098856	0.497	0.355	1.93E-09	10
TPST2	2.30E-13	0.724118578	0.454	0.31	4.73E-09	10
APOBEC3G	2.55E-13	0.569813753	0.373	0.188	5.25E-09	10
SKAP1	2.77E-13	0.535848074	0.324	0.152	5.71E-09	10
GIMAP1	3.09E-13	0.589281545	0.335	0.166	6.37E-09	10
PPP2R5C	3.23E-13	0.805482768	0.524	0.396	6.66E-09	10
LSP1	6.94E-13	0.469753349	0.557	0.364	1.43E-08	10
GSAP	7.43E-13	0.732049906	0.303	0.15	1.53E-08	10
RPS4Y1	8.00E-13	0.485536343	0.578	0.388	1.65E-08	10
PRKCB	1.25E-12	0.543165519	0.335	0.171	2.58E-08	10
RASSF5	1.29E-12	0.567365387	0.341	0.173	2.65E-08	10
BTG2	1.68E-12	0.814797916	0.562	0.423	3.47E-08	10
HSPA6	2.41E-12	1.32915416	0.465	0.29	4.97E-08	10
SH3BGRL3	2.72E-12	0.304709204	0.914	0.749	5.60E-08	10
DDIT4	4.33E-12	0.566360807	0.562	0.384	8.91E-08	10
TCF25	4.43E-12	0.617735248	0.584	0.517	9.13E-08	10
FAU	7.52E-12	0.254340072	0.962	0.903	1.55E-07	10

WIPF1	1.15E-11	0.520016003	0.454	0.298	2.36E-07	10
GPSM3	1.32E-11	0.514071784	0.492	0.33	2.72E-07	10
HLA-F	1.47E-11	0.513168092	0.378	0.211	3.03E-07	10
EIF3G	1.54E-11	0.60753292	0.616	0.586	3.17E-07	10
CARD16	1.56E-11	0.558066126	0.497	0.33	3.22E-07	10
DHRS7	1.99E-11	0.667861946	0.476	0.369	4.10E-07	10
FOS	2.50E-11	0.543643779	0.697	0.482	5.16E-07	10
PSME1	2.97E-11	0.475178885	0.681	0.648	6.12E-07	10
AOAH	3.33E-11	0.555107672	0.308	0.158	6.86E-07	10
MYO1G	3.74E-11	0.552532972	0.303	0.153	7.70E-07	10
SEMA4D	3.92E-11	0.457562201	0.265	0.12	8.07E-07	10
INPP5D	4.74E-11	0.541913632	0.319	0.17	9.76E-07	10
LCP1	4.79E-11	0.354575848	0.649	0.415	9.86E-07	10
NR4A1	4.92E-11	0.510445483	0.492	0.308	1.01E-06	10
HSPA8	4.96E-11	0.448021163	0.827	0.744	1.02E-06	10
ZFP36L2	6.15E-11	0.479322948	0.692	0.635	1.27E-06	10
SASH3	7.92E-11	0.459130816	0.27	0.127	1.63E-06	10
STARD3NL	1.79E-10	0.730288443	0.341	0.205	3.69E-06	10
INTS6	2.52E-10	0.643666506	0.524	0.42	5.20E-06	10
ETS1	2.96E-10	0.413940296	0.254	0.111	6.09E-06	10
TRIM22	5.30E-10	0.558433118	0.314	0.172	1.09E-05	10
BIN1	5.52E-10	0.660389593	0.422	0.307	1.14E-05	10
ARHGEF1	6.36E-10	0.613649314	0.395	0.263	1.31E-05	10
HNRNPDL	6.51E-10	0.468488438	0.659	0.643	1.34E-05	10
SLC2A3	7.87E-10	0.624360046	0.438	0.272	1.62E-05	10
CYFIP2	8.13E-10	0.503952781	0.27	0.136	1.67E-05	10
DUSP1	1.11E-09	0.466231437	0.773	0.635	2.28E-05	10
RAB27A	1.66E-09	0.477924389	0.314	0.172	3.41E-05	10
ACTG1	2.11E-09	0.291191311	0.962	0.912	4.35E-05	10
CD47	2.70E-09	0.622757537	0.459	0.371	5.56E-05	10
AKAP13	2.77E-09	0.645194559	0.514	0.443	5.70E-05	10
LAT	2.82E-09	0.365252251	0.27	0.129	5.81E-05	10
PSMB8	3.03E-09	0.4920902	0.551	0.48	6.24E-05	10
CACYBP	3.39E-09	0.694168595	0.616	0.596	6.99E-05	10
BST2	3.83E-09	0.443626511	0.481	0.335	7.89E-05	10
REL	3.96E-09	0.60018625	0.524	0.423	8.16E-05	10
SIGIRR	4.15E-09	0.605963964	0.351	0.226	8.55E-05	10
LNPEP	6.67E-09	0.587357099	0.346	0.226	0.000137338	10
C9orf16	7.33E-09	0.463090246	0.568	0.508	0.000150867	10
IQGAP1	1.07E-08	0.459752687	0.47	0.345	0.000220468	10
SH3BP5	1.10E-08	0.586074692	0.416	0.311	0.000226721	10
C1orf162	1.31E-08	0.429016439	0.395	0.238	0.000269752	10
RAB29	2.25E-08	0.685050127	0.389	0.29	0.000463665	10
MSN	2.26E-08	0.490945995	0.514	0.414	0.000465309	10
CTSC	3.04E-08	0.303667208	0.6	0.42	0.000626839	10

CCND2	3.50E-08	0.368807113	0.314	0.174	0.000721836	10
MAPRE2	3.69E-08	0.579020283	0.314	0.197	0.000759411	10
PREX1	4.11E-08	0.521528379	0.319	0.2	0.000845573	10
GMFG	5.90E-08	0.317024436	0.584	0.432	0.001214161	10
TAGAP	7.31E-08	0.474087066	0.324	0.188	0.001506223	10
PPP1R12A	8.25E-08	0.550509198	0.481	0.42	0.001699683	10
RGS3	8.86E-08	0.510568203	0.259	0.145	0.001824709	10
YWHAZ	1.00E-07	0.424901223	0.611	0.578	0.002063228	10
NFKBIA	1.11E-07	0.703381269	0.643	0.619	0.00228901	10
APOL6	2.01E-07	0.556538494	0.389	0.298	0.004130517	10
VAMP2	2.36E-07	0.582869706	0.503	0.45	0.004853604	10
SLC9A3R1	2.71E-07	0.413236029	0.486	0.418	0.005585687	10
CLIC1	3.30E-07	0.27573748	0.854	0.793	0.006800798	10
DGKZ	3.34E-07	0.602429064	0.335	0.233	0.006868803	10
XBP1	3.59E-07	0.47957764	0.568	0.53	0.007386649	10
HMGN2	4.43E-07	0.286758475	0.611	0.563	0.009132214	10
HIPK1	5.17E-07	0.578989299	0.378	0.295	0.010639771	10
PRPF38B	5.27E-07	0.549572194	0.514	0.494	0.010862834	10
PAXX	6.41E-07	0.579133364	0.405	0.321	0.013200774	10
NFATC2	9.42E-07	0.582992161	0.297	0.199	0.019395293	10
PRR5	9.84E-07	0.523940665	0.265	0.164	0.02025742	10
FMNL1	1.19E-06	0.505695307	0.362	0.258	0.024535999	10
HCLS1	1.22E-06	0.360771159	0.432	0.318	0.025128009	10
ARHGAP30	1.36E-06	0.393073552	0.303	0.196	0.028052903	10
AAK1	1.45E-06	0.467569163	0.432	0.36	0.029795497	10
TTC38	1.58E-06	0.562060995	0.368	0.292	0.032534569	10
SRRM2	3.45E-06	0.341907123	0.627	0.659	0.071130739	10
SAMD9	4.40E-06	0.447771877	0.265	0.165	0.090667521	10
CBLB	5.25E-06	0.451557157	0.276	0.177	0.108144581	10
TSPYL2	5.86E-06	0.589606847	0.308	0.202	0.12067576	10
SON	8.58E-06	0.298759659	0.605	0.629	0.176775396	10
LPCAT1	9.03E-06	0.491176386	0.314	0.23	0.18595438	10
SEPT1	9.25E-06	0.41328106	0.281	0.182	0.190446368	10
MAP3K8	1.04E-05	0.586104291	0.297	0.212	0.214597459	10
ARPC4	1.47E-05	0.358455316	0.546	0.534	0.302628277	10
RORA	1.92E-05	0.501630138	0.368	0.289	0.396274764	10
RHOC	2.03E-05	0.410875719	0.492	0.467	0.418883474	10
NR4A2	2.06E-05	0.344040498	0.416	0.291	0.42466512	10
PTPN12	2.27E-05	0.591979468	0.368	0.318	0.467696624	10
PKN1	2.63E-05	0.516663763	0.351	0.289	0.542363679	10
AGTRAP	3.25E-05	0.471801378	0.292	0.212	0.670347376	10
IDS	3.56E-05	0.477933822	0.368	0.294	0.732335062	10
TRIR	3.86E-05	0.421836716	0.568	0.624	0.794615358	10
CCDC85B	4.44E-05	0.557200864	0.432	0.398	0.914833193	10
PRMT2	4.84E-05	0.489837472	0.341	0.274	0.995947205	10

GBP2	4.99E-05	0.374737582	0.476	0.439	1	10
DAZAP2	6.40E-05	0.400811203	0.524	0.536	1	10
CDC42SE2	6.75E-05	0.500719021	0.373	0.315	1	10
YPEL3	7.37E-05	0.53986421	0.427	0.404	1	10
CD55	8.16E-05	0.490425242	0.254	0.173	1	10
MGAT4A	8.67E-05	0.465783549	0.276	0.203	1	10
HSPH1	9.63E-05	0.40948655	0.568	0.506	1	10
TAPBP	0.000115647	0.418851385	0.519	0.547	1	10
STK10	0.000116382	0.492624284	0.265	0.189	1	10
RSRP1	0.00014159	0.486818491	0.486	0.489	1	10
MCL1	0.00016657	0.343666282	0.605	0.621	1	10
CYCS	0.000211019	0.421869886	0.584	0.625	1	10
ARID1B	0.000231396	0.57117333	0.422	0.417	1	10
SRSF5	0.000250931	0.391850014	0.53	0.57	1	10
PTPN6	0.000268253	0.455639166	0.346	0.286	1	10
ARF6	0.000277221	0.481101331	0.47	0.476	1	10
KMT2E	0.00031976	0.413078608	0.486	0.515	1	10
CHMP4A	0.000337988	0.413751386	0.335	0.271	1	10
BRD2	0.000340225	0.463967244	0.503	0.536	1	10
CDC42	0.000343317	0.362508508	0.568	0.617	1	10
CIRBP	0.000346732	0.340050272	0.541	0.577	1	10
MXD1	0.000388056	0.523625259	0.281	0.211	1	10
CYTH4	0.00046296	0.394717714	0.254	0.185	1	10
FKBP11	0.000531141	0.420891718	0.276	0.212	1	10
STK17A	0.000633244	0.407005091	0.314	0.248	1	10
APOBEC3C	0.000667451	0.388686285	0.254	0.181	1	10
ORAI1	0.000671115	0.491250208	0.281	0.225	1	10
IER5	0.000718646	0.265951099	0.541	0.474	1	10
TSC22D4	0.00073381	0.395722227	0.292	0.233	1	10
FERMT3	0.000805363	0.347142502	0.319	0.254	1	10
PRKACB	0.000817723	0.389189114	0.254	0.195	1	10
WAS	0.000956095	0.334250298	0.259	0.197	1	10
ILF3-DT	0.00096956	0.459510635	0.303	0.247	1	10
LYST	0.001252511	0.429411359	0.297	0.242	1	10
SSR2	0.001272131	0.295108341	0.562	0.636	1	10
CEMIP2	0.001458038	0.494283592	0.303	0.257	1	10
UPP1	0.001946014	0.429509315	0.27	0.219	1	10
PNISR	0.002016467	0.367118178	0.524	0.574	1	10
RAB11FIP1	0.002224257	0.457828398	0.308	0.263	1	10
YME1L1	0.002225943	0.413606411	0.459	0.48	1	10
OPTN	0.002321165	0.364013203	0.303	0.253	1	10
TECR	0.002558829	0.478210826	0.432	0.468	1	10
TNFRSF1B	0.003013287	0.275508514	0.362	0.305	1	10
PLA2G16	0.003132726	0.435290364	0.265	0.218	1	10
UBXN1	0.003138931	0.398644863	0.459	0.505	1	10

SH3KBP1	0.003280407	0.387912648	0.292	0.242	1	10
KDM6B	0.003705935	0.413645558	0.292	0.241	1	10
IGF2R	0.003742614	0.590122904	0.308	0.285	1	10
MIEN1	0.00380348	0.487234188	0.33	0.313	1	10
DDIT3	0.003876611	0.470770728	0.341	0.308	1	10
CSK	0.004119562	0.437928047	0.346	0.324	1	10
MBNL1	0.004516946	0.429077638	0.443	0.471	1	10
UBE2F	0.004613145	0.479059707	0.341	0.334	1	10
GLRX	0.004753108	0.405685896	0.405	0.413	1	10
DNAJB6	0.0051295	0.332990922	0.497	0.55	1	10
STOM	0.005348723	0.407016016	0.373	0.376	1	10
FNIP1	0.005415191	0.485200301	0.324	0.308	1	10
CYTH1	0.006168452	0.463699235	0.308	0.286	1	10
KLF6	0.006641239	0.267392437	0.632	0.652	1	10
DNAJA4	0.006883373	0.403290976	0.281	0.221	1	10
ARID4B	0.007819296	0.519584443	0.405	0.438	1	10
TFDP2	0.00794509	0.470730378	0.265	0.234	1	10
CASP8	0.008966691	0.460075211	0.286	0.258	1	10
BSG	0.008976954	0.329963974	0.465	0.528	1	10
IDI1	0.009467799	0.343106886	0.465	0.492	1	10
ICAM3	0.009543611	0.40840835	0.308	0.276	1	10
RGS5	0	3.417046016	0.964	0.052	0	11
THY1	0	2.546965917	0.825	0.031	0	11
COL6A2	0	2.534793834	0.803	0.036	0	11
COL3A1	0	2.132988283	0.715	0.007	0	11
COL6A1	0	2.08436916	0.766	0.027	0	11
C11orf96	0	2.051245508	0.774	0.037	0	11
COL1A2	0	1.942866542	0.715	0.007	0	11
NOTCH3	0	1.851114978	0.701	0.012	0	11
COL6A3	0	1.849962401	0.628	0.01	0	11
TBX2	0	1.713309164	0.664	0.008	0	11
HIGD1B	0	1.697365604	0.701	0.007	0	11
CARMN	0	1.645814829	0.577	0.002	0	11
MFGE8	0	1.631897354	0.708	0.031	0	11
THBS4	0	1.560534591	0.467	0.006	0	11
CYGB	0	1.502578692	0.65	0.017	0	11
FAM162B	0	1.474507884	0.613	0.005	0	11
COX4I2	0	1.408552002	0.599	0.004	0	11
GUCY1A2	0	1.406479331	0.591	0.003	0	11
LHFPL6	0	1.392844108	0.62	0.018	0	11
MAP1B	0	1.3819887	0.65	0.022	0	11
TGFB1I1	0	1.358001357	0.635	0.016	0	11
CD248	0	1.349074535	0.62	0.004	0	11
PLAC9	0	1.323669574	0.569	0.005	0	11
GJA4	0	1.275316327	0.569	0.007	0	11

TPPP3	0	1.249659697	0.526	0.01	0	11
FRZB	0	1.173745237	0.577	0.005	0	11
CSPG4	0	1.173101207	0.496	0.006	0	11
PLXDC1	0	1.14708168	0.496	0.009	0	11
GJC1	0	1.093499356	0.504	0.005	0	11
COL5A3	0	1.074394492	0.467	0.004	0	11
EBF1	0	1.074085518	0.547	0.01	0	11
EBF2	0	1.005730743	0.496	0.003	0	11
CCDC3	0	0.994194522	0.518	0.004	0	11
ARHGEF17	0	0.883074258	0.489	0.009	0	11
EDNRA	0	0.846008771	0.387	0.002	0	11
SFTA1P	0	0.832129759	0.365	0.003	0	11
OLFML2A	0	0.81391004	0.453	0.005	0	11
PRKG1	0	0.779350216	0.453	0.002	0	11
SEMA5A	0	0.753827116	0.35	0.003	0	11
POSTN	0	0.720694352	0.314	0.002	0	11
NFASC	0	0.678834826	0.314	0	0	11
EGFLAM	0	0.621856235	0.358	0.002	0	11
SEMA5B	0	0.620491561	0.314	0.002	0	11
RAB6B	0	0.585958048	0.314	0.002	0	11
PDGFRB	5.45E-306	2.397389084	0.766	0.04	1.12E-301	11
CDH6	1.11E-301	0.460258213	0.277	0.001	2.30E-297	11
LINGO1	9.72E-295	0.548820038	0.277	0.001	2.00E-290	11
AKAP12	6.06E-292	0.630025817	0.387	0.006	1.25E-287	11
ADGRF5	1.85E-289	1.104624027	0.584	0.02	3.82E-285	11
S1PR3	4.45E-289	0.884232454	0.38	0.006	9.16E-285	11
RASL12	4.58E-284	0.504551752	0.299	0.002	9.43E-280	11
NID2	1.70E-281	0.969302143	0.482	0.012	3.51E-277	11
FABP4	7.67E-281	2.847445798	0.839	0.056	1.58E-276	11
ACTA2	8.46E-278	2.497484861	0.708	0.037	1.74E-273	11
ABCC9	3.04E-274	1.452212545	0.547	0.019	6.25E-270	11
C1QTNF1	5.74E-269	0.519959822	0.277	0.002	1.18E-264	11
BGN	7.00E-269	0.753618602	0.35	0.005	1.44E-264	11
GUCY1A1	9.91E-267	1.299069029	0.584	0.024	2.04E-262	11
COL4A2	3.80E-260	2.317166476	0.766	0.049	7.83E-256	11
TINAGL1	1.41E-253	1.075956935	0.482	0.015	2.91E-249	11
COL1A1	6.46E-249	1.547440048	0.387	0.008	1.33E-244	11
TPM2	3.26E-248	2.172011082	0.81	0.062	6.72E-244	11
TCIM	3.54E-245	1.054614497	0.489	0.016	7.29E-241	11
ANO1	8.77E-245	0.634847226	0.358	0.007	1.81E-240	11
SSTR2	5.36E-242	0.578244192	0.321	0.005	1.10E-237	11
PPP1R14A	2.78E-240	1.081653608	0.518	0.02	5.73E-236	11
CCDC102B	9.09E-240	1.258469313	0.577	0.027	1.87E-235	11
ADGRA2	1.80E-235	0.696970113	0.35	0.007	3.71E-231	11
MCAM	6.47E-235	1.407840273	0.635	0.034	1.33E-230	11

SPARCL1	1.69E-234	1.794409685	0.657	0.038	3.48E-230	11
ADAMTS4	1.03E-233	1.044049794	0.438	0.013	2.11E-229	11
COL4A1	1.07E-218	2.287492755	0.723	0.053	2.20E-214	11
FILIP1	1.39E-213	0.822883114	0.445	0.016	2.87E-209	11
RBMS3	1.00E-212	0.619269448	0.358	0.009	2.07E-208	11
EHD2	4.54E-211	0.854445887	0.482	0.02	9.35E-207	11
LAMA4	1.36E-202	1.146915989	0.533	0.027	2.80E-198	11
AOC3	7.47E-199	0.667736793	0.387	0.012	1.54E-194	11
ITGA7	1.05E-197	0.468806034	0.285	0.005	2.16E-193	11
MGP	3.20E-197	1.177890738	0.409	0.014	6.59E-193	11
FHL1	1.73E-194	1.009794535	0.482	0.023	3.57E-190	11
ADIRF	9.67E-194	1.478188468	0.496	0.025	1.99E-189	11
NDUFA4L2	5.17E-193	2.39380593	0.869	0.103	1.07E-188	11
GUCY1B1	2.20E-192	1.13974355	0.518	0.028	4.54E-188	11
CPE	2.92E-192	2.065884595	0.737	0.069	6.01E-188	11
ENPEP	1.06E-191	1.232983028	0.547	0.033	2.19E-187	11
KCNJ8	2.35E-191	1.697968045	0.693	0.06	4.84E-187	11
GNG12	3.98E-186	0.536183848	0.343	0.01	8.19E-182	11
LOXL2	1.77E-180	0.742090776	0.387	0.014	3.64E-176	11
PMEPA1	1.21E-178	1.068843941	0.474	0.025	2.49E-174	11
SPARC	2.76E-176	2.798627529	0.912	0.131	5.69E-172	11
COX7A1	2.13E-174	0.650753909	0.365	0.013	4.39E-170	11
STEAP4	2.17E-174	0.687065477	0.314	0.009	4.47E-170	11
PXDN	9.06E-174	0.675365268	0.387	0.015	1.87E-169	11
NR2F1	3.96E-169	0.493551917	0.263	0.006	8.16E-165	11
ADAMTS1	1.36E-164	1.232630939	0.438	0.022	2.81E-160	11
FJX1	3.38E-164	0.664846704	0.27	0.006	6.97E-160	11
FSTL1	2.50E-163	0.63125731	0.35	0.013	5.15E-159	11
DAAM2	3.89E-163	0.636005	0.387	0.017	8.00E-159	11
IGFBP7	8.55E-163	2.953195058	0.912	0.146	1.76E-158	11
NES	9.66E-163	0.842344235	0.401	0.018	1.99E-158	11
ANTXR1	3.30E-162	0.425268084	0.292	0.008	6.81E-158	11
PRSS23	4.15E-158	0.872710294	0.46	0.026	8.54E-154	11
CYR61	1.19E-155	0.980741757	0.365	0.016	2.45E-151	11
AC100793.2	1.04E-153	0.554466346	0.285	0.008	2.14E-149	11
MSRB3	2.79E-153	0.517488222	0.285	0.008	5.75E-149	11
CRISPLD2	7.68E-150	0.769981404	0.387	0.019	1.58E-145	11
CRIP2	7.10E-148	1.143112448	0.628	0.057	1.46E-143	11
ESAM	1.14E-147	0.85075083	0.46	0.028	2.34E-143	11
CAV2	7.42E-146	0.733668836	0.445	0.027	1.53E-141	11
CAV1	7.89E-140	1.28040958	0.599	0.056	1.63E-135	11
TAGLN	1.28E-138	2.13978134	0.547	0.049	2.63E-134	11
BTNL9	1.04E-132	0.537021734	0.314	0.013	2.15E-128	11
TMEM74B	1.81E-128	0.538377269	0.321	0.015	3.73E-124	11
PGF	3.23E-128	0.640773449	0.387	0.023	6.65E-124	11

FKBP10	3.75E-128	0.524165944	0.263	0.009	7.72E-124	11
FRMD3	5.79E-128	0.655031494	0.328	0.016	1.19E-123	11
ANGPT2	4.99E-127	0.741070834	0.409	0.026	1.03E-122	11
DDR2	1.02E-120	0.581766111	0.358	0.021	2.11E-116	11
PLAT	1.95E-118	0.691136249	0.277	0.012	4.01E-114	11
CCR10	2.54E-118	0.478789581	0.277	0.012	5.23E-114	11
SEPT4	4.44E-115	0.960760866	0.518	0.054	9.14E-111	11
HOXB3	5.11E-111	0.314588264	0.255	0.01	1.05E-106	11
PHLDB1	1.81E-109	0.657578312	0.401	0.03	3.73E-105	11
NEURL1B	2.62E-108	0.502594809	0.299	0.016	5.40E-104	11
LAMB1	6.83E-108	0.78569131	0.372	0.026	1.41E-103	11
TDO2	2.59E-105	0.70062311	0.299	0.017	5.33E-101	11
PLCE1	2.72E-104	0.672153226	0.401	0.033	5.60E-100	11
EDNRB	4.34E-104	0.624231272	0.453	0.041	8.93E-100	11
SGCB	1.25E-102	0.452202245	0.263	0.012	2.56E-98	11
GRK5	1.29E-102	0.801662706	0.467	0.046	2.65E-98	11
VSTM4	1.64E-98	0.659754056	0.292	0.017	3.38E-94	11
LRRC32	3.37E-98	0.483937311	0.292	0.017	6.94E-94	11
CD36	8.30E-98	2.291752329	0.825	0.21	1.71E-93	11
ADAMTS9	2.06E-97	0.708737803	0.38	0.031	4.25E-93	11
MYL9	1.48E-96	1.82332639	0.818	0.201	3.04E-92	11
FILIP1L	4.64E-96	1.264056983	0.562	0.079	9.55E-92	11
TPM1	1.66E-93	2.00069929	0.869	0.262	3.42E-89	11
FAT1	1.73E-93	0.470901054	0.299	0.02	3.56E-89	11
NR2F2	2.20E-93	1.778851943	0.81	0.211	4.53E-89	11
IFI27	1.08E-89	1.321083113	0.526	0.067	2.23E-85	11
TMEM47	6.86E-89	0.344834765	0.27	0.016	1.41E-84	11
SMTN	7.18E-89	0.966595197	0.511	0.067	1.48E-84	11
GNG11	3.68E-87	0.793750945	0.489	0.057	7.58E-83	11
CAVIN3	4.70E-85	0.659866706	0.343	0.029	9.68E-81	11
JAG1	1.80E-83	0.80780308	0.409	0.043	3.71E-79	11
SYTL2	2.48E-81	0.62502599	0.336	0.029	5.11E-77	11
NDN	2.69E-81	0.610480191	0.372	0.038	5.55E-77	11
CLEC11A	5.74E-81	0.809128709	0.482	0.065	1.18E-76	11
OLFML2B	9.65E-81	0.773376444	0.387	0.041	1.99E-76	11
TESC	2.01E-80	0.91474742	0.511	0.071	4.14E-76	11
SLC12A2	2.12E-80	0.974732883	0.453	0.057	4.36E-76	11
HES4	3.24E-75	0.999704844	0.569	0.098	6.67E-71	11
FBLIM1	4.07E-73	0.489264609	0.314	0.029	8.38E-69	11
PARVA	7.48E-73	0.630062917	0.35	0.037	1.54E-68	11
F2R	1.47E-72	0.496154251	0.358	0.036	3.03E-68	11
HIC1	2.81E-72	0.886971415	0.431	0.056	5.79E-68	11
EMILIN1	1.55E-71	0.67331545	0.314	0.03	3.20E-67	11
ITGA1	1.75E-70	1.392967139	0.686	0.177	3.60E-66	11
CALD1	1.34E-69	1.832323367	0.898	0.408	2.76E-65	11

PLCL1	1.68E-69	0.463136645	0.299	0.027	3.46E-65	11
APOLD1	2.01E-69	0.69369162	0.431	0.057	4.15E-65	11
PDLIM3	7.46E-69	0.744624254	0.321	0.032	1.54E-64	11
CTGF	3.53E-68	1.280333657	0.46	0.072	7.28E-64	11
FXYD6	2.13E-67	0.8823382	0.467	0.072	4.38E-63	11
CAVIN1	8.77E-60	0.619140868	0.343	0.041	1.81E-55	11
EPB41L1	1.40E-59	0.774237401	0.445	0.076	2.88E-55	11
LGALS1	1.36E-56	1.662435182	0.964	0.586	2.80E-52	11
IGFBP5	1.75E-56	0.679631995	0.343	0.044	3.60E-52	11
ARHGAP42	2.81E-55	0.487974912	0.336	0.045	5.78E-51	11
MEF2C	3.60E-55	1.253099673	0.664	0.191	7.42E-51	11
MYLK	6.41E-55	0.871338711	0.453	0.086	1.32E-50	11
TUBA1A	1.80E-54	1.220857686	0.818	0.291	3.70E-50	11
COL18A1	3.91E-54	1.831246182	0.737	0.285	8.06E-50	11
SOX4	7.73E-54	0.936355326	0.577	0.133	1.59E-49	11
SERPINH1	8.99E-52	1.195845874	0.686	0.218	1.85E-47	11
UACA	1.04E-49	1.458209804	0.591	0.176	2.14E-45	11
LGALS3BP	3.66E-49	1.075332523	0.562	0.146	7.53E-45	11
BMP1	3.77E-48	0.837864548	0.438	0.09	7.77E-44	11
PTK2	1.86E-47	0.983882927	0.569	0.162	3.83E-43	11
VCL	3.26E-45	0.832364884	0.474	0.109	6.71E-41	11
MPRIP	6.64E-45	0.804432652	0.547	0.145	1.37E-40	11
TMEM204	9.62E-45	0.341193576	0.255	0.03	1.98E-40	11
A2M	5.85E-44	1.322696902	0.723	0.283	1.20E-39	11
DOCK6	1.16E-43	0.536916921	0.321	0.05	2.38E-39	11
SEPT11	1.43E-43	1.34511596	0.701	0.313	2.94E-39	11
TPM4	1.48E-43	1.156249007	0.796	0.367	3.04E-39	11
SPRY1	2.60E-43	0.714245895	0.431	0.091	5.35E-39	11
TIMP1	3.88E-42	1.442331426	0.752	0.304	8.00E-38	11
TUSC3	1.89E-41	0.469779019	0.307	0.049	3.88E-37	11
VIM	7.56E-41	1.088434429	0.985	0.571	1.56E-36	11
CHSY1	1.52E-40	0.46008583	0.35	0.061	3.13E-36	11
MYO1B	8.39E-40	1.090715001	0.606	0.216	1.73E-35	11
AMOTL1	2.19E-38	0.394750256	0.255	0.036	4.51E-34	11
DLC1	3.21E-38	0.917932191	0.547	0.176	6.62E-34	11
HOXB2	4.11E-38	0.33989291	0.328	0.055	8.47E-34	11
EPS8	1.05E-37	1.216142309	0.584	0.204	2.16E-33	11
SPECC1	1.14E-37	0.565063613	0.321	0.058	2.36E-33	11
GEM	1.86E-37	0.475936706	0.343	0.061	3.82E-33	11
NCK2	3.03E-37	0.588795875	0.394	0.08	6.24E-33	11
TCF4	3.10E-37	0.95944587	0.65	0.26	6.39E-33	11
CD81	3.66E-37	1.105024566	0.796	0.402	7.55E-33	11
SEPT7	4.85E-37	1.10757058	0.839	0.561	9.98E-33	11
FABP5	6.07E-37	0.935961538	0.847	0.396	1.25E-32	11
KANK2	7.24E-37	0.582314716	0.365	0.077	1.49E-32	11

IFITM3	2.28E-36	1.136777493	0.898	0.647	4.70E-32	11
COL5A2	3.13E-36	0.92179062	0.526	0.168	6.44E-32	11
ASAP2	8.45E-36	0.496270952	0.314	0.058	1.74E-31	11
MAP1LC3A	1.02E-35	0.667008822	0.394	0.088	2.11E-31	11
SLCO3A1	1.76E-35	0.535535828	0.409	0.094	3.62E-31	11
COLEC11	3.42E-35	0.967730878	0.387	0.092	7.03E-31	11
CTBP2	4.16E-35	0.545548663	0.431	0.103	8.57E-31	11
RHBDF1	5.51E-35	0.392921608	0.255	0.04	1.14E-30	11
MYOF	7.95E-35	0.460354992	0.314	0.058	1.64E-30	11
FLNA	2.01E-34	1.104599295	0.664	0.268	4.15E-30	11
TNS1	7.42E-34	0.783155542	0.555	0.191	1.53E-29	11
GSN	1.32E-33	0.961247449	0.701	0.297	2.72E-29	11
TNKS1BP1	1.76E-33	0.567887667	0.321	0.066	3.63E-29	11
ARHGAP26	2.96E-32	0.675221409	0.423	0.112	6.09E-28	11
DCBLD1	3.14E-32	0.47327088	0.292	0.055	6.47E-28	11
ITGB1	8.99E-32	1.246000636	0.788	0.551	1.85E-27	11
CSRP1	1.45E-31	0.701335048	0.438	0.119	2.98E-27	11
RCN1	2.18E-31	0.58284225	0.372	0.088	4.48E-27	11
SLC38A11	2.01E-30	0.592646481	0.255	0.046	4.14E-26	11
MARCKSL1	3.71E-30	1.117121256	0.686	0.346	7.64E-26	11
ID3	4.56E-30	0.948268914	0.467	0.138	9.39E-26	11
ADCY3	7.06E-30	0.466214711	0.285	0.055	1.45E-25	11
CD63	8.28E-30	0.827946664	0.891	0.738	1.71E-25	11
IGFBP4	1.72E-29	0.911133572	0.628	0.281	3.53E-25	11
RGS16	3.64E-29	0.561913635	0.314	0.065	7.50E-25	11
APP	3.81E-29	0.740161623	0.54	0.205	7.86E-25	11
PTP4A3	8.81E-29	0.304528482	0.277	0.051	1.81E-24	11
FERMT2	9.56E-29	0.858819009	0.504	0.185	1.97E-24	11
RHOC	6.80E-28	0.941723942	0.745	0.462	1.40E-23	11
KIAA0040	1.16E-27	0.387133452	0.307	0.064	2.39E-23	11
MIR4435-2HG	1.21E-27	0.909709925	0.577	0.236	2.49E-23	11
INAFM1	1.43E-27	0.45737049	0.299	0.066	2.94E-23	11
TUBB6	6.82E-27	0.564198394	0.314	0.073	1.40E-22	11
RRAS	7.52E-27	0.863092404	0.46	0.153	1.55E-22	11
PTMS	1.80E-26	0.979200032	0.803	0.557	3.71E-22	11
EFEMP2	1.84E-26	0.410651327	0.35	0.093	3.79E-22	11
MYH9	4.59E-26	1.014025623	0.752	0.522	9.45E-22	11
FAM241A	5.82E-26	0.590931115	0.394	0.115	1.20E-21	11
APBB2	5.93E-26	0.376875271	0.255	0.052	1.22E-21	11
FBLN1	8.38E-26	0.773184322	0.416	0.131	1.73E-21	11
PPFIBP1	8.67E-26	0.395962399	0.321	0.08	1.79E-21	11
CNN3	2.86E-25	0.936967178	0.599	0.276	5.88E-21	11
ITM2C	4.78E-25	0.910393525	0.584	0.267	9.85E-21	11
ADAP2	1.49E-24	0.633229317	0.489	0.168	3.08E-20	11

TRIB2	3.25E-24	0.298535165	0.292	0.067	6.70E-20	11
PELO	3.77E-24	0.53769208	0.387	0.119	7.76E-20	11
NID1	6.52E-24	0.831192857	0.547	0.248	1.34E-19	11
CCND1	2.36E-23	1.073949305	0.555	0.263	4.86E-19	11
DMD	4.45E-23	0.493250926	0.292	0.074	9.16E-19	11
LIMA1	5.97E-23	0.694808407	0.416	0.139	1.23E-18	11
OSBPL1A	1.26E-22	0.376464955	0.299	0.076	2.59E-18	11
KLHL23	2.23E-22	0.789920309	0.453	0.174	4.60E-18	11
PLS3	2.77E-22	0.62808148	0.438	0.164	5.71E-18	11
EVA1B	4.22E-22	0.749476102	0.511	0.22	8.69E-18	11
RNF152	7.36E-22	0.654173789	0.38	0.129	1.52E-17	11
SASH1	7.75E-22	0.437439114	0.358	0.105	1.60E-17	11
TMEM273	8.56E-22	0.405635337	0.27	0.064	1.76E-17	11
MTUS1	4.65E-21	0.64931983	0.482	0.2	9.57E-17	11
CD151	8.23E-21	0.781792452	0.511	0.23	1.69E-16	11
TNFRSF21	1.01E-20	0.429249068	0.321	0.093	2.07E-16	11
LAMC1	1.30E-20	0.667631233	0.431	0.168	2.69E-16	11
ITPRIPL2	1.46E-20	0.425085018	0.343	0.104	3.00E-16	11
OAZ2	1.71E-20	0.705248724	0.635	0.351	3.52E-16	11
SELENOW	3.80E-20	0.773408337	0.693	0.431	7.83E-16	11
AXL	7.49E-20	0.439526157	0.314	0.089	1.54E-15	11
NRP1	9.74E-20	0.595350653	0.489	0.212	2.01E-15	11
NDRG2	1.62E-19	0.751372678	0.504	0.233	3.34E-15	11
FHOD1	2.08E-19	0.28801735	0.27	0.069	4.29E-15	11
PEAK1	2.21E-19	0.376748748	0.321	0.097	4.55E-15	11
PPP1R12B	4.56E-19	0.581808057	0.328	0.106	9.39E-15	11
PPIC	6.16E-19	0.463543061	0.365	0.129	1.27E-14	11
PDLIM7	1.03E-18	0.471868093	0.285	0.08	2.11E-14	11
CCL2	1.74E-18	0.673612259	0.27	0.071	3.58E-14	11
CDC42BPA	3.56E-18	0.585534666	0.409	0.162	7.33E-14	11
PLEKHH3	4.60E-18	0.531250992	0.321	0.105	9.48E-14	11
CYBRD1	5.35E-18	0.32790255	0.27	0.073	1.10E-13	11
CALM2	1.25E-17	0.601569823	0.876	0.763	2.58E-13	11
MGST3	1.41E-17	0.866335588	0.723	0.553	2.91E-13	11
PIEZO2	1.53E-17	0.546409134	0.358	0.131	3.16E-13	11
PDLIM1	2.02E-17	0.806685965	0.628	0.349	4.15E-13	11
TAX1BP3	2.43E-17	0.389406113	0.321	0.102	5.01E-13	11
MXRA7	3.64E-17	0.372454656	0.285	0.085	7.49E-13	11
CD59	4.28E-17	0.727907606	0.599	0.339	8.82E-13	11
INF2	7.94E-17	0.500787737	0.372	0.14	1.63E-12	11
ZEB2	1.54E-16	0.564167831	0.547	0.249	3.17E-12	11
DSTN	1.63E-16	0.806511302	0.672	0.474	3.36E-12	11
TIMP3	6.33E-16	0.836096349	0.533	0.285	1.30E-11	11
C12orf57	6.66E-16	0.742635071	0.664	0.426	1.37E-11	11
SPON2	8.28E-16	0.820175033	0.547	0.296	1.71E-11	11

CCND2	1.22E-15	0.373946161	0.445	0.172	2.50E-11	11
ARHGEF12	1.25E-15	0.552935093	0.46	0.223	2.57E-11	11
CYSTM1	1.27E-15	0.797517498	0.555	0.32	2.62E-11	11
RBPMS	1.71E-15	0.608321278	0.416	0.191	3.53E-11	11
SELENOM	1.87E-15	0.667040584	0.555	0.297	3.85E-11	11
LDHB	2.27E-15	0.629944831	0.628	0.353	4.68E-11	11
LAMB2	2.28E-15	0.629663311	0.496	0.253	4.70E-11	11
CLMN	2.36E-15	0.538027256	0.321	0.119	4.86E-11	11
ATN1	2.63E-15	0.370410925	0.299	0.099	5.41E-11	11
PLK2	3.47E-15	0.392888249	0.307	0.101	7.14E-11	11
MYL6	3.74E-15	0.66097698	0.898	0.856	7.70E-11	11
MT2A	4.91E-15	0.287213158	0.781	0.585	1.01E-10	11
STOM	5.36E-15	0.726448761	0.584	0.371	1.10E-10	11
MAGED2	1.09E-14	0.759756778	0.511	0.285	2.25E-10	11
CYTOR	1.31E-14	0.763988307	0.62	0.396	2.69E-10	11
ZEB1	1.32E-14	0.321959205	0.35	0.134	2.73E-10	11
ARHGDIB	1.67E-14	0.505573806	0.847	0.506	3.44E-10	11
ANXA6	2.83E-14	0.77210765	0.65	0.438	5.83E-10	11
ARMCX3	7.42E-14	0.434936205	0.401	0.167	1.53E-09	11
LMO4	1.04E-13	0.417841664	0.445	0.192	2.14E-09	11
RASAL2	1.06E-13	0.374103476	0.307	0.115	2.18E-09	11
CLIC4	1.11E-13	0.563079773	0.431	0.211	2.28E-09	11
LBH	1.22E-13	0.399606299	0.394	0.148	2.51E-09	11
TSC22D1	1.48E-13	0.623760578	0.533	0.312	3.05E-09	11
TRAK1	1.63E-13	0.370967685	0.314	0.121	3.35E-09	11
GNAS	1.85E-13	0.492358701	0.861	0.762	3.80E-09	11
DCBLD2	2.96E-13	0.529474321	0.365	0.164	6.09E-09	11
C1orf54	4.91E-13	0.274670519	0.365	0.141	1.01E-08	11
CAMK2N1	6.11E-13	0.764789055	0.555	0.322	1.26E-08	11
HIP1	8.40E-13	0.317211931	0.285	0.102	1.73E-08	11
FAM213A	1.00E-12	0.637676246	0.599	0.384	2.06E-08	11
MT-CO3	1.07E-12	0.434670321	1	1	2.20E-08	11
PLXND1	1.35E-12	0.547115609	0.423	0.208	2.79E-08	11
IFITM1	1.35E-12	0.326422479	0.277	0.09	2.79E-08	11
ECE1	1.70E-12	0.419948426	0.38	0.172	3.49E-08	11
BEX3	1.84E-12	0.549802536	0.511	0.292	3.79E-08	11
DGKH	1.91E-12	0.38451746	0.38	0.169	3.93E-08	11
RBP1	2.33E-12	0.514473679	0.328	0.138	4.80E-08	11
SERPING1	3.30E-12	0.655912767	0.664	0.441	6.80E-08	11
CSNK1E	3.44E-12	0.55247772	0.445	0.24	7.09E-08	11
PHLDA1	3.91E-12	0.448334255	0.526	0.281	8.05E-08	11
MT-ATP6	4.30E-12	0.407978909	1	1	8.85E-08	11
YBX3	5.15E-12	0.530019009	0.46	0.228	1.06E-07	11
NFIB	5.85E-12	0.523357976	0.401	0.2	1.20E-07	11
VASN	7.03E-12	0.360076015	0.27	0.104	1.45E-07	11

EPAS1	8.15E-12	0.521378457	0.489	0.282	1.68E-07	11
IFIT3	1.25E-11	0.408059456	0.27	0.101	2.57E-07	11
EFHD1	1.30E-11	0.487991488	0.314	0.136	2.67E-07	11
S100A11	1.39E-11	0.384281762	0.796	0.49	2.85E-07	11
PTPRK	1.54E-11	0.379133483	0.343	0.154	3.17E-07	11
NBEAL1	3.05E-11	0.51426731	0.431	0.231	6.29E-07	11
TNS2	3.84E-11	0.474368742	0.277	0.11	7.91E-07	11
MYADM	4.04E-11	0.348911623	0.453	0.226	8.32E-07	11
PLAU	4.15E-11	0.330020611	0.263	0.093	8.54E-07	11
IFIT2	4.80E-11	0.363782978	0.255	0.094	9.88E-07	11
PAG1	5.57E-11	0.364431679	0.394	0.176	1.15E-06	11
PKIG	1.10E-10	0.47823981	0.401	0.203	2.27E-06	11
RABGAP1	1.14E-10	0.413419754	0.336	0.155	2.36E-06	11
MAP4K4	1.26E-10	0.59872311	0.46	0.274	2.59E-06	11
ACADVL	1.45E-10	0.57877859	0.606	0.443	2.98E-06	11
ISYNA1	1.54E-10	0.557532313	0.299	0.132	3.18E-06	11
SH3BGRL	1.87E-10	0.456138042	0.569	0.328	3.84E-06	11
ARHGAP29	1.90E-10	0.500839505	0.394	0.205	3.92E-06	11
ZMAT3	2.17E-10	0.563650872	0.372	0.185	4.47E-06	11
TSPAN3	2.21E-10	0.328620919	0.365	0.177	4.56E-06	11
UBA2	2.51E-10	0.528116123	0.504	0.317	5.16E-06	11
RSU1	3.53E-10	0.564445606	0.467	0.294	7.27E-06	11
SLC25A6	4.42E-10	0.767020151	0.715	0.628	9.11E-06	11
SLC43A3	4.76E-10	0.403583185	0.358	0.173	9.80E-06	11
TAGLN2	5.52E-10	0.569868404	0.642	0.468	1.14E-05	11
PPARG	5.86E-10	0.593287852	0.409	0.233	1.21E-05	11
SPTBN1	5.92E-10	0.388280113	0.606	0.356	1.22E-05	11
MAP3K20	6.33E-10	0.577886291	0.409	0.237	1.30E-05	11
ARHGEF7	7.16E-10	0.437127616	0.35	0.17	1.47E-05	11
RAPGEF2	8.07E-10	0.293582875	0.299	0.131	1.66E-05	11
TCEAL9	8.34E-10	0.506680408	0.35	0.176	1.72E-05	11
AGRN	1.08E-09	0.387217363	0.314	0.146	2.23E-05	11
ATP2B4	1.26E-09	0.253603546	0.299	0.125	2.60E-05	11
LMNA	2.05E-09	0.463562949	0.686	0.517	4.22E-05	11
ASAH1	2.84E-09	0.458360048	0.642	0.467	5.84E-05	11
CRTC3	3.03E-09	0.392118887	0.328	0.162	6.25E-05	11
ST5	3.16E-09	0.314847711	0.255	0.107	6.51E-05	11
GAPDH	4.11E-09	0.391543511	0.942	0.919	8.47E-05	11
ROCK2	4.17E-09	0.438772789	0.445	0.276	8.59E-05	11
MARCKS	4.18E-09	0.304168083	0.73	0.541	8.62E-05	11
PACSIN3	4.63E-09	0.436823198	0.292	0.136	9.54E-05	11
ABI2	4.70E-09	0.441120432	0.416	0.246	9.67E-05	11
PLOD1	4.76E-09	0.541511887	0.343	0.179	9.79E-05	11
OPTN	7.36E-09	0.398766456	0.445	0.25	0.000151554	11
LEPROT	7.74E-09	0.515918543	0.562	0.432	0.000159471	11

RAPH1	1.59E-08	0.390210579	0.358	0.198	0.000327702	11
SH3PXD2A	1.66E-08	0.408171182	0.314	0.16	0.000341266	11
KLHDC8B	1.70E-08	0.317379566	0.263	0.116	0.000349378	11
EID1	2.03E-08	0.569072863	0.628	0.554	0.000417203	11
CHD9	2.15E-08	0.416792808	0.489	0.322	0.000442954	11
RBFOX2	2.67E-08	0.336738348	0.328	0.169	0.000550441	11
LASP1	3.33E-08	0.364688362	0.496	0.321	0.000684926	11
PAWR	4.12E-08	0.471173874	0.365	0.207	0.000848818	11
CCDC92	5.30E-08	0.306922483	0.255	0.112	0.00109207	11
CAPN2	5.49E-08	0.394354442	0.314	0.148	0.001130853	11
PPP1R12A	5.51E-08	0.499889703	0.555	0.419	0.001134787	11
DYNC1I2	7.21E-08	0.55220798	0.504	0.361	0.001484722	11
FN1	7.62E-08	0.461818074	0.745	0.534	0.001569705	11
NREP	7.89E-08	0.4233661	0.27	0.129	0.00162529	11
TMEM98	9.94E-08	0.294101062	0.263	0.124	0.002046529	11
AP2M1	1.10E-07	0.526445405	0.613	0.516	0.002271698	11
ASAP1	1.13E-07	0.402659997	0.409	0.243	0.00231775	11
TACC1	1.17E-07	0.454422204	0.504	0.326	0.002409738	11
GSTP1	1.35E-07	0.307315856	0.613	0.367	0.002790418	11
C20orf27	1.43E-07	0.483144357	0.467	0.321	0.002948984	11
RAC1	1.77E-07	0.428877537	0.745	0.692	0.003654835	11
DYNLT1	1.82E-07	0.503090798	0.547	0.406	0.003742209	11
DPYSL2	1.92E-07	0.423703493	0.394	0.235	0.003952825	11
SCARB2	2.22E-07	0.429312194	0.504	0.353	0.004562208	11
TMEM165	2.49E-07	0.370811371	0.401	0.237	0.00512689	11
EPB41L2	3.13E-07	0.358181383	0.372	0.211	0.006437444	11
FKBP1A	3.92E-07	0.470834287	0.686	0.64	0.008082881	11
MYO1C	4.28E-07	0.282687021	0.314	0.169	0.008810477	11
MAP4	5.36E-07	0.402939831	0.453	0.314	0.011032372	11
VASP	5.38E-07	0.476756458	0.511	0.35	0.011079268	11
ARHGAP1	5.68E-07	0.293272489	0.307	0.165	0.011701944	11
ANXA5	5.74E-07	0.488268343	0.584	0.431	0.011824226	11
PHC2	6.00E-07	0.463383287	0.409	0.261	0.012360901	11
RUFY3	6.07E-07	0.38365709	0.336	0.189	0.012498086	11
YWHAB	6.25E-07	0.357777986	0.788	0.711	0.012872232	11
BBX	6.59E-07	0.474948775	0.526	0.378	0.01356619	11
RAB31	7.28E-07	0.282676071	0.343	0.181	0.014986981	11
PHGDH	8.53E-07	0.540470759	0.292	0.157	0.017577732	11
CALM1	1.20E-06	0.330744335	0.81	0.739	0.02464918	11
NNT-AS1	1.23E-06	0.25822151	0.255	0.123	0.02535947	11
BST2	1.54E-06	0.334837756	0.518	0.335	0.031686665	11
CYB5R3	1.82E-06	0.526189756	0.467	0.351	0.037382466	11
BSG	2.09E-06	0.389988168	0.613	0.524	0.042980411	11
RPS4Y1	2.18E-06	0.373680379	0.577	0.39	0.044944425	11
NECTIN2	2.63E-06	0.342153535	0.401	0.266	0.054176215	11

DYNLL1	2.99E-06	0.401158822	0.774	0.757	0.061542499	11
CD99	3.53E-06	0.382554739	0.642	0.574	0.072769656	11
RPS27L	3.74E-06	0.611721547	0.672	0.639	0.077114917	11
C9orf3	4.35E-06	0.33427036	0.285	0.158	0.089627913	11
DENND5A	5.16E-06	0.337640137	0.307	0.176	0.106292822	11
TJP1	5.18E-06	0.256656914	0.27	0.146	0.106704618	11
CCNI	5.37E-06	0.380066913	0.686	0.62	0.110512656	11
CTNNA1	6.31E-06	0.37783013	0.467	0.356	0.130033474	11
ADD1	6.40E-06	0.403957854	0.453	0.318	0.131722498	11
ACTG1	6.78E-06	0.285449352	0.912	0.913	0.139624975	11
FARP1	7.11E-06	0.482488797	0.409	0.274	0.146337281	11
RHOQ	7.49E-06	0.353773771	0.365	0.225	0.154297821	11
MYL12A	7.92E-06	0.321161139	0.818	0.811	0.163081373	11
SYNE2	8.87E-06	0.346174091	0.401	0.253	0.182742782	11
UFSP2	9.24E-06	0.296750944	0.277	0.149	0.190242802	11
SRI	9.50E-06	0.365021764	0.453	0.304	0.195750175	11
GBP4	1.04E-05	0.286564653	0.328	0.184	0.213455846	11
REV3L	1.22E-05	0.312991078	0.277	0.154	0.251869693	11
MAP2K1	1.37E-05	0.291191946	0.336	0.201	0.282459462	11
ZSWIM7	1.82E-05	0.33648593	0.292	0.161	0.37432375	11
HERC1	2.01E-05	0.370070588	0.35	0.224	0.413093461	11
ACTR1A	2.13E-05	0.296842366	0.336	0.203	0.438907972	11
GYG1	2.37E-05	0.399251122	0.307	0.182	0.488346923	11
CTTN	2.66E-05	0.411290972	0.336	0.215	0.547132184	11
DYNC1LI2	3.49E-05	0.32001989	0.409	0.294	0.718588745	11
CTNND1	4.25E-05	0.328395205	0.343	0.23	0.876175323	11
ACTN1	4.84E-05	0.494524598	0.431	0.337	0.996910786	11
WDFY2	4.89E-05	0.438438821	0.328	0.207	1	11
SKP1	4.91E-05	0.449729641	0.708	0.677	1	11
NT5DC2	6.11E-05	0.30790522	0.336	0.218	1	11
RUNX1	6.34E-05	0.431584972	0.263	0.151	1	11
TNFRSF1A	6.37E-05	0.346775108	0.431	0.313	1	11
ARHGAP17	6.45E-05	0.310130071	0.27	0.159	1	11
YWHAH	7.26E-05	0.444239492	0.511	0.414	1	11
GNB1	7.28E-05	0.370309922	0.591	0.522	1	11
HP1BP3	8.08E-05	0.283594572	0.54	0.452	1	11
H2AFJ	9.33E-05	0.34320737	0.482	0.389	1	11
SLC25A3	9.59E-05	0.334410389	0.788	0.738	1	11
EIF5A	0.000105627	0.463429487	0.613	0.533	1	11
EMP2	0.000108939	0.328838363	0.336	0.222	1	11
PLXNB2	0.000111073	0.466777234	0.38	0.275	1	11
SUMO2	0.000116212	0.293241173	0.774	0.775	1	11
VGLL4	0.000124204	0.324591184	0.321	0.214	1	11
AP2A1	0.000126371	0.344685396	0.314	0.202	1	11
HCFC1R1	0.000140692	0.316756127	0.336	0.229	1	11

ATP5F1B	0.00015023	0.316587096	0.679	0.643	1	11
EIF4A1	0.000154149	0.371697403	0.569	0.517	1	11
CD9	0.000178821	0.325135645	0.394	0.28	1	11
NENF	0.000204716	0.38809264	0.504	0.401	1	11
ZNF428	0.000225015	0.312455645	0.394	0.277	1	11
TCEAL8	0.000225735	0.401455515	0.387	0.281	1	11
RABAC1	0.000225943	0.422525173	0.533	0.478	1	11
CIRBP	0.000233553	0.358902014	0.628	0.574	1	11
ANXA11	0.000244696	0.383436891	0.526	0.438	1	11
DDX17	0.000264573	0.336729824	0.606	0.548	1	11
CDC42BPB	0.000265513	0.254159159	0.255	0.158	1	11
NBDY	0.000269984	0.260064209	0.328	0.208	1	11
TLN1	0.000279769	0.456186078	0.474	0.4	1	11
MYO6	0.00029339	0.273320862	0.255	0.159	1	11
ZFHX3	0.000301797	0.365013814	0.38	0.287	1	11
TMEM50A	0.000356609	0.339315996	0.569	0.518	1	11
MAP7D1	0.000372742	0.292091203	0.321	0.212	1	11
TAOK1	0.000373394	0.311140663	0.387	0.276	1	11
ATP5ME	0.000408751	0.394308485	0.657	0.607	1	11
CRK	0.000459843	0.317126739	0.343	0.242	1	11
SPTSSA	0.000478853	0.324473106	0.394	0.302	1	11
LUC7L3	0.000572396	0.346684055	0.562	0.487	1	11
RDX	0.000599733	0.391038251	0.482	0.414	1	11
CD40	0.000602014	0.299183147	0.321	0.226	1	11
CADM1	0.000603004	0.315724138	0.292	0.199	1	11
C1R	0.000632313	0.525526124	0.299	0.198	1	11
SSRP1	0.000643162	0.429444654	0.336	0.244	1	11
ARF3	0.000654025	0.287393676	0.416	0.316	1	11
ARL2	0.000673779	0.270354615	0.285	0.182	1	11
PRKAR1A	0.000756229	0.310455204	0.54	0.483	1	11
MRFAP1	0.000816782	0.416126914	0.526	0.475	1	11
PPP2CB	0.000900225	0.394553823	0.401	0.317	1	11
NFIA	0.000914851	0.303634643	0.35	0.251	1	11
SEL1L	0.00098788	0.260770892	0.336	0.236	1	11
PTEN	0.001036175	0.358595499	0.453	0.383	1	11
SEPT2	0.001233086	0.33706377	0.504	0.462	1	11
CDK2AP1	0.001305347	0.274875743	0.255	0.167	1	11
PALLD	0.00137742	0.319429501	0.292	0.2	1	11
RTL8C	0.00143861	0.274737456	0.314	0.225	1	11
ATP5F1D	0.001602397	0.321095766	0.65	0.631	1	11
SORBS3	0.001795412	0.319989144	0.336	0.246	1	11
NCKAP1	0.001880865	0.29301086	0.343	0.246	1	11
CCNG1	0.0019195	0.317286546	0.38	0.289	1	11
TMOD3	0.001943631	0.266548954	0.394	0.3	1	11
VAMP2	0.001979129	0.26164681	0.533	0.449	1	11

MACF1	0.00202464	0.334164465	0.445	0.376	1	11
CALU	0.002084448	0.304440691	0.46	0.398	1	11
MORF4L1	0.002275127	0.39399615	0.613	0.603	1	11
CLIC1	0.002451625	0.282455832	0.745	0.796	1	11
C19orf70	0.002477332	0.399014501	0.489	0.41	1	11
METTL9	0.002498349	0.378134823	0.387	0.301	1	11
HOOK3	0.002584661	0.463508845	0.387	0.315	1	11
NDUFA13	0.003049166	0.251722364	0.628	0.614	1	11
DEGS1	0.003151923	0.265147503	0.343	0.253	1	11
AHCYL1	0.003843762	0.289069493	0.46	0.391	1	11
SEC31A	0.004080232	0.319941811	0.423	0.351	1	11
GBP1	0.00459538	0.361094132	0.387	0.303	1	11
JAK1	0.004676133	0.2901714	0.496	0.458	1	11
ELOB	0.004846304	0.299163935	0.723	0.723	1	11
COBLL1	0.00535899	0.334121829	0.299	0.22	1	11
DLGAP4	0.005715587	0.264856863	0.285	0.206	1	11
PRMT1	0.005918996	0.43314831	0.445	0.376	1	11
CHURC1	0.006187194	0.354301846	0.482	0.428	1	11
RBX1	0.006341936	0.28036724	0.599	0.574	1	11
FBH1	0.006447185	0.278704223	0.255	0.181	1	11
FYTTD1	0.006648869	0.37052076	0.328	0.252	1	11
MALAT1	0.007063919	0.408044056	0.898	0.955	1	11
NDUFB4	0.008235504	0.409484371	0.606	0.597	1	11
ZNF207	0.008312128	0.3172702	0.474	0.422	1	11
TNPO1	0.008881026	0.262184313	0.358	0.287	1	11
EIF3K	0.009180363	0.331498956	0.657	0.669	1	11
DUT	0.009954602	0.341728966	0.409	0.345	1	11
MZB1	0	2.876047154	0.981	0.033	0	12
JCHAIN	0	2.669113127	0.766	0.007	0	12
IGHG2	0	2.452059997	0.692	0.005	0	12
CD79A	0	1.307008534	0.832	0.029	0	12
IGHV4-39	0	0.999069443	0.262	0	0	12
FCRL5	0	0.813398815	0.692	0.006	0	12
JSRP1	0	0.687223473	0.542	0.008	0	12
POU2AF1	0	0.632628638	0.598	0.012	0	12
IGLV3-1	0	0.602918817	0.402	0.001	0	12
TNFRSF17	0	0.529225611	0.551	0.002	0	12
AC012236.1	0	0.40812201	0.467	0.003	0	12
DERL3	4.16E-292	1.549917636	0.85	0.044	8.57E-288	12
IGHA1	1.81E-286	0.835415527	0.495	0.01	3.72E-282	12
FAM30A	5.62E-255	0.669950394	0.374	0.005	1.16E-250	12
IGHG1	7.40E-158	6.317064625	0.981	0.151	1.52E-153	12
ZBP1	1.30E-157	0.292798752	0.449	0.019	2.68E-153	12
CPNE5	3.89E-156	0.396545199	0.43	0.018	8.01E-152	12
IGHG4	3.85E-146	5.366173508	0.981	0.167	7.92E-142	12

IGHG3	4.25E-131	5.046076483	0.869	0.128	8.75E-127	12
TENT5C	1.64E-110	0.706106996	0.701	0.074	3.38E-106	12
FKBP11	5.13E-107	1.529217911	0.944	0.2	1.06E-102	12
KCNN3	5.06E-103	0.274797687	0.327	0.016	1.04E-98	12
IGHM	7.58E-98	2.164755883	0.336	0.019	1.56E-93	12
BHLHE41	4.60E-96	0.45747865	0.383	0.026	9.48E-92	12
CD38	1.83E-67	0.427495965	0.607	0.091	3.77E-63	12
PIM2	4.87E-61	0.70865249	0.645	0.111	1.00E-56	12
SSR4	6.67E-61	2.021606939	0.963	0.672	1.37E-56	12
SPAG4	2.27E-58	0.379698027	0.505	0.078	4.68E-54	12
ITM2C	7.53E-57	1.01703801	0.822	0.264	1.55E-52	12
EAF2	1.20E-56	0.487200724	0.542	0.089	2.48E-52	12
XBP1	1.89E-56	1.38675456	0.972	0.523	3.90E-52	12
ISG20	4.27E-55	0.748351037	0.804	0.182	8.80E-51	12
SLAMF7	8.44E-55	0.412341875	0.598	0.101	1.74E-50	12
GNG7	1.56E-54	0.272048713	0.374	0.044	3.21E-50	12
PRDX4	2.38E-52	1.161897098	0.879	0.377	4.91E-48	12
IGLC3	1.59E-51	4.812581029	0.308	0.033	3.28E-47	12
CD79B	4.76E-51	0.318173992	0.561	0.091	9.81E-47	12
FUT8	4.00E-46	0.273240839	0.336	0.041	8.23E-42	12
HSH2D	2.28E-45	0.298622889	0.308	0.034	4.70E-41	12
SEL1L3	2.98E-44	0.356479819	0.486	0.085	6.15E-40	12
FKBP2	1.92E-42	0.996700547	0.925	0.457	3.96E-38	12
IGKC	5.59E-40	6.693823469	0.869	0.553	1.15E-35	12
TPD52	2.18E-38	0.397212869	0.607	0.162	4.50E-34	12
SEC11C	6.66E-34	0.946234874	0.869	0.473	1.37E-29	12
PLPP5	1.25E-32	0.473390853	0.636	0.204	2.58E-28	12
SPATS2	2.81E-31	0.358913033	0.533	0.146	5.79E-27	12
SELENOS	1.14E-30	0.669427004	0.832	0.389	2.34E-26	12
HERPUD1	1.32E-30	0.983074389	0.935	0.591	2.71E-26	12
SPCS3	1.11E-29	0.736535157	0.85	0.416	2.29E-25	12
NUCB2	5.83E-27	0.357257206	0.729	0.287	1.20E-22	12
HIST1H2BG	2.97E-25	0.632789776	0.514	0.156	6.11E-21	12
MEF2C	3.30E-25	0.348810197	0.645	0.194	6.80E-21	12
BTG2	1.22E-24	0.724566596	0.879	0.419	2.51E-20	12
IGLC2	5.19E-24	6.468896949	0.486	0.162	1.07E-19	12
TXNDC11	6.08E-24	0.421567688	0.617	0.225	1.25E-19	12
UBE2J1	2.06E-23	0.467071403	0.673	0.283	4.23E-19	12
ERLEC1	2.93E-23	0.434117992	0.664	0.28	6.03E-19	12
PDK1	4.87E-22	0.256233535	0.467	0.143	1.00E-17	12
SEL1L	1.38E-21	0.411119738	0.607	0.231	2.85E-17	12
RABAC1	7.96E-20	0.489940588	0.841	0.473	1.64E-15	12
CLPTM1L	1.02E-19	0.34252305	0.701	0.323	2.11E-15	12
ERN1	4.08E-19	0.444859889	0.523	0.193	8.41E-15	12
TXNDC15	2.11E-18	0.286967835	0.514	0.199	4.35E-14	12

ANKRD28	2.58E-18	0.454095095	0.505	0.185	5.32E-14	12
KLF13	2.62E-18	0.263577797	0.589	0.235	5.39E-14	12
BLNK	9.17E-18	0.319171337	0.421	0.139	1.89E-13	12
MANF	4.22E-17	0.422149308	0.794	0.412	8.69E-13	12
MYDGF	1.37E-15	0.416771995	0.804	0.457	2.82E-11	12
RRBP1	1.52E-15	0.569942256	0.86	0.461	3.13E-11	12
SDF2L1	3.49E-15	0.466767312	0.813	0.464	7.19E-11	12
CRELD2	6.77E-15	0.269629378	0.607	0.276	1.39E-10	12
SPCS2	1.80E-14	0.403172456	0.841	0.614	3.70E-10	12
LMAN1	3.94E-14	0.430931055	0.822	0.465	8.12E-10	12
AC007952.4	4.26E-14	0.31264654	0.327	0.106	8.78E-10	12
RGS16	6.70E-14	0.365116928	0.262	0.067	1.38E-09	12
SDC1	5.31E-12	0.270070938	0.607	0.278	1.09E-07	12
FAM214A	8.29E-12	0.262718842	0.374	0.146	1.71E-07	12
SUB1	3.82E-11	0.556245521	0.907	0.709	7.87E-07	12
RPL22L1	1.41E-10	0.428790876	0.72	0.43	2.89E-06	12
AL627171.1	2.02E-10	0.269133809	0.299	0.106	4.15E-06	12
CYBA	3.69E-10	0.28743842	0.935	0.534	7.60E-06	12
CPEB4	3.90E-10	0.377871125	0.514	0.26	8.04E-06	12
HSP90B1	6.02E-10	0.458945964	0.944	0.68	1.24E-05	12
SLC17A9	2.13E-09	0.270508836	0.318	0.13	4.38E-05	12
JUN	9.80E-09	0.630039126	0.869	0.68	0.00020187	12
CYTOR	2.26E-08	0.266655911	0.682	0.396	0.000465867	12
EDEM1	1.25E-07	0.260277081	0.383	0.191	0.002579273	12
SRM	2.62E-07	0.267176865	0.636	0.388	0.005399606	12
SSR3	7.03E-07	0.267655436	0.813	0.583	0.014469193	12
SEC61B	2.60E-06	0.314369887	0.907	0.679	0.053510523	12
COPE	4.90E-06	0.267542308	0.804	0.583	0.100927519	12
SLC5A3	8.01E-06	0.300884396	0.43	0.248	0.165063676	12
PPIB	3.02E-05	0.307354465	0.907	0.699	0.621125124	12
HSPA6	9.17E-05	0.378397469	0.458	0.292	1	12
MS4A1	0	2.501947249	0.928	0.006	0	13
CD79A	0	2.472678558	0.964	0.03	0	13
BANK1	0	1.858773508	0.831	0.005	0	13
IGHD	0	1.308749065	0.349	0.001	0	13
TNFRSF13C	0	1.197392775	0.602	0.01	0	13
SPIB	0	1.159681844	0.614	0.013	0	13
LINC00926	0	1.158621357	0.53	0.009	0	13
FCRLA	0	1.088430602	0.542	0.003	0	13
PAX5	0	0.789946511	0.398	0.001	0	13
BLK	3.03E-306	0.782964289	0.313	0.001	6.24E-302	13
IGHM	2.56E-284	2.866696327	0.627	0.016	5.28E-280	13
VPREB3	3.99E-246	1.304205402	0.47	0.009	8.21E-242	13
FCRL1	9.64E-232	0.664625828	0.289	0.002	1.99E-227	13
LINC02397	1.11E-219	0.63889526	0.289	0.003	2.28E-215	13

CD22	2.55E-177	0.987559076	0.422	0.012	5.25E-173	13
IGHA1	1.30E-162	1.352488405	0.422	0.013	2.68E-158	13
CD19	5.35E-154	0.786124827	0.337	0.008	1.10E-149	13
FAM30A	1.24E-128	0.769558939	0.301	0.008	2.55E-124	13
HLA-DOB	3.54E-121	0.799557406	0.398	0.017	7.29E-117	13
POU2AF1	1.67E-113	0.781778914	0.386	0.017	3.43E-109	13
TNFRSF13B	1.01E-107	0.641840922	0.313	0.011	2.09E-103	13
CD79B	1.68E-102	1.560959774	0.759	0.09	3.46E-98	13
TLR10	3.42E-102	0.64782269	0.313	0.012	7.04E-98	13
FCRL5	3.41E-90	0.969105865	0.313	0.014	7.03E-86	13
GAPT	4.17E-79	0.555024547	0.301	0.015	8.58E-75	13
BCL11A	9.83E-75	0.969651861	0.434	0.037	2.02E-70	13
CCR7	8.47E-67	0.889357257	0.361	0.027	1.74E-62	13
CD37	4.61E-63	1.984582047	0.976	0.35	9.50E-59	13
POU2F2	1.78E-59	1.415528583	0.759	0.16	3.66E-55	13
FCMR	7.91E-59	1.122768195	0.602	0.092	1.63E-54	13
CPNE5	5.85E-50	0.577987148	0.277	0.022	1.20E-45	13
ADAM28	1.97E-49	0.860545444	0.41	0.051	4.06E-45	13
LTB	1.64E-48	1.185218116	0.843	0.211	3.37E-44	13
MIR29B2CHG	4.45E-46	1.009782742	0.47	0.072	9.17E-42	13
HLA-DQA1	7.69E-45	1.030519946	0.94	0.26	1.58E-40	13
CD52	7.35E-44	1.354983543	0.952	0.367	1.51E-39	13
HLA-DQB1	1.97E-42	0.996688983	0.916	0.273	4.05E-38	13
HLA-DQA2	1.37E-40	1.058756887	0.831	0.235	2.83E-36	13
CD24	1.40E-40	0.824684942	0.325	0.038	2.88E-36	13
SP140	9.71E-40	0.837607655	0.494	0.088	2.00E-35	13
RIPOR2	6.97E-39	0.952731498	0.542	0.104	1.44E-34	13
LINC01857	6.61E-38	0.65916742	0.277	0.03	1.36E-33	13
SELL	3.41E-36	1.020646352	0.458	0.084	7.02E-32	13
TMEM156	2.49E-35	0.600042264	0.337	0.046	5.13E-31	13
IGLC3	5.23E-35	0.957908401	0.289	0.034	1.08E-30	13
TMEM154	2.14E-31	0.598605095	0.325	0.047	4.42E-27	13
PPM1K	7.93E-31	0.802952553	0.542	0.132	1.63E-26	13
HLA-DRB1	1.03E-29	0.632147137	0.976	0.332	2.13E-25	13
NCR3	1.50E-29	0.487518525	0.277	0.035	3.08E-25	13
PARP15	3.61E-29	0.552406527	0.289	0.04	7.43E-25	13
FAU	8.98E-29	0.721339094	0.988	0.904	1.85E-24	13
CD74	1.38E-28	0.98472162	1	0.621	2.84E-24	13
RPL19	4.97E-28	0.717057229	1	0.946	1.02E-23	13
RPL23A	7.55E-27	0.732576441	1	0.92	1.56E-22	13
HLA-DRA	9.15E-27	0.764027849	1	0.518	1.88E-22	13
RPL13A	1.84E-26	0.661971059	0.988	0.961	3.79E-22	13
MEF2C	4.12E-26	0.877004345	0.602	0.197	8.49E-22	13
RPS8	5.47E-26	0.69522761	0.964	0.938	1.13E-21	13
IKZF3	1.10E-25	0.633007357	0.422	0.086	2.27E-21	13

RPL18A	1.68E-25	0.714542811	1	0.96	3.47E-21	13
RPS23	2.63E-25	0.600892998	1	0.974	5.41E-21	13
BASP1	4.04E-25	0.8529938	0.578	0.177	8.32E-21	13
IER5	4.96E-25	1.161920162	0.892	0.47	1.02E-20	13
HLA-DPB1	1.21E-24	0.650667304	0.94	0.393	2.50E-20	13
HLA-DPA1	1.51E-24	0.590095862	0.976	0.451	3.12E-20	13
JUND	1.52E-24	1.047573275	0.976	0.807	3.13E-20	13
LYL1	2.57E-24	0.766489942	0.422	0.104	5.29E-20	13
LIMD2	6.52E-24	0.871253302	0.771	0.335	1.34E-19	13
RALGPS2	8.95E-24	1.017559156	0.53	0.178	1.84E-19	13
RPS29	1.29E-23	0.652903778	1	0.925	2.65E-19	13
NCF1	1.49E-23	0.746195433	0.53	0.161	3.06E-19	13
RPS12	3.41E-23	0.634301652	0.988	0.969	7.01E-19	13
RPL41	2.49E-22	0.551350597	1	0.982	5.13E-18	13
CDKN2D	3.61E-22	0.725750131	0.422	0.105	7.44E-18	13
1-Mar	6.07E-22	0.893995607	0.506	0.163	1.25E-17	13
RPS28	6.18E-22	0.533156449	1	0.942	1.27E-17	13
RPS15	1.29E-21	0.540654744	1	0.95	2.65E-17	13
LAPTM5	2.06E-21	0.779904575	0.928	0.448	4.24E-17	13
CXCR4	2.38E-21	1.106044386	0.735	0.332	4.90E-17	13
IRF8	2.98E-21	0.700946251	0.506	0.158	6.14E-17	13
RPL34	3.54E-21	0.603025171	1	0.951	7.29E-17	13
SP110	5.01E-21	0.890058668	0.687	0.333	1.03E-16	13
ORAI2	8.97E-21	0.689294131	0.398	0.104	1.85E-16	13
ARHGAP24	1.32E-20	0.646436952	0.313	0.067	2.72E-16	13
CD83	3.29E-20	1.072534224	0.614	0.232	6.78E-16	13
HLA-DMA	5.88E-20	0.545731725	0.855	0.329	1.21E-15	13
SWAP70	7.84E-20	0.802498513	0.53	0.204	1.61E-15	13
LBH	2.17E-19	0.585050188	0.506	0.149	4.47E-15	13
RPL28	2.25E-19	0.503689572	1	0.973	4.63E-15	13
ID3	4.98E-19	0.906572137	0.47	0.141	1.02E-14	13
RPS19	5.44E-19	0.591570614	0.988	0.975	1.12E-14	13
BIRC3	6.28E-19	0.734032327	0.651	0.258	1.29E-14	13
RPLP2	7.20E-19	0.49530684	0.988	0.962	1.48E-14	13
PTPN6	1.73E-18	0.727046496	0.639	0.283	3.56E-14	13
RPS3A	2.56E-18	0.538927055	0.988	0.962	5.26E-14	13
RPL21	2.71E-18	0.587513975	1	0.953	5.58E-14	13
RPL13	2.83E-18	0.498914505	1	0.991	5.83E-14	13
RPS11	3.82E-18	0.518081688	0.988	0.936	7.86E-14	13
FCGR2B	4.47E-18	0.483488457	0.313	0.07	9.20E-14	13
RPL9	5.45E-18	0.542064694	0.988	0.951	1.12E-13	13
CD55	7.17E-18	0.859782258	0.482	0.171	1.48E-13	13
RPS5	1.10E-17	0.594499543	1	0.923	2.26E-13	13
SMAP2	1.14E-17	0.908517495	0.699	0.401	2.35E-13	13
RPS25	1.15E-17	0.524192131	1	0.941	2.38E-13	13

RPL30	1.17E-17	0.4764862	1	0.973	2.40E-13	13
AREG	1.23E-17	0.897748317	0.349	0.086	2.54E-13	13
CD69	1.41E-17	0.762418378	0.578	0.202	2.91E-13	13
RPS27	1.83E-17	0.469204932	1	0.989	3.76E-13	13
RGS16	2.35E-17	0.868596528	0.301	0.068	4.84E-13	13
MALAT1	3.04E-17	0.616234343	1	0.953	6.26E-13	13
RHOH	3.06E-17	0.554403868	0.434	0.122	6.31E-13	13
BCAS4	3.11E-17	0.606512088	0.289	0.066	6.40E-13	13
KLF2	3.53E-17	0.809021778	0.699	0.301	7.27E-13	13
STK17A	3.70E-17	0.823380723	0.578	0.245	7.63E-13	13
RPL11	5.71E-17	0.445035966	1	0.974	1.18E-12	13
PLAC8	1.18E-16	0.464234104	0.386	0.099	2.43E-12	13
TCF4	2.39E-16	0.785436725	0.578	0.265	4.93E-12	13
DNAJB1	3.68E-16	0.841899649	0.928	0.623	7.59E-12	13
RPL10	4.01E-16	0.44600004	1	0.987	8.26E-12	13
CD48	4.53E-16	0.705452353	0.735	0.357	9.33E-12	13
TSC22D3	8.19E-16	0.731174207	0.747	0.357	1.69E-11	13
FOSB	1.35E-15	0.752214763	0.807	0.432	2.78E-11	13
RPS26	4.04E-15	0.488534463	0.964	0.835	8.33E-11	13
DUSP1	4.73E-15	0.752965243	0.916	0.635	9.73E-11	13
DAPP1	4.74E-15	0.589596744	0.349	0.103	9.76E-11	13
RPS4Y1	5.76E-15	0.690615235	0.723	0.389	1.19E-10	13
RPS9	5.88E-15	0.502381417	0.976	0.939	1.21E-10	13
RPL18	6.99E-15	0.44970443	1	0.959	1.44E-10	13
GRASP	9.21E-15	0.510088452	0.289	0.072	1.90E-10	13
RPL12	9.51E-15	0.433064624	0.988	0.948	1.96E-10	13
RPS14	1.41E-14	0.411954399	0.988	0.973	2.90E-10	13
RPS3	1.91E-14	0.432813004	1	0.96	3.92E-10	13
LAT2	2.06E-14	0.692443499	0.373	0.124	4.23E-10	13
RPS15A	2.89E-14	0.391578083	0.988	0.965	5.95E-10	13
RPL26	2.92E-14	0.42050644	0.988	0.954	6.02E-10	13
RPL36	3.14E-14	0.429852474	0.976	0.92	6.46E-10	13
JUNB	4.13E-14	0.711138257	0.855	0.551	8.50E-10	13
RPS2	6.00E-14	0.4104815	1	0.975	1.24E-09	13
SMIM14	7.97E-14	0.995607207	0.554	0.306	1.64E-09	13
PPP1R15A	1.09E-13	0.635990311	0.795	0.459	2.24E-09	13
HSP90AA1	1.09E-13	0.588363725	0.988	0.891	2.25E-09	13
RPL39	1.17E-13	0.331102237	0.976	0.985	2.41E-09	13
IER2	1.44E-13	0.737271757	0.819	0.498	2.97E-09	13
ISG20	1.84E-13	0.658428085	0.494	0.189	3.79E-09	13
RPL8	1.94E-13	0.404325149	0.988	0.957	3.99E-09	13
REL	2.03E-13	0.867422927	0.687	0.423	4.18E-09	13
ACAP1	2.34E-13	0.506271161	0.494	0.179	4.83E-09	13
TNFAIP8	2.79E-13	0.60457703	0.554	0.253	5.75E-09	13
TNF	4.76E-13	0.673964467	0.398	0.137	9.80E-09	13

NFKBID	5.76E-13	0.744726543	0.41	0.156	1.19E-08	13
MARCKSL1	5.77E-13	0.74138028	0.627	0.35	1.19E-08	13
RPS20	1.40E-12	0.482682653	0.964	0.89	2.87E-08	13
SRRM2	1.68E-12	0.515769807	0.855	0.656	3.46E-08	13
RPLP1	1.72E-12	0.395407344	1	0.986	3.54E-08	13
HVCN1	1.76E-12	0.630790065	0.325	0.108	3.62E-08	13
BTG1	2.06E-12	0.519039498	0.916	0.76	4.25E-08	13
CD53	2.11E-12	0.528580036	0.771	0.402	4.34E-08	13
PNISR	2.28E-12	0.686959858	0.747	0.57	4.69E-08	13
EZR	2.87E-12	0.659328058	0.554	0.275	5.91E-08	13
RASGRP2	3.64E-12	0.549586318	0.277	0.078	7.51E-08	13
RPS16	4.19E-12	0.338748727	1	0.957	8.62E-08	13
RPS4X	5.49E-12	0.337560655	0.976	0.951	1.13E-07	13
EVI2B	6.37E-12	0.497822722	0.53	0.238	1.31E-07	13
LY86	6.51E-12	0.52126104	0.41	0.16	1.34E-07	13
TRAF5	1.12E-11	0.534529086	0.301	0.094	2.30E-07	13
GGA2	1.16E-11	0.585702364	0.458	0.213	2.38E-07	13
RCSD1	1.58E-11	0.580055908	0.422	0.176	3.25E-07	13
HSPA1B	1.78E-11	0.469055474	0.94	0.63	3.66E-07	13
CORO1A	1.87E-11	0.533662971	0.723	0.377	3.85E-07	13
RPL7A	4.71E-11	0.352449264	0.976	0.954	9.70E-07	13
HLA-DMB	5.52E-11	0.425106526	0.566	0.232	1.14E-06	13
JUN	6.98E-11	0.558463788	0.916	0.68	1.44E-06	13
CAMK1D	8.90E-11	0.461134211	0.289	0.094	1.83E-06	13
YPEL5	1.11E-10	0.825153969	0.639	0.434	2.29E-06	13
GPSM3	1.24E-10	0.498087564	0.627	0.331	2.55E-06	13
RPL23	1.24E-10	0.337528544	1	0.877	2.56E-06	13
RPL10A	1.25E-10	0.36190847	0.976	0.939	2.58E-06	13
SLC38A1	1.79E-10	0.481714097	0.337	0.114	3.69E-06	13
RAC2	2.05E-10	0.577859013	0.663	0.397	4.22E-06	13
PRDM2	2.92E-10	0.606396647	0.41	0.183	6.02E-06	13
RPS27A	4.32E-10	0.342813797	1	0.983	8.90E-06	13
PLEKHO1	6.22E-10	0.442672605	0.518	0.247	1.28E-05	13
RPS13	6.66E-10	0.343137997	0.964	0.953	1.37E-05	13
TRIM38	7.40E-10	0.718357322	0.494	0.276	1.52E-05	13
AFF3	8.24E-10	0.570816845	0.289	0.105	1.70E-05	13
HLA-E	1.10E-09	0.398597287	0.94	0.739	2.27E-05	13
NOP53	1.16E-09	0.434496138	0.807	0.621	2.39E-05	13
BTK	1.36E-09	0.413888568	0.289	0.105	2.80E-05	13
RPL27A	1.66E-09	0.292194723	0.988	0.938	3.42E-05	13
NR4A2	1.67E-09	0.85358536	0.542	0.292	3.45E-05	13
RPSA	1.81E-09	0.32682346	0.988	0.923	3.72E-05	13
TAGLN2	1.90E-09	0.480096272	0.723	0.469	3.92E-05	13
TXNIP	2.10E-09	0.677302249	0.807	0.641	4.33E-05	13
RPL3	2.81E-09	0.314885897	0.976	0.965	5.79E-05	13

ARID1B	2.93E-09	0.59282471	0.602	0.415	6.04E-05	13
DNAJA1	3.86E-09	0.493633306	0.843	0.702	7.96E-05	13
UBA52	3.98E-09	0.339430566	0.976	0.918	8.20E-05	13
FOS	4.85E-09	0.753534201	0.747	0.485	9.98E-05	13
IFT57	4.92E-09	0.559515203	0.289	0.11	0.000101422	13
ZFAS1	5.78E-09	0.40710385	0.771	0.603	0.000118941	13
PTMA	5.80E-09	0.285470334	1	0.954	0.000119444	13
TBC1D10C	6.29E-09	0.41495326	0.361	0.139	0.000129536	13
HLA-B	6.65E-09	0.439077615	0.976	0.662	0.000136981	13
EIF1	8.78E-09	0.306417281	1	0.914	0.000180904	13
NUDT1	9.04E-09	0.499515044	0.41	0.198	0.000186171	13
SP100	9.46E-09	0.603855825	0.554	0.362	0.000194853	13
CIITA	9.94E-09	0.480955234	0.301	0.119	0.000204613	13
YWHAZ	1.15E-08	0.479541791	0.711	0.577	0.000237595	13
DDX5	1.17E-08	0.453627532	0.892	0.784	0.000240323	13
NR4A1	1.29E-08	0.537780437	0.578	0.31	0.000265165	13
ANKRD44	1.82E-08	0.586011773	0.482	0.271	0.000374308	13
MYO1G	3.58E-08	0.296087102	0.373	0.155	0.000737807	13
SEL1L3	4.01E-08	0.396530005	0.253	0.09	0.000825906	13
RPS6	5.59E-08	0.258579065	0.988	0.974	0.001151418	13
CD40	5.92E-08	0.696246933	0.41	0.226	0.00121902	13
RPL35	6.57E-08	0.261228664	0.976	0.934	0.001352712	13
FUS	8.37E-08	0.382358161	0.783	0.651	0.001723831	13
STK17B	9.77E-08	0.45822026	0.458	0.239	0.002012415	13
RBM38	1.03E-07	0.468930817	0.325	0.142	0.002119083	13
LSM7	1.03E-07	0.553041593	0.614	0.479	0.002129629	13
FAM107B	1.28E-07	0.583852334	0.542	0.367	0.002628834	13
UBC	2.30E-07	0.473662845	0.94	0.884	0.004741879	13
SRSF7	2.44E-07	0.438881686	0.783	0.656	0.005025766	13
EEF1B2	2.52E-07	0.310507299	0.94	0.892	0.005184465	13
PRKCB	2.58E-07	0.541508978	0.361	0.173	0.005319866	13
CACYBP	2.72E-07	0.510839942	0.735	0.594	0.005607654	13
ARHGDIB	2.86E-07	0.291183103	0.904	0.508	0.005888914	13
KDM6B	2.93E-07	0.686409842	0.434	0.24	0.006028272	13
SYNGR2	3.36E-07	0.56182781	0.578	0.447	0.006915194	13
CYBA	3.55E-07	0.261993018	0.892	0.536	0.007302938	13
PPP1R2	4.14E-07	0.408407226	0.554	0.369	0.008526805	13
CYTIP	4.33E-07	0.358651643	0.482	0.248	0.008909132	13
H3F3B	4.41E-07	0.395523873	0.964	0.881	0.009084198	13
PFDN5	4.81E-07	0.378218876	0.916	0.828	0.009905276	13
RPL38	5.09E-07	0.292874988	0.928	0.892	0.010487842	13
BCAS2	5.53E-07	0.834869802	0.518	0.371	0.011389313	13
RACK1	5.73E-07	0.291534318	0.952	0.9	0.011806716	13
TMEM243	6.61E-07	0.416946235	0.349	0.176	0.013616425	13
HSPH1	6.94E-07	0.453607739	0.699	0.506	0.014297598	13

ILF3-DT	7.34E-07	0.648676785	0.422	0.246	0.015111693	13
CELF2	7.84E-07	0.390025605	0.506	0.293	0.016140264	13
SNX2	7.85E-07	0.510581579	0.506	0.335	0.016161756	13
SEPT6	8.70E-07	0.644774148	0.518	0.376	0.017926331	13
CDC42EP3	9.83E-07	0.320486303	0.313	0.135	0.020244985	13
IFI16	9.90E-07	0.429544189	0.554	0.342	0.020385473	13
PCSK7	1.10E-06	0.518676847	0.494	0.31	0.022642138	13
EIF1AY	1.17E-06	0.434898078	0.325	0.154	0.024110952	13
TOMM7	1.42E-06	0.364954031	0.843	0.747	0.029299455	13
CD72	1.47E-06	0.435056469	0.265	0.113	0.030181835	13
PLEKHF2	1.62E-06	0.451038584	0.301	0.145	0.033297907	13
RNASET2	1.80E-06	0.370148951	0.627	0.382	0.037113034	13
BCL2	1.89E-06	0.389630114	0.301	0.132	0.038885486	13
PTK2B	2.07E-06	0.475789383	0.337	0.172	0.042548211	13
CYB561A3	2.17E-06	0.576040506	0.301	0.147	0.044743658	13
BTG2	2.23E-06	0.489375796	0.602	0.425	0.04599346	13
HSPA8	2.35E-06	0.26164931	0.928	0.744	0.048459112	13
IRF7	2.62E-06	0.321986345	0.265	0.113	0.053992296	13
HNRNPDL	2.79E-06	0.44226128	0.723	0.642	0.057421221	13
BLNK	2.81E-06	0.471908674	0.289	0.142	0.057930982	13
HERPUD1	2.97E-06	0.628610617	0.711	0.596	0.061095928	13
GNB5	3.51E-06	0.523840155	0.277	0.131	0.072372765	13
ARHGAP25	3.58E-06	0.329903118	0.265	0.115	0.073773409	13
OTUD1	3.80E-06	0.636674049	0.325	0.168	0.078301917	13
ARPC3	3.97E-06	0.292934798	0.904	0.755	0.081800944	13
IKZF1	4.39E-06	0.419375851	0.446	0.25	0.09042958	13
EIF3F	4.54E-06	0.295934537	0.783	0.643	0.093437383	13
SS18L2	4.73E-06	0.520849459	0.494	0.366	0.09747226	13
EEF1D	5.24E-06	0.309250908	0.916	0.826	0.107892511	13
TRIR	5.33E-06	0.394451237	0.675	0.621	0.109866652	13
SNHG8	5.43E-06	0.518081694	0.578	0.462	0.11187317	13
CYCS	5.99E-06	0.475637433	0.711	0.623	0.123366472	13
EIF5	6.04E-06	0.456513297	0.687	0.613	0.124366309	13
SERTAD1	6.07E-06	0.413521786	0.434	0.251	0.125008531	13
RSRP1	6.13E-06	0.397789125	0.627	0.487	0.126236933	13
ANAPC16	6.18E-06	0.475284011	0.627	0.536	0.1273339	13
MOB3A	6.20E-06	0.397760618	0.325	0.165	0.127668455	13
CCNI	6.27E-06	0.431508356	0.723	0.62	0.129160709	13
ANKRD13A	6.44E-06	0.536656767	0.289	0.145	0.132560622	13
DDX3Y	6.81E-06	0.372235534	0.422	0.234	0.140271118	13
IRF1	7.30E-06	0.502335958	0.639	0.435	0.150253213	13
COX4I1	7.36E-06	0.281955899	0.892	0.778	0.151481018	13
CLEC2D	7.53E-06	0.322318344	0.41	0.217	0.154986601	13
GPBP1	7.88E-06	0.495430278	0.542	0.41	0.162274244	13
PMAIP1	9.27E-06	0.478857707	0.482	0.287	0.190962534	13

PKIG	1.03E-05	0.517914141	0.349	0.205	0.212317465	13
EEF2	1.07E-05	0.316265591	0.855	0.751	0.221376764	13
TAF7	1.12E-05	0.392441844	0.518	0.37	0.231447806	13
CIRBP	1.26E-05	0.462554101	0.663	0.574	0.259888996	13
TSPYL2	1.28E-05	0.37473331	0.386	0.203	0.262750907	13
9-Mar	1.47E-05	0.408062468	0.253	0.117	0.302206692	13
EMB	1.98E-05	0.272172053	0.277	0.129	0.407502673	13
UXT	2.08E-05	0.327307603	0.59	0.485	0.42760192	13
INPP5D	2.17E-05	0.390966873	0.325	0.172	0.446368003	13
TRIM22	2.87E-05	0.449210725	0.325	0.174	0.591591011	13
TUT4	3.22E-05	0.425213819	0.398	0.262	0.663706375	13
DUSP2	3.31E-05	0.47266974	0.446	0.257	0.682328378	13
UCP2	3.59E-05	0.351876849	0.554	0.345	0.739469649	13
AKNA	3.90E-05	0.270485079	0.337	0.178	0.802271978	13
MYCBP2	4.44E-05	0.474718953	0.482	0.363	0.914320631	13
CYLD	4.70E-05	0.390805128	0.434	0.29	0.968938935	13
CD44	5.01E-05	0.323966395	0.494	0.317	1	13
NIPA2	5.34E-05	0.417523811	0.325	0.193	1	13
MRPL18	5.70E-05	0.658682686	0.566	0.493	1	13
RBM39	6.08E-05	0.347440037	0.735	0.671	1	13
SNHG7	6.14E-05	0.536587153	0.422	0.295	1	13
TRAF3IP3	6.35E-05	0.344941705	0.313	0.16	1	13
TAGAP	7.02E-05	0.448783393	0.349	0.19	1	13
TUBA1A	7.16E-05	0.381608969	0.482	0.301	1	13
SLC25A6	7.35E-05	0.363338651	0.687	0.63	1	13
UBE2S	8.28E-05	0.499890605	0.506	0.365	1	13
HNRNPA2B1	8.49E-05	0.273183074	0.928	0.814	1	13
RPL22L1	9.97E-05	0.519059725	0.518	0.434	1	13
EMP3	0.000122601	0.274683607	0.602	0.41	1	13
CTSH	0.000122731	0.481982642	0.506	0.38	1	13
YME1L1	0.000136484	0.584131908	0.566	0.478	1	13
KDM4B	0.00015974	0.458782038	0.253	0.135	1	13
HSPE1	0.000167453	0.313200054	0.904	0.835	1	13
AL121944.1	0.000181142	0.605934461	0.253	0.137	1	13
RBM25	0.00018218	0.40685995	0.602	0.52	1	13
SRSF5	0.000188872	0.480752754	0.614	0.568	1	13
HNRNPA1	0.000190216	0.26411952	0.867	0.797	1	13
TRA2B	0.000194274	0.386831328	0.614	0.547	1	13
VAMP2	0.000207168	0.355124585	0.554	0.45	1	13
CYFIP2	0.000218602	0.311386369	0.265	0.138	1	13
PSMB9	0.000224254	0.342619463	0.59	0.472	1	13
CHORDC1	0.000246691	0.518956051	0.458	0.344	1	13
TAF1D	0.000249112	0.317102195	0.578	0.497	1	13
DDX6	0.000250431	0.369040224	0.434	0.323	1	13
FXYD5	0.000255669	0.2646683	0.542	0.36	1	13

DNAJB4	0.000275772	0.414046203	0.41	0.275	1	13
ZEB2	0.00027656	0.269297576	0.422	0.253	1	13
HSPA1A	0.000280914	0.256451684	0.904	0.866	1	13
ATF7IP	0.000290618	0.402197341	0.337	0.215	1	13
SMCHD1	0.000312107	0.455889823	0.434	0.333	1	13
DDX21	0.000353324	0.47096705	0.506	0.444	1	13
SEPT7	0.000353531	0.386276355	0.614	0.567	1	13
UPF2	0.000354812	0.393412118	0.41	0.296	1	13
IL16	0.000367647	0.433096115	0.265	0.148	1	13
COQ7	0.000377221	0.312372146	0.253	0.137	1	13
TPD52	0.00049847	0.465407866	0.277	0.168	1	13
DEDD2	0.000512243	0.58088314	0.349	0.235	1	13
RGS2	0.000531253	0.318580268	0.53	0.36	1	13
SYPL1	0.000584011	0.481306122	0.458	0.382	1	13
PRMT9	0.000601414	0.319932755	0.265	0.146	1	13
CD82	0.000610493	0.467847956	0.386	0.273	1	13
FNBP1	0.000611318	0.37449671	0.47	0.364	1	13
DOCK8	0.000693296	0.344273081	0.325	0.199	1	13
NSD3	0.00069718	0.435531066	0.458	0.381	1	13
TUBA4A	0.000742016	0.398412137	0.458	0.348	1	13
APOBEC3C	0.000856517	0.307781651	0.301	0.182	1	13
SNHG15	0.000880273	0.420733801	0.349	0.234	1	13
CNN2	0.000894729	0.397484813	0.422	0.319	1	13
LSP1	0.001403627	0.269270117	0.518	0.368	1	13
CLK1	0.001406591	0.350864937	0.518	0.426	1	13
ARHGAP15	0.001460636	0.268699953	0.253	0.142	1	13
SNHG12	0.001729659	0.339848488	0.253	0.147	1	13
H2AFX	0.001743728	0.399235247	0.349	0.242	1	13
SET	0.0017713	0.279904696	0.663	0.665	1	13
DRAM2	0.001894449	0.366623001	0.41	0.322	1	13
UBE2D2	0.002083083	0.262606657	0.554	0.512	1	13
CIB1	0.002130895	0.391471108	0.614	0.588	1	13
ICAM3	0.002134325	0.314782657	0.386	0.275	1	13
PSME1	0.002201624	0.272797663	0.699	0.648	1	13
PLP2	0.002296587	0.309136401	0.301	0.188	1	13
CNPY3	0.002643353	0.371809449	0.434	0.365	1	13
ZNF331	0.002957423	0.793213721	0.458	0.398	1	13
SEPT9	0.003135743	0.390700352	0.482	0.403	1	13
RGS19	0.003139265	0.365513763	0.313	0.217	1	13
KMT2E	0.00321992	0.363856011	0.542	0.513	1	13
CNTRL	0.003289493	0.389172488	0.265	0.17	1	13
NCOA3	0.00347825	0.432588527	0.373	0.306	1	13
FNIP1	0.003578871	0.390707413	0.386	0.307	1	13
CCNL1	0.003703725	0.372784556	0.566	0.529	1	13
CDC42SE2	0.004287803	0.270691286	0.41	0.315	1	13

ELF1	0.004486515	0.296662492	0.47	0.391	1	13
MIS18BP1	0.004513523	0.4284337	0.313	0.229	1	13
SH3BP5	0.004558453	0.359968031	0.386	0.314	1	13
LSM8	0.004597814	0.340517617	0.506	0.476	1	13
CAPG	0.004691509	0.30807099	0.361	0.265	1	13
ARL4A	0.004987099	0.447566468	0.313	0.222	1	13
FMNL1	0.005227319	0.324106096	0.361	0.26	1	13
HSP90AB1	0.006123277	0.305428681	0.94	0.882	1	13
NCF4	0.006180089	0.263904106	0.265	0.171	1	13
PARP14	0.006224704	0.323449335	0.41	0.337	1	13
CHD9	0.006227173	0.405101281	0.386	0.325	1	13
CHD7	0.006345573	0.402270915	0.265	0.187	1	13
IFRD1	0.006375069	0.327374997	0.434	0.347	1	13
CASP4	0.006682759	0.257252077	0.422	0.343	1	13
PTPN1	0.006956328	0.369248418	0.349	0.281	1	13
PPP1R12A	0.007017109	0.318726846	0.47	0.422	1	13
TLE4	0.008171126	0.267219558	0.301	0.204	1	13
NFKB2	0.008846912	0.269549226	0.313	0.225	1	13
TCOF1	0.009856971	0.362116612	0.277	0.2	1	13
CEP55	2.34E-230	0.588549439	0.71	0.023	4.81E-226	14
SHCBP1	1.14E-217	0.516394704	0.71	0.026	2.35E-213	14
TK1	7.15E-217	0.992844451	0.899	0.045	1.47E-212	14
GTSE1	1.28E-212	0.889977728	0.739	0.029	2.64E-208	14
PIMREG	9.26E-206	0.281617294	0.377	0.006	1.91E-201	14
AURKB	3.79E-198	0.616060771	0.739	0.031	7.80E-194	14
NCAPH	2.94E-190	0.46426556	0.696	0.029	6.05E-186	14
MKI67	1.77E-189	1.513254854	0.913	0.055	3.65E-185	14
CDK1	2.46E-182	1.210752351	0.87	0.051	5.07E-178	14
KIF11	2.24E-178	0.527133263	0.652	0.027	4.61E-174	14
PCLAF	2.15E-170	1.110621768	0.942	0.065	4.42E-166	14
PRC1	2.40E-170	0.609010728	0.768	0.041	4.95E-166	14
ASF1B	1.02E-169	0.598892306	0.768	0.041	2.10E-165	14
ANLN	1.02E-169	0.504739833	0.638	0.027	2.10E-165	14
NCAPG	7.51E-161	0.496083566	0.667	0.032	1.55E-156	14
BUB1B	1.07E-159	0.360034884	0.551	0.021	2.21E-155	14
KIF23	1.41E-158	0.477170475	0.594	0.025	2.90E-154	14
HMMR	5.57E-158	0.96661573	0.652	0.032	1.15E-153	14
NUF2	1.38E-152	0.561411405	0.71	0.039	2.84E-148	14
CCNA2	1.51E-150	0.559560886	0.638	0.031	3.11E-146	14
FOXM1	9.31E-150	0.415677477	0.609	0.029	1.92E-145	14
PKMYT1	2.55E-146	0.368737603	0.493	0.018	5.25E-142	14
CENPU	1.95E-145	0.556755027	0.768	0.049	4.01E-141	14
CLSPN	7.27E-144	0.595513814	0.667	0.036	1.50E-139	14
CBR3	2.80E-138	0.399237779	0.536	0.023	5.77E-134	14
ASPM	1.42E-136	0.884014154	0.754	0.05	2.93E-132	14

UHRF1	2.94E-135	0.451313632	0.551	0.026	6.05E-131	14
KIF18B	9.13E-133	0.292721199	0.42	0.014	1.88E-128	14
CCNB2	2.79E-131	0.79044927	0.71	0.046	5.75E-127	14
CDCA5	1.58E-130	0.349514339	0.536	0.025	3.25E-126	14
BUB1	4.23E-130	0.331224803	0.507	0.022	8.71E-126	14
CENPF	1.14E-129	1.299875805	0.812	0.064	2.34E-125	14
RAD51AP1	7.58E-128	0.686276812	0.783	0.061	1.56E-123	14
ZWINT	2.40E-126	0.604843726	0.783	0.059	4.95E-122	14
DLGAP5	8.15E-124	0.423588373	0.522	0.025	1.68E-119	14
KIF2C	2.17E-123	0.371955013	0.464	0.02	4.46E-119	14
SGO1	2.31E-121	0.423504839	0.667	0.043	4.76E-117	14
KIF14	6.47E-120	0.397487096	0.464	0.02	1.33E-115	14
PLK1	8.07E-120	0.667669441	0.551	0.03	1.66E-115	14
SPAG5	5.34E-118	0.403836041	0.551	0.03	1.10E-113	14
MYBL2	1.58E-117	0.496426092	0.609	0.037	3.25E-113	14
TOP2A	5.58E-117	1.526919977	0.913	0.092	1.15E-112	14
KIF20A	3.95E-116	0.331277792	0.435	0.018	8.14E-112	14
CIT	5.40E-114	0.267979656	0.435	0.019	1.11E-109	14
CDCA3	1.02E-113	0.47669369	0.478	0.023	2.11E-109	14
DEPDC1	1.81E-113	0.446303381	0.507	0.026	3.73E-109	14
RRM2	5.84E-113	0.934412275	0.783	0.066	1.20E-108	14
NUSAP1	8.21E-112	1.009921684	0.841	0.081	1.69E-107	14
CENPA	2.49E-111	0.401246037	0.507	0.027	5.14E-107	14
CENPE	5.32E-111	0.704447884	0.623	0.042	1.10E-106	14
CDCA8	7.35E-111	0.331697445	0.507	0.027	1.51E-106	14
TYMS	7.84E-111	1.220953329	0.957	0.11	1.61E-106	14
BRCA1	8.94E-110	0.468867508	0.638	0.045	1.84E-105	14
KIFC1	7.27E-108	0.534499421	0.681	0.052	1.50E-103	14
MAD2L1	9.65E-108	0.551163935	0.783	0.069	1.99E-103	14
SKA3	1.14E-107	0.257609739	0.464	0.023	2.36E-103	14
CENPM	5.92E-107	0.665110746	0.739	0.063	1.22E-102	14
KIF4A	6.90E-107	0.336793555	0.507	0.028	1.42E-102	14
BIRC5	7.36E-106	0.819862753	0.783	0.071	1.52E-101	14
CIP2A	9.16E-106	0.342922433	0.594	0.04	1.89E-101	14
TCF19	1.38E-104	0.356570539	0.623	0.044	2.85E-100	14
NDC80	1.84E-104	0.466430514	0.58	0.038	3.78E-100	14
UBE2C	4.60E-104	1.454303722	0.783	0.074	9.47E-100	14
SPC25	5.55E-104	0.321035931	0.536	0.032	1.14E-99	14
CDC20	1.98E-103	0.907015255	0.58	0.039	4.07E-99	14
CENPK	3.08E-103	0.468812591	0.725	0.06	6.35E-99	14
TPX2	9.59E-103	0.74344618	0.739	0.065	1.97E-98	14
CKAP2L	1.72E-102	0.432156539	0.565	0.037	3.54E-98	14
TTK	1.90E-101	0.336376713	0.464	0.025	3.92E-97	14
RACGAP1	5.44E-101	0.445826327	0.652	0.052	1.12E-96	14
MELK	7.75E-101	0.337109886	0.522	0.032	1.60E-96	14

TACC3	5.55E-100	0.630879439	0.826	0.083	1.14E-95	14
HMGB3	3.81E-98	0.685254345	0.754	0.074	7.84E-94	14
PRR11	3.18E-97	0.396043423	0.58	0.042	6.55E-93	14
HJURP	1.44E-95	0.321091656	0.464	0.026	2.98E-91	14
RPL39L	2.55E-93	0.259098542	0.507	0.031	5.24E-89	14
NCAPD2	4.09E-92	0.53783422	0.71	0.068	8.42E-88	14
FBXO5	1.12E-91	0.488757607	0.594	0.047	2.31E-87	14
SGO2	7.49E-89	0.584771387	0.696	0.069	1.54E-84	14
ARHGAP11A	7.43E-88	0.425622508	0.594	0.049	1.53E-83	14
TEDC1	5.01E-87	0.263715107	0.507	0.034	1.03E-82	14
FAM111B	5.58E-84	0.39995329	0.435	0.026	1.15E-79	14
NRM	1.36E-83	0.34881944	0.681	0.067	2.80E-79	14
CENPW	9.80E-83	0.600783521	0.768	0.091	2.02E-78	14
KIF15	1.41E-81	0.283765966	0.435	0.027	2.91E-77	14
KNL1	1.23E-80	0.41941322	0.623	0.059	2.53E-76	14
NRGN	4.33E-80	0.337711953	0.522	0.039	8.92E-76	14
CDKN3	1.55E-79	0.912258269	0.754	0.092	3.19E-75	14
TROAP	1.37E-78	0.350442132	0.435	0.029	2.82E-74	14
TUBB6	2.19E-78	0.487557675	0.681	0.071	4.51E-74	14
NEK2	6.44E-77	0.290206714	0.406	0.025	1.33E-72	14
FANCI	1.16E-75	0.315937219	0.551	0.048	2.39E-71	14
POLA2	1.73E-75	0.251782061	0.522	0.042	3.55E-71	14
C21orf58	2.93E-75	0.310748534	0.536	0.046	6.04E-71	14
CEP152	6.25E-72	0.295667083	0.609	0.061	1.29E-67	14
CCNB1	9.60E-71	0.956759764	0.594	0.062	1.98E-66	14
COQ2	1.10E-70	0.528482037	0.783	0.111	2.26E-66	14
FCGR1A	9.99E-70	0.628271428	0.783	0.102	2.06E-65	14
LMNB2	4.36E-69	0.321794207	0.638	0.071	8.97E-65	14
LMNB1	4.60E-69	0.624959822	0.87	0.14	9.47E-65	14
FEN1	3.31E-68	0.519446715	0.623	0.071	6.82E-64	14
C4orf46	8.27E-68	0.312761686	0.464	0.037	1.70E-63	14
CDCA7L	1.01E-67	0.321554437	0.493	0.043	2.08E-63	14
ZWILCH	2.60E-67	0.253804491	0.551	0.054	5.35E-63	14
RPP25	2.80E-66	0.333699794	0.565	0.056	5.76E-62	14
HPSE	5.96E-66	0.437497918	0.652	0.076	1.23E-61	14
PMP22	1.66E-65	0.402623956	0.739	0.094	3.41E-61	14
CD163	5.31E-65	0.928563414	0.899	0.139	1.09E-60	14
VSIG4	6.39E-65	0.849869828	0.783	0.112	1.32E-60	14
RNASE6	6.71E-65	1.018135307	0.971	0.179	1.38E-60	14
ORC6	4.73E-64	0.401185366	0.536	0.054	9.74E-60	14
SASH1	2.15E-63	0.412446486	0.768	0.103	4.43E-59	14
MS4A4A	3.79E-63	1.313103696	0.899	0.164	7.80E-59	14
PLAU	7.95E-63	0.708656575	0.696	0.09	1.64E-58	14
VRK1	1.88E-62	0.334192086	0.638	0.078	3.87E-58	14
GPR34	2.10E-62	0.50447621	0.754	0.102	4.33E-58	14

CDC25B	2.63E-62	0.437252058	0.754	0.108	5.41E-58	14
MRC1	7.86E-62	0.92899922	0.928	0.157	1.62E-57	14
STAB1	2.33E-61	1.141551652	0.913	0.17	4.80E-57	14
BRCA2	6.03E-61	0.320864249	0.58	0.066	1.24E-56	14
OTULINL	1.52E-60	0.540891876	0.783	0.118	3.13E-56	14
SMC2	6.32E-59	0.668876734	0.841	0.16	1.30E-54	14
CYBB	7.52E-59	0.980157407	0.986	0.193	1.55E-54	14
GPSM2	2.68E-58	0.383542257	0.536	0.061	5.52E-54	14
KCTD12	2.86E-58	0.844414083	0.986	0.189	5.89E-54	14
PHF19	4.60E-58	0.515222448	0.797	0.131	9.48E-54	14
KIF20B	7.81E-58	0.600446227	0.812	0.14	1.61E-53	14
FAM111A	1.76E-57	0.761407443	0.87	0.167	3.63E-53	14
HIRIP3	3.85E-57	0.377145343	0.652	0.091	7.93E-53	14
AURKA	5.36E-57	0.455290906	0.536	0.062	1.10E-52	14
C1QC	6.17E-57	1.53569563	0.957	0.203	1.27E-52	14
IGSF6	7.37E-57	1.104498492	1	0.207	1.52E-52	14
MS4A6A	1.31E-56	1.381139914	1	0.236	2.70E-52	14
DAB2	1.42E-56	1.054983774	0.957	0.183	2.93E-52	14
NCAPG2	1.54E-56	0.252045433	0.464	0.046	3.17E-52	14
DHFR	3.04E-56	0.600696562	0.696	0.11	6.27E-52	14
LRRC25	8.72E-56	0.606930984	0.812	0.138	1.80E-51	14
P2RY6	2.33E-55	0.404207807	0.609	0.077	4.80E-51	14
SLAMF8	6.14E-55	0.521469439	0.768	0.121	1.26E-50	14
CENPL	1.37E-54	0.339034703	0.536	0.064	2.82E-50	14
LAIR1	1.43E-54	0.705260416	0.913	0.17	2.94E-50	14
NCAPD3	1.52E-54	0.377257214	0.609	0.085	3.13E-50	14
CSF1R	4.84E-54	0.674352247	0.928	0.169	9.96E-50	14
CLEC7A	5.07E-54	0.573995024	0.841	0.146	1.04E-49	14
CCR1	9.84E-54	0.595494127	0.826	0.138	2.03E-49	14
CENPH	1.10E-53	0.284782793	0.507	0.057	2.26E-49	14
MNDA	1.34E-53	0.665313117	0.841	0.151	2.75E-49	14
TBXAS1	5.97E-53	0.481161297	0.841	0.144	1.23E-48	14
TLR2	7.42E-53	0.290539328	0.638	0.084	1.53E-48	14
IL18	7.57E-53	0.408689909	0.754	0.116	1.56E-48	14
MPEG1	9.80E-53	1.0158536	0.942	0.195	2.02E-48	14
CD209	1.29E-52	0.563019683	0.667	0.095	2.65E-48	14
FPR3	4.17E-52	0.532055982	0.826	0.14	8.58E-48	14
CLEC4A	5.05E-52	0.523676645	0.681	0.105	1.04E-47	14
NCF4	7.80E-52	0.596896098	0.87	0.164	1.61E-47	14
ENPP2	1.02E-51	0.299436543	0.58	0.073	2.10E-47	14
SIGLEC7	1.13E-51	0.365714778	0.71	0.106	2.34E-47	14
SYK	1.20E-51	0.547383152	0.783	0.136	2.47E-47	14
NUDT1	1.30E-51	0.618120948	0.899	0.192	2.68E-47	14
FOLR2	1.65E-51	0.911683872	0.797	0.136	3.40E-47	14
SDC3	3.12E-51	0.67850858	0.768	0.131	6.42E-47	14

UBE2T	4.09E-51	0.304532488	0.623	0.089	8.42E-47	14
TGFBI	6.99E-51	1.120477814	1	0.281	1.44E-46	14
FCGR1B	1.00E-50	0.281560633	0.493	0.055	2.06E-46	14
S100B	1.17E-50	0.428704396	0.42	0.041	2.42E-46	14
STMN1	1.38E-50	1.79715408	1	0.395	2.84E-46	14
ST14	1.70E-50	0.270958724	0.449	0.046	3.51E-46	14
PARVB	2.43E-50	0.453178189	0.797	0.139	5.01E-46	14
C1orf54	2.82E-50	0.53758239	0.797	0.138	5.80E-46	14
DCK	4.14E-50	0.430630014	0.754	0.13	8.52E-46	14
STAC3	5.58E-50	0.253249882	0.507	0.059	1.15E-45	14
EPB41L3	3.86E-49	0.434873661	0.725	0.116	7.94E-45	14
DSE	4.49E-49	0.313020705	0.696	0.108	9.24E-45	14
CD163L1	7.62E-49	0.41231802	0.551	0.071	1.57E-44	14
CENPN	9.74E-49	0.373823789	0.652	0.105	2.01E-44	14
MCM4	1.12E-48	0.470474375	0.58	0.084	2.31E-44	14
HCK	1.17E-48	0.562550886	0.87	0.167	2.40E-44	14
REEP4	1.82E-48	0.360256819	0.594	0.088	3.74E-44	14
OTOA	1.99E-48	0.367546231	0.594	0.081	4.09E-44	14
TIMP2	2.88E-48	0.825808347	0.986	0.214	5.93E-44	14
ADAP2	3.21E-48	0.711491302	0.855	0.168	6.60E-44	14
SLC15A3	3.75E-48	0.537394436	0.826	0.149	7.72E-44	14
CD86	3.98E-48	0.561982698	0.884	0.168	8.20E-44	14
WDR76	4.88E-48	0.251305185	0.449	0.049	1.00E-43	14
C1QB	6.33E-48	1.596478912	0.971	0.268	1.30E-43	14
FGD2	6.79E-48	0.328660778	0.696	0.107	1.40E-43	14
SH2B3	1.27E-47	0.544852415	0.899	0.188	2.61E-43	14
SIGLEC10	1.42E-47	0.274586269	0.551	0.07	2.93E-43	14
FCGR2A	1.62E-47	0.837784247	0.942	0.2	3.34E-43	14
CPVL	1.75E-47	1.073173544	1	0.259	3.60E-43	14
FGL2	2.07E-47	1.002485061	0.986	0.237	4.26E-43	14
GNB4	2.72E-47	0.397935837	0.812	0.141	5.60E-43	14
CALHM6	6.10E-47	0.551145698	0.754	0.131	1.26E-42	14
RAB31	6.23E-47	0.62251583	0.87	0.176	1.28E-42	14
PLA2G7	6.39E-47	0.748079626	0.841	0.163	1.32E-42	14
C1QA	7.00E-47	1.568636355	0.971	0.277	1.44E-42	14
MS4A7	9.02E-47	0.875864962	0.942	0.2	1.86E-42	14
BCAT1	1.33E-46	0.401140401	0.725	0.119	2.73E-42	14
MMP9	1.97E-46	1.095136164	0.681	0.118	4.06E-42	14
CBX5	2.27E-46	0.495950078	0.812	0.171	4.67E-42	14
CD93	3.24E-46	0.544059665	0.812	0.153	6.67E-42	14
UNC93B1	4.45E-46	0.644448087	0.913	0.216	9.16E-42	14
ACVRL1	5.64E-46	0.279305555	0.551	0.074	1.16E-41	14
MMP12	5.67E-46	1.488557433	0.449	0.053	1.17E-41	14
DRAM1	1.09E-45	0.504767514	0.754	0.144	2.25E-41	14
SPI1	1.21E-45	0.819755267	0.928	0.208	2.49E-41	14

AKR1B1	1.42E-45	0.562669989	0.87	0.176	2.93E-41	14
CD14	1.56E-45	1.512038339	0.942	0.27	3.22E-41	14
DTYMK	2.29E-45	0.660314332	0.899	0.234	4.71E-41	14
CKAP5	3.86E-45	0.51577896	0.71	0.138	7.96E-41	14
ADA2	1.29E-44	0.713982178	0.971	0.214	2.66E-40	14
LILRB2	1.65E-44	0.494890476	0.797	0.149	3.39E-40	14
CSTA	1.84E-44	0.399217645	0.739	0.134	3.78E-40	14
ARPIN	2.25E-44	0.284935752	0.667	0.113	4.64E-40	14
LAT2	2.26E-44	0.416125763	0.696	0.121	4.65E-40	14
MAFB	2.33E-44	1.116449148	1	0.28	4.80E-40	14
TMEM107	2.35E-44	0.306718065	0.652	0.109	4.85E-40	14
CD180	2.44E-44	0.320447085	0.551	0.078	5.03E-40	14
EMILIN2	6.20E-44	0.465079783	0.739	0.137	1.28E-39	14
PPT1	1.61E-43	0.979400058	1	0.342	3.32E-39	14
CHAF1A	2.17E-43	0.287692182	0.464	0.059	4.47E-39	14
POLD1	2.20E-43	0.262402434	0.478	0.063	4.52E-39	14
HLA-DQA2	2.35E-43	0.884971772	0.986	0.234	4.85E-39	14
AOAH	2.72E-43	0.476280103	0.812	0.155	5.61E-39	14
AIF1	3.13E-43	1.303798397	1	0.305	6.44E-39	14
RNF135	3.47E-43	0.256641739	0.609	0.092	7.14E-39	14
SMC4	4.06E-43	0.88359428	0.942	0.278	8.37E-39	14
C1orf162	5.67E-43	0.823837548	0.957	0.235	1.17E-38	14
FRMD4A	8.66E-43	0.458772883	0.609	0.097	1.78E-38	14
CD300A	1.08E-42	0.57315606	0.826	0.17	2.22E-38	14
CREG1	1.51E-42	0.971777049	0.928	0.249	3.12E-38	14
RB1	1.77E-42	0.617287129	0.855	0.212	3.65E-38	14
SLC9A9	2.07E-42	0.283010448	0.565	0.082	4.25E-38	14
H2AFZ	2.31E-42	1.591896454	1	0.625	4.75E-38	14
NSD2	2.34E-42	0.325541602	0.681	0.126	4.81E-38	14
SLC7A7	2.95E-42	0.529897528	0.754	0.147	6.08E-38	14
SUCNR1	3.08E-42	0.417394019	0.536	0.079	6.34E-38	14
TTYH3	3.10E-42	0.744937839	0.884	0.233	6.38E-38	14
CLEC12A	3.79E-42	0.338150286	0.478	0.061	7.80E-38	14
MTHFD2	5.20E-42	0.462642468	0.812	0.167	1.07E-37	14
TFEC	5.24E-42	0.351878987	0.638	0.106	1.08E-37	14
ABI3	7.70E-42	0.485905992	0.855	0.176	1.59E-37	14
GFRA2	9.39E-42	0.368189467	0.507	0.069	1.93E-37	14
DOCK2	1.12E-41	0.439178452	0.841	0.166	2.32E-37	14
FAR1	1.21E-41	0.285633923	0.681	0.116	2.49E-37	14
NRP2	1.60E-41	0.34551145	0.667	0.111	3.30E-37	14
LILRB1	1.86E-41	0.360899419	0.739	0.135	3.83E-37	14
DNMT1	2.07E-41	0.751702834	0.884	0.244	4.26E-37	14
ATAD5	2.20E-41	0.326029807	0.42	0.051	4.53E-37	14
TUBA1B	2.33E-41	1.841029172	1	0.68	4.80E-37	14
PTTG1	3.03E-41	1.014507637	0.797	0.183	6.23E-37	14

RGL1	3.30E-41	0.383766411	0.638	0.107	6.80E-37	14
SKA2	6.66E-41	0.414123394	0.768	0.166	1.37E-36	14
MPP1	6.82E-41	0.44193594	0.783	0.15	1.40E-36	14
ARHGAP18	6.88E-41	0.424772081	0.812	0.164	1.42E-36	14
SNX24	8.53E-41	0.389948198	0.681	0.131	1.76E-36	14
PAK1	8.76E-41	0.45751799	0.754	0.161	1.80E-36	14
PTAFR	9.24E-41	0.406437843	0.638	0.11	1.90E-36	14
HLA-DMB	1.11E-40	0.781716934	0.971	0.228	2.28E-36	14
JDP2	1.40E-40	0.25828229	0.551	0.081	2.89E-36	14
FES	1.42E-40	0.319510643	0.667	0.12	2.92E-36	14
LST1	1.75E-40	0.769520929	0.971	0.248	3.60E-36	14
BLVRA	2.77E-40	0.615058667	0.913	0.233	5.70E-36	14
HOMER3	2.78E-40	0.301558877	0.623	0.104	5.74E-36	14
SPIC	3.44E-40	0.269361975	0.304	0.027	7.08E-36	14
KPNA2	3.76E-40	1.267960993	0.855	0.243	7.74E-36	14
FKBP15	4.46E-40	0.35254133	0.754	0.155	9.18E-36	14
HVCN1	4.66E-40	0.311576425	0.623	0.105	9.59E-36	14
FCER1G	1.19E-39	1.278084177	1	0.329	2.46E-35	14
CDT1	1.35E-39	0.305117564	0.464	0.064	2.79E-35	14
MSR1	1.69E-39	0.661523923	0.739	0.143	3.48E-35	14
ASRGL1	1.90E-39	0.289572216	0.536	0.087	3.92E-35	14
NRP1	2.02E-39	0.631081158	0.841	0.211	4.16E-35	14
CYBRD1	2.25E-39	0.290098134	0.507	0.072	4.63E-35	14
RAB32	2.27E-39	0.75016053	0.884	0.254	4.68E-35	14
LY86	3.21E-39	0.442159807	0.783	0.156	6.61E-35	14
PARVG	3.28E-39	0.373247957	0.783	0.158	6.76E-35	14
CKS1B	3.36E-39	0.849591357	0.957	0.315	6.92E-35	14
ACER3	6.03E-39	0.431057984	0.696	0.141	1.24E-34	14
LILRB5	8.03E-39	0.362810187	0.623	0.102	1.65E-34	14
GINS2	9.87E-39	0.327109087	0.391	0.047	2.03E-34	14
NAIP	1.60E-38	0.41165375	0.725	0.145	3.29E-34	14
LY96	1.75E-38	0.599121828	0.884	0.205	3.60E-34	14
FANCA	2.05E-38	0.279114505	0.478	0.07	4.21E-34	14
TUBA1C	2.21E-38	1.208091925	0.971	0.359	4.54E-34	14
PLBD1	2.59E-38	0.289934614	0.594	0.1	5.34E-34	14
IL18BP	4.85E-38	0.44189173	0.725	0.142	9.99E-34	14
HMGB2	6.19E-38	1.246444183	0.971	0.38	1.27E-33	14
TUBB	7.99E-38	1.463802044	1	0.684	1.64E-33	14
CTSH	1.13E-37	0.967149206	1	0.374	2.32E-33	14
MKNK1	1.23E-37	0.449920331	0.884	0.217	2.54E-33	14
SLC7A8	1.47E-37	0.285584771	0.507	0.074	3.03E-33	14
SPINT2	1.60E-37	0.3954782	0.725	0.143	3.29E-33	14
KIF22	2.44E-37	0.458274506	0.797	0.208	5.02E-33	14
IMPDH1	2.85E-37	0.253090064	0.551	0.087	5.88E-33	14
ALDH16A1	2.90E-37	0.312095999	0.667	0.136	5.98E-33	14

MCUB	3.26E-37	0.277857043	0.71	0.133	6.71E-33	14
ALDH3B1	3.59E-37	0.283518077	0.522	0.082	7.40E-33	14
NCF2	4.37E-37	0.332351318	0.841	0.179	8.99E-33	14
CR1	5.14E-37	0.280981401	0.449	0.062	1.06E-32	14
SCIMP	6.26E-37	0.25443684	0.623	0.106	1.29E-32	14
IDH2	7.37E-37	0.801739976	0.971	0.366	1.52E-32	14
ARL2	8.95E-37	0.295110507	0.797	0.177	1.84E-32	14
H2AFX	9.83E-37	0.747287386	0.855	0.236	2.03E-32	14
HLA-DMA	1.03E-36	1.018288236	1	0.329	2.13E-32	14
HLA-DRB1	1.51E-36	1.12518881	1	0.333	3.10E-32	14
FCGR3A	1.58E-36	0.932918084	0.884	0.222	3.25E-32	14
1-Mar	1.59E-36	0.469341055	0.768	0.161	3.28E-32	14
SLC43A3	1.72E-36	0.466610398	0.739	0.17	3.54E-32	14
SPRED1	2.28E-36	0.404130143	0.58	0.1	4.70E-32	14
LYL1	2.40E-36	0.272130744	0.594	0.103	4.94E-32	14
MCM6	2.88E-36	0.501331971	0.565	0.101	5.94E-32	14
NAGK	3.02E-36	0.754014405	0.971	0.314	6.22E-32	14
SAE1	3.22E-36	0.343630381	0.725	0.166	6.64E-32	14
CD33	4.74E-36	0.353074457	0.609	0.109	9.77E-32	14
CD4	6.27E-36	0.709700021	0.986	0.262	1.29E-31	14
PTPRE	8.07E-36	0.547909376	0.884	0.211	1.66E-31	14
HLA-DQB1	9.42E-36	0.868870023	0.971	0.274	1.94E-31	14
SIGLEC1	1.08E-35	0.30980609	0.565	0.095	2.22E-31	14
NCKAP1L	1.28E-35	0.529753194	0.928	0.232	2.64E-31	14
CAPG	1.36E-35	0.692706418	0.899	0.259	2.80E-31	14
TMEM109	1.38E-35	0.533315117	0.87	0.253	2.85E-31	14
CSRP1	1.69E-35	0.320771707	0.638	0.12	3.48E-31	14
OGFRL1	2.17E-35	0.442616787	0.739	0.161	4.47E-31	14
SLC16A3	2.18E-35	0.458646691	0.725	0.152	4.48E-31	14
LGALS3	2.24E-35	0.814805008	1	0.287	4.62E-31	14
COLEC12	3.31E-35	0.387836886	0.449	0.065	6.82E-31	14
FERMT3	3.69E-35	0.582215574	0.942	0.248	7.60E-31	14
GSTP1	4.84E-35	0.979482315	1	0.365	9.97E-31	14
RASSF4	5.23E-35	0.555897374	0.899	0.27	1.08E-30	14
FMNL2	5.29E-35	0.276253751	0.551	0.092	1.09E-30	14
ATL3	6.54E-35	0.342208278	0.783	0.19	1.35E-30	14
SLC8A1	6.58E-35	0.303685878	0.638	0.119	1.36E-30	14
TLR4	8.69E-35	0.31847543	0.623	0.118	1.79E-30	14
CMKLR1	9.68E-35	0.332988297	0.609	0.111	1.99E-30	14
HELLS	1.00E-34	0.378077894	0.522	0.091	2.06E-30	14
SLC1A5	1.03E-34	0.255059771	0.638	0.12	2.11E-30	14
SAMHD1	1.07E-34	0.963955976	0.986	0.375	2.21E-30	14
AP1B1	1.17E-34	0.462554516	0.841	0.23	2.41E-30	14
AP2S1	1.25E-34	0.932420478	1	0.541	2.57E-30	14
ANXA5	1.30E-34	0.934001227	1	0.428	2.69E-30	14

GPNMB	1.42E-34	0.578994597	0.797	0.184	2.92E-30	14
CD68	1.70E-34	1.146946907	0.957	0.341	3.51E-30	14
TMPO	1.89E-34	0.634632427	0.899	0.29	3.90E-30	14
NAAA	2.71E-34	0.307702594	0.768	0.169	5.57E-30	14
DMXL2	2.87E-34	0.483603435	0.768	0.189	5.91E-30	14
TMIGD3	3.13E-34	0.382911302	0.464	0.069	6.44E-30	14
HMGB1	4.08E-34	1.050685082	1	0.837	8.41E-30	14
DAPK1	4.21E-34	0.411480123	0.725	0.175	8.67E-30	14
CD1C	4.61E-34	0.522137127	0.377	0.048	9.49E-30	14
RAB34	5.59E-34	0.322960846	0.739	0.182	1.15E-29	14
KNSTRN	6.40E-34	0.343711527	0.42	0.063	1.32E-29	14
CYTH4	6.89E-34	0.341195776	0.812	0.179	1.42E-29	14
CNTRL	1.16E-33	0.359506237	0.725	0.165	2.39E-29	14
LGALS9	1.23E-33	0.535100651	0.841	0.208	2.54E-29	14
LGMN	1.32E-33	1.09524913	0.942	0.311	2.71E-29	14
HLA-DOA	1.33E-33	0.322075307	0.652	0.126	2.73E-29	14
HLA-DQA1	1.34E-33	0.846355481	0.942	0.262	2.75E-29	14
THBD	1.43E-33	0.421769877	0.449	0.067	2.95E-29	14
RBBP8	1.52E-33	0.333031526	0.565	0.11	3.12E-29	14
HACD4	1.70E-33	0.270320949	0.594	0.109	3.49E-29	14
PKM	2.93E-33	0.900148159	1	0.377	6.03E-29	14
GIMAP7	3.06E-33	0.478317015	0.87	0.21	6.30E-29	14
AXL	3.25E-33	0.371708893	0.522	0.089	6.70E-29	14
FAM49A	4.01E-33	0.321236343	0.725	0.153	8.26E-29	14
THEMIS2	4.38E-33	0.420811129	0.841	0.192	9.02E-29	14
TREM2	4.56E-33	0.528201851	0.551	0.096	9.40E-29	14
SNRNP25	4.60E-33	0.4708364	0.783	0.217	9.47E-29	14
LILRB4	4.99E-33	0.369601978	0.667	0.134	1.03E-28	14
DDX39A	5.32E-33	0.545510257	0.841	0.246	1.10E-28	14
PLEK	6.27E-33	0.507668108	0.957	0.254	1.29E-28	14
TCN2	7.29E-33	0.284628641	0.58	0.109	1.50E-28	14
LYN	9.35E-33	0.515693809	0.899	0.243	1.92E-28	14
GLIPR1	1.00E-32	0.583402078	0.957	0.268	2.07E-28	14
CKAP2	1.15E-32	0.443640614	0.551	0.107	2.38E-28	14
NCEH1	1.50E-32	0.277185266	0.522	0.091	3.08E-28	14
IFNGR1	1.55E-32	0.74708193	0.899	0.282	3.19E-28	14
VSIR	2.10E-32	0.508189875	0.884	0.23	4.31E-28	14
NPC2	2.76E-32	1.19286757	1	0.548	5.69E-28	14
BMP2K	3.07E-32	0.304036883	0.71	0.157	6.32E-28	14
TYROBP	3.53E-32	1.01739106	1	0.323	7.28E-28	14
DPYSL2	4.04E-32	0.456951959	0.855	0.231	8.32E-28	14
YIF1B	5.02E-32	0.377943304	0.71	0.181	1.03E-27	14
LMO2	5.78E-32	0.284050759	0.638	0.128	1.19E-27	14
WDFY2	6.01E-32	0.409345678	0.768	0.204	1.24E-27	14
CD84	6.63E-32	0.39750688	0.841	0.195	1.37E-27	14

RNASEH2B	6.91E-32	0.386252893	0.826	0.228	1.42E-27	14
C3AR1	8.05E-32	0.46214912	0.696	0.149	1.66E-27	14
EZH2	8.26E-32	0.319432763	0.667	0.15	1.70E-27	14
VMA21	1.03E-31	0.532180027	0.884	0.302	2.13E-27	14
RAB20	1.11E-31	0.39720324	0.681	0.147	2.29E-27	14
DEK	1.34E-31	0.845272021	1	0.588	2.75E-27	14
MFSD1	1.37E-31	0.577547877	0.899	0.291	2.83E-27	14
SNX10	1.60E-31	0.681046529	0.884	0.279	3.29E-27	14
GSN	1.66E-31	0.613796596	0.971	0.299	3.41E-27	14
TNFSF13B	2.21E-31	0.260680923	0.855	0.19	4.55E-27	14
MIS18BP1	2.52E-31	0.458351727	0.812	0.224	5.19E-27	14
SCLT1	2.55E-31	0.266672591	0.58	0.113	5.26E-27	14
SLC1A3	2.55E-31	0.283396032	0.507	0.087	5.26E-27	14
RNPEP	2.58E-31	0.528180004	0.812	0.245	5.32E-27	14
LGALS3BP	2.72E-31	0.369662255	0.681	0.15	5.60E-27	14
HLA-DPA1	2.81E-31	1.174929077	1	0.452	5.79E-27	14
DNAJC9	3.28E-31	0.405344343	0.638	0.148	6.75E-27	14
VAMP8	3.35E-31	0.958717873	1	0.549	6.91E-27	14
PCMT1	3.49E-31	0.477097115	0.884	0.281	7.19E-27	14
GLA	3.72E-31	0.434790348	0.754	0.2	7.66E-27	14
M6PR	4.30E-31	0.725703308	0.986	0.4	8.86E-27	14
HAVCR2	4.49E-31	0.371397247	0.754	0.172	9.25E-27	14
WAS	5.47E-31	0.344390517	0.812	0.191	1.13E-26	14
RPS6KA1	6.26E-31	0.261549785	0.681	0.149	1.29E-26	14
CTSZ	6.44E-31	1.005356477	1	0.557	1.33E-26	14
PLTP	6.58E-31	0.332958029	0.696	0.153	1.35E-26	14
RHNO1	7.83E-31	0.259586714	0.507	0.093	1.61E-26	14
BARD1	8.18E-31	0.349338543	0.609	0.133	1.69E-26	14
HLA-DPB1	8.53E-31	1.049707073	1	0.394	1.76E-26	14
ITGB2	8.56E-31	0.843867095	1	0.356	1.76E-26	14
ENTPD1	9.01E-31	0.292809715	0.826	0.196	1.86E-26	14
RRM1	9.52E-31	0.407225941	0.609	0.138	1.96E-26	14
PLD3	9.80E-31	0.852144729	0.928	0.347	2.02E-26	14
ADAM9	1.02E-30	0.365931111	0.754	0.19	2.11E-26	14
PLEKHO1	1.19E-30	0.508571255	0.928	0.243	2.46E-26	14
ZEB2	1.36E-30	0.580373274	0.913	0.248	2.80E-26	14
GRN	1.89E-30	0.991981972	1	0.561	3.89E-26	14
CD72	2.00E-30	0.274171197	0.58	0.11	4.12E-26	14
BLVRB	2.05E-30	0.940797366	0.986	0.461	4.23E-26	14
POLD3	2.23E-30	0.273861703	0.493	0.093	4.59E-26	14
SHTN1	2.25E-30	0.333218766	0.768	0.2	4.63E-26	14
LILRB3	2.31E-30	0.314456596	0.652	0.137	4.76E-26	14
CYFIP1	2.54E-30	0.422012514	0.87	0.267	5.24E-26	14
VMO1	2.59E-30	0.300676872	0.522	0.095	5.33E-26	14
LPCAT2	2.81E-30	0.253305125	0.58	0.118	5.79E-26	14

DPP7	3.62E-30	0.517652366	0.957	0.29	7.45E-26	14
PLAUR	4.07E-30	0.301160511	0.754	0.173	8.39E-26	14
CXCL16	4.11E-30	0.475925862	0.913	0.276	8.46E-26	14
SAP30	4.89E-30	0.385694506	0.696	0.179	1.01E-25	14
MCM2	5.20E-30	0.307351362	0.406	0.065	1.07E-25	14
IFI16	5.51E-30	0.681407375	0.986	0.337	1.14E-25	14
ARHGAP4	6.39E-30	0.444951328	0.855	0.239	1.32E-25	14
CCDC88A	6.93E-30	0.620693149	0.971	0.382	1.43E-25	14
SGPL1	7.89E-30	0.41665676	0.768	0.22	1.62E-25	14
TUBA1A	7.89E-30	0.815856067	0.899	0.297	1.63E-25	14
LMO4	8.01E-30	0.342602716	0.783	0.191	1.65E-25	14
TNFAIP8L2	8.53E-30	0.328133274	0.58	0.115	1.76E-25	14
CYBA	9.46E-30	0.969851186	1	0.536	1.95E-25	14
SLCO2B1	1.01E-29	0.523888663	0.783	0.221	2.09E-25	14
HIST1H4C	1.12E-29	1.385077001	0.884	0.338	2.31E-25	14
DUT	1.26E-29	0.812662345	0.884	0.34	2.60E-25	14
CST3	1.38E-29	1.167007554	1	0.62	2.85E-25	14
TCEAL3	1.51E-29	0.295617713	0.449	0.075	3.10E-25	14
TSPAN4	1.56E-29	0.601688623	0.884	0.297	3.21E-25	14
CTSS	1.59E-29	0.845933357	1	0.369	3.27E-25	14
RABL6	2.47E-29	0.389704934	0.754	0.206	5.08E-25	14
RGS10	2.54E-29	0.545287907	0.957	0.274	5.23E-25	14
DEGS1	2.97E-29	0.386248789	0.855	0.248	6.13E-25	14
CCL2	3.36E-29	1.109524855	0.435	0.071	6.93E-25	14
ITGAM	3.86E-29	0.255158446	0.58	0.113	7.95E-25	14
PCNA	4.55E-29	0.982735558	0.797	0.273	9.38E-25	14
SNN	4.57E-29	0.261590071	0.594	0.125	9.41E-25	14
RNF130	4.57E-29	0.566211897	0.928	0.307	9.42E-25	14
CDKN2C	5.74E-29	0.47043042	0.696	0.192	1.18E-24	14
NIN	6.87E-29	0.292487829	0.696	0.172	1.41E-24	14
AP1S2	7.28E-29	0.506621998	0.884	0.261	1.50E-24	14
ITGA4	8.83E-29	0.331866346	0.681	0.148	1.82E-24	14
GIMAP8	9.75E-29	0.298529861	0.493	0.089	2.01E-24	14
RCC2	1.05E-28	0.329449234	0.725	0.195	2.16E-24	14
LACC1	1.19E-28	0.384415485	0.522	0.107	2.45E-24	14
HLA-DRA	1.39E-28	1.185760004	1	0.52	2.86E-24	14
H2AFV	1.45E-28	0.892144766	0.986	0.539	2.99E-24	14
ATG3	1.50E-28	0.562304941	0.928	0.359	3.09E-24	14
FSCN1	1.60E-28	0.254985127	0.58	0.114	3.29E-24	14
YWHAH	1.81E-28	0.749956364	0.928	0.41	3.73E-24	14
HMGN2	2.76E-28	1.343233536	0.942	0.56	5.68E-24	14
MFSD12	2.93E-28	0.309954048	0.725	0.187	6.04E-24	14
GNPTAB	2.97E-28	0.29233316	0.841	0.239	6.12E-24	14
ACP5	3.26E-28	0.547098236	0.783	0.201	6.72E-24	14
ADGRE2	3.99E-28	0.270640899	0.551	0.109	8.21E-24	14

PLXNC1	4.27E-28	0.270960042	0.594	0.129	8.79E-24	14
ATG4C	4.29E-28	0.260502744	0.536	0.112	8.83E-24	14
RHOQ	4.50E-28	0.345498552	0.812	0.221	9.26E-24	14
CD74	5.93E-28	1.213611795	1	0.622	1.22E-23	14
LCP1	6.70E-28	0.835419555	1	0.415	1.38E-23	14
SIRPA	7.93E-28	0.489014216	0.768	0.236	1.63E-23	14
LGALS2	1.02E-27	0.358448426	0.652	0.143	2.10E-23	14
ME2	1.13E-27	0.353379674	0.739	0.199	2.32E-23	14
GBA	1.19E-27	0.388270404	0.739	0.218	2.44E-23	14
CASP1	1.19E-27	0.472649475	0.884	0.253	2.45E-23	14
SSRP1	1.20E-27	0.340021433	0.783	0.239	2.47E-23	14
SPCS3	1.22E-27	0.621076901	0.971	0.417	2.52E-23	14
FRMD4B	1.26E-27	0.394667294	0.739	0.192	2.59E-23	14
MCM5	1.35E-27	0.479424471	0.652	0.17	2.79E-23	14
EPSTI1	1.39E-27	0.275915524	0.739	0.177	2.87E-23	14
PSAP	1.58E-27	1.088483943	1	0.643	3.25E-23	14
ATP6V1B2	1.86E-27	0.564348231	0.884	0.317	3.83E-23	14
PHACTR1	2.11E-27	0.425925373	0.609	0.132	4.35E-23	14
FLII	2.37E-27	0.303047924	0.768	0.219	4.88E-23	14
HK1	2.49E-27	0.27539004	0.638	0.14	5.13E-23	14
NPL	2.52E-27	0.364924476	0.638	0.141	5.20E-23	14
ADCY7	3.25E-27	0.300210976	0.623	0.138	6.69E-23	14
LAPTM5	3.28E-27	0.876486847	1	0.449	6.76E-23	14
TPM4	4.34E-27	0.624111888	0.986	0.37	8.94E-23	14
SERPINB6	4.81E-27	0.445627825	0.899	0.29	9.90E-23	14
COPS3	5.63E-27	0.359390983	0.739	0.214	1.16E-22	14
GM2A	7.49E-27	0.450830064	0.739	0.215	1.54E-22	14
NUCKS1	7.69E-27	0.81973291	1	0.59	1.58E-22	14
IFI30	1.10E-26	0.34026703	0.58	0.128	2.26E-22	14
NAGA	1.11E-26	0.407472519	0.725	0.217	2.28E-22	14
LGALS1	1.31E-26	1.165759495	1	0.59	2.69E-22	14
UCP2	1.34E-26	0.619939427	0.986	0.341	2.75E-22	14
PPIA	1.95E-26	0.683305623	1	0.846	4.01E-22	14
IQGAP1	1.96E-26	0.542881767	1	0.341	4.04E-22	14
ETV5	2.03E-26	0.416669208	0.681	0.199	4.17E-22	14
MEF2C	2.06E-26	0.27254612	0.768	0.196	4.24E-22	14
SMIM25	2.35E-26	0.261386959	0.478	0.09	4.85E-22	14
RAD21	3.03E-26	0.650254699	0.957	0.419	6.24E-22	14
SLC40A1	3.12E-26	0.926819565	0.913	0.359	6.42E-22	14
CKS2	3.32E-26	0.649704403	0.754	0.238	6.84E-22	14
BRI3BP	3.37E-26	0.406170618	0.609	0.165	6.93E-22	14
FAM110A	3.90E-26	0.256952376	0.594	0.133	8.03E-22	14
CTSB	4.81E-26	1.069084163	1	0.652	9.90E-22	14
F13A1	4.98E-26	0.389134929	0.42	0.073	1.03E-21	14
JPT1	5.13E-26	0.888211418	0.928	0.429	1.06E-21	14

VEGFB	5.26E-26	0.386452998	0.899	0.312	1.08E-21	14
PGD	5.42E-26	0.343955602	0.768	0.242	1.12E-21	14
PLP2	5.67E-26	0.383953296	0.71	0.183	1.17E-21	14
PYCARD	6.07E-26	0.64615714	0.899	0.323	1.25E-21	14
NUP58	6.55E-26	0.362351239	0.681	0.188	1.35E-21	14
KCNK6	8.02E-26	0.313840496	0.551	0.117	1.65E-21	14
HNRNPAB	8.63E-26	0.573102582	0.884	0.348	1.78E-21	14
HDAC2	8.82E-26	0.36645113	0.783	0.255	1.82E-21	14
ADPGK	8.95E-26	0.375829061	0.841	0.275	1.84E-21	14
RNASET2	1.11E-25	0.665813756	0.986	0.379	2.28E-21	14
SCPEP1	1.21E-25	0.448804265	0.884	0.317	2.48E-21	14
PSMC1	1.21E-25	0.426786931	0.855	0.301	2.49E-21	14
CD63	1.24E-25	0.946644922	1	0.739	2.55E-21	14
IFNGR2	1.28E-25	0.377575773	0.855	0.28	2.64E-21	14
LARP7	1.69E-25	0.357584939	0.841	0.284	3.47E-21	14
RHOG	1.91E-25	0.556271228	0.971	0.374	3.93E-21	14
IRF8	1.95E-25	0.281831586	0.667	0.157	4.01E-21	14
ACTR1A	1.95E-25	0.26458139	0.71	0.2	4.02E-21	14
TFDP1	1.96E-25	0.252136123	0.623	0.156	4.04E-21	14
PTPN6	2.13E-25	0.472936748	0.884	0.281	4.39E-21	14
TMEM106C	2.36E-25	0.480196016	0.783	0.26	4.85E-21	14
SNX2	2.42E-25	0.619932162	0.855	0.332	4.98E-21	14
GNPDA1	2.47E-25	0.490546685	0.667	0.194	5.09E-21	14
USP1	2.84E-25	0.363971776	0.725	0.215	5.84E-21	14
RGS19	3.14E-25	0.376826718	0.754	0.212	6.47E-21	14
ACTB	3.20E-25	0.873772029	1	0.946	6.59E-21	14
H2AFY	3.29E-25	0.716165566	0.957	0.433	6.77E-21	14
EXOSC8	3.37E-25	0.281492213	0.71	0.209	6.94E-21	14
CNPY3	3.94E-25	0.551230993	0.899	0.359	8.12E-21	14
TTC1	4.10E-25	0.278980561	0.812	0.256	8.45E-21	14
LIPA	5.29E-25	0.763242986	0.942	0.404	1.09E-20	14
PLIN3	5.77E-25	0.440157102	0.754	0.238	1.19E-20	14
SGK3	7.08E-25	0.277594647	0.696	0.198	1.46E-20	14
TGFB1	7.28E-25	0.300606866	0.884	0.251	1.50E-20	14
MCOLN1	7.78E-25	0.342285992	0.681	0.184	1.60E-20	14
TCIRG1	1.04E-24	0.378884064	0.841	0.276	2.14E-20	14
FUCA2	1.09E-24	0.448552907	0.783	0.273	2.26E-20	14
TMEM173	1.23E-24	0.329204642	0.681	0.171	2.54E-20	14
CTSC	1.27E-24	0.833739236	0.986	0.419	2.62E-20	14
PLEKHB2	1.30E-24	0.45396117	0.899	0.321	2.69E-20	14
NTAN1	1.41E-24	0.301092185	0.812	0.258	2.90E-20	14
BUB3	1.50E-24	0.485361397	0.942	0.385	3.09E-20	14
C4orf48	1.56E-24	0.281444972	0.797	0.215	3.21E-20	14
SLC25A24	1.58E-24	0.252139253	0.652	0.177	3.24E-20	14
CLEC2B	1.67E-24	0.455368519	0.971	0.319	3.43E-20	14

HCLS1	1.89E-24	0.550750236	0.957	0.314	3.88E-20	14
SUZ12	2.06E-24	0.296462023	0.797	0.255	4.24E-20	14
ATP6V1C1	2.19E-24	0.334677441	0.739	0.237	4.51E-20	14
MPDU1	2.21E-24	0.306370142	0.681	0.201	4.54E-20	14
S100A11	2.26E-24	0.830403163	1	0.491	4.66E-20	14
SLC39A8	2.31E-24	0.279512212	0.536	0.13	4.76E-20	14
PREX1	2.53E-24	0.311274625	0.739	0.198	5.21E-20	14
COMMD9	2.57E-24	0.30102474	0.768	0.246	5.29E-20	14
COMMD4	2.80E-24	0.442194761	0.826	0.304	5.76E-20	14
LCP2	3.05E-24	0.397511957	0.87	0.254	6.28E-20	14
CMTM7	3.15E-24	0.257056582	0.725	0.205	6.48E-20	14
MIF4GD	3.31E-24	0.271448404	0.652	0.174	6.81E-20	14
PPIH	3.48E-24	0.255093073	0.623	0.164	7.16E-20	14
LSM4	3.68E-24	0.585342217	0.928	0.43	7.58E-20	14
KIAA0930	4.16E-24	0.346107337	0.841	0.295	8.57E-20	14
FCGRT	4.22E-24	0.785965581	1	0.538	8.70E-20	14
TMEM106A	4.51E-24	0.277016545	0.609	0.158	9.29E-20	14
DNAJC15	4.75E-24	0.407342005	0.899	0.341	9.78E-20	14
ACTN1	5.69E-24	0.474884009	0.841	0.333	1.17E-19	14
PRNP	7.07E-24	0.272787532	0.768	0.202	1.46E-19	14
VPS29	7.94E-24	0.62116554	0.971	0.515	1.63E-19	14
ARL8B	8.46E-24	0.36077922	0.855	0.321	1.74E-19	14
SLC38A6	8.64E-24	0.283323144	0.594	0.154	1.78E-19	14
DCP2	1.04E-23	0.271439503	0.768	0.237	2.15E-19	14
BST2	1.06E-23	0.512655769	0.971	0.332	2.17E-19	14
CLEC11A	1.14E-23	0.301841474	0.391	0.071	2.34E-19	14
RTN3	1.16E-23	0.461744623	0.855	0.343	2.39E-19	14
PLXNB2	1.16E-23	0.35547843	0.797	0.271	2.39E-19	14
ASAH1	1.26E-23	0.70357486	0.986	0.464	2.59E-19	14
COTL1	1.28E-23	0.753428018	1	0.41	2.63E-19	14
RBBP7	1.55E-23	0.402368767	0.783	0.274	3.19E-19	14
PEA15	1.71E-23	0.403859906	0.826	0.299	3.52E-19	14
RPA3	1.72E-23	0.521547114	0.913	0.381	3.55E-19	14
EHBP1L1	1.90E-23	0.322854192	0.841	0.273	3.91E-19	14
MYO9B	1.91E-23	0.269594547	0.754	0.214	3.94E-19	14
ANXA2	2.01E-23	0.838399471	0.986	0.624	4.14E-19	14
MARCKS	2.03E-23	0.921119372	0.986	0.541	4.17E-19	14
FABP5	2.18E-23	0.739445115	0.957	0.4	4.49E-19	14
SNX9	2.24E-23	0.265424462	0.725	0.208	4.62E-19	14
ARRB2	2.71E-23	0.37648797	0.87	0.283	5.57E-19	14
EIF4EBP1	2.87E-23	0.364099314	0.841	0.309	5.91E-19	14
NAA38	3.07E-23	0.449161427	0.899	0.352	6.33E-19	14
HBEGF	3.58E-23	0.298497797	0.493	0.104	7.38E-19	14
CCDC112	3.61E-23	0.25374689	0.565	0.143	7.44E-19	14
EFHD2	3.74E-23	0.522928745	0.986	0.407	7.69E-19	14

YBX1	3.86E-23	0.640924503	1	0.792	7.95E-19	14
COX8A	4.39E-23	0.683338511	1	0.633	9.04E-19	14
RPA1	4.75E-23	0.282707747	0.696	0.212	9.79E-19	14
TOR3A	4.76E-23	0.265429646	0.638	0.177	9.81E-19	14
YBX3	4.87E-23	0.258986449	0.826	0.227	1.00E-18	14
HMOX1	4.88E-23	0.809032086	0.768	0.233	1.01E-18	14
CORO1C	5.52E-23	0.367232268	0.696	0.229	1.14E-18	14
RAN	6.11E-23	0.716766169	0.986	0.655	1.26E-18	14
ELOVL1	6.13E-23	0.281626326	0.725	0.232	1.26E-18	14
TACC1	7.09E-23	0.392705613	0.928	0.323	1.46E-18	14
PTGS2	7.53E-23	0.254871369	0.406	0.076	1.55E-18	14
ALYREF	9.74E-23	0.378528595	0.812	0.302	2.01E-18	14
LYZ	1.56E-22	0.994512209	0.986	0.613	3.22E-18	14
GAA	2.01E-22	0.385904219	0.812	0.289	4.14E-18	14
WASHC3	2.11E-22	0.308333552	0.739	0.242	4.34E-18	14
PGP	2.33E-22	0.333853585	0.826	0.296	4.80E-18	14
VIM	2.98E-22	0.812409717	1	0.576	6.13E-18	14
DICER1	3.30E-22	0.299203471	0.797	0.266	6.79E-18	14
ACTR2	3.34E-22	0.655226255	1	0.566	6.88E-18	14
PARP1	3.58E-22	0.578616842	0.942	0.407	7.38E-18	14
RAC1	3.66E-22	0.659174925	1	0.69	7.55E-18	14
COPRS	3.99E-22	0.314843396	0.783	0.275	8.22E-18	14
NASP	4.04E-22	0.62868026	0.855	0.367	8.33E-18	14
SCARB2	4.06E-22	0.567964329	0.841	0.35	8.37E-18	14
SHKBP1	4.51E-22	0.291829836	0.725	0.222	9.30E-18	14
CERS6	5.33E-22	0.296236926	0.623	0.184	1.10E-17	14
COLGALT1	5.56E-22	0.390333457	0.696	0.223	1.14E-17	14
IL17RA	5.69E-22	0.263939927	0.58	0.153	1.17E-17	14
ATP6V0D1	5.70E-22	0.4497275	0.899	0.375	1.17E-17	14
ANXA1	7.42E-22	0.3821786	0.971	0.328	1.53E-17	14
TAOK3	8.05E-22	0.448015504	0.928	0.396	1.66E-17	14
CTSD	8.21E-22	0.988258253	1	0.684	1.69E-17	14
MYO1F	9.27E-22	0.271310102	0.768	0.223	1.91E-17	14
PRKCB	9.36E-22	0.293242899	0.652	0.17	1.93E-17	14
AKR1A1	9.42E-22	0.547295264	0.942	0.453	1.94E-17	14
EVI2B	1.10E-21	0.312663819	0.812	0.235	2.26E-17	14
OSTF1	1.15E-21	0.389387665	0.913	0.353	2.38E-17	14
NDUFA9	1.16E-21	0.282083764	0.797	0.279	2.39E-17	14
ARPC5	1.24E-21	0.657205389	1	0.605	2.55E-17	14
OAZ1	1.33E-21	0.676647501	1	0.802	2.75E-17	14
TSPO	1.39E-21	0.557580898	0.928	0.396	2.87E-17	14
BLOC1S1	1.44E-21	0.509027769	0.986	0.506	2.97E-17	14
IL10RA	1.46E-21	0.304277261	0.841	0.247	3.02E-17	14
WSB1	1.47E-21	0.757603407	0.986	0.542	3.03E-17	14
QKI	1.63E-21	0.441791473	0.957	0.409	3.36E-17	14

KPNB1	1.73E-21	0.569126071	0.986	0.483	3.57E-17	14
UBE2S	1.82E-21	0.715033641	0.87	0.361	3.75E-17	14
DBNL	2.07E-21	0.404004636	0.884	0.353	4.27E-17	14
SKAP2	2.51E-21	0.400215122	0.855	0.317	5.17E-17	14
IKBIP	2.61E-21	0.307708541	0.725	0.246	5.38E-17	14
PLXND1	2.90E-21	0.253454311	0.681	0.208	5.97E-17	14
NANS	3.06E-21	0.377449714	0.812	0.318	6.31E-17	14
SNX8	3.35E-21	0.256662385	0.58	0.16	6.90E-17	14
CFL1	3.41E-21	0.673924487	1	0.842	7.01E-17	14
SOX4	3.47E-21	0.461011503	0.565	0.138	7.14E-17	14
RNH1	4.21E-21	0.532673156	0.942	0.45	8.66E-17	14
NUCB2	4.43E-21	0.478364997	0.754	0.29	9.13E-17	14
PTMA	4.99E-21	0.524932569	1	0.954	1.03E-16	14
JAML	5.43E-21	0.414965923	0.725	0.232	1.12E-16	14
NSL1	7.23E-21	0.324314858	0.841	0.319	1.49E-16	14
SCAMP2	7.36E-21	0.390867368	0.87	0.34	1.51E-16	14
FXYD5	7.96E-21	0.43710231	0.971	0.355	1.64E-16	14
SNX18	1.00E-20	0.255135115	0.609	0.171	2.06E-16	14
HEXA	1.10E-20	0.343344897	0.855	0.334	2.27E-16	14
ABRACL	1.12E-20	0.329531153	0.913	0.292	2.32E-16	14
GRB2	1.13E-20	0.489145974	0.957	0.45	2.34E-16	14
MYL6	1.15E-20	0.630124554	1	0.855	2.38E-16	14
CALM3	1.25E-20	0.60786381	0.971	0.512	2.58E-16	14
SMC1A	1.26E-20	0.319985147	0.739	0.254	2.59E-16	14
MAPRE1	1.28E-20	0.371422341	0.855	0.332	2.64E-16	14
TRIOBP	1.37E-20	0.280961317	0.768	0.276	2.83E-16	14
TUBB4B	1.43E-20	0.80131352	1	0.583	2.95E-16	14
TNFAIP2	1.48E-20	0.337556024	0.739	0.226	3.05E-16	14
C20orf27	1.50E-20	0.362801209	0.841	0.318	3.09E-16	14
SLC25A5	1.52E-20	0.670320592	0.986	0.646	3.12E-16	14
TNFRSF1A	1.52E-20	0.350742478	0.841	0.31	3.14E-16	14
TYMP	1.53E-20	0.694049394	0.971	0.492	3.14E-16	14
ZDHHC12	1.60E-20	0.267221302	0.667	0.215	3.29E-16	14
EPB41L2	1.63E-20	0.259621137	0.681	0.21	3.36E-16	14
RER1	1.66E-20	0.42232736	0.942	0.443	3.43E-16	14
SEC14L1	1.83E-20	0.259391839	0.768	0.258	3.76E-16	14
ARHGDIA	1.87E-20	0.474153851	0.986	0.518	3.84E-16	14
OAZ2	2.12E-20	0.368998071	0.899	0.351	4.36E-16	14
FIBP	2.46E-20	0.296802491	0.826	0.315	5.06E-16	14
CHMP4A	3.32E-20	0.265155298	0.841	0.267	6.83E-16	14
CD81	3.96E-20	0.550877944	0.986	0.405	8.15E-16	14
MGAT1	3.99E-20	0.428561923	0.913	0.377	8.22E-16	14
CNDP2	4.25E-20	0.450745613	0.87	0.372	8.75E-16	14
VDAC3	4.43E-20	0.438656565	0.899	0.406	9.13E-16	14
GABARAP	4.95E-20	0.34893851	0.797	0.307	1.02E-15	14

TMED9	4.99E-20	0.477994114	0.942	0.482	1.03E-15	14
PSMC3	5.78E-20	0.412246382	0.855	0.378	1.19E-15	14
MINOS1	6.16E-20	0.511041635	1	0.53	1.27E-15	14
LDHB	6.40E-20	0.367527283	0.942	0.352	1.32E-15	14
CAPZA1	6.53E-20	0.438231815	0.986	0.482	1.35E-15	14
SLC31A1	6.81E-20	0.258037737	0.71	0.243	1.40E-15	14
PQLC3	7.05E-20	0.263153286	0.594	0.164	1.45E-15	14
PFN1	7.91E-20	0.697415899	1	0.824	1.63E-15	14
CEP350	7.92E-20	0.272280679	0.826	0.304	1.63E-15	14
ARHGDIB	8.72E-20	0.708729376	1	0.508	1.80E-15	14
PIK3AP1	1.00E-19	0.342420394	0.739	0.261	2.06E-15	14
ZYX	1.31E-19	0.369358196	0.826	0.292	2.71E-15	14
BCKDK	1.48E-19	0.282593846	0.725	0.26	3.05E-15	14
TWF2	1.71E-19	0.333119234	0.884	0.37	3.52E-15	14
DCAF12	1.73E-19	0.272221128	0.638	0.206	3.56E-15	14
STXBP2	1.78E-19	0.292936183	0.725	0.236	3.66E-15	14
SLC20A1	1.92E-19	0.361374401	0.797	0.287	3.96E-15	14
PID1	1.93E-19	0.303887417	0.551	0.162	3.97E-15	14
ATP13A2	2.00E-19	0.259684153	0.42	0.097	4.12E-15	14
MAF	2.16E-19	0.641616777	0.899	0.434	4.45E-15	14
MRPS16	2.37E-19	0.283325576	0.913	0.366	4.88E-15	14
MCM7	2.50E-19	0.455413948	0.652	0.225	5.15E-15	14
STX7	2.75E-19	0.283677005	0.725	0.243	5.67E-15	14
CSK	3.67E-19	0.274516137	0.884	0.318	7.57E-15	14
BNIP2	4.06E-19	0.302637129	0.783	0.293	8.37E-15	14
ATP1B3	4.09E-19	0.441671837	0.899	0.369	8.42E-15	14
RBM47	4.12E-19	0.337098336	0.739	0.26	8.49E-15	14
NUP214	4.88E-19	0.315127334	0.638	0.208	1.01E-14	14
PTBP3	5.47E-19	0.337419536	0.826	0.334	1.13E-14	14
TUBG1	5.58E-19	0.275239987	0.58	0.181	1.15E-14	14
NUTF2	6.08E-19	0.284146403	0.841	0.351	1.25E-14	14
TMSB4X	6.22E-19	0.740526233	1	0.825	1.28E-14	14
MMD	6.26E-19	0.340402205	0.652	0.221	1.29E-14	14
FAM49B	7.30E-19	0.402791971	0.971	0.419	1.50E-14	14
ARPC3	8.23E-19	0.6434477	1	0.755	1.70E-14	14
CAPZB	8.75E-19	0.568901993	1	0.615	1.80E-14	14
GNAI2	9.44E-19	0.659759385	1	0.704	1.94E-14	14
TBC1D9	9.93E-19	0.256730643	0.493	0.129	2.04E-14	14
ACP2	1.02E-18	0.34270881	0.725	0.26	2.10E-14	14
MCM3	1.03E-18	0.39035802	0.594	0.193	2.12E-14	14
ARL4C	1.09E-18	0.518339511	0.942	0.355	2.24E-14	14
POLE4	1.11E-18	0.257393139	0.71	0.242	2.28E-14	14
PPP1R18	1.25E-18	0.285605416	0.899	0.316	2.58E-14	14
ARL6IP1	1.37E-18	1.04787192	0.986	0.555	2.82E-14	14
HAT1	1.44E-18	0.251052694	0.71	0.252	2.96E-14	14

ANP32B	1.68E-18	0.592586017	0.957	0.568	3.47E-14	14
BID	1.69E-18	0.386855551	0.826	0.354	3.48E-14	14
KHSRP	1.77E-18	0.319903749	0.71	0.255	3.64E-14	14
PRMT2	2.02E-18	0.258250018	0.768	0.27	4.16E-14	14
CTNND1	2.05E-18	0.270121202	0.667	0.227	4.23E-14	14
NDUFS8	2.08E-18	0.375537745	0.986	0.469	4.28E-14	14
ATP6V0B	2.10E-18	0.598743392	0.986	0.568	4.32E-14	14
BAZ1A	2.17E-18	0.298803219	0.87	0.343	4.48E-14	14
SUGP2	2.31E-18	0.279603224	0.667	0.229	4.75E-14	14
TAGLN2	2.61E-18	0.574245382	0.942	0.467	5.37E-14	14
HIF1A	2.67E-18	0.28098143	0.797	0.299	5.50E-14	14
TXNDC12	2.75E-18	0.366498492	0.783	0.315	5.66E-14	14
POLE3	2.82E-18	0.284015022	0.71	0.26	5.80E-14	14
TECR	3.30E-18	0.383115969	0.942	0.461	6.80E-14	14
PDXK	3.33E-18	0.374053559	0.812	0.336	6.86E-14	14
CCL3L1	3.81E-18	0.336326324	0.696	0.221	7.85E-14	14
PSMA4	4.09E-18	0.516877863	0.942	0.504	8.41E-14	14
RHOA	4.13E-18	0.591057624	0.986	0.713	8.50E-14	14
CTSL	4.28E-18	0.661962359	0.841	0.392	8.82E-14	14
TMEM167A	4.42E-18	0.366433817	0.87	0.396	9.11E-14	14
UBE2A	4.55E-18	0.325549315	0.783	0.311	9.38E-14	14
TPRKB	4.62E-18	0.257032479	0.826	0.325	9.52E-14	14
LSM3	5.00E-18	0.44436922	0.971	0.521	1.03E-13	14
LAMTOR1	5.24E-18	0.409929667	0.971	0.497	1.08E-13	14
NUP62	5.99E-18	0.260605053	0.768	0.289	1.23E-13	14
CTDNEP1	6.43E-18	0.303496168	0.884	0.362	1.32E-13	14
SAC3D1	6.56E-18	0.324533473	0.522	0.161	1.35E-13	14
TNF	7.32E-18	0.384621976	0.522	0.136	1.51E-13	14
SUMO3	7.43E-18	0.361441751	0.928	0.441	1.53E-13	14
ARPC1B	8.98E-18	0.570572074	1	0.624	1.85E-13	14
GBP1	9.29E-18	0.552148582	0.783	0.299	1.91E-13	14
CDK4	1.00E-17	0.297261702	0.725	0.283	2.06E-13	14
RNASEH2C	1.10E-17	0.272091855	0.87	0.372	2.26E-13	14
POMP	1.13E-17	0.506010767	1	0.649	2.32E-13	14
WDR1	1.19E-17	0.426311989	0.899	0.408	2.46E-13	14
TMEM147	1.36E-17	0.257085928	0.841	0.342	2.81E-13	14
AGPAT2	1.49E-17	0.251133245	0.594	0.189	3.06E-13	14
MVP	1.55E-17	0.267365496	0.725	0.247	3.19E-13	14
TLN1	1.62E-17	0.366106579	0.899	0.395	3.34E-13	14
GNAQ	1.65E-17	0.252451497	0.739	0.268	3.40E-13	14
TPI1	1.71E-17	0.561454168	0.986	0.711	3.53E-13	14
VOPP1	1.81E-17	0.332634046	0.797	0.318	3.72E-13	14
SFT2D1	1.87E-17	0.261324182	0.797	0.293	3.85E-13	14
GNS	1.97E-17	0.369337301	0.812	0.333	4.06E-13	14
TPM3	2.07E-17	0.579187147	1	0.674	4.27E-13	14

SLC25A11	2.12E-17	0.288007064	0.71	0.276	4.37E-13	14
TMEM160	2.21E-17	0.309001887	0.87	0.389	4.55E-13	14
SRGN	2.53E-17	0.62206128	1	0.53	5.22E-13	14
TMX1	2.71E-17	0.290075025	0.681	0.248	5.58E-13	14
CALR	2.72E-17	0.608423653	1	0.758	5.61E-13	14
ATP6AP2	2.79E-17	0.529001641	0.928	0.498	5.74E-13	14
CCT5	2.90E-17	0.428761735	0.899	0.45	5.97E-13	14
ARF3	3.02E-17	0.270390881	0.783	0.313	6.23E-13	14
TENT5A	3.18E-17	0.293328036	0.754	0.281	6.54E-13	14
ARPC2	3.25E-17	0.622912337	1	0.759	6.70E-13	14
FAM50A	3.34E-17	0.265483465	0.841	0.337	6.88E-13	14
ATP6AP1	3.48E-17	0.304295199	0.928	0.415	7.17E-13	14
DNASE1L3	4.16E-17	0.581671077	0.42	0.102	8.57E-13	14
PGAM1	4.78E-17	0.458022731	0.957	0.557	9.84E-13	14
ZNF706	5.15E-17	0.49293364	0.942	0.507	1.06E-12	14
PSMD7	5.18E-17	0.404724625	0.899	0.447	1.07E-12	14
ARL8A	5.18E-17	0.284095312	0.725	0.27	1.07E-12	14
GPR183	5.20E-17	0.306622887	0.565	0.158	1.07E-12	14
PRKDC	5.59E-17	0.29905828	0.783	0.306	1.15E-12	14
RNASE1	5.64E-17	0.623040721	0.507	0.141	1.16E-12	14
HEXB	5.67E-17	0.396960418	0.855	0.378	1.17E-12	14
CD47	5.91E-17	0.288369437	0.87	0.368	1.22E-12	14
RAB5C	5.99E-17	0.401898315	0.971	0.507	1.23E-12	14
UBE2L6	6.00E-17	0.250017353	0.841	0.315	1.24E-12	14
GRK2	6.60E-17	0.300554915	0.826	0.344	1.36E-12	14
CETP	7.35E-17	0.365524304	0.29	0.054	1.51E-12	14
CALM2	7.67E-17	0.701248396	0.986	0.763	1.58E-12	14
TPP1	7.69E-17	0.437863366	0.971	0.507	1.58E-12	14
ILK	8.73E-17	0.27694842	0.855	0.37	1.80E-12	14
DERL1	8.84E-17	0.255182899	0.783	0.311	1.82E-12	14
AP1M1	9.34E-17	0.261325594	0.609	0.21	1.92E-12	14
PBDC1	9.58E-17	0.288888377	0.652	0.231	1.97E-12	14
PARP14	9.74E-17	0.328377921	0.812	0.332	2.01E-12	14
PSME2	9.77E-17	0.614898064	1	0.639	2.01E-12	14
SGK1	1.03E-16	0.275240505	0.768	0.258	2.13E-12	14
COX5A	1.12E-16	0.48628748	0.986	0.594	2.31E-12	14
PTTG1IP	1.20E-16	0.354117233	0.884	0.404	2.48E-12	14
HPRT1	1.28E-16	0.271064076	0.754	0.301	2.64E-12	14
NDUFB1	1.29E-16	0.347161729	0.942	0.458	2.66E-12	14
CAP1	1.30E-16	0.546087472	1	0.653	2.69E-12	14
PITHD1	1.35E-16	0.271550542	0.725	0.271	2.77E-12	14
AP3D1	1.47E-16	0.296896573	0.739	0.297	3.03E-12	14
SNX5	1.84E-16	0.402993197	0.899	0.447	3.79E-12	14
EGR1	1.88E-16	0.288681666	0.754	0.259	3.88E-12	14
FUCA1	1.97E-16	0.346297976	0.797	0.318	4.06E-12	14

HMGN3	2.01E-16	0.293647465	0.942	0.432	4.13E-12	14
MOB1A	2.03E-16	0.41208462	0.928	0.469	4.18E-12	14
GIMAP4	2.19E-16	0.324669806	0.826	0.284	4.51E-12	14
MSN	2.24E-16	0.384096048	0.971	0.411	4.61E-12	14
ATAD2	2.30E-16	0.337454023	0.565	0.189	4.74E-12	14
HSBP1	2.33E-16	0.438235768	0.928	0.53	4.79E-12	14
SDCBP	2.58E-16	0.495515886	0.957	0.522	5.31E-12	14
FADS1	2.75E-16	0.434253785	0.58	0.205	5.67E-12	14
MGST3	3.40E-16	0.525277919	0.957	0.552	7.01E-12	14
SMS	3.60E-16	0.343063481	0.812	0.364	7.41E-12	14
PPP1CA	3.62E-16	0.402990735	0.986	0.573	7.46E-12	14
RAB14	4.05E-16	0.256869038	0.87	0.384	8.34E-12	14
PA2G4	4.19E-16	0.435831613	0.899	0.467	8.63E-12	14
CLTA	4.42E-16	0.528032569	0.986	0.632	9.10E-12	14
RBPJ	4.44E-16	0.288435852	0.899	0.401	9.14E-12	14
SAT1	4.93E-16	0.624162547	1	0.727	1.02E-11	14
SH3BGRL	5.51E-16	0.324751981	0.899	0.327	1.13E-11	14
VPS35	5.82E-16	0.349534607	0.826	0.368	1.20E-11	14
LAMTOR2	5.89E-16	0.365592467	0.928	0.452	1.21E-11	14
LAP3	6.53E-16	0.410432354	0.783	0.345	1.34E-11	14
HNRNPR	7.32E-16	0.302652433	0.928	0.439	1.51E-11	14
ENO1	7.43E-16	0.562033028	1	0.73	1.53E-11	14
HCST	8.19E-16	0.516304991	0.986	0.415	1.69E-11	14
VASP	9.31E-16	0.339730064	0.841	0.348	1.92E-11	14
ABCA1	9.43E-16	0.372246535	0.826	0.349	1.94E-11	14
CSTB	9.73E-16	0.494425437	0.986	0.636	2.00E-11	14
DNAJC8	1.00E-15	0.304053856	0.942	0.471	2.06E-11	14
CD53	1.02E-15	0.442835724	0.957	0.401	2.11E-11	14
ERP44	1.09E-15	0.274999702	0.884	0.401	2.24E-11	14
PSMB6	1.36E-15	0.441464408	0.913	0.485	2.81E-11	14
PPIF	1.39E-15	0.293318515	0.739	0.302	2.86E-11	14
PFKL	1.42E-15	0.267611917	0.884	0.404	2.93E-11	14
GLUL	1.64E-15	0.562640876	0.971	0.526	3.37E-11	14
CNIH4	1.74E-15	0.319246056	0.754	0.317	3.59E-11	14
MT1E	1.79E-15	0.504110785	0.551	0.18	3.69E-11	14
ACTR3	1.83E-15	0.474363029	0.957	0.552	3.77E-11	14
H2AFJ	1.97E-15	0.336966921	0.855	0.385	4.06E-11	14
GTF2A2	2.13E-15	0.378176792	0.797	0.38	4.39E-11	14
TMSB10	2.74E-15	0.573277924	1	0.9	5.63E-11	14
SELENOT	2.78E-15	0.298161998	0.942	0.454	5.73E-11	14
PSMD2	2.80E-15	0.259361959	0.826	0.356	5.76E-11	14
FNBP1	2.91E-15	0.252335503	0.87	0.359	6.00E-11	14
EMP3	3.63E-15	0.433055577	0.928	0.406	7.47E-11	14
LRRC58	3.82E-15	0.26757316	0.696	0.277	7.87E-11	14
WASF2	5.21E-15	0.35704374	0.913	0.443	1.07E-10	14

RAP2B	6.35E-15	0.288969647	0.783	0.331	1.31E-10	14
G3BP1	6.91E-15	0.260140993	0.884	0.389	1.42E-10	14
PPP2CA	7.51E-15	0.254928969	0.812	0.35	1.55E-10	14
XPO1	7.73E-15	0.271311898	0.797	0.346	1.59E-10	14
HP1BP3	8.83E-15	0.411774754	0.855	0.449	1.82E-10	14
NENF	9.41E-15	0.299257282	0.855	0.398	1.94E-10	14
RAC2	1.01E-14	0.313163897	0.942	0.394	2.08E-10	14
ARPC4	1.06E-14	0.390181214	0.942	0.529	2.19E-10	14
SFT2D2	1.20E-14	0.256035547	0.71	0.288	2.47E-10	14
PGK1	1.23E-14	0.473066219	0.971	0.611	2.53E-10	14
RAB8A	1.25E-14	0.254029327	0.783	0.341	2.58E-10	14
KIF5B	1.30E-14	0.298682339	0.942	0.473	2.69E-10	14
ILF3	1.44E-14	0.259804576	0.797	0.351	2.96E-10	14
CISD2	1.45E-14	0.251322774	0.754	0.322	2.99E-10	14
GDI2	1.81E-14	0.381721527	0.957	0.534	3.74E-10	14
RAB10	2.14E-14	0.292707637	0.884	0.427	4.41E-10	14
REX1BD	2.52E-14	0.26456771	0.913	0.421	5.20E-10	14
NAPA	2.89E-14	0.260848661	0.783	0.337	5.94E-10	14
LSM5	3.13E-14	0.332637309	0.899	0.459	6.45E-10	14
CLEC10A	3.14E-14	0.351494304	0.406	0.109	6.47E-10	14
RBMX	3.23E-14	0.275505568	0.884	0.446	6.65E-10	14
GUSB	3.80E-14	0.312568811	0.913	0.432	7.82E-10	14
TCP1	3.84E-14	0.296322056	0.884	0.433	7.90E-10	14
DCAF7	4.17E-14	0.345253392	0.768	0.357	8.59E-10	14
MLEC	4.36E-14	0.312365781	0.928	0.432	8.99E-10	14
TXNDC17	4.57E-14	0.345028288	0.87	0.449	9.42E-10	14
HNRNPD	4.68E-14	0.392539652	0.928	0.533	9.64E-10	14
CHD4	4.73E-14	0.2919466	0.855	0.399	9.74E-10	14
CD5L	5.16E-14	0.8073807	0.275	0.058	1.06E-09	14
GNAI3	5.25E-14	0.287388971	0.826	0.397	1.08E-09	14
SLBP	5.35E-14	0.394005701	0.638	0.269	1.10E-09	14
SDS	5.78E-14	0.267529766	0.29	0.063	1.19E-09	14
SERF2	5.87E-14	0.368622473	1	0.912	1.21E-09	14
ENY2	6.34E-14	0.380838385	0.957	0.558	1.31E-09	14
ANP32A	6.80E-14	0.32169272	0.899	0.481	1.40E-09	14
RBX1	6.97E-14	0.380855352	0.957	0.57	1.44E-09	14
ANAPC11	8.40E-14	0.336774709	0.971	0.549	1.73E-09	14
MBD2	1.11E-13	0.303802881	0.855	0.433	2.28E-09	14
SNX3	1.43E-13	0.349448481	0.971	0.579	2.94E-09	14
FTL	1.54E-13	0.447873072	1	0.998	3.17E-09	14
ANP32E	1.55E-13	0.362784272	0.797	0.396	3.19E-09	14
LRP1	1.58E-13	0.273489889	0.739	0.328	3.25E-09	14
A2M	2.10E-13	0.290265646	0.725	0.288	4.33E-09	14
ATP1B1	2.11E-13	0.324950862	0.768	0.346	4.35E-09	14
RAB7A	2.12E-13	0.37201248	0.957	0.543	4.36E-09	14

PSMA7	2.15E-13	0.443290077	1	0.721	4.43E-09	14
MT1F	2.16E-13	0.397413363	0.406	0.117	4.45E-09	14
NDUFS7	2.28E-13	0.309410446	0.855	0.43	4.70E-09	14
CXCL10	2.39E-13	0.453692289	0.29	0.065	4.93E-09	14
SMC3	2.55E-13	0.28963957	0.768	0.348	5.25E-09	14
CKLF	2.77E-13	0.357570382	0.913	0.515	5.71E-09	14
GGH	3.05E-13	0.26475421	0.681	0.277	6.29E-09	14
UBE2L3	3.56E-13	0.338245882	0.913	0.526	7.33E-09	14
ARF1	3.56E-13	0.35276782	0.986	0.601	7.34E-09	14
ERH	3.64E-13	0.329048938	0.942	0.533	7.51E-09	14
SLIRP	3.76E-13	0.296559402	0.812	0.393	7.73E-09	14
SYNCRIP	4.06E-13	0.278103326	0.884	0.428	8.36E-09	14
SRSF3	4.81E-13	0.353620257	1	0.651	9.91E-09	14
LRPAP1	5.54E-13	0.259480396	0.855	0.427	1.14E-08	14
CORO1A	5.55E-13	0.295242393	0.913	0.376	1.14E-08	14
XRCC6	5.57E-13	0.321485593	0.942	0.512	1.15E-08	14
ITPR2	6.54E-13	0.282549877	0.71	0.302	1.35E-08	14
LAMP1	7.17E-13	0.424945826	0.928	0.536	1.48E-08	14
NDFIP1	8.84E-13	0.266442744	0.87	0.438	1.82E-08	14
ELOC	1.04E-12	0.300355531	0.87	0.458	2.15E-08	14
NFIC	1.13E-12	0.29107168	0.696	0.314	2.32E-08	14
MRPL51	1.13E-12	0.282944364	0.928	0.475	2.34E-08	14
H3F3A	1.28E-12	0.426815955	1	0.893	2.63E-08	14
ERP29	1.34E-12	0.354061374	0.957	0.62	2.76E-08	14
NDUFA2	1.35E-12	0.26963029	0.957	0.501	2.78E-08	14
SH3BGRL3	1.37E-12	0.508824621	1	0.751	2.82E-08	14
TMBIM6	1.48E-12	0.419032278	1	0.758	3.04E-08	14
CEBPA	1.56E-12	0.250677249	0.725	0.324	3.21E-08	14
TMBIM4	1.61E-12	0.371663002	0.942	0.547	3.32E-08	14
EIF5A	1.83E-12	0.309518383	0.928	0.531	3.76E-08	14
RALY	2.35E-12	0.298233116	0.826	0.418	4.85E-08	14
YWHAE	2.63E-12	0.32228277	0.942	0.534	5.41E-08	14
CDC42	2.73E-12	0.316138699	1	0.61	5.62E-08	14
HNRNPM	3.16E-12	0.270037052	0.942	0.508	6.52E-08	14
SIVA1	3.34E-12	0.330933155	0.884	0.459	6.87E-08	14
ATP5PO	3.78E-12	0.351195965	0.928	0.601	7.78E-08	14
PSMB3	3.98E-12	0.389742408	0.942	0.574	8.19E-08	14
COX7B	4.12E-12	0.359430673	0.971	0.644	8.48E-08	14
PRDX4	4.34E-12	0.256513842	0.797	0.382	8.95E-08	14
IER5	4.47E-12	0.295874983	0.928	0.471	9.20E-08	14
CTSA	4.61E-12	0.345826342	0.957	0.515	9.50E-08	14
S100A6	4.85E-12	0.297454848	0.957	0.453	9.99E-08	14
GNB1	4.96E-12	0.302726299	0.928	0.519	1.02E-07	14
SCAF11	4.97E-12	0.297809549	0.913	0.491	1.02E-07	14
MT1G	6.03E-12	0.886329886	0.391	0.123	1.24E-07	14

FKBP2	6.43E-12	0.299136641	0.87	0.461	1.33E-07	14
COPE	6.98E-12	0.342886252	0.957	0.583	1.44E-07	14
PSMB8	8.12E-12	0.250840524	0.913	0.477	1.67E-07	14
SRRM1	8.47E-12	0.28499122	0.884	0.497	1.74E-07	14
MAT2A	8.53E-12	0.280764575	0.826	0.396	1.76E-07	14
GALM	8.79E-12	0.252791828	0.725	0.34	1.81E-07	14
PSMB2	8.82E-12	0.310033016	0.928	0.493	1.82E-07	14
CENPX	8.86E-12	0.269399918	0.768	0.366	1.82E-07	14
ATOX1	9.67E-12	0.31318876	0.957	0.519	1.99E-07	14
HNRNPF	1.08E-11	0.327803111	0.957	0.569	2.23E-07	14
GMFG	1.14E-11	0.327885851	0.957	0.431	2.35E-07	14
SNRPD1	1.36E-11	0.30161512	0.841	0.465	2.80E-07	14
DCTN3	1.78E-11	0.297756685	0.783	0.417	3.67E-07	14
ATP6V0E1	1.81E-11	0.374996368	0.971	0.645	3.72E-07	14
RANBP1	1.99E-11	0.353225126	0.913	0.536	4.10E-07	14
PPP1R14B	2.29E-11	0.278802915	0.884	0.465	4.71E-07	14
RHOB	2.51E-11	0.486689431	0.957	0.593	5.18E-07	14
TMED10	3.30E-11	0.260620708	0.913	0.489	6.80E-07	14
S100A4	3.80E-11	0.257393618	0.957	0.454	7.82E-07	14
FKBP1A	5.49E-11	0.265855594	0.986	0.637	1.13E-06	14
NUCB1	5.53E-11	0.28018762	0.754	0.4	1.14E-06	14
PSMC4	5.56E-11	0.274041443	0.725	0.356	1.14E-06	14
GNAS	5.57E-11	0.374233325	1	0.762	1.15E-06	14
PSMA3	6.63E-11	0.265932843	0.884	0.48	1.37E-06	14
HES1	6.75E-11	0.681619036	0.638	0.301	1.39E-06	14
NDUFS6	7.54E-11	0.269183619	0.957	0.569	1.55E-06	14
LAMP2	9.02E-11	0.255126691	0.884	0.457	1.86E-06	14
SNX6	9.42E-11	0.307809146	0.884	0.477	1.94E-06	14
NAP1L1	9.50E-11	0.358319106	0.971	0.65	1.96E-06	14
PRDX3	1.02E-10	0.253270372	0.957	0.504	2.09E-06	14
CD164	1.09E-10	0.329933967	0.942	0.542	2.24E-06	14
PRDX1	1.10E-10	0.532187337	1	0.763	2.27E-06	14
BANF1	1.34E-10	0.296106039	0.87	0.509	2.76E-06	14
SNRPG	1.39E-10	0.312890948	0.957	0.594	2.86E-06	14
MANF	1.73E-10	0.283860878	0.812	0.414	3.56E-06	14
UQCRQ	1.75E-10	0.294901182	1	0.625	3.60E-06	14
GNG5	1.92E-10	0.316581692	1	0.69	3.96E-06	14
SRSF2	2.01E-10	0.343922063	0.928	0.59	4.14E-06	14
ATP5PB	2.11E-10	0.312029127	0.957	0.569	4.34E-06	14
SNRPD3	2.22E-10	0.250716864	0.971	0.546	4.57E-06	14
GAPDH	2.88E-10	0.336730791	1	0.919	5.93E-06	14
STAT1	2.93E-10	0.387419621	0.783	0.42	6.03E-06	14
CALM1	3.03E-10	0.345501974	0.986	0.738	6.24E-06	14
HNRNPA2B1	3.70E-10	0.321597218	1	0.813	7.63E-06	14
MYDGF	4.33E-10	0.253514826	0.87	0.459	8.92E-06	14

NINJ1	4.50E-10	0.271298898	0.783	0.404	9.26E-06	14
NDUFB2	5.07E-10	0.295572285	0.971	0.659	1.04E-05	14
COX7A2	6.38E-10	0.29382687	0.971	0.697	1.31E-05	14
VDAC1	7.43E-10	0.266919165	0.899	0.534	1.53E-05	14
SFPQ	7.79E-10	0.279360672	0.884	0.547	1.60E-05	14
PSMB1	8.60E-10	0.291994502	0.942	0.603	1.77E-05	14
MDH1	8.66E-10	0.262941335	0.899	0.517	1.78E-05	14
GNB2	9.31E-10	0.288042894	0.957	0.578	1.92E-05	14
ZFP36L1	9.52E-10	0.361690879	1	0.651	1.96E-05	14
MT2A	1.10E-09	0.885849352	0.928	0.586	2.27E-05	14
EIF3K	1.16E-09	0.319351033	0.986	0.665	2.38E-05	14
ATP6V1F	1.70E-09	0.264568708	0.942	0.569	3.51E-05	14
TALDO1	2.58E-09	0.372154745	0.986	0.587	5.30E-05	14
DAZAP2	2.87E-09	0.256268511	0.899	0.532	5.92E-05	14
ACTG1	3.77E-09	0.343745648	1	0.912	7.76E-05	14
YWHAB	4.59E-09	0.327346739	0.986	0.709	9.45E-05	14
PCBP1	4.87E-09	0.297209253	1	0.673	0.000100251	14
COX6C	6.35E-09	0.293791189	0.986	0.719	0.000130767	14
PCBP2	9.54E-09	0.254730271	0.986	0.629	0.000196447	14
RPN2	1.13E-08	0.265048275	0.913	0.561	0.00023277	14
IQGAP2	1.16E-08	0.277743714	0.913	0.467	0.00023821	14
COX4I1	1.17E-08	0.357619672	0.971	0.778	0.000240964	14
ATP5MG	1.55E-08	0.271218317	1	0.774	0.000318659	14
SET	1.71E-08	0.263537105	0.957	0.661	0.000351988	14
IER3	2.11E-08	0.26527548	0.768	0.439	0.000433821	14
PTMS	2.25E-08	0.377631077	0.928	0.559	0.000463647	14
ITM2B	3.12E-08	0.365039034	1	0.873	0.000642727	14
ATP5F1B	4.97E-08	0.269541452	0.986	0.639	0.001023853	14
UQCR10	5.81E-08	0.288385251	0.971	0.676	0.001196399	14
APLP2	6.15E-08	0.262788994	0.986	0.572	0.001267266	14
RTN4	7.80E-08	0.270208925	0.971	0.621	0.001607239	14
HLA-B	9.16E-08	0.400045804	1	0.662	0.001886365	14
MT1X	9.75E-08	0.543193031	0.652	0.348	0.002008838	14
CHCHD2	1.37E-07	0.266882065	0.986	0.803	0.002828721	14
SELENOP	5.63E-07	0.553071296	0.913	0.66	0.011587874	14
B2M	1.41E-06	0.372123025	1	0.947	0.028992154	14
HSP90B1	1.42E-06	0.25652151	0.986	0.681	0.029190746	14
SPP1	0.000359196	0.493916019	0.391	0.215	1	14
IL1RN	0.006947775	0.28912712	0.348	0.23	1	14
CMTM2	0	2.924670015	0.643	0.003	0	15
FCGR3B	0	2.822519827	0.738	0.004	0	15
CXCR2	3.25E-187	2.096318028	0.5	0.009	6.70E-183	15
S100A8	1.15E-97	3.913287403	0.762	0.049	2.38E-93	15
CXCL8	1.52E-73	4.435002443	0.881	0.1	3.14E-69	15
G0S2	1.56E-73	3.923491386	0.881	0.099	3.22E-69	15

S100A9	7.66E-51	3.304551685	0.952	0.181	1.58E-46	15
PHACTR1	1.58E-33	2.488349728	0.69	0.133	3.25E-29	15
S100A12	2.77E-33	3.285678412	0.286	0.02	5.71E-29	15
ALOX5AP	1.74E-31	2.469802379	0.667	0.131	3.58E-27	15
IFITM2	3.89E-27	2.71710303	0.929	0.458	8.01E-23	15
MNDA	6.92E-27	2.546276742	0.667	0.156	1.43E-22	15
SRGN	8.05E-25	2.086503297	0.952	0.533	1.66E-20	15
NAMPT	3.52E-24	2.90540141	0.905	0.505	7.26E-20	15
FPR1	3.40E-23	1.983664353	0.5	0.092	7.01E-19	15
FOS	8.27E-23	2.217114521	0.929	0.486	1.70E-18	15
H3F3A	2.73E-22	1.785575457	0.976	0.893	5.62E-18	15
H3F3B	4.90E-21	1.623929271	0.976	0.882	1.01E-16	15
BCL2A1	8.42E-21	2.248685134	0.619	0.166	1.73E-16	15
NEAT1	1.90E-19	1.620830574	0.976	0.782	3.91E-15	15
DUSP1	5.76E-18	1.566711554	0.952	0.637	1.19E-13	15
S100A11	1.04E-17	1.389673255	0.905	0.494	2.15E-13	15
HCAR3	2.86E-16	1.455252287	0.262	0.034	5.89E-12	15
VNN2	4.16E-16	1.704187076	0.333	0.056	8.57E-12	15
CXCR4	8.32E-16	1.999299536	0.738	0.335	1.71E-11	15
FTH1	1.01E-15	1.078780404	1	0.981	2.09E-11	15
SOD2	4.29E-14	1.756408879	0.81	0.567	8.83E-10	15
SELL	1.67E-13	1.692321541	0.381	0.087	3.43E-09	15
B2M	2.75E-13	0.850675184	1	0.947	5.66E-09	15
LITAF	9.18E-12	1.873831929	0.738	0.512	1.89E-07	15
DNAJB1	1.30E-11	1.33602982	0.857	0.626	2.67E-07	15
RGS2	1.73E-11	1.554599451	0.69	0.36	3.56E-07	15
TMEM154	3.15E-11	1.325152927	0.262	0.049	6.48E-07	15
SAT1	1.11E-10	1.171174603	0.905	0.729	2.28E-06	15
ITM2B	1.27E-10	1.010179502	0.857	0.875	2.61E-06	15
NFKBIA	1.35E-10	1.161538454	0.833	0.618	2.79E-06	15
JUNB	3.90E-10	1.100793995	0.81	0.553	8.03E-06	15
FCER1G	5.75E-10	0.934576878	0.762	0.334	1.19E-05	15
HSPA1A	1.17E-09	1.294536699	0.857	0.867	2.41E-05	15
QPCT	1.61E-09	1.386312659	0.262	0.057	3.32E-05	15
CDC42EP3	1.64E-09	1.879402939	0.405	0.135	3.38E-05	15
SMIM25	2.27E-09	1.57820469	0.333	0.092	4.67E-05	15
HSP90AA1	8.49E-09	0.845847021	0.905	0.892	0.000174757	15
BASP1	1.15E-07	1.910543571	0.429	0.181	0.00237453	15
LST1	1.64E-07	1.410289289	0.5	0.255	0.003383564	15
MT-ND2	1.84E-07	0.827944997	1	0.998	0.003780971	15
MT-ND3	3.45E-07	0.585179979	1	0.999	0.007098221	15
EIF1	5.40E-07	0.700584084	0.929	0.915	0.011113536	15
HSPA1B	6.77E-07	1.002458483	0.762	0.633	0.013947113	15
MALAT1	8.43E-07	0.474967251	1	0.953	0.017363385	15
PTGS2	1.31E-06	2.275199141	0.262	0.079	0.02696466	15

MCL1	4.24E-06	1.442490323	0.643	0.62	0.087238948	15
GCA	5.65E-06	1.838697389	0.476	0.285	0.116407002	15
SLC25A37	5.90E-06	1.999163547	0.429	0.227	0.121540505	15
ZFP36	9.10E-06	1.78843281	0.548	0.396	0.187431067	15
GLUL	1.00E-05	1.364967021	0.619	0.531	0.206625281	15
MXD1	1.71E-05	1.683378373	0.405	0.212	0.352375654	15
LCP1	3.92E-05	1.035282146	0.548	0.421	0.806927491	15
UBE2D3	4.73E-05	1.467990383	0.619	0.626	0.973910526	15
BTG1	4.93E-05	0.835036161	0.738	0.762	1	15
MAP1LC3B	7.66E-05	1.590420125	0.571	0.527	1	15
TNFAIP3	0.000112648	1.099813683	0.5	0.328	1	15
NCF2	0.000117662	1.454016334	0.357	0.186	1	15
TXNIP	0.000118101	1.196092907	0.643	0.643	1	15
CD53	0.000121239	0.946548749	0.524	0.407	1	15
PLEK	0.000151909	1.193031832	0.429	0.261	1	15
DDX3X	0.000196677	1.773592285	0.548	0.491	1	15
AC023157.3	0.00020608	1.399423093	0.262	0.109	1	15
TRIB1	0.000235935	1.842207022	0.381	0.224	1	15
PNRC1	0.000261688	0.917083885	0.667	0.733	1	15
RSF1	0.000387768	0.255669373	0.095	0.417	1	15
NFKBIZ	0.000428795	1.674192595	0.476	0.371	1	15
MT-CYB	0.00047721	0.442868508	1	1	1	15
SLC2A3	0.000500211	1.429937858	0.429	0.277	1	15
GABARAPL2	0.000541109	0.294529451	0.143	0.502	1	15
DNAJA1	0.000593198	0.977827917	0.643	0.705	1	15
PAK2	0.000649529	0.270291986	0.119	0.45	1	15
PPP2CA	0.000654028	0.250815298	0.071	0.358	1	15
ZFP36L1	0.000655335	0.959604199	0.619	0.655	1	15
DNAJB6	0.000754344	1.416160279	0.548	0.548	1	15
EVI2B	0.000761212	1.223909707	0.381	0.241	1	15
CHURC1	0.001182485	0.41528451	0.119	0.432	1	15
TNFRSF14	0.001186469	0.251922756	0.119	0.428	1	15
POGZ	0.001533576	0.273076765	0.071	0.331	1	15
FAM107B	0.00174932	0.278388758	0.095	0.372	1	15
MT-ND1	0.001998457	0.33640592	1	0.999	1	15
NBR1	0.002048449	0.399813838	0.071	0.323	1	15
BUD31	0.002098284	0.28741642	0.119	0.412	1	15
USP16	0.002338088	0.250711877	0.071	0.319	1	15
VPS28	0.002404678	0.279660289	0.19	0.55	1	15
FAM133B	0.002427109	0.289218386	0.143	0.448	1	15
KARS	0.002428808	0.25683947	0.071	0.317	1	15
RBMS1	0.002472138	0.34910071	0.143	0.452	1	15
UBE2D1	0.002495066	1.356323602	0.381	0.271	1	15
AMD1	0.002548266	0.425566194	0.119	0.406	1	15
CELF1	0.002588374	0.28643119	0.119	0.405	1	15

RALBP1	0.002619143	0.297307663	0.119	0.403	1	15
MKNK2	0.002728552	0.25136375	0.095	0.354	1	15
PDLIM5	0.002796657	0.274286068	0.048	0.268	1	15
USP8	0.002823706	0.289504823	0.095	0.357	1	15
CHMP1B	0.002876076	0.343048734	0.119	0.401	1	15
IER2	0.002905608	1.042081758	0.548	0.502	1	15
TYROBP	0.002924406	0.507768332	0.548	0.329	1	15
ZNF24	0.003036936	0.258554971	0.071	0.31	1	15
HEBP2	0.003111837	0.366825939	0.095	0.354	1	15
KPNB1	0.003338186	0.311664824	0.167	0.491	1	15
ASH1L	0.003566522	0.285452728	0.119	0.393	1	15
PHF20	0.003651325	0.332316004	0.095	0.349	1	15
GOLGA7	0.003715547	0.277707248	0.071	0.304	1	15
EGR1	0.003747994	1.192236416	0.381	0.264	1	15
RIOK3	0.003758281	0.486253935	0.095	0.348	1	15
FNDC3B	0.003829165	0.362993624	0.119	0.392	1	15
HNRNPH3	0.003920104	0.35392709	0.143	0.438	1	15
FAM120A	0.003983541	0.577720089	0.143	0.435	1	15
RNF149	0.004024112	1.40163462	0.452	0.404	1	15
IFI6	0.004200577	0.296376776	0.071	0.299	1	15
LYN	0.004311367	1.352636846	0.357	0.251	1	15
CHMP3	0.004368302	0.272579729	0.071	0.298	1	15
NDUFB6	0.004528919	0.342726322	0.119	0.386	1	15
OGFRL1	0.004614322	1.225048955	0.286	0.167	1	15
ADAR	0.004786429	0.318157288	0.167	0.477	1	15
NDUFB3	0.00485542	0.373539163	0.19	0.519	1	15
MAP3K13	0.004941708	0.477293017	0.119	0.383	1	15
ZNF148	0.005009522	0.380141413	0.095	0.338	1	15
HLA-C	0.005475062	0.365954485	0.786	0.875	1	15
RAB21	0.005581118	0.286998959	0.095	0.334	1	15
N4BP2L2	0.0056831	0.456112233	0.214	0.562	1	15
UBL3	0.005690737	0.260248577	0.071	0.289	1	15
MT-ND4	0.005707927	0.269819589	1	1	1	15
6-Mar	0.005734705	0.305683054	0.095	0.333	1	15
SEC62	0.006202821	0.432909384	0.214	0.559	1	15
PSMB1	0.006217392	0.29675424	0.238	0.61	1	15
KPNA4	0.006326774	0.317100896	0.095	0.33	1	15
CSK	0.006335649	0.33846017	0.095	0.326	1	15
BLOC1S1	0.006385084	0.385251912	0.19	0.514	1	15
MORF4L2	0.006537353	0.40973741	0.143	0.419	1	15
HNRNPK	0.006951391	0.25378658	0.286	0.711	1	15
ATXN1	0.007066494	0.372663094	0.095	0.326	1	15
FAM53C	0.007202778	1.298311709	0.31	0.202	1	15
SH3GLB1	0.007253762	0.552745082	0.119	0.37	1	15
MBD4	0.007359956	0.284445988	0.071	0.281	1	15

GPBP1	0.007368616	0.320030681	0.143	0.414	1	15
ANKRD10	0.007380718	0.315316499	0.071	0.281	1	15
ISCU	0.007468698	0.418966635	0.167	0.461	1	15
CNIH4	0.007530471	0.333161924	0.095	0.324	1	15
MT-CO2	0.007655521	0.271684736	1	1	1	15
TXNDC12	0.007828095	0.264358471	0.095	0.322	1	15
CNN2	0.007922746	0.391420159	0.095	0.322	1	15
PUM1	0.007939226	0.314573829	0.119	0.365	1	15
PTPN12	0.008067551	0.334603799	0.095	0.321	1	15
PTTG1IP	0.008126342	0.531447516	0.143	0.412	1	15
NOSIP	0.008152882	0.304055138	0.095	0.321	1	15
NFIC	0.008164015	0.254168325	0.095	0.32	1	15
UBE2B	0.008231995	0.423375782	0.19	0.505	1	15
C5AR1	0.008436893	1.105838273	0.262	0.148	1	15
IER3	0.008440671	1.213712525	0.476	0.443	1	15
ARF3	0.008502212	0.346340407	0.095	0.32	1	15
COX7A2	0.008751805	0.278419621	0.286	0.703	1	15
SETX	0.008907859	0.330525329	0.095	0.318	1	15
TNFRSF1A	0.00894838	0.331908375	0.095	0.318	1	15
ABCA1	0.009129334	0.296257926	0.119	0.357	1	15
ANXA7	0.009340745	0.440081608	0.143	0.406	1	15
RAB7A	0.009413127	0.403998395	0.214	0.551	1	15
MLF2	0.009713406	0.315372711	0.143	0.402	1	15
ZNF655	0.00977078	0.347554677	0.119	0.359	1	15
C1orf122	0.009942123	0.357148413	0.095	0.314	1	15

Table S2. Subclusters-specific marker genes of hepatocytes.

gene	p_val	avg_logFC	pct.1	pct.2	p_val_adj	cluster
CP	2.57E-67	0.845130577	0.963	0.853	5.29E-63	0
HP	1.47E-57	1.159372252	0.938	0.819	3.02E-53	0
IGFBP1	3.64E-53	0.576057134	0.984	0.929	7.51E-49	0
C3	1.58E-42	0.652968082	0.92	0.823	3.26E-38	0
APCS	8.98E-42	0.87930285	0.722	0.459	1.85E-37	0
C1S	1.96E-39	0.658943561	0.823	0.675	4.04E-35	0
LBP	1.35E-37	0.911374454	0.794	0.597	2.77E-33	0
F10	4.85E-33	0.477850688	0.84	0.743	9.98E-29	0
IL32	2.08E-30	0.651931963	0.506	0.272	4.29E-26	0
APOE	1.23E-29	0.333372233	0.998	0.988	2.54E-25	0
TMEM176B	4.17E-28	0.379699524	0.916	0.853	8.59E-24	0
LEAP2	1.79E-26	0.463688704	0.728	0.599	3.68E-22	0
ITIH1	4.80E-25	0.34204654	0.942	0.841	9.89E-21	0
SOD2	6.96E-25	0.505008228	0.805	0.677	1.43E-20	0
IGFBP2	1.55E-24	0.492059649	0.862	0.691	3.20E-20	0
GBP2	2.88E-24	0.280131973	0.611	0.4	5.93E-20	0
IFITM3	2.38E-23	0.45451162	0.895	0.829	4.90E-19	0
AZGP1	3.79E-23	0.415401965	0.901	0.842	7.80E-19	0
CFB	2.75E-21	0.453905873	0.695	0.54	5.66E-17	0
MST1	3.08E-21	0.36821657	0.798	0.684	6.34E-17	0
RBP4	1.89E-20	0.312282549	0.996	0.976	3.88E-16	0
HLA-A	2.53E-20	0.35329177	0.922	0.84	5.22E-16	0
ALDH1L1	2.91E-20	0.425559632	0.757	0.63	5.99E-16	0
CYP27A1	3.28E-20	0.349478342	0.778	0.687	6.76E-16	0
PROS1	5.32E-20	0.350415743	0.718	0.556	1.10E-15	0
CTSD	6.10E-20	0.344162084	0.858	0.753	1.26E-15	0
TAPBP	1.50E-19	0.346276658	0.671	0.526	3.08E-15	0
CD63	1.85E-19	0.377785198	0.805	0.733	3.80E-15	0
CES1	5.14E-19	0.456094206	0.924	0.843	1.06E-14	0
TMEM176A	5.82E-19	0.323848219	0.912	0.878	1.20E-14	0
HLA-C	1.06E-18	0.380206714	0.881	0.788	2.19E-14	0
TMEM123	1.42E-18	0.308564515	0.794	0.685	2.93E-14	0
C1R	3.10E-18	0.468204718	0.566	0.401	6.38E-14	0
RHOB	3.25E-18	0.31844962	0.747	0.652	6.70E-14	0
C4BPA	3.30E-17	0.366674778	0.601	0.436	6.80E-13	0
TYMP	2.28E-16	0.326889849	0.527	0.368	4.70E-12	0
CEBPD	4.01E-16	0.305514889	0.749	0.665	8.25E-12	0
HEXB	4.08E-16	0.284906995	0.591	0.441	8.41E-12	0
CFH	5.37E-16	0.391270588	0.716	0.578	1.11E-11	0
MASP2	2.53E-15	0.324703422	0.733	0.645	5.20E-11	0
SERPING1	5.59E-15	0.328751256	0.792	0.696	1.15E-10	0
CYP2D6	6.51E-15	0.335563504	0.677	0.567	1.34E-10	0

HSD11B1	1.19E-14	0.356254541	0.27	0.128	2.46E-10	0
MGAT4B	1.64E-14	0.266286938	0.712	0.612	3.38E-10	0
TFPI	1.93E-14	0.317173543	0.837	0.763	3.98E-10	0
ORM1	2.13E-14	0.602212784	0.887	0.827	4.38E-10	0
RARRES2	2.41E-14	0.300516184	0.93	0.883	4.97E-10	0
HPR	3.49E-14	0.383892676	0.586	0.459	7.19E-10	0
ITIH3	6.94E-14	0.34090465	0.811	0.715	1.43E-09	0
FN1	7.84E-14	0.424006615	0.914	0.814	1.61E-09	0
F2	4.01E-13	0.301687991	0.866	0.779	8.27E-09	0
LRG1	7.93E-13	0.341249861	0.496	0.351	1.63E-08	0
ABCC3	9.15E-13	0.288995634	0.593	0.451	1.88E-08	0
LAMP2	1.73E-12	0.280496317	0.693	0.581	3.57E-08	0
A2M	1.78E-12	0.303494184	0.37	0.229	3.66E-08	0
ITIH4	4.61E-12	0.273040115	0.516	0.372	9.49E-08	0
ITGAV	4.86E-12	0.281880229	0.42	0.281	1.00E-07	0
UGT2B4	5.07E-12	0.355266253	0.722	0.632	1.04E-07	0
LMAN2	5.55E-12	0.267976984	0.728	0.612	1.14E-07	0
CYP4F3	7.61E-12	0.286157946	0.549	0.4	1.57E-07	0
PCOLCE	7.90E-12	0.260714014	0.644	0.541	1.63E-07	0
MT-ND2	9.66E-12	0.285921125	1	0.998	1.99E-07	0
RHOC	2.78E-11	0.297062108	0.586	0.477	5.73E-07	0
POR	2.85E-11	0.250383162	0.784	0.695	5.88E-07	0
CLU	3.72E-11	0.280957059	0.864	0.775	7.67E-07	0
NEAT1	6.14E-11	0.362196479	0.726	0.672	1.26E-06	0
NFKBIA	1.40E-10	0.291811071	0.648	0.531	2.89E-06	0
SAA4	1.65E-10	0.324854083	0.749	0.684	3.40E-06	0
UBD	1.67E-10	0.431364874	0.426	0.304	3.44E-06	0
HPX	3.83E-10	0.301486041	0.418	0.294	7.89E-06	0
SQSTM1	6.30E-10	0.330095416	0.798	0.735	1.30E-05	0
ITGB1	8.86E-10	0.260030969	0.776	0.671	1.83E-05	0
ZBTB20	2.08E-09	0.275865102	0.669	0.578	4.28E-05	0
PCK1	3.59E-09	0.466784098	0.681	0.584	7.40E-05	0
PALLD	5.59E-09	0.254178366	0.473	0.355	0.000115182	0
SELENOM	1.02E-08	0.270631864	0.572	0.478	0.00021011	0
ADH1B	1.43E-08	0.516741901	0.391	0.289	0.000294114	0
GRN	2.80E-08	0.264164652	0.761	0.677	0.000576162	0
MT-ND4L	3.90E-08	0.312529557	0.864	0.777	0.000803416	0
APOB	6.02E-08	0.339617156	0.984	0.932	0.001239634	0
CD151	1.22E-07	0.296102802	0.337	0.245	0.002506017	0
KRT18	1.44E-07	0.261475198	0.782	0.752	0.002969835	0
XIST	1.50E-07	0.33735242	0.675	0.598	0.003089141	0
CCL20	2.33E-07	0.637612146	0.741	0.663	0.004801466	0
ORM2	2.73E-07	0.372051687	0.819	0.766	0.005619695	0
CYP2E1	2.86E-07	0.258373756	0.862	0.869	0.005897428	0
TM4SF1	3.14E-07	0.266869381	0.267	0.176	0.006456537	0

CYP2C8	2.33E-06	0.274138389	0.28	0.184	0.047915559	0
FGB	3.61E-06	0.269705826	0.85	0.793	0.074318118	0
SPP1	4.80E-05	0.445211203	0.27	0.192	0.987874304	0
FGA	0.000675012	0.274047067	0.792	0.719	1	0
HSPA5	0.000807009	0.25883352	0.811	0.713	1	0
FTL	7.34E-76	0.774452878	1	1	1.51E-71	1
RPL32	1.06E-70	0.704624815	0.987	0.998	2.18E-66	1
RPS18	1.21E-65	0.607203115	0.998	0.999	2.50E-61	1
RPS3A	3.89E-65	0.737076692	0.945	0.972	8.00E-61	1
RPL29	1.38E-64	0.632342529	0.962	0.988	2.84E-60	1
RPS24	1.30E-63	0.688971464	0.942	0.985	2.68E-59	1
EEF1A1	5.45E-62	0.533098072	0.993	0.999	1.12E-57	1
RPL18A	4.13E-61	0.672456189	0.927	0.974	8.51E-57	1
RPL39	1.50E-60	0.651543724	0.982	0.993	3.09E-56	1
RPS12	2.86E-59	0.713790744	0.945	0.981	5.89E-55	1
RPL11	1.28E-58	0.553805905	0.96	0.987	2.64E-54	1
RPS6	9.42E-57	0.649532194	0.978	0.99	1.94E-52	1
RPL13	4.05E-56	0.460295818	0.993	0.997	8.33E-52	1
RPS7	8.19E-56	0.556954127	0.985	0.995	1.69E-51	1
RPL9	1.39E-54	0.591009801	0.914	0.965	2.86E-50	1
RPL14	2.99E-54	0.547739391	0.976	0.99	6.15E-50	1
RPLP1	3.67E-54	0.561661588	0.987	0.99	7.56E-50	1
RPS27	2.24E-53	0.57016124	0.989	0.997	4.61E-49	1
RPL12	3.06E-53	0.724856699	0.9	0.963	6.31E-49	1
RPL26	4.03E-53	0.691563663	0.925	0.963	8.30E-49	1
RPL35	1.74E-52	0.582783309	0.896	0.963	3.58E-48	1
RPL13A	5.19E-52	0.494931893	0.936	0.981	1.07E-47	1
RPL37A	7.63E-52	0.520242902	0.973	0.989	1.57E-47	1
RPS14	1.10E-50	0.567102198	0.956	0.98	2.27E-46	1
RPL22	2.10E-50	0.666407667	0.9	0.964	4.32E-46	1
RPLP2	4.40E-50	0.524504979	0.942	0.978	9.06E-46	1
RPS15A	4.49E-50	0.617016194	0.942	0.977	9.25E-46	1
RPS23	1.32E-49	0.593331958	0.958	0.981	2.72E-45	1
RPL10	3.83E-49	0.528712408	0.978	0.995	7.88E-45	1
RPL30	8.15E-49	0.628383835	0.947	0.983	1.68E-44	1
RPL10A	9.49E-47	0.603780115	0.927	0.971	1.95E-42	1
RPL24	2.38E-46	0.537966177	0.94	0.983	4.91E-42	1
RPL5	3.02E-46	0.539188231	0.96	0.985	6.22E-42	1
RPS27A	9.42E-46	0.496042973	0.982	0.992	1.94E-41	1
RPL35A	1.30E-45	0.562721355	0.934	0.978	2.67E-41	1
RPL37	2.39E-45	0.557170116	0.967	0.984	4.92E-41	1
RPL21	4.56E-45	0.644533763	0.923	0.967	9.39E-41	1
RPS2	2.59E-43	0.496462945	0.958	0.989	5.33E-39	1
RPL38	5.38E-43	0.590076825	0.845	0.945	1.11E-38	1
RPL8	7.92E-43	0.479422008	0.934	0.982	1.63E-38	1

RPS3	1.75E-42	0.466109647	0.947	0.983	3.60E-38	1
RPS29	2.04E-42	0.62684359	0.856	0.944	4.21E-38	1
RPL27A	4.60E-42	0.510962884	0.881	0.974	9.47E-38	1
TPT1	7.84E-42	0.704386181	0.989	0.993	1.61E-37	1
RPS13	1.06E-41	0.648784637	0.92	0.969	2.17E-37	1
RPL6	2.33E-41	0.490578662	0.918	0.974	4.80E-37	1
RPL7	2.39E-41	0.638978688	0.861	0.959	4.93E-37	1
UBA52	2.44E-41	0.638064706	0.827	0.943	5.02E-37	1
RPS4X	3.06E-41	0.615485162	0.909	0.976	6.30E-37	1
RPS25	5.11E-41	0.553189888	0.867	0.963	1.05E-36	1
RPS10	6.37E-41	0.508801215	0.909	0.968	1.31E-36	1
RPL41	1.50E-40	0.512518213	0.969	0.989	3.10E-36	1
EEF1B2	1.63E-40	0.648963475	0.869	0.942	3.36E-36	1
RPL34	2.68E-40	0.572923101	0.909	0.971	5.52E-36	1
NACA	4.31E-40	0.530512263	0.887	0.961	8.88E-36	1
RPS16	5.09E-40	0.492063932	0.934	0.979	1.05E-35	1
RPL27	3.67E-39	0.582526818	0.863	0.955	7.57E-35	1
RPL23A	5.82E-39	0.681182395	0.83	0.942	1.20E-34	1
RPL19	1.91E-38	0.574394668	0.883	0.958	3.94E-34	1
FTH1	1.79E-37	0.893248122	0.956	0.992	3.68E-33	1
RPL36	2.55E-35	0.514126955	0.85	0.946	5.25E-31	1
RPL3	1.36E-34	0.385209805	0.967	0.986	2.81E-30	1
RPS15	7.39E-34	0.454140707	0.903	0.959	1.52E-29	1
RPL31	1.55E-33	0.75995839	0.759	0.897	3.19E-29	1
RPS11	1.92E-33	0.546663953	0.889	0.963	3.95E-29	1
RPS20	6.45E-33	0.511802446	0.819	0.938	1.33E-28	1
RPS9	1.52E-32	0.441472942	0.863	0.957	3.12E-28	1
RPL28	9.25E-30	0.37976294	0.931	0.984	1.91E-25	1
RPL15	1.15E-29	0.437247383	0.967	0.99	2.36E-25	1
RPS21	2.44E-29	0.507588119	0.876	0.963	5.03E-25	1
RPS19	6.52E-28	0.350312764	0.958	0.986	1.34E-23	1
RPL4	5.38E-27	0.513399862	0.796	0.927	1.11E-22	1
ATP5F1E	1.43E-26	0.580503221	0.781	0.921	2.95E-22	1
RPS5	1.22E-25	0.420746062	0.874	0.958	2.51E-21	1
RPS8	2.57E-25	0.515408802	0.872	0.965	5.30E-21	1
RPL7A	9.85E-23	0.320711528	0.925	0.978	2.03E-18	1
RPL23	4.69E-22	0.43183558	0.777	0.933	9.66E-18	1
RPL18	9.64E-20	0.278423736	0.92	0.982	1.99E-15	1
TXNL1	9.87E-19	0.267825578	0.292	0.666	2.03E-14	1
GABARAPL2	1.53E-17	0.29778653	0.31	0.657	3.16E-13	1
CHMP5	7.03E-17	0.279579021	0.285	0.633	1.45E-12	1
SH3BGRL3	8.46E-17	0.300364568	0.288	0.622	1.74E-12	1
GADD45GIP1	9.72E-17	0.273283382	0.294	0.642	2.00E-12	1
RPL36A	2.36E-16	0.512801729	0.715	0.903	4.87E-12	1
EIF2S3	2.81E-16	0.316777416	0.29	0.645	5.78E-12	1

YBX1	6.35E-16	0.791358603	0.675	0.847	1.31E-11	1
RPLP0	7.66E-16	0.269424362	0.878	0.969	1.58E-11	1
EIF3M	1.38E-14	0.286917518	0.305	0.655	2.85E-10	1
RPS28	2.85E-14	0.298702791	0.852	0.96	5.88E-10	1
COMMD6	4.45E-14	0.547569126	0.664	0.849	9.16E-10	1
RBM3	7.54E-14	0.296178039	0.281	0.588	1.55E-09	1
BTF3	2.43E-13	0.467357741	0.673	0.861	5.00E-09	1
MRPL13	4.12E-13	0.293290029	0.283	0.573	8.49E-09	1
TRAPPC2L	5.22E-13	0.259184326	0.237	0.499	1.07E-08	1
CCDC167	5.22E-13	0.253048828	0.27	0.557	1.08E-08	1
RACK1	1.36E-12	0.357861216	0.783	0.95	2.81E-08	1
MRPL11	3.50E-12	0.25187134	0.312	0.642	7.21E-08	1
MAP3K13	5.46E-12	0.269677399	0.352	0.702	1.12E-07	1
EIF2A	1.82E-11	0.338462707	0.338	0.661	3.75E-07	1
HLA-B	1.98E-11	0.250028579	0.108	0.277	4.08E-07	1
SRSF9	2.16E-11	0.266752136	0.376	0.738	4.45E-07	1
POLR1D	2.85E-11	0.307597212	0.332	0.674	5.88E-07	1
DMAC1	7.06E-11	0.25531517	0.204	0.42	1.45E-06	1
FKBP1A	9.44E-11	0.262005251	0.383	0.738	1.94E-06	1
THOC7	1.35E-10	0.303624655	0.363	0.7	2.77E-06	1
RSL1D1	2.61E-10	0.285821652	0.356	0.711	5.37E-06	1
SMDT1	1.05E-09	0.252101616	0.369	0.728	2.16E-05	1
FABP1	1.58E-09	0.299092994	0.892	0.945	3.25E-05	1
MRPS33	1.61E-09	0.256240512	0.361	0.705	3.31E-05	1
ROMO1	1.67E-09	0.274030451	0.334	0.646	3.44E-05	1
UFC1	5.49E-09	0.394062587	0.321	0.621	0.000113128	1
CSAG1	8.16E-09	0.309694009	0.23	0.443	0.000168048	1
TMEM258	1.36E-08	0.299355695	0.387	0.726	0.000279479	1
FAU	1.57E-08	0.285570365	0.741	0.915	0.000323373	1
C19orf53	1.60E-08	0.340103749	0.356	0.681	0.000328923	1
SNRPC	1.69E-08	0.258120139	0.405	0.759	0.00034773	1
UQCC2	1.98E-08	0.301929212	0.365	0.679	0.000408011	1
HSBP1	2.90E-08	0.316080344	0.383	0.722	0.000597518	1
GAPDH	8.65E-08	0.25172571	0.812	0.956	0.001781999	1
EIF1	1.12E-07	0.284031409	0.743	0.929	0.002298916	1
UQCRB	1.28E-07	0.422924512	0.639	0.881	0.002644616	1
SUMO1	1.38E-07	0.35442274	0.412	0.765	0.002846446	1
HINT1	2.75E-07	0.310246166	0.759	0.93	0.005672702	1
COPS9	2.89E-07	0.25489149	0.381	0.697	0.005946791	1
UQCR11	3.53E-07	0.291960927	0.376	0.699	0.00727837	1
SNRPE	3.82E-07	0.269047287	0.409	0.738	0.007870787	1
PFDN5	4.45E-07	0.453466353	0.626	0.849	0.009161586	1
HMGN1	6.12E-07	0.280331455	0.427	0.784	0.01260473	1
HPD	7.18E-07	0.347869833	0.392	0.682	0.014779011	1
SERF2	9.61E-07	0.295035451	0.743	0.929	0.01979775	1

BOLA3	1.22E-06	0.380523328	0.33	0.598	0.025083624	1
PRDX5	1.57E-06	0.257004308	0.418	0.763	0.03237884	1
APRT	1.70E-06	0.309035489	0.376	0.681	0.03506269	1
ATP5PO	1.74E-06	0.274385222	0.398	0.735	0.035900002	1
RTRAF	2.00E-06	0.338532914	0.374	0.678	0.04117619	1
NDUFS6	2.66E-06	0.37976181	0.4	0.719	0.054821002	1
NTAN1	2.72E-06	0.347909968	0.179	0.318	0.056106196	1
ATP5ME	4.07E-06	0.339167166	0.414	0.742	0.083740004	1
RPS26	4.95E-06	0.419808854	0.628	0.876	0.10186781	1
UQCRH	6.29E-06	0.434179744	0.619	0.855	0.129610886	1
PPDPF	6.70E-06	0.38032924	0.235	0.411	0.137946611	1
EEF1D	1.69E-05	0.33298076	0.65	0.875	0.348142074	1
ATP5MPL	2.48E-05	0.303132829	0.427	0.762	0.510490284	1
NPM1	2.66E-05	0.34062316	0.662	0.882	0.54725255	1
ANAPC11	3.02E-05	0.263825641	0.436	0.768	0.621280726	1
HLA-DRA	3.64E-05	0.607727092	0.217	0.367	0.749859566	1
COX7C	5.82E-05	0.340886297	0.635	0.863	1	1
TXN	7.69E-05	0.378423306	0.668	0.886	1	1
EIF3E	0.00010496	0.439437891	0.613	0.865	1	1
NDUFB5	0.000117868	0.353934739	0.409	0.701	1	1
TMSB10	0.000182975	0.472654613	0.653	0.832	1	1
SAP18	0.00027566	0.272239704	0.473	0.799	1	1
NDUFB2	0.000283923	0.288799891	0.46	0.799	1	1
DDT	0.000376051	0.29035634	0.692	0.882	1	1
MIF	0.000519464	0.253117175	0.462	0.782	1	1
PARK7	0.00134275	0.279180523	0.442	0.758	1	1
GNG5	0.001378926	0.298080162	0.491	0.82	1	1
UBL5	0.001667375	0.329194854	0.454	0.778	1	1
RPL17	0.002204968	0.340236072	0.427	0.746	1	1
CHCHD10	0.002359089	0.256816581	0.675	0.895	1	1
TMSB4X	0.003939001	0.927001825	0.489	0.6	1	1
SNRPD2	0.004605487	0.358118288	0.423	0.709	1	1
ATP5MF	0.006946369	0.280509281	0.646	0.883	1	1
MYL12A	0.00882061	0.275541534	0.531	0.835	1	1
CYP3A7	1.18E-81	0.501630567	0.65	0.208	2.44E-77	2
CFHR1	7.68E-74	0.726084664	0.961	0.657	1.58E-69	2
IGFBP2	2.71E-73	0.946344157	0.971	0.673	5.58E-69	2
SERPINA5	1.04E-72	0.498510433	0.779	0.368	2.14E-68	2
ITIH3	4.98E-71	0.784918167	0.968	0.683	1.03E-66	2
SLC2A2	7.70E-69	0.559346337	0.924	0.542	1.59E-64	2
TNFSF10	5.54E-67	0.50614795	0.86	0.515	1.14E-62	2
SERPINA11	2.33E-62	0.40121668	0.748	0.366	4.79E-58	2
SLC9A3R2	2.60E-61	0.474791088	0.926	0.658	5.36E-57	2
HSD17B6	8.96E-61	0.572571157	0.885	0.552	1.85E-56	2
APOH	6.11E-59	0.497500387	1	0.945	1.26E-54	2

ITM2B	9.81E-58	0.428425978	0.995	0.872	2.02E-53	2
NT5E	4.20E-54	0.393652837	0.755	0.426	8.65E-50	2
ITIH2	4.51E-54	0.510697211	0.963	0.698	9.29E-50	2
COQ8A	2.03E-51	0.298962513	0.672	0.319	4.17E-47	2
CYP2C8	2.15E-51	0.414480516	0.468	0.145	4.42E-47	2
FOXN3	2.77E-50	0.328184224	0.718	0.378	5.70E-46	2
CFHR2	1.84E-49	0.260822675	0.659	0.316	3.79E-45	2
IGSF8	4.63E-49	0.358389492	0.701	0.362	9.54E-45	2
SERPINA4	7.62E-49	0.253115637	0.61	0.261	1.57E-44	2
SCCPDH	7.90E-49	0.317890551	0.789	0.48	1.63E-44	2
A1BG	2.99E-48	0.521658239	0.951	0.725	6.17E-44	2
AOX1	3.47E-48	0.435541585	0.836	0.516	7.14E-44	2
ACSM2A	5.03E-48	0.267959998	0.515	0.179	1.04E-43	2
PON3	9.61E-47	0.450003435	0.907	0.59	1.98E-42	2
CPB2	3.49E-45	0.351420314	0.885	0.574	7.19E-41	2
RRBP1	4.90E-45	0.481767573	0.912	0.638	1.01E-40	2
SERPINA6	1.02E-43	0.337781836	0.787	0.48	2.09E-39	2
EPHX1	1.99E-43	0.498406367	0.939	0.716	4.11E-39	2
KNG1	2.26E-43	0.426747003	0.949	0.689	4.65E-39	2
SERPINA7	8.50E-43	0.259285438	0.429	0.141	1.75E-38	2
DEPP1	1.73E-42	0.286305542	0.75	0.402	3.56E-38	2
ATRN	3.33E-40	0.264068211	0.725	0.404	6.86E-36	2
HAAO	1.33E-39	0.262729798	0.819	0.499	2.73E-35	2
CTSZ	1.35E-39	0.291105416	0.868	0.594	2.79E-35	2
NR0B2	2.59E-39	0.30659546	0.728	0.402	5.32E-35	2
SPP2	3.77E-39	0.359078239	0.86	0.554	7.76E-35	2
BCAM	9.73E-38	0.2783062	0.797	0.496	2.00E-33	2
SLC2A4RG	1.16E-37	0.278875575	0.809	0.497	2.38E-33	2
PDK4	1.77E-37	0.284619342	0.676	0.354	3.64E-33	2
ARL4D	2.20E-37	0.290423103	0.765	0.449	4.54E-33	2
VTN	2.59E-37	0.369441077	0.99	0.862	5.33E-33	2
MTR	8.17E-37	0.250643486	0.659	0.353	1.68E-32	2
CRLS1	8.44E-37	0.265695955	0.816	0.542	1.74E-32	2
HAL	2.12E-36	0.309049504	0.799	0.514	4.37E-32	2
ABAT	4.03E-36	0.275162138	0.797	0.489	8.30E-32	2
ESF1	4.13E-36	0.293939679	0.865	0.574	8.50E-32	2
PROX1	4.73E-36	0.314903159	0.806	0.5	9.75E-32	2
CHD6	2.17E-35	0.319380866	0.743	0.432	4.46E-31	2
AHCY	2.60E-35	0.351412417	0.902	0.656	5.35E-31	2
HAO1	3.06E-35	0.275683351	0.885	0.601	6.29E-31	2
NAA20	6.70E-35	0.297016526	0.897	0.635	1.38E-30	2
SERPING1	8.50E-35	0.357226486	0.919	0.672	1.75E-30	2
CES2	1.40E-34	0.278943005	0.806	0.512	2.88E-30	2
UNC93A	2.67E-34	0.256069759	0.556	0.259	5.49E-30	2
CYP2D6	3.30E-34	0.365631866	0.843	0.534	6.79E-30	2

PROC	3.26E-33	0.278557884	0.953	0.722	6.71E-29	2
HMGCS2	4.61E-33	0.349068823	0.87	0.589	9.50E-29	2
AGT	5.96E-33	0.32176172	1	0.902	1.23E-28	2
CTSA	9.55E-33	0.262693251	0.909	0.642	1.97E-28	2
CYP4A11	1.66E-32	0.277175504	0.657	0.36	3.42E-28	2
GLYCTK	2.45E-32	0.291935632	0.904	0.598	5.04E-28	2
PCK1	2.94E-32	0.358714567	0.858	0.548	6.06E-28	2
DHCR24	7.38E-32	0.337409421	0.904	0.627	1.52E-27	2
ADH1B	1.32E-31	0.293052733	0.561	0.255	2.71E-27	2
GATM	1.84E-30	0.327620527	0.926	0.715	3.78E-26	2
DHTKD1	5.70E-30	0.278246472	0.73	0.444	1.17E-25	2
CD46	1.35E-29	0.260175348	0.821	0.565	2.77E-25	2
EIF6	3.79E-29	0.266679262	0.89	0.66	7.80E-25	2
TMEM176B	1.20E-28	0.34401747	0.978	0.841	2.46E-24	2
ALDH2	5.14E-28	0.273775068	0.904	0.667	1.06E-23	2
P4HA1	9.48E-28	0.260490677	0.718	0.439	1.95E-23	2
CLU	2.95E-27	0.313211582	0.958	0.758	6.08E-23	2
HSD11B1	3.84E-27	0.384028962	0.333	0.119	7.90E-23	2
IL6R	3.63E-26	0.253510845	0.848	0.571	7.47E-22	2
RBP4	8.45E-26	0.325621235	1	0.976	1.74E-21	2
PEG10	5.29E-25	0.375557716	0.89	0.643	1.09E-20	2
DCXR	6.61E-25	0.301079075	0.944	0.778	1.36E-20	2
APOM	1.41E-24	0.357919456	0.988	0.909	2.90E-20	2
MASP2	2.51E-24	0.258787141	0.855	0.62	5.18E-20	2
TMEM56	2.75E-24	0.267153985	0.882	0.599	5.67E-20	2
TTR	1.83E-23	0.338146286	0.966	0.783	3.78E-19	2
FMO5	2.16E-23	0.316884256	0.939	0.72	4.45E-19	2
ERBB3	3.28E-23	0.299537644	0.868	0.606	6.76E-19	2
GALK1	6.30E-23	0.251745783	0.875	0.632	1.30E-18	2
IDH1	1.14E-22	0.25897569	0.922	0.694	2.34E-18	2
SERPINC1	2.03E-22	0.288176629	0.983	0.768	4.19E-18	2
AZGP1	3.38E-22	0.290208617	0.983	0.826	6.97E-18	2
TF	6.72E-22	0.251885648	0.978	0.807	1.38E-17	2
LEPR	1.64E-20	0.275073463	0.924	0.669	3.37E-16	2
TMEM176A	2.08E-19	0.264266774	0.985	0.863	4.27E-15	2
MT-CYB	3.91E-17	0.273145866	1	1	8.06E-13	2
RARRES2	5.52E-17	0.261170485	0.983	0.873	1.14E-12	2
AKR1B10	4.38E-15	0.338862685	0.625	0.423	9.01E-11	2
MT-CO1	1.35E-11	0.258585114	1	0.998	2.78E-07	2
CPS1	1.71E-71	0.810797742	0.942	0.61	3.53E-67	3
MEG8	9.96E-61	0.490279083	0.786	0.388	2.05E-56	3
ADH4	7.44E-59	0.356653927	0.788	0.376	1.53E-54	3
DUSP9	3.05E-58	0.552515867	0.839	0.45	6.28E-54	3
MEG3	3.92E-54	0.806649479	0.844	0.54	8.08E-50	3
ACSL4	6.00E-54	0.502780827	0.897	0.559	1.23E-49	3

VCAM1	1.70E-52	0.344095165	0.746	0.356	3.50E-48	3
COL5A2	7.21E-52	0.3346149	0.69	0.321	1.48E-47	3
C9	2.52E-50	0.254688649	0.534	0.177	5.18E-46	3
FASN	9.07E-49	0.533455819	0.909	0.565	1.87E-44	3
ZBED3	4.40E-48	0.258113083	0.627	0.286	9.06E-44	3
AFP	1.71E-46	0.579945142	0.544	0.212	3.52E-42	3
GPC3	1.65E-45	0.429611788	0.982	0.909	3.40E-41	3
PEG10	1.72E-45	0.596555427	0.945	0.632	3.54E-41	3
HMGCR	2.51E-45	0.42840426	0.715	0.375	5.18E-41	3
SMARCA1	8.53E-45	0.354854059	0.814	0.479	1.76E-40	3
TKFC	2.00E-44	0.346461547	0.771	0.428	4.11E-40	3
LSS	5.60E-44	0.333525918	0.668	0.319	1.15E-39	3
MVD	4.26E-43	0.349382235	0.662	0.321	8.77E-39	3
GPAM	6.11E-43	0.441184494	0.849	0.53	1.26E-38	3
LECT2	6.44E-43	0.329495094	0.783	0.432	1.33E-38	3
IDH1	4.18E-41	0.385616284	0.95	0.689	8.61E-37	3
MBNL3	6.34E-41	0.358502503	0.761	0.427	1.31E-36	3
APOB	7.43E-40	0.381220744	0.992	0.932	1.53E-35	3
TRIM24	1.37E-39	0.300002612	0.821	0.524	2.83E-35	3
MAGED1	6.83E-39	0.25623322	0.703	0.39	1.41E-34	3
SQLE	7.63E-39	0.39950912	0.826	0.501	1.57E-34	3
FBLN1	1.78E-38	0.253717914	0.562	0.252	3.68E-34	3
TM7SF2	2.34E-38	0.297487473	0.829	0.487	4.82E-34	3
CDK6	3.59E-38	0.278727033	0.786	0.466	7.40E-34	3
GABPB1-AS1	3.90E-38	0.286154801	0.652	0.326	8.04E-34	3
FDPS	1.62E-37	0.504664906	0.972	0.743	3.33E-33	3
MIR122HG	1.64E-37	0.300287456	0.69	0.384	3.37E-33	3
HSD17B12	3.13E-37	0.259048782	0.761	0.433	6.44E-33	3
UGT1A1	4.44E-37	0.286996252	0.761	0.445	9.14E-33	3
ODC1	1.01E-36	0.344423373	0.695	0.383	2.07E-32	3
FIGN	1.06E-36	0.251597417	0.574	0.271	2.19E-32	3
IQGAP2	3.83E-36	0.339650904	0.839	0.552	7.89E-32	3
PSMD1	1.46E-35	0.338888691	0.771	0.476	3.00E-31	3
KCNQ1OT1	3.50E-35	0.544985741	0.809	0.529	7.21E-31	3
TCAF1	5.06E-35	0.258557463	0.778	0.474	1.04E-30	3
TOB1	3.98E-34	0.266642035	0.851	0.554	8.19E-30	3
HMGCS1	2.21E-33	0.493696983	0.751	0.469	4.56E-29	3
SLC38A4	5.23E-33	0.27955686	0.685	0.397	1.08E-28	3
SERPINF2	8.08E-33	0.308405159	0.897	0.618	1.66E-28	3
PCSK9	1.14E-31	0.255010822	0.562	0.276	2.36E-27	3
NCOA4	2.27E-31	0.291974324	0.869	0.609	4.67E-27	3
PNPLA3	3.14E-31	0.258617497	0.668	0.363	6.46E-27	3
TF	4.56E-31	0.387366152	0.98	0.808	9.38E-27	3
MSMO1	6.90E-31	0.275835403	0.864	0.554	1.42E-26	3
AKR1C4	7.72E-31	0.320249318	0.894	0.608	1.59E-26	3

CLTC	2.29E-30	0.266066869	0.836	0.565	4.73E-26	3
SLC2A2	2.93E-30	0.281965144	0.884	0.553	6.03E-26	3
BHMT2	7.66E-30	0.252222044	0.826	0.561	1.58E-25	3
ONECUT2	9.40E-30	0.370194167	0.912	0.658	1.94E-25	3
CXCL12	4.83E-29	0.257256404	0.776	0.497	9.94E-25	3
FST	1.97E-28	0.359303262	0.877	0.651	4.05E-24	3
PNN	3.90E-28	0.277300511	0.834	0.548	8.02E-24	3
HIST1H1C	6.82E-28	0.419679565	0.866	0.621	1.40E-23	3
EBP	9.77E-28	0.342944402	0.914	0.643	2.01E-23	3
DUSP6	1.45E-27	0.283748266	0.761	0.473	2.99E-23	3
HMGCS2	3.39E-27	0.275795468	0.897	0.585	6.98E-23	3
GSTA1	1.48E-26	0.383076489	0.952	0.694	3.05E-22	3
TMEM97	3.03E-26	0.252471634	0.753	0.454	6.24E-22	3
HIPK2	3.72E-24	0.254655283	0.798	0.529	7.66E-20	3
NCL	8.89E-24	0.276296542	0.904	0.699	1.83E-19	3
APOA1	1.05E-23	0.289096595	1	0.984	2.16E-19	3
FABP1	6.43E-23	0.442812719	0.992	0.921	1.33E-18	3
MDK	3.66E-22	0.262685588	0.821	0.568	7.55E-18	3
AGT	1.35E-20	0.273278168	0.995	0.904	2.78E-16	3
ITIH2	2.82E-20	0.265160252	0.947	0.703	5.80E-16	3
GC	3.31E-16	0.287127457	0.932	0.756	6.81E-12	3
MT-ATP6	4.41E-15	0.260098688	1	1	9.08E-11	3
MALAT1	7.56E-12	0.283546855	0.95	0.906	1.56E-07	3
TOP2A	4.12E-164	1.399994866	0.698	0.073	8.50E-160	4
SPC25	1.68E-159	0.516068454	0.427	0.009	3.45E-155	4
PBK	2.00E-155	0.52721586	0.491	0.021	4.12E-151	4
KIFC1	1.73E-151	0.524544937	0.491	0.023	3.57E-147	4
PCLAF	6.42E-151	0.61483251	0.565	0.04	1.32E-146	4
ZWINT	1.55E-149	0.555963854	0.513	0.029	3.19E-145	4
TPX2	3.90E-145	0.868359907	0.578	0.047	8.03E-141	4
UBE2C	7.56E-143	1.250887834	0.599	0.056	1.56E-138	4
SGO1	2.90E-141	0.531346594	0.474	0.024	5.97E-137	4
ASPM	9.46E-136	0.657917055	0.522	0.039	1.95E-131	4
MKI67	1.40E-135	0.597434816	0.47	0.026	2.88E-131	4
CDK1	5.25E-135	0.54147723	0.444	0.021	1.08E-130	4
MYBL2	1.29E-125	0.416801609	0.418	0.02	2.65E-121	4
RRM2	2.63E-124	1.040101768	0.552	0.057	5.41E-120	4
NCAPG	2.67E-124	0.409139548	0.384	0.014	5.50E-120	4
NUF2	4.78E-124	0.622142411	0.422	0.022	9.85E-120	4
CKAP2L	5.09E-123	0.342050856	0.435	0.025	1.05E-118	4
E2F8	5.59E-121	0.281161067	0.332	0.008	1.15E-116	4
MELK	6.04E-120	0.27591443	0.384	0.016	1.24E-115	4
HJURP	3.64E-118	0.309102984	0.332	0.009	7.49E-114	4
NEK2	6.90E-118	0.410981581	0.345	0.011	1.42E-113	4
MAD2L1	1.79E-117	0.488312957	0.504	0.045	3.68E-113	4

TCF19	7.94E-116	0.329547385	0.418	0.025	1.63E-111	4
RAD51AP1	2.53E-115	0.308246659	0.418	0.025	5.20E-111	4
TK1	5.71E-113	0.271693486	0.371	0.017	1.18E-108	4
TYMS	3.53E-111	1.021423519	0.685	0.129	7.26E-107	4
KIF15	7.26E-111	0.313721208	0.341	0.013	1.49E-106	4
BIRC5	1.05E-109	0.80636194	0.56	0.069	2.16E-105	4
CENPU	1.64E-107	0.25581776	0.414	0.029	3.38E-103	4
NUSAP1	9.22E-105	0.742688048	0.534	0.068	1.90E-100	4
ORC6	1.33E-104	0.355418705	0.418	0.032	2.75E-100	4
CENPM	1.10E-102	0.320437894	0.478	0.049	2.27E-98	4
AURKB	2.02E-102	0.316911988	0.323	0.013	4.15E-98	4
CCNA2	7.16E-102	0.312381275	0.306	0.01	1.47E-97	4
NDC80	9.47E-98	0.331113009	0.328	0.016	1.95E-93	4
TTK	1.20E-97	0.301627771	0.302	0.011	2.48E-93	4
ANLN	3.65E-97	0.292219594	0.323	0.015	7.52E-93	4
CCNB2	3.54E-96	0.580596309	0.397	0.031	7.30E-92	4
CENPF	4.25E-96	0.754420072	0.457	0.048	8.75E-92	4
DEPDC1	5.35E-95	0.408739588	0.336	0.019	1.10E-90	4
HMGB2	4.95E-94	1.154606688	0.797	0.279	1.02E-89	4
CDT1	2.90E-92	0.395844409	0.517	0.072	5.97E-88	4
ARHGAP11A	2.30E-90	0.324821455	0.366	0.028	4.73E-86	4
CDC20	8.43E-89	0.519490645	0.349	0.025	1.74E-84	4
FOXM1	9.11E-88	0.266445095	0.302	0.015	1.88E-83	4
TUBA1B	4.79E-87	1.350300935	0.935	0.582	9.86E-83	4
PRC1	2.80E-86	0.319066895	0.345	0.025	5.76E-82	4
CENPA	3.57E-86	0.365177561	0.272	0.011	7.35E-82	4
GTSE1	6.62E-86	0.375599193	0.284	0.013	1.36E-81	4
GINS2	3.69E-83	0.272178329	0.371	0.034	7.61E-79	4
PLK1	9.42E-83	0.434019232	0.293	0.016	1.94E-78	4
E2F1	1.81E-81	0.365474573	0.375	0.037	3.73E-77	4
CDKN3	1.18E-78	0.683228712	0.556	0.108	2.43E-74	4
TUBB	8.89E-77	1.108508038	0.957	0.781	1.83E-72	4
SGO2	5.29E-75	0.508726375	0.457	0.071	1.09E-70	4
C21orf58	6.65E-75	0.28657054	0.362	0.038	1.37E-70	4
STMN1	1.76E-72	1.2175454	0.845	0.448	3.63E-68	4
PRR11	3.78E-71	0.261934814	0.263	0.016	7.79E-67	4
UBE2T	1.30E-70	0.462207966	0.5	0.096	2.69E-66	4
FEN1	5.77E-69	0.275785348	0.431	0.068	1.19E-64	4
AURKA	5.33E-68	0.486550981	0.397	0.056	1.10E-63	4
HELLS	8.29E-68	0.495442719	0.517	0.11	1.71E-63	4
PHF19	1.29E-67	0.388756765	0.513	0.103	2.65E-63	4
H2AFX	4.72E-64	0.496093607	0.56	0.136	9.73E-60	4
PTTG1	1.21E-63	0.711216459	0.509	0.107	2.49E-59	4
SMC4	3.05E-63	0.850951975	0.711	0.297	6.28E-59	4
CCNB1	3.60E-63	0.653310661	0.392	0.061	7.41E-59	4

TACC3	3.45E-62	0.251916928	0.263	0.021	7.11E-58	4
CENPW	1.02E-60	0.420149573	0.431	0.077	2.10E-56	4
MCM6	8.02E-60	0.504220475	0.427	0.082	1.65E-55	4
CENPE	8.34E-58	0.253279127	0.259	0.023	1.72E-53	4
ECT2	3.64E-57	0.250691977	0.362	0.054	7.49E-53	4
CKS2	1.41E-56	0.788115871	0.569	0.164	2.90E-52	4
MCM2	1.86E-55	0.327253336	0.427	0.085	3.83E-51	4
DHFR	6.76E-55	0.428876418	0.552	0.156	1.39E-50	4
H2AFZ	1.34E-54	0.915549489	0.853	0.574	2.76E-50	4
DTYMK	9.18E-54	0.744453795	0.737	0.361	1.89E-49	4
CDCA7	1.26E-53	0.282408817	0.31	0.042	2.59E-49	4
CKS1B	1.87E-52	0.889805726	0.823	0.506	3.86E-48	4
UBE2S	5.73E-51	0.77539655	0.634	0.231	1.18E-46	4
MCM4	3.46E-49	0.377925751	0.422	0.096	7.13E-45	4
ITGB3BP	1.67E-45	0.346519929	0.483	0.136	3.43E-41	4
HMGN2	8.95E-45	0.977114505	0.815	0.456	1.84E-40	4
FAM111A	6.41E-44	0.251973646	0.435	0.112	1.32E-39	4
EZH2	1.36E-41	0.421681087	0.522	0.177	2.81E-37	4
PCNA	1.57E-40	0.908383669	0.599	0.281	3.23E-36	4
DEK	4.13E-39	0.758188144	0.841	0.68	8.51E-35	4
KIF20B	1.03E-38	0.431692377	0.435	0.127	2.12E-34	4
SNRPD1	1.92E-38	0.668777005	0.819	0.588	3.95E-34	4
LMNB1	1.15E-37	0.295194658	0.487	0.154	2.36E-33	4
MCM5	1.47E-37	0.369934293	0.5	0.178	3.03E-33	4
CENPN	2.44E-37	0.332148633	0.466	0.144	5.03E-33	4
CDC25B	1.08E-36	0.343259837	0.336	0.076	2.23E-32	4
SMC2	3.04E-36	0.44298275	0.547	0.212	6.27E-32	4
HMGB1	2.18E-35	0.645544202	0.935	0.823	4.50E-31	4
PTMA	2.32E-35	0.506016511	0.983	0.938	4.78E-31	4
TUBB4B	7.47E-35	0.755322302	0.832	0.587	1.54E-30	4
RAN	9.11E-34	0.630180317	0.853	0.666	1.88E-29	4
MIS18A	4.61E-33	0.270771231	0.466	0.162	9.50E-29	4
DSN1	8.69E-33	0.278249355	0.418	0.13	1.79E-28	4
CCDC34	1.19E-32	0.397767481	0.534	0.225	2.44E-28	4
FBLN1	1.96E-32	0.400719583	0.612	0.272	4.04E-28	4
H2AFV	4.62E-32	0.641499412	0.767	0.572	9.51E-28	4
TFDP1	4.93E-32	0.264671337	0.483	0.175	1.02E-27	4
DBF4	1.84E-31	0.287506533	0.457	0.16	3.78E-27	4
KPNA2	1.19E-30	0.582648564	0.547	0.239	2.45E-26	4
MGME1	6.33E-30	0.292011884	0.56	0.254	1.30E-25	4
DNMT1	2.86E-29	0.381345533	0.496	0.211	5.88E-25	4
GMNN	2.85E-28	0.546235106	0.703	0.468	5.88E-24	4
RANBP1	1.20E-27	0.660793522	0.823	0.68	2.47E-23	4
LSM5	8.25E-27	0.538834361	0.81	0.613	1.70E-22	4
JPT1	9.05E-27	0.572729264	0.625	0.334	1.86E-22	4

MCM3	1.63E-26	0.545995311	0.552	0.294	3.36E-22	4
TMPO	2.99E-26	0.412997037	0.664	0.39	6.15E-22	4
SNRPF	3.50E-26	0.509805438	0.81	0.638	7.20E-22	4
CCHCR1	5.39E-26	0.359406052	0.5	0.202	1.11E-21	4
MCM7	1.06E-25	0.51458448	0.616	0.372	2.18E-21	4
DKK1	1.81E-25	0.442853375	0.414	0.152	3.73E-21	4
PGP	2.19E-25	0.398122593	0.629	0.359	4.52E-21	4
CTNNAL1	4.02E-25	0.278865197	0.483	0.203	8.29E-21	4
RPA3	5.45E-25	0.508158567	0.746	0.516	1.12E-20	4
TUBG1	6.55E-25	0.353097021	0.586	0.318	1.35E-20	4
MIS18BP1	6.55E-25	0.288473054	0.44	0.173	1.35E-20	4
ACTB	1.98E-24	0.405063655	0.978	0.897	4.07E-20	4
UNG	1.80E-23	0.323052147	0.487	0.216	3.71E-19	4
ANP32E	2.30E-23	0.506213797	0.733	0.544	4.73E-19	4
TUBA1C	3.21E-23	0.607022406	0.603	0.327	6.62E-19	4
SNRNP25	9.17E-23	0.304393953	0.543	0.273	1.89E-18	4
SRSF3	1.53E-22	0.405301033	0.823	0.632	3.14E-18	4
USP1	1.92E-22	0.274288848	0.547	0.29	3.94E-18	4
CALM2	2.24E-22	0.59393038	0.905	0.781	4.60E-18	4
DUT	4.29E-22	0.508170195	0.595	0.354	8.83E-18	4
JPT2	5.17E-22	0.313008574	0.543	0.27	1.07E-17	4
AKR1C4	6.30E-22	0.500882806	0.832	0.639	1.30E-17	4
HNRNPA3	6.95E-22	0.426971791	0.853	0.747	1.43E-17	4
HSP90AA1	7.01E-22	0.424943613	0.922	0.805	1.44E-17	4
SNRPB	7.91E-22	0.458157619	0.841	0.727	1.63E-17	4
PHGDH	2.33E-21	0.304504924	0.599	0.329	4.79E-17	4
MZT1	2.64E-21	0.375626336	0.556	0.304	5.44E-17	4
PSIP1	2.76E-21	0.374967166	0.616	0.376	5.69E-17	4
ALYREF	3.86E-21	0.458144115	0.612	0.371	7.95E-17	4
NUCKS1	1.51E-20	0.490833603	0.815	0.698	3.12E-16	4
PEG10	3.83E-20	0.518124583	0.832	0.672	7.89E-16	4
TMEM106C	8.33E-20	0.403135746	0.655	0.458	1.72E-15	4
HNRNPAB	1.11E-19	0.386992507	0.595	0.353	2.29E-15	4
ATAD2	1.43E-19	0.339850882	0.573	0.347	2.94E-15	4
PPIL1	1.70E-19	0.267984344	0.496	0.234	3.49E-15	4
SNRPA	4.20E-19	0.291730329	0.595	0.347	8.65E-15	4
NT5DC2	6.81E-19	0.334427856	0.634	0.42	1.40E-14	4
CKAP5	8.45E-19	0.298156942	0.384	0.16	1.74E-14	4
LSM3	1.61E-18	0.401158706	0.793	0.642	3.31E-14	4
NASP	2.51E-18	0.379632087	0.638	0.432	5.17E-14	4
PTGES3	3.42E-18	0.368456346	0.853	0.716	7.05E-14	4
HSPE1	4.46E-18	0.354867626	0.922	0.882	9.18E-14	4
CDKN2C	4.78E-18	0.369555925	0.53	0.285	9.84E-14	4
CSE1L	6.48E-18	0.2916363	0.573	0.351	1.33E-13	4
SRSF2	7.62E-18	0.361058581	0.75	0.615	1.57E-13	4

TXN	1.50E-17	0.420603253	0.918	0.832	3.09E-13	4
GSTA1	1.57E-17	0.454462215	0.879	0.724	3.23E-13	4
COMMD4	2.42E-17	0.304741398	0.616	0.392	4.99E-13	4
CENPX	4.39E-17	0.521177017	0.711	0.562	9.05E-13	4
GGCT	5.45E-17	0.253877016	0.586	0.359	1.12E-12	4
GPX2	7.88E-17	0.428688856	0.586	0.338	1.62E-12	4
RBM8A	1.09E-16	0.41585614	0.832	0.704	2.25E-12	4
H2AFY	1.19E-16	0.334761111	0.565	0.341	2.46E-12	4
ISYNA1	1.25E-16	0.288523015	0.461	0.235	2.58E-12	4
H1FX	1.36E-16	0.441320778	0.741	0.544	2.80E-12	4
HSPD1	1.69E-16	0.303647746	0.948	0.891	3.48E-12	4
DKC1	2.50E-16	0.362431282	0.599	0.378	5.15E-12	4
BRI3BP	3.22E-16	0.255562597	0.457	0.232	6.62E-12	4
NAP1L1	3.30E-16	0.337467607	0.806	0.676	6.79E-12	4
RAD21	3.83E-16	0.460744359	0.672	0.508	7.90E-12	4
HIST1H4C	4.17E-16	0.833467454	0.591	0.393	8.59E-12	4
PPIA	5.86E-16	0.395251549	0.922	0.834	1.21E-11	4
POLE3	6.60E-16	0.318960025	0.569	0.336	1.36E-11	4
GCHFR	9.00E-16	0.38541445	0.733	0.539	1.85E-11	4
ATP5MC1	1.00E-15	0.346627188	0.806	0.661	2.07E-11	4
HMGN1	2.22E-15	0.328941527	0.802	0.7	4.58E-11	4
CCDC88A	4.55E-15	0.482895906	0.625	0.455	9.36E-11	4
SNRPG	5.01E-15	0.392152852	0.784	0.653	1.03E-10	4
UPK3A	5.13E-15	0.27872221	0.638	0.431	1.06E-10	4
LSM8	8.41E-15	0.297228889	0.716	0.564	1.73E-10	4
BUB3	9.77E-15	0.296635399	0.603	0.406	2.01E-10	4
SET	1.33E-14	0.333344748	0.823	0.693	2.74E-10	4
CDKN2A	1.58E-14	0.324598962	0.711	0.557	3.26E-10	4
XRCC5	1.74E-14	0.358420155	0.741	0.649	3.58E-10	4
SNRPD3	1.77E-14	0.309174984	0.789	0.672	3.65E-10	4
PSMD14	2.38E-14	0.266140218	0.621	0.416	4.91E-10	4
CYCS	3.54E-14	0.292762284	0.767	0.618	7.29E-10	4
FADS1	3.81E-14	0.312559554	0.556	0.349	7.84E-10	4
HNRNPDL	4.06E-14	0.337107392	0.737	0.587	8.36E-10	4
RBMX	4.57E-14	0.340470311	0.685	0.49	9.42E-10	4
ADH4	4.58E-14	0.292521823	0.642	0.427	9.43E-10	4
PEA15	4.73E-14	0.298518436	0.53	0.298	9.74E-10	4
CCT6A	6.14E-14	0.290321196	0.728	0.606	1.27E-09	4
CMSS1	7.60E-14	0.260879583	0.595	0.375	1.56E-09	4
ELOVL2	8.98E-14	0.30247435	0.582	0.392	1.85E-09	4
SFN	1.01E-13	0.250661281	0.573	0.352	2.07E-09	4
KPNB1	1.35E-13	0.377479514	0.672	0.562	2.78E-09	4
ACTL6A	1.42E-13	0.253754179	0.552	0.332	2.93E-09	4
DYNLL1	1.62E-13	0.404361198	0.875	0.772	3.33E-09	4
AKR1C3	2.85E-13	0.401193822	0.888	0.798	5.86E-09	4

UQCC2	2.92E-13	0.312415645	0.741	0.6	6.01E-09	4
BOLA3	4.07E-13	0.280392755	0.698	0.525	8.39E-09	4
HSPA8	4.13E-13	0.385852472	0.767	0.606	8.51E-09	4
CALM3	7.30E-13	0.264846915	0.625	0.422	1.50E-08	4
DUSP9	8.13E-13	0.332134399	0.716	0.497	1.67E-08	4
TPM3	8.69E-13	0.304715019	0.789	0.664	1.79E-08	4
MARCKS	1.83E-12	0.301980854	0.81	0.642	3.78E-08	4
TMEM14A	1.92E-12	0.30446618	0.69	0.543	3.95E-08	4
MARCKSL1	2.33E-12	0.253620122	0.595	0.393	4.81E-08	4
SNRPE	2.42E-12	0.290408364	0.784	0.657	4.98E-08	4
CDK4	2.57E-12	0.344283661	0.578	0.413	5.29E-08	4
TRIOBP	2.95E-12	0.255614149	0.543	0.342	6.08E-08	4
GGH	3.43E-12	0.296195447	0.711	0.561	7.05E-08	4
LYZ	4.13E-12	0.382584863	0.888	0.715	8.51E-08	4
SNX5	4.67E-12	0.292782802	0.672	0.533	9.61E-08	4
LSM2	5.87E-12	0.348529934	0.707	0.57	1.21E-07	4
SFPQ	7.60E-12	0.359563532	0.685	0.545	1.57E-07	4
LSM4	8.92E-12	0.251165483	0.698	0.583	1.84E-07	4
MSH6	9.10E-12	0.291420096	0.478	0.31	1.87E-07	4
SNRPC	1.13E-11	0.298491659	0.806	0.672	2.33E-07	4
NME1	1.15E-11	0.272593157	0.754	0.59	2.37E-07	4
FUS	1.42E-11	0.352567742	0.716	0.617	2.92E-07	4
TMSB10	1.77E-11	0.274958645	0.905	0.782	3.64E-07	4
CBX3	2.07E-11	0.281818102	0.698	0.571	4.26E-07	4
LECT2	2.29E-11	0.256888164	0.659	0.476	4.72E-07	4
GAMT	2.42E-11	0.30492668	0.81	0.688	4.98E-07	4
ANP32B	2.76E-11	0.297854417	0.728	0.634	5.69E-07	4
SEM1	2.77E-11	0.272010736	0.866	0.757	5.70E-07	4
PDAP1	3.70E-11	0.304125776	0.759	0.611	7.62E-07	4
BANF1	3.83E-11	0.364946196	0.75	0.623	7.88E-07	4
GLO1	4.06E-11	0.294906294	0.784	0.69	8.35E-07	4
LBR	4.25E-11	0.33852415	0.655	0.494	8.74E-07	4
NCL	4.30E-11	0.321650635	0.832	0.724	8.85E-07	4
RAB5IF	5.64E-11	0.281510607	0.746	0.613	1.16E-06	4
HNRNPD	6.51E-11	0.275220595	0.668	0.538	1.34E-06	4
ERH	7.98E-11	0.286297022	0.703	0.595	1.64E-06	4
ACAT2	8.58E-11	0.307444933	0.703	0.546	1.77E-06	4
HNRNPA2B1	8.68E-11	0.269754102	0.841	0.774	1.79E-06	4
SORD	1.87E-10	0.26854699	0.728	0.589	3.85E-06	4
MDK	2.49E-10	0.256449667	0.759	0.596	5.13E-06	4
YWHAQ	3.13E-10	0.267096152	0.724	0.62	6.45E-06	4
PA2G4	3.59E-10	0.261398645	0.659	0.544	7.40E-06	4
ANAPC11	4.49E-10	0.258488454	0.806	0.687	9.25E-06	4
RRP7A	4.57E-10	0.275775179	0.634	0.48	9.40E-06	4
ARL6IP1	4.77E-10	0.571945993	0.759	0.626	9.82E-06	4

CLIC1	7.12E-10	0.300075211	0.884	0.782	1.47E-05	4
FKBP1A	9.24E-10	0.311603627	0.806	0.648	1.90E-05	4
IGF2BP3	1.09E-09	0.2536194	0.556	0.386	2.24E-05	4
SLC25A1	1.25E-09	0.287882239	0.711	0.596	2.57E-05	4
PRKDC	1.26E-09	0.284274527	0.586	0.465	2.60E-05	4
CCAR1	2.16E-09	0.271284432	0.56	0.393	4.44E-05	4
FDPS	2.38E-09	0.255394894	0.871	0.775	4.91E-05	4
TXNRD1	3.39E-09	0.274165758	0.681	0.52	6.98E-05	4
SLC25A5	3.62E-09	0.250563954	0.81	0.693	7.46E-05	4
PSMA3	4.58E-09	0.280621514	0.668	0.557	9.43E-05	4
C20orf27	4.94E-09	0.321234859	0.612	0.439	0.00010182	4
HNRNPR	5.56E-09	0.265327745	0.659	0.533	0.00011456	4
CPS1	9.90E-09	0.293488487	0.78	0.657	0.000203809	4
SMC3	1.08E-08	0.345382789	0.608	0.493	0.00022266	4
EIF5A	1.68E-08	0.266073106	0.681	0.53	0.000346719	4
SMC1A	2.09E-08	0.258742081	0.509	0.37	0.000431045	4
C6orf62	1.07E-07	0.269068557	0.707	0.578	0.002208619	4
ACTN4	2.95E-07	0.250874334	0.621	0.52	0.006079517	4
EIF3B	3.78E-07	0.270881818	0.655	0.523	0.007785705	4
IDI1	5.33E-07	0.262001428	0.72	0.6	0.010980412	4
LGALS1	8.48E-07	0.414295764	0.547	0.394	0.017460133	4
NOL11	1.36E-06	0.25001579	0.513	0.375	0.028040804	4
SIVA1	1.60E-06	0.253942869	0.69	0.627	0.032962538	4
DNAJA1	1.63E-06	0.266470437	0.746	0.625	0.033616747	4
MEG8	2.08E-05	0.262635726	0.565	0.447	0.428568667	4
MORF4L1	0.000117678	0.295342721	0.703	0.618	1	4
ADM	8.20E-113	1.297070557	0.718	0.149	1.69E-108	5
SERPINE1	1.52E-105	1.832680046	0.767	0.181	3.14E-101	5
NDUFA4L2	1.99E-105	1.124485606	0.705	0.139	4.09E-101	5
NDRG1	1.14E-100	1.274170073	0.85	0.29	2.35E-96	5
FAM13A	2.86E-98	0.999521899	0.784	0.232	5.88E-94	5
HSD3B7	4.02E-89	1.383044309	0.89	0.444	8.27E-85	5
SLC6A8	2.47E-81	0.486673973	0.348	0.029	5.09E-77	5
VEGFA	3.50E-81	1.169896086	0.93	0.56	7.20E-77	5
ERO1A	3.71E-76	0.925971556	0.872	0.432	7.64E-72	5
P2RX7	1.11E-74	0.542089804	0.507	0.092	2.28E-70	5
TNS1	2.93E-74	0.779328537	0.793	0.304	6.04E-70	5
EFNA1	1.25E-70	0.980758226	0.96	0.662	2.58E-66	5
IGFBP1	3.72E-69	1.108750005	0.996	0.935	7.67E-65	5
PLOD2	1.22E-67	1.017795874	0.894	0.56	2.51E-63	5
FCGBP	2.37E-67	0.273166338	0.352	0.042	4.87E-63	5
SCARB1	2.39E-64	0.891795737	0.863	0.485	4.92E-60	5
SMIM3	2.84E-64	0.670929152	0.63	0.193	5.85E-60	5
ERRFI1	3.12E-64	1.033929269	0.921	0.629	6.43E-60	5
JUN	3.28E-63	1.282755091	0.85	0.442	6.76E-59	5

NEAT1	1.02E-62	1.328087858	0.938	0.655	2.10E-58	5
HILPDA	5.06E-62	1.110888597	0.665	0.25	1.04E-57	5
INSIG2	8.44E-61	1.013377118	0.841	0.521	1.74E-56	5
GBP2	1.54E-60	0.7533087	0.833	0.402	3.18E-56	5
EFNA3	2.10E-57	0.372041771	0.493	0.116	4.32E-53	5
MAFF	2.80E-56	0.58984457	0.626	0.211	5.77E-52	5
BNIP3L	1.31E-54	0.770642454	0.797	0.42	2.70E-50	5
LDHA	3.12E-54	0.816808864	0.965	0.855	6.43E-50	5
SORL1	1.03E-53	1.025243592	0.819	0.497	2.12E-49	5
HSF4	1.97E-53	0.46569399	0.493	0.123	4.06E-49	5
PLIN2	1.76E-52	1.049896022	0.855	0.569	3.62E-48	5
SLC38A2	5.38E-52	0.857955048	0.841	0.514	1.11E-47	5
SPAG4	1.21E-51	0.411730754	0.498	0.133	2.50E-47	5
DUSP1	3.21E-51	0.902620828	0.762	0.342	6.62E-47	5
PLCB1	5.77E-51	0.741652735	0.872	0.553	1.19E-46	5
ANKRD37	6.57E-51	0.758938216	0.493	0.13	1.35E-46	5
ZNF395	4.47E-50	0.597487303	0.74	0.361	9.21E-46	5
PHF1	1.06E-49	0.607399366	0.722	0.338	2.19E-45	5
FOSL2	2.43E-49	0.409092415	0.48	0.127	5.01E-45	5
JUND	2.65E-49	0.925979097	0.934	0.691	5.45E-45	5
ETS2	9.47E-49	0.696990523	0.709	0.333	1.95E-44	5
GBE1	3.41E-48	0.664471011	0.652	0.279	7.03E-44	5
APOL1	5.79E-48	0.638863947	0.661	0.273	1.19E-43	5
MXI1	6.40E-48	0.582695725	0.758	0.416	1.32E-43	5
LPIN2	9.80E-47	0.770583153	0.859	0.559	2.02E-42	5
EGFR	1.16E-46	0.628734432	0.722	0.358	2.38E-42	5
ARG2	2.98E-46	0.311285198	0.278	0.038	6.15E-42	5
PNCK	3.17E-46	0.599368702	0.26	0.033	6.53E-42	5
SERINC2	2.43E-45	0.797576298	0.815	0.469	5.01E-41	5
PDK1	2.68E-45	0.457528586	0.586	0.213	5.53E-41	5
BHLHE40	3.41E-45	0.67521388	0.599	0.23	7.02E-41	5
SORBS1	5.79E-45	0.453825473	0.577	0.217	1.19E-40	5
ATF5	7.90E-45	0.799571797	0.93	0.728	1.63E-40	5
NEDD9	8.57E-45	0.7207571	0.714	0.33	1.76E-40	5
PPP1R3C	9.41E-44	0.88687051	0.727	0.383	1.94E-39	5
CSTB	1.57E-43	0.899397481	0.872	0.661	3.24E-39	5
GPR157	1.62E-43	0.384483306	0.441	0.119	3.33E-39	5
GPI	1.95E-43	0.690402479	0.767	0.435	4.02E-39	5
TMEM123	4.45E-43	0.625614727	0.894	0.688	9.16E-39	5
SMAD3	4.55E-43	0.493451344	0.652	0.292	9.36E-39	5
CTSB	6.36E-43	0.699548337	0.96	0.768	1.31E-38	5
MCL1	8.81E-43	0.789334434	0.872	0.566	1.81E-38	5
FUNDC2	3.36E-42	0.902896471	0.731	0.423	6.91E-38	5
SNHG7	3.80E-42	0.768015558	0.736	0.408	7.82E-38	5
SLC7A2	9.76E-42	0.682421514	0.916	0.65	2.01E-37	5

MIR210HG	1.63E-40	0.333661285	0.436	0.119	3.36E-36	5
ADGRD1	1.90E-40	0.402648672	0.366	0.086	3.92E-36	5
PLEKHA1	3.52E-40	0.544961974	0.784	0.484	7.26E-36	5
CITED2	6.15E-40	0.624825993	0.555	0.22	1.27E-35	5
BTG1	1.48E-39	0.659481878	0.881	0.653	3.05E-35	5
RAP2B	1.83E-39	0.57912803	0.665	0.316	3.77E-35	5
BACH1	2.34E-39	0.519953868	0.78	0.453	4.82E-35	5
RHOB	3.46E-39	0.99322344	0.863	0.651	7.12E-35	5
CALD1	8.57E-39	0.580581462	0.965	0.774	1.76E-34	5
GAPDH	2.93E-38	0.635774973	0.982	0.92	6.04E-34	5
WSB1	3.05E-38	0.640265683	0.811	0.514	6.28E-34	5
IER3	2.27E-37	1.130442679	0.793	0.465	4.67E-33	5
CSRNP1	4.20E-37	0.367642723	0.344	0.082	8.65E-33	5
ZBTB20	7.33E-37	0.617981522	0.833	0.571	1.51E-32	5
VPS37A	9.25E-37	0.443540527	0.634	0.304	1.91E-32	5
COL27A1	1.36E-36	0.569619972	0.696	0.368	2.79E-32	5
MEAF6	1.92E-36	0.536502598	0.802	0.507	3.96E-32	5
PTGR1	2.35E-36	1.169773049	0.916	0.746	4.84E-32	5
PPARA	2.55E-36	0.568632696	0.793	0.509	5.24E-32	5
CEBPB	3.30E-36	0.81831291	0.907	0.697	6.80E-32	5
VKORC1	4.94E-36	0.632404006	0.819	0.59	1.02E-31	5
INSR	1.31E-35	0.59371657	0.775	0.466	2.69E-31	5
ERGIC1	1.33E-35	0.512654494	0.771	0.467	2.74E-31	5
PPP1R15A	1.62E-35	0.409488937	0.454	0.146	3.33E-31	5
SNRNP48	3.45E-35	0.43675008	0.674	0.364	7.11E-31	5
WEE1	1.01E-34	0.580370054	0.696	0.369	2.08E-30	5
KLF6	1.21E-34	0.763181215	0.837	0.549	2.49E-30	5
ANKRD12	1.84E-34	0.552557992	0.837	0.574	3.78E-30	5
IMPAD1	2.10E-34	0.490660224	0.683	0.381	4.33E-30	5
ELF3	2.21E-34	0.672151789	0.727	0.38	4.54E-30	5
LEAP2	2.24E-34	0.749792938	0.872	0.599	4.62E-30	5
CEBPD	3.10E-34	0.760383651	0.872	0.662	6.38E-30	5
FNDC3B	6.14E-34	0.616305278	0.841	0.566	1.26E-29	5
FAM210A	9.14E-34	0.471644186	0.621	0.303	1.88E-29	5
PHKA2	1.81E-33	0.44256398	0.692	0.38	3.72E-29	5
ADIPOR2	3.33E-33	0.428768593	0.621	0.311	6.85E-29	5
FXYD1	5.69E-33	0.487976082	0.749	0.394	1.17E-28	5
MT2A	1.28E-32	2.039960679	0.85	0.631	2.63E-28	5
NGLY1	1.33E-32	0.510053076	0.621	0.321	2.73E-28	5
ID2	1.40E-32	0.74958028	0.841	0.621	2.89E-28	5
MAFK	2.44E-32	0.256510652	0.304	0.07	5.02E-28	5
ZFP36	3.82E-32	0.356042714	0.471	0.176	7.87E-28	5
FBXO17	5.14E-32	0.395528396	0.648	0.345	1.06E-27	5
RNF149	5.89E-32	0.402572596	0.775	0.493	1.21E-27	5
SESTD1	7.60E-32	0.572179798	0.727	0.453	1.56E-27	5

CPEB4	9.49E-32	0.409509369	0.586	0.272	1.95E-27	5
SLC11A1	1.16E-31	0.340306257	0.282	0.063	2.39E-27	5
TMCC1	1.50E-31	0.581620216	0.767	0.492	3.09E-27	5
AGPAT2	2.54E-31	0.506274748	0.48	0.191	5.23E-27	5
SLC9A3R1	2.82E-31	0.577836705	0.819	0.626	5.80E-27	5
HSPA1A	8.50E-31	1.152820253	0.899	0.784	1.75E-26	5
EIF1	1.32E-30	0.622175337	0.956	0.884	2.73E-26	5
EPAS1	2.45E-30	0.468918142	0.789	0.511	5.05E-26	5
GADD45B	3.96E-30	0.698618544	0.586	0.276	8.16E-26	5
RNMT	4.09E-30	0.514979116	0.797	0.537	8.43E-26	5
PGK1	4.26E-30	0.715553335	0.806	0.601	8.77E-26	5
NSMF	4.84E-30	0.561167797	0.454	0.178	9.97E-26	5
SAT1	1.52E-29	0.672857007	0.841	0.59	3.13E-25	5
DUSP8	1.57E-29	0.259901747	0.374	0.113	3.24E-25	5
NADSYN1	1.98E-29	0.312172658	0.52	0.227	4.08E-25	5
SBDS	2.02E-29	0.437487084	0.815	0.575	4.16E-25	5
FNIP1	2.36E-29	0.367258998	0.608	0.319	4.87E-25	5
CXXC5	3.74E-29	0.423742876	0.718	0.458	7.70E-25	5
DDIT3	5.98E-29	0.433464416	0.568	0.279	1.23E-24	5
PNRC1	1.33E-28	0.683585321	0.934	0.758	2.74E-24	5
CNOT11	1.61E-28	0.383998391	0.7	0.431	3.32E-24	5
ZFP36L1	2.44E-28	0.663633314	0.846	0.61	5.02E-24	5
ABHD2	5.84E-28	0.37687626	0.753	0.438	1.20E-23	5
PIK3AP1	6.04E-28	0.468842134	0.559	0.281	1.24E-23	5
RSBN1	1.27E-27	0.375769959	0.546	0.258	2.61E-23	5
TM2D3	2.99E-27	0.493590761	0.656	0.403	6.16E-23	5
ASGR1	3.10E-27	0.471890335	0.952	0.771	6.38E-23	5
EIF4A2	4.76E-27	0.53768303	0.903	0.73	9.81E-23	5
TRIB3	5.43E-27	0.861995107	0.573	0.304	1.12E-22	5
RNF19A	5.79E-27	0.392855116	0.581	0.307	1.19E-22	5
DIO1	8.33E-27	0.569781453	0.573	0.313	1.72E-22	5
ANGPTL8	1.45E-26	0.573296422	0.656	0.38	2.98E-22	5
MPZL2	1.46E-26	0.349284664	0.568	0.287	3.00E-22	5
SLC39A1	1.60E-26	0.39067783	0.793	0.573	3.30E-22	5
ABCA5	1.98E-26	0.360582475	0.551	0.277	4.07E-22	5
DNAJC12	3.11E-26	0.273250294	0.542	0.245	6.41E-22	5
MASP1	4.06E-26	0.282165223	0.454	0.184	8.37E-22	5
SPAG9	5.87E-26	0.394398122	0.67	0.399	1.21E-21	5
TNFRSF12A	6.55E-26	0.42175972	0.652	0.343	1.35E-21	5
MAP3K2	7.86E-26	0.388121295	0.749	0.498	1.62E-21	5
NEDD4L	7.91E-26	0.39545214	0.665	0.415	1.63E-21	5
6-Mar	9.67E-26	0.36313881	0.727	0.465	1.99E-21	5
CCNL1	1.05E-25	0.508047688	0.78	0.522	2.16E-21	5
NUPR1	1.08E-25	0.583800721	0.903	0.741	2.23E-21	5
TPI1	1.09E-25	0.599676467	0.863	0.714	2.24E-21	5

LPCAT1	1.15E-25	0.471653914	0.529	0.259	2.36E-21	5
CP	1.21E-25	0.587923878	0.965	0.867	2.50E-21	5
GSE1	1.99E-25	0.425071401	0.573	0.309	4.10E-21	5
ZCCHC24	2.25E-25	0.423873782	0.595	0.319	4.64E-21	5
PNISR	2.33E-25	0.426711096	0.811	0.569	4.80E-21	5
PPP1R3G	2.41E-25	0.423590153	0.573	0.287	4.97E-21	5
AC087482.1	2.67E-25	0.407068197	0.529	0.261	5.50E-21	5
EGFR-AS1	3.01E-25	0.281690002	0.419	0.168	6.20E-21	5
VWCE	3.45E-25	0.413268344	0.559	0.301	7.10E-21	5
HLA-E	5.48E-25	0.51804397	0.753	0.547	1.13E-20	5
PCSK6	5.57E-25	0.34456608	0.515	0.247	1.15E-20	5
HERPUD1	6.06E-25	0.527345436	0.811	0.586	1.25E-20	5
LMAN1	8.68E-25	0.481419814	0.859	0.657	1.79E-20	5
NFIL3	9.77E-25	0.388349787	0.555	0.287	2.01E-20	5
CERS2	1.55E-24	0.417778862	0.912	0.689	3.19E-20	5
KLHL24	2.04E-24	0.441825583	0.696	0.448	4.19E-20	5
CD68	2.30E-24	0.256118702	0.537	0.258	4.74E-20	5
BCAR1	2.35E-24	0.310775057	0.383	0.141	4.84E-20	5
DUSP3	2.51E-24	0.324850673	0.665	0.396	5.16E-20	5
GCC2	3.01E-24	0.410733163	0.753	0.511	6.20E-20	5
HSPA1B	3.09E-24	0.734422281	0.555	0.285	6.37E-20	5
TRIM56	3.68E-24	0.527499257	0.687	0.436	7.58E-20	5
KDSR	4.02E-24	0.316478872	0.67	0.425	8.27E-20	5
KANK1	4.48E-24	0.454660106	0.797	0.563	9.22E-20	5
GDF15	6.01E-24	1.016081889	0.489	0.232	1.24E-19	5
CPT1A	1.00E-23	0.45326596	0.63	0.371	2.06E-19	5
DSP	1.06E-23	0.609509579	0.877	0.638	2.19E-19	5
STAT3	1.21E-23	0.404420464	0.678	0.421	2.50E-19	5
DDIT4	1.45E-23	0.512772999	0.436	0.185	2.98E-19	5
RORA	1.84E-23	0.373433806	0.678	0.407	3.79E-19	5
PHF21A	2.53E-23	0.29145678	0.37	0.135	5.21E-19	5
DARS	2.82E-23	0.41459438	0.793	0.587	5.80E-19	5
RAPGEF1	2.97E-23	0.304977735	0.449	0.191	6.11E-19	5
IFRD1	5.06E-23	0.390468817	0.476	0.224	1.04E-18	5
CDO1	6.60E-23	0.360555161	0.7	0.49	1.36E-18	5
PALM2	8.87E-23	0.266307821	0.352	0.125	1.83E-18	5
DNAJB9	9.23E-23	0.695038809	0.744	0.49	1.90E-18	5
RETREG2	1.04E-22	0.364214632	0.78	0.566	2.14E-18	5
UBE2B	1.07E-22	0.396903635	0.789	0.58	2.21E-18	5
LRRFIP1	1.20E-22	0.356039874	0.815	0.58	2.47E-18	5
CREBRF	1.52E-22	0.425088621	0.608	0.346	3.13E-18	5
PURA	1.69E-22	0.35526703	0.683	0.432	3.49E-18	5
FN1	2.29E-22	0.420085822	0.978	0.819	4.72E-18	5
UBC	2.78E-22	0.527076871	0.938	0.831	5.73E-18	5
PHF3	3.51E-22	0.427987876	0.811	0.558	7.23E-18	5

PNPLA2	4.96E-22	0.306398293	0.718	0.479	1.02E-17	5
PRKAB2	5.74E-22	0.431311366	0.802	0.579	1.18E-17	5
BAAT	8.11E-22	0.43393934	0.564	0.306	1.67E-17	5
KDM3A	1.30E-21	0.339373771	0.493	0.247	2.67E-17	5
SAT2	1.55E-21	0.406335348	0.828	0.628	3.19E-17	5
PPP2CB	2.28E-21	0.360208213	0.678	0.458	4.69E-17	5
C4orf3	2.53E-21	0.343588795	0.811	0.635	5.21E-17	5
TMEM37	3.38E-21	0.329517554	0.643	0.417	6.96E-17	5
TAF1D	3.48E-21	0.394832255	0.775	0.569	7.18E-17	5
TPST2	3.82E-21	0.389828873	0.621	0.396	7.87E-17	5
CAMLG	4.26E-21	0.264469584	0.639	0.406	8.78E-17	5
HPN	6.13E-21	0.354354499	0.617	0.362	1.26E-16	5
MKRN1	6.87E-21	0.304406002	0.7	0.473	1.41E-16	5
KMT2E	8.19E-21	0.306749748	0.784	0.564	1.69E-16	5
MUC20-OT1	8.60E-21	0.380800057	0.727	0.492	1.77E-16	5
SPTBN1	8.65E-21	0.399616785	0.899	0.649	1.78E-16	5
MED13L	8.95E-21	0.44317929	0.608	0.384	1.84E-16	5
MYH9	9.73E-21	0.357988655	0.731	0.486	2.00E-16	5
DAZAP2	1.02E-20	0.300803323	0.678	0.455	2.10E-16	5
MALAT1	1.06E-20	0.488843741	0.974	0.907	2.19E-16	5
AP002387.2	1.17E-20	0.355147588	0.515	0.282	2.42E-16	5
SHMT2	1.33E-20	0.351519929	0.802	0.584	2.75E-16	5
ATXN1	1.57E-20	0.435864245	0.652	0.423	3.24E-16	5
APOL2	1.58E-20	0.262117343	0.498	0.253	3.26E-16	5
ZCCHC2	1.63E-20	0.307065392	0.683	0.427	3.35E-16	5
CRKL	1.73E-20	0.302431718	0.546	0.3	3.56E-16	5
TPP1	1.78E-20	0.308307937	0.802	0.578	3.66E-16	5
IRS2	1.83E-20	0.416586979	0.899	0.643	3.78E-16	5
CEMIP2	2.67E-20	0.397668061	0.559	0.318	5.50E-16	5
UBE2D2	2.70E-20	0.303672857	0.705	0.504	5.57E-16	5
RAF1	2.88E-20	0.361070228	0.736	0.506	5.94E-16	5
ERN1	3.69E-20	0.300629713	0.427	0.194	7.61E-16	5
EEF2	3.80E-20	0.371660845	0.96	0.798	7.82E-16	5
SELENOK	4.27E-20	0.421107464	0.841	0.688	8.80E-16	5
NPC2	4.30E-20	0.339376424	0.762	0.573	8.85E-16	5
ALKBH5	4.74E-20	0.305477267	0.524	0.293	9.77E-16	5
TOR1AIP2	4.92E-20	0.376898882	0.718	0.494	1.01E-15	5
CERK	5.39E-20	0.394858989	0.612	0.375	1.11E-15	5
EGLN1	5.73E-20	0.340457742	0.564	0.327	1.18E-15	5
TNRC6B	6.11E-20	0.341117527	0.775	0.526	1.26E-15	5
EPB41L4A-AS1	6.18E-20	0.28760961	0.599	0.378	1.27E-15	5
PTTG1IP	6.92E-20	0.354316884	0.705	0.499	1.43E-15	5
SCD	8.12E-20	0.491257117	0.965	0.795	1.67E-15	5
SH3BP4	8.13E-20	0.541088431	0.608	0.379	1.67E-15	5

STAU1	8.23E-20	0.364167236	0.828	0.641	1.69E-15	5
BRI3	1.14E-19	0.316610049	0.982	0.852	2.36E-15	5
DHRS3	1.45E-19	0.295914787	0.731	0.529	2.98E-15	5
CYTOR	1.49E-19	0.328327752	0.551	0.304	3.06E-15	5
LONP2	1.55E-19	0.324883338	0.696	0.473	3.19E-15	5
CYTH1	2.10E-19	0.298086958	0.524	0.278	4.32E-15	5
SUN2	2.39E-19	0.277445909	0.564	0.312	4.93E-15	5
GPX3	2.80E-19	0.538204342	0.828	0.692	5.76E-15	5
KTN1	2.94E-19	0.28338618	0.868	0.648	6.06E-15	5
SLC5A6	3.29E-19	0.405742165	0.709	0.477	6.77E-15	5
YPEL3	3.53E-19	0.301307519	0.687	0.481	7.26E-15	5
TRAM1	3.70E-19	0.323897362	0.744	0.531	7.61E-15	5
SCAF11	6.76E-19	0.315760222	0.762	0.522	1.39E-14	5
VMP1	7.02E-19	0.475114605	0.749	0.521	1.45E-14	5
CYB5R3	7.02E-19	0.443266506	0.705	0.506	1.45E-14	5
PPP2R5A	1.01E-18	0.346075104	0.767	0.584	2.09E-14	5
F10	1.19E-18	0.373509717	0.912	0.747	2.45E-14	5
SARAF	1.21E-18	0.316263438	0.775	0.611	2.49E-14	5
RASD1	1.31E-18	0.479323473	0.256	0.079	2.70E-14	5
RYBP	1.33E-18	0.419545644	0.648	0.433	2.73E-14	5
MT1X	1.53E-18	2.163099123	0.542	0.351	3.15E-14	5
NOP53	2.24E-18	0.332634233	0.85	0.711	4.60E-14	5
SMAD5	2.50E-18	0.324810466	0.59	0.363	5.15E-14	5
RHOU	2.87E-18	0.31517173	0.577	0.368	5.90E-14	5
SELENOM	3.05E-18	0.408538857	0.692	0.476	6.28E-14	5
SRRM2	3.17E-18	0.380528234	0.855	0.629	6.54E-14	5
EIF4B	3.34E-18	0.28522374	0.899	0.745	6.88E-14	5
ZBTB38	3.49E-18	0.368595983	0.714	0.511	7.18E-14	5
RB1CC1	4.23E-18	0.306378597	0.731	0.525	8.71E-14	5
TRIM8	6.55E-18	0.32115808	0.524	0.308	1.35E-13	5
PDXK	6.57E-18	0.293757678	0.63	0.416	1.35E-13	5
NAGS	7.13E-18	0.283399563	0.48	0.246	1.47E-13	5
ENAH	7.30E-18	0.328073022	0.643	0.427	1.50E-13	5
HDAC5	9.31E-18	0.330312879	0.52	0.297	1.92E-13	5
WNK1	1.01E-17	0.283996518	0.74	0.524	2.08E-13	5
DST	1.04E-17	0.50829376	0.837	0.61	2.14E-13	5
SERP1	1.09E-17	0.333499508	0.859	0.714	2.25E-13	5
EAF1	1.18E-17	0.285504777	0.511	0.283	2.44E-13	5
MAPK14	1.62E-17	0.293442699	0.643	0.447	3.34E-13	5
PCK1	1.72E-17	0.596353431	0.797	0.583	3.54E-13	5
ST6GAL1	1.74E-17	0.401662953	0.722	0.518	3.59E-13	5
H1F0	1.77E-17	0.336847955	0.841	0.663	3.64E-13	5
ZNF331	1.83E-17	0.332058846	0.621	0.407	3.76E-13	5
TFRC	2.29E-17	0.313653057	0.502	0.28	4.72E-13	5
LRP1	2.37E-17	0.308610709	0.674	0.464	4.88E-13	5

UGP2	2.47E-17	0.323006408	0.815	0.649	5.10E-13	5
SERINC3	2.51E-17	0.324115273	0.78	0.595	5.17E-13	5
TSC22D1	2.53E-17	0.285701589	0.696	0.48	5.20E-13	5
DENND4A	3.17E-17	0.269809976	0.524	0.305	6.54E-13	5
AP3S1	3.32E-17	0.251488168	0.749	0.566	6.83E-13	5
APCS	3.32E-17	0.561918561	0.731	0.492	6.83E-13	5
IER2	4.16E-17	0.380549403	0.436	0.219	8.57E-13	5
TMEM140	4.76E-17	0.29767584	0.533	0.314	9.80E-13	5
NR3C1	5.96E-17	0.278612966	0.661	0.446	1.23E-12	5
CCNY	7.04E-17	0.353166281	0.621	0.433	1.45E-12	5
ZFAND5	7.50E-17	0.339219033	0.63	0.418	1.55E-12	5
TNIP1	8.00E-17	0.253006047	0.634	0.409	1.65E-12	5
NFKBIA	8.15E-17	0.495828414	0.758	0.534	1.68E-12	5
TBL1XR1	9.98E-17	0.301305279	0.67	0.468	2.05E-12	5
IRS1	1.02E-16	0.429576605	0.74	0.524	2.11E-12	5
CYP4F3	1.26E-16	0.330392234	0.63	0.41	2.58E-12	5
MKLN1	1.40E-16	0.330283931	0.687	0.487	2.89E-12	5
NR1D2	1.49E-16	0.337340745	0.524	0.315	3.07E-12	5
RBPMS	1.52E-16	0.255386211	0.626	0.409	3.13E-12	5
LAMP2	1.55E-16	0.295296812	0.784	0.585	3.19E-12	5
CLDN1	1.85E-16	0.346783925	0.819	0.608	3.81E-12	5
XIAP	2.02E-16	0.259039126	0.678	0.438	4.16E-12	5
DDX3X	2.40E-16	0.334821978	0.621	0.43	4.95E-12	5
CDK19	2.47E-16	0.273759115	0.471	0.259	5.08E-12	5
PPP4R2	2.47E-16	0.352457156	0.762	0.562	5.09E-12	5
SCARB2	2.63E-16	0.262201304	0.595	0.4	5.43E-12	5
HIPK1	2.85E-16	0.264036693	0.564	0.345	5.87E-12	5
RBM39	3.40E-16	0.387507431	0.894	0.67	6.99E-12	5
RBM33	3.67E-16	0.25250026	0.634	0.426	7.57E-12	5
TIPARP	3.76E-16	0.251750726	0.414	0.216	7.75E-12	5
SOD2	3.77E-16	0.475911405	0.863	0.687	7.77E-12	5
SIAH1	4.34E-16	0.272720705	0.449	0.246	8.94E-12	5
EIF4EBP2	4.66E-16	0.250425842	0.775	0.569	9.59E-12	5
MGAT4B	4.82E-16	0.32832226	0.837	0.611	9.93E-12	5
ROCK1	5.03E-16	0.282889749	0.771	0.571	1.04E-11	5
BRWD1	5.61E-16	0.250616626	0.604	0.383	1.16E-11	5
CCDC198	5.82E-16	0.266392708	0.317	0.133	1.20E-11	5
NKTR	6.03E-16	0.31808774	0.784	0.556	1.24E-11	5
PPP1R16A	7.18E-16	0.272673167	0.744	0.571	1.48E-11	5
ESYT2	7.60E-16	0.295250755	0.52	0.314	1.56E-11	5
DNAJB1	7.89E-16	0.277786846	0.559	0.347	1.63E-11	5
SERINC1	8.09E-16	0.255661818	0.705	0.5	1.67E-11	5
NEU1	8.29E-16	0.263956108	0.767	0.569	1.71E-11	5
CCDC107	1.01E-15	0.292180955	0.661	0.486	2.07E-11	5
MTDH	1.01E-15	0.307847004	0.793	0.59	2.07E-11	5

SMIM13	1.25E-15	0.300785176	0.533	0.325	2.58E-11	5
MTCH1	1.29E-15	0.295866684	0.846	0.654	2.65E-11	5
ENO1	1.37E-15	0.5069316	0.907	0.769	2.83E-11	5
ACADVL	1.66E-15	0.325605864	0.736	0.54	3.43E-11	5
SQSTM1	1.70E-15	0.288689565	0.89	0.733	3.49E-11	5
ZFAS1	1.73E-15	0.360950958	0.828	0.665	3.55E-11	5
RBCK1	1.95E-15	0.261278454	0.718	0.537	4.02E-11	5
ZNF503	2.26E-15	0.254902406	0.423	0.218	4.66E-11	5
AGFG2	2.27E-15	0.313468122	0.595	0.411	4.69E-11	5
ORAI3	2.28E-15	0.276423688	0.656	0.474	4.69E-11	5
DNAJB11	2.30E-15	0.395942606	0.696	0.505	4.74E-11	5
SSR4	2.32E-15	0.348095176	0.828	0.655	4.79E-11	5
CEBPG	2.57E-15	0.366223014	0.564	0.371	5.30E-11	5
ORMDL3	2.80E-15	0.351426591	0.762	0.573	5.77E-11	5
RAB6A	3.20E-15	0.250620414	0.612	0.421	6.59E-11	5
TMBIM6	3.31E-15	0.312497767	0.947	0.775	6.83E-11	5
KMT2C	3.78E-15	0.29780108	0.678	0.488	7.79E-11	5
NONO	3.98E-15	0.277801336	0.824	0.626	8.21E-11	5
TMBIM1	4.05E-15	0.290552928	0.626	0.423	8.34E-11	5
QKI	4.12E-15	0.278882838	0.634	0.436	8.48E-11	5
HNRNPH1	4.32E-15	0.284354241	0.762	0.533	8.90E-11	5
HIST1H2BN	4.41E-15	0.299771753	0.467	0.26	9.08E-11	5
SMOC1	5.16E-15	0.333213725	0.515	0.332	1.06E-10	5
XIST	5.17E-15	0.259762075	0.85	0.588	1.07E-10	5
NFKBIL1	5.25E-15	0.280256615	0.559	0.371	1.08E-10	5
GNL1	6.62E-15	0.293980265	0.555	0.364	1.36E-10	5
CHD1L	6.76E-15	0.450678012	0.705	0.509	1.39E-10	5
N4BP2L1	7.61E-15	0.256403055	0.511	0.294	1.57E-10	5
SNX27	7.85E-15	0.27065457	0.665	0.487	1.62E-10	5
HHEX	9.32E-15	0.308500925	0.687	0.49	1.92E-10	5
SLC1A2	9.81E-15	0.310072462	0.652	0.443	2.02E-10	5
SLC3A2	9.81E-15	0.438957048	0.489	0.297	2.02E-10	5
ASH1L	1.39E-14	0.250086289	0.744	0.543	2.86E-10	5
AIP	1.40E-14	0.270416166	0.648	0.461	2.88E-10	5
PIM1	1.46E-14	0.288003504	0.696	0.486	3.00E-10	5
TMEM141	1.65E-14	0.323629391	0.744	0.577	3.40E-10	5
H6PD	1.68E-14	0.349877371	0.639	0.443	3.46E-10	5
GSDMB	1.89E-14	0.250736098	0.419	0.227	3.88E-10	5
SAR1B	1.95E-14	0.318830157	0.722	0.527	4.01E-10	5
CLK1	2.06E-14	0.258950199	0.511	0.307	4.24E-10	5
RSBN1L	2.33E-14	0.305327639	0.722	0.519	4.80E-10	5
SF3B2	2.66E-14	0.306700254	0.767	0.595	5.48E-10	5
EIF1B	2.84E-14	0.322739639	0.749	0.575	5.85E-10	5
N4BP2L2	3.13E-14	0.273994422	0.811	0.603	6.44E-10	5
IER5L	3.73E-14	0.324755696	0.515	0.317	7.69E-10	5

A1CF	3.78E-14	0.33033549	0.815	0.671	7.78E-10	5
CTSD	3.86E-14	0.28317393	0.899	0.763	7.94E-10	5
TLE1	3.90E-14	0.295016843	0.775	0.584	8.03E-10	5
ATF4	4.14E-14	0.319719302	0.758	0.573	8.54E-10	5
EPS8L2	4.21E-14	0.25055214	0.524	0.324	8.66E-10	5
NAMPT	4.58E-14	0.320096761	0.744	0.568	9.44E-10	5
UGDH	4.67E-14	0.35255672	0.789	0.6	9.61E-10	5
ZCCHC14	4.68E-14	0.345485415	0.551	0.352	9.63E-10	5
RSRP1	4.84E-14	0.285909668	0.595	0.407	9.98E-10	5
BRD2	5.02E-14	0.309941386	0.74	0.557	1.03E-09	5
PCBP2	5.14E-14	0.276204153	0.85	0.7	1.06E-09	5
CIDEB	5.78E-14	0.27889187	0.595	0.401	1.19E-09	5
PPP1CB	6.41E-14	0.268897558	0.731	0.543	1.32E-09	5
ANO6	7.45E-14	0.255772121	0.604	0.409	1.53E-09	5
WDR45B	1.12E-13	0.278666759	0.568	0.382	2.31E-09	5
TENT5A	1.16E-13	0.325374355	0.441	0.253	2.38E-09	5
SDC4	1.34E-13	0.479489645	0.736	0.545	2.76E-09	5
MIF	1.45E-13	0.328201729	0.828	0.703	2.99E-09	5
PXDC1	1.60E-13	0.363744545	0.595	0.405	3.29E-09	5
KLF11	1.63E-13	0.327449676	0.471	0.294	3.35E-09	5
SDC2	1.67E-13	0.275706344	0.841	0.688	3.45E-09	5
HBP1	1.72E-13	0.295971092	0.665	0.492	3.53E-09	5
ACOX1	2.74E-13	0.277572192	0.727	0.542	5.65E-09	5
CXADR	2.99E-13	0.281883784	0.793	0.593	6.16E-09	5
SH3BP5	3.11E-13	0.342922563	0.515	0.335	6.41E-09	5
KCMF1	3.78E-13	0.28721219	0.643	0.472	7.79E-09	5
TAPBP	4.20E-13	0.308683185	0.705	0.541	8.65E-09	5
TROVE2	4.44E-13	0.258782052	0.604	0.409	9.14E-09	5
EGR1	4.62E-13	0.296960731	0.26	0.105	9.52E-09	5
WASL	4.73E-13	0.28050869	0.749	0.578	9.74E-09	5
ZNF451	4.75E-13	0.260671039	0.621	0.427	9.78E-09	5
GNB2	4.77E-13	0.263740142	0.815	0.681	9.83E-09	5
BAG6	4.93E-13	0.265378416	0.806	0.621	1.02E-08	5
PTPRF	5.44E-13	0.271557813	0.718	0.532	1.12E-08	5
HECTD1	5.77E-13	0.26275874	0.714	0.523	1.19E-08	5
VAPA	5.84E-13	0.261035209	0.828	0.687	1.20E-08	5
CFDP1	6.83E-13	0.256446116	0.736	0.576	1.41E-08	5
CANX	6.88E-13	0.295620434	0.907	0.75	1.42E-08	5
LURAP1L	7.73E-13	0.312826916	0.581	0.401	1.59E-08	5
BRD4	8.68E-13	0.254738175	0.678	0.489	1.79E-08	5
PDCD4	8.98E-13	0.251593024	0.524	0.341	1.85E-08	5
PJA2	9.00E-13	0.256103661	0.608	0.422	1.85E-08	5
TMF1	9.01E-13	0.270053621	0.577	0.401	1.86E-08	5
MAF	9.63E-13	0.279077032	0.599	0.428	1.98E-08	5
TCEA1	1.34E-12	0.322747386	0.718	0.534	2.76E-08	5

SIVA1	1.43E-12	0.30893906	0.749	0.62	2.95E-08	5
ARL5B	1.44E-12	0.272688245	0.458	0.277	2.96E-08	5
GOLGB1	1.73E-12	0.258613685	0.718	0.512	3.56E-08	5
ARFGAP3	2.54E-12	0.252742423	0.52	0.353	5.23E-08	5
DHCR7	2.54E-12	0.323098665	0.806	0.631	5.23E-08	5
GCLC	2.62E-12	0.324384966	0.648	0.49	5.41E-08	5
PERP	2.82E-12	0.278246561	0.683	0.525	5.82E-08	5
MTMR4	2.93E-12	0.27332409	0.674	0.489	6.03E-08	5
CMTM8	3.13E-12	0.275098201	0.7	0.534	6.44E-08	5
ZNF292	3.27E-12	0.280121994	0.564	0.386	6.74E-08	5
VDAC2	3.97E-12	0.260331576	0.683	0.55	8.17E-08	5
TFPI	4.09E-12	0.281482729	0.93	0.762	8.43E-08	5
CGGBP1	5.00E-12	0.258517985	0.639	0.478	1.03E-07	5
ITGB1	5.04E-12	0.263677337	0.872	0.673	1.04E-07	5
YIPF4	5.47E-12	0.250463737	0.749	0.564	1.13E-07	5
AZGP1	5.63E-12	0.316506844	0.956	0.844	1.16E-07	5
EML4	8.65E-12	0.312203123	0.819	0.623	1.78E-07	5
ADAR	1.12E-11	0.311430236	0.78	0.569	2.31E-07	5
TTC39C	1.57E-11	0.315327622	0.648	0.482	3.23E-07	5
AGTR1	1.75E-11	0.280124417	0.617	0.457	3.61E-07	5
DDX17	1.88E-11	0.264523712	0.771	0.584	3.87E-07	5
C2	2.08E-11	0.314005281	0.956	0.811	4.28E-07	5
ELL2	2.08E-11	0.272336994	0.621	0.455	4.29E-07	5
TMED7	2.31E-11	0.259813411	0.555	0.391	4.76E-07	5
GPRC5C	2.76E-11	0.314139567	0.595	0.424	5.69E-07	5
BNIP3	3.14E-11	0.339809154	0.793	0.67	6.47E-07	5
HPS4	3.29E-11	0.273129504	0.471	0.299	6.78E-07	5
MAFB	3.74E-11	0.389697732	0.366	0.199	7.71E-07	5
PTP4A1	5.30E-11	0.426644643	0.899	0.763	1.09E-06	5
AJUBA	5.59E-11	0.272070104	0.414	0.249	1.15E-06	5
ITGA6	6.21E-11	0.303170367	0.581	0.417	1.28E-06	5
MT1E	9.67E-11	1.65063905	0.317	0.17	1.99E-06	5
SBNO1	1.00E-10	0.265489753	0.581	0.427	2.06E-06	5
NAV2	1.02E-10	0.273532004	0.529	0.363	2.10E-06	5
MAP1LC3B	1.29E-10	0.360968003	0.626	0.479	2.66E-06	5
RYK	1.51E-10	0.259915605	0.551	0.395	3.10E-06	5
TXNIP	1.66E-10	0.289724714	0.643	0.491	3.42E-06	5
UBN2	1.94E-10	0.268382299	0.542	0.376	3.99E-06	5
COL18A1	2.19E-10	0.266797364	0.718	0.564	4.51E-06	5
GOLGA4	3.03E-10	0.283088158	0.837	0.671	6.24E-06	5
ZKSCAN1	3.38E-10	0.262890788	0.802	0.615	6.97E-06	5
SLC50A1	3.46E-10	0.286747968	0.661	0.532	7.12E-06	5
MAFG	3.60E-10	0.26725044	0.568	0.408	7.41E-06	5
MAPK6	6.40E-10	0.287729406	0.604	0.457	1.32E-05	5
IER5	7.81E-10	0.273114776	0.414	0.252	1.61E-05	5

ATP1B1	8.58E-10	0.279526966	0.559	0.413	1.77E-05	5
LRG1	1.39E-09	0.269059348	0.511	0.368	2.85E-05	5
GID8	1.75E-09	0.254720006	0.648	0.514	3.61E-05	5
FNBP4	4.53E-09	0.270737045	0.493	0.356	9.34E-05	5
PUM2	5.44E-09	0.25477584	0.621	0.466	0.000111981	5
TCF25	6.24E-09	0.250173198	0.634	0.492	0.000128413	5
HP	1.01E-08	0.378706065	0.925	0.836	0.000208662	5
CAB39	1.30E-08	0.281086045	0.52	0.374	0.000267528	5
PRPF4B	1.77E-08	0.251560187	0.626	0.492	0.000363995	5
PYGL	2.03E-08	0.263131685	0.617	0.486	0.000417326	5
UGCG	2.17E-08	0.295165211	0.577	0.445	0.000446079	5
ANG	2.20E-08	0.311615624	0.722	0.591	0.000452218	5
SLC30A1	4.45E-08	0.40101601	0.493	0.38	0.000917379	5
IL6ST	6.03E-08	0.256290885	0.656	0.498	0.001242901	5
SNRNP70	6.93E-08	0.262786832	0.643	0.516	0.001426997	5
CCL20	1.11E-07	0.671793943	0.784	0.668	0.002295907	5
INTS6	1.16E-07	0.262401947	0.542	0.398	0.002396904	5
SNTB1	1.23E-07	0.258866939	0.546	0.45	0.002530998	5
MICAL3	1.65E-07	0.271156149	0.427	0.306	0.003394072	5
AMD1	2.52E-07	0.2532462	0.542	0.422	0.005184325	5
CYP3A5	5.82E-07	0.373549474	0.705	0.568	0.011990993	5
AHSG	6.56E-06	0.303544538	0.991	0.975	0.135111175	5
